# A nomenclator of extant and fossil taxa of the Valvatidae (Gastropoda, Ectobranchia)

**DOI:** 10.3897/zookeys.377.6032

**Published:** 2014-02-05

**Authors:** Gerhard Haszprunar

**Affiliations:** 1 SNSB - Zoologische Staatssammlung München, Münchhausenstr. 21, D-81247 München, Germany; 2 Dept. Biology II and GeoBio-Center of LMU Munich, Biozentrum, Großhaderner Str. 2, D-82152 Planegg, Germany

**Keywords:** Gastropoda, Valvatidae, freshwater snails, taxonomy, www-references

## Abstract

A compilation of all supra- and (infra-) specific taxa of extant and fossil Valvatidae, a group of freshwater operculate snails, is provided, including taxa initially described in this family and subsequently classified in other families, as well as names containing errors or misspellings. The extensive reference list is directly linked to the available electronic source (digital view or pdf-download) of the respective papers.

## Introduction

Georg Ritter von Frauenfeld (1864: Introduction): “Ich habe in nachfolgendem Verzeichnisse versucht, alle Namen sowohl der fossilen wie der lebenden Arten, die mir in der alten Lamark’schen Gattung *Paludina* bekannt, oder nach deren Zerfallen in die betreffenden Gattungen bis in die neueste Zeit in die Literatur eingeführt wurden, zusammen zu stellen ...... Dass ich .. somit manches todte Synonym zur Welt bringe, wird wohl nicht getadelt werden, da solche Namen fort und fort wie Irrlichter in den Sammlungen herumwandern, ohne Ruhe zu finden. Die Arbeit selbst .... lässt die bedeutenden Lücken sehen, die für mich durch die unsicheren und mir unbekannten Arten noch bestehen.”

[“In the following catalog I have tried to collect all names of the fossil and extant species, which are known to me in the old genus *Paludina* of Lamarck, or have been described after its splitting into the various genera until most recent times in the literature...... That I will give rise to certain dead synonyms will probably not be criticized, since such names are like ghost-lights migrating without rest in the collections. The work itself ...... also sheds light on the considerable gaps, which still exist due to the uncertain and unknown species”].

This citation, which is nearly 150 years old, is equally applicable to another group of freshwater operculate gastropods, the Valvatidae or valve snails. The main goal of current catalogue is to summarize all the available information on all supra- and (infra-) specific taxa of extant and fossil Valvatidae in order to facilitate future taxonomic research. This is the first such compilation for the Valvatidae since the list of Wenz (1928b), which was restricted to fossil taxa. In addition, the present contribution should also facilitate future taxonomic work of Valvatidae and other freshwater gastropods by providing direct internet links to many important older taxonomic papers.

## Part I: Supraspecific taxa in Valvatidae

### General remarks

Until recently nearly all supraspecific taxa of Valvatidae were described based on shell characters alone. However, shells of certain taxa (e.g. the wide-spread *Valvata piscinalis*) are highly variable due to ecomorphism or ontogenetic change (e.g. Baker 1928; Favre 1927, 1935; Haas 1938; Binder 1967a; Scholtz and Glaubrecht 2010, Welter-Schultes et al. 2011); further some hydrobiid species with very similar shells have been often misidentified as valvatids.

Recent molecular data cast strong doubts that any of these higher-level names (at least concerning extant taxa) represent a monophyletic clade. The only notable exception may be *Borysthenia* Lindholm, 1914, which is characterized not only by shell characters, but also by very slender (vs. broad) marginal radular teeth, which is probably an apomorphic character among the Valvatidae. On the other hand, anatomical characters of *Borysthenia naticina* are very similar to those of other valvatids (Hawe et al. 2013). In particular molecular (as well as anatomical) data are needed to clear up the supraspecific systematics of Valvatidae.

I also list here supraspecific “names” based on various types of error in order to correct the various internet listings.

### Part IA. Family-group level taxa

BORYSTHENIINAE Starobogatov, 1983 (in Sitnikova 1983)

Original source: Sitnikova 1983: 34.

Original classification: Subfamily of Valvatidae.

Type genus: *Borysthenia* Lindholm, 1914.

ECTOBRANCHIA Fischer, 1884

Original source: Fischer 1884: 652.

Remarks: Ectobranchia currently corresponds to superfamily VALVATOIDEA Gray, 1840 (first used as superfamily name by Hannibal 1912: 196). I prefer Ectobranchia in favor of Valvatoidea, because (1) Ectobranchia is a non- ranked taxon, whereas the ending “-oidea” makes Valvatoidea a superfamily (ICZN Art. 29.2), and (2) Ectobranchia cannot be confused with Valvatida resp. Valvatacea Blake, 1987 (Echinodermata - Asteroidea), a junior homonym.

+ PROVALVATIDAE Bandel, 1991

Original source: Bandel 1991: 21.

Original classification: Family of Valvatoidea.

Type genus (monotypy): + *Provalvata* Bandel, 1991.

Remarks: Provalvatidae was erected for Jurassic ectobranchs with a hyperstrophic protoconch and living in brackish water. The latter habitat is considered transitional between marine forerunners and the modern freshwater valvatids. However, many Jurassic valvatids were described from purely freshwater habitats, thus *Provalvata* cannot be an ancestor of them.

VALVATIDAE Gray, 1840

Original source: Gray 1840: 79.

Remarks: According to Bouchet and Rocroi (2005: 181) authorship of Valvatidae is sometimes (e.g. by Bandel 2010) attributed to Thompson (1840; Valvatadae) (often also cited as Thomson in online resources). However, Thompson’s paper (September 1840) appeared after Gray (March to June 1840).

### Part IB. Genus–level taxa

+ ***Aegaea*** Oppenheim, 1891 (non Oppenheim 1890: 592 – nomen nudum)

Original source: Oppenheim 1891: 462.

Original classification: Subgenus of *Valvata*.

Type species: *Valvata (Aegaea) vivipariformis* Oppenheim, 1891 by monotypy.

Remarks: (1) Note page 462, footnote: “Nachträglich sehe ich, dass *Valvata (Cincinna) sorensis* Dybowski aus dem Baikalsee sich durch ihre Verzierungen mit Längsrippen der fossilen Form einigermaßen nähert [Afterwards I see that *Valvata (Cincinna) sorensis* Dybowski from the Lake Baikal is to some extent close to this fossil form by its decorations with longitudinal ribs]“

(2) Considered to be preoccupied by *Aegea* Rambur, 1866 (ed. 2, fasc. 2, pl. 19; Lepidoptera: **Geometridae**), therefore replaced by *Aegionia* Cossmann, 1921. However, the Code (ICZN Art. 56.2) now accepts one–letter differences for genus–group names.

+ ***Aegionia*** Cossmann, 1921

Original source: Cossmann 1921: 180.

Original classification: Subgenus of *Valvata*.

Type species: *Valvata (Aegaea) vivipariformis* Oppenheim, 1891 by original designation.

Remarks: *Aegionia* Cossmann, 1921 was expressly established as a new replacement name for *Aegaea* Oppenheim, 1891 in the belief that *Aegea* Rambur, 1866 (Lepidoptera) would be a senior homonym (which is not the case, however, see for *Aegaea* Oppenheim, 1891).

***Alienella*** Starobogatov & Zatravkin, 1985

Original source: Starobogatov and Zatravkin 1985: 1156.

Original classification: Sectio (“sect.n.”) of subgenus *Sibirovalvata* in genus *Cincinna*.

Type species: *Valvata aliena* Westerlund, 1877 by original designation.

+ ***Amplovalvata*** Yen, 1952

Original source: Yen 1952: 21, 27, 32, 33 (name only), 39 (definition).

Original classification: Genus of Valvatidae.

Type species: + *Amplovalvata cyclostoma* Yen, 1952 by original designation.

***Andrusovia*** Brusina, 1902 (in Westerlund 1902)

Original source: Westerlund 1902: 133.

Original classification: Genus of Valvatidae (still listed there e.g. in ION).

Type species: *Andrusovia dybowskii* Brusina, 1902 (in Westerlund 1902) by original designation by Brusina (MS).

Remarks: Westerlund (1902: 133) made “Brusina (MS)“ responsible for the genus name, thus the requirement of ICZN Art. 50.1.1 is fulfilled. Currently (e.g. Kantor et al. 2011: 38) classified among the **Hydrobiidae – Belgrandiinae**.

“***Andrussovi***” mentioned in Dybowski and Grochmalicki (1917: 51)

Misspelling of *Andrusovia* Brusina in Westerlund, 1902, not *Andrussovia* Derjavin, 1927 (Crustacea: Amphipoda).

+ ***Aphanotylus*** Brusina, 1894

Original source: Brusina 1894: 179 (name only), 182 (definition).

Original classification: Genus of Valvatidae.

Type species: + *Aphanotylus cossmanni* Brusina, 1894 by original designation.

Remarks: Replacement name for *Pachystoma* Sandberger, 1875 (see there), which was preoccupied by *Pachystoma* Guilding, 1828 [**Ampullariidae**]. However, Oppenheim (1892) previously proposed *Stiphrostoma* as a replacement name for *Pachystoma* Sandberger, 1875 (see also Bandel and Riedel 1994: 22).

+ ***Ariomphalus*** Bandel & Riedel, 1994

Original source: Bandel and Riedel 1994: 22.

Original classification: Genus of Valvatidae.

Type species: + *Pachystoma varicatum* Tausch, 1886 by original designation.

***Atropidina*** Lindholm, 1906

Original source: Lindholm 1906: 190.

Original classification: Subgenus of *Valvata* Müller, 1773.

Type species: “*Valvata pulchella* Stud.” by original designation.

Remarks: Lindholm (1906) did not provide more detailed information that could distinguish Studer’s 1789 usage (= *Valvata piscinalis* (Müller 1774)) from that of Studer 1820 (= *Valvata studeri* Boeters & Falkner, 1998). Studer (1820) just used the 1789 name and did not establish another new name in 1820 - and for those authors who argue that he did, there is no indication that Lindholm (1906) meant the 1820 usage or name. This problem needs a ruling by the ICZN to designate *Valvata studeri* Boeters & Falkner, 1998 as type under the plenary powers in ordert o correspond tot he modern understanding of *Atropidina* as being used for what is now known as *Valvata studeri* and related species.

***Biwakovalvata*** Starobogatov, 1983 (in Sitnikova 1983)

Original source: Sitnikova 1983: 35.

Original classification: Subgenus of *Megalovalvata* Lindholm, 1906.

Type species: *Valvata (Cincinna) biwaensis* Preston, 1916 by original designation.

***Borysthenia*** Lindholm, 1914

Original source: Lindholm 1914: 167.

Original classification: Genus of Valvatidae.

Type species: *Valvata jelskii* Crosse, 1863 by original designation by Bourguignat (1877).

Remarks: Replacement name for preoccupied *Jelskia* Bourguignat, 1877, non Taczanowski, 1871: 128 (Arachnida: Araneae). This is the only genus characterized by current taxonomists also by radular characters (slender marginals), whereas the usually stated ovovivipary is at least doubtful (Hawe et al. 2013).

**“*Cicinna*“** mentioned in Picaglia (1892: 188).

Misspelling of *Cincinna*.

**“*Cinciana*”** mentioned in Oppenheim (1891: 462).

Misspelling of *Cincinna*.

**“*Cincinna*”** mentioned in Menke (1848)

Original source: Hübner 1810: 56 (Zwei Briefe, I), *fide* Menke (1848: 55).

Type species: *Nerita piscinalis* Müller, 1774 [according to Menke (1848)].

Remarks: This name is usually attributed to Hübner (1810). However, Kennard and Woodward (1926: 27: “teste Menke 1848”) doubted the validity of this name. According to Welter-Schultes (2012a: 42; Welter-Schultes et al. 2011: 10) and “animal base” (checked Sept. 2013; see http://www.animalbase.uni-goettingen.de/zooweb/servlet/AnimalBase/home/genustaxon?id=3710) the name *Cincinna* currently is not available and the name cannot be used under the Code: “Attributed to Hübner 1810, appeared in a letter, obviously unpublished, not preserved, not even its title is known. Falkner et al. (2002: 93) regarded this name as available without providing evidence that (1) Hübner’s letter was published work under ICZN Art. 8 and 9 (both Menke 1848: 55 and Férussac [1821–1822]: 83 (folio p. 87) did not state that Hübner’s letter was printed), and (2) that the name was made available by meeting the conditions in ICZN Art. 11 and 12 (Menke mentioned the name, but no constraints to assume that Hübner had combined it with a description or indication). The assumption, that Hübner might have employed it for *Valvata piscinalis* is arbitrary and unsubstatiated. Falkner et al.’ 2002 view is not tenable. *Cincinna* was not mentioned by Férussac [1821–1822] who stated “[Hübner] a publié deux lettres”. Menke (1848: 55–56) also talked of letters by Hübner, one letter with 2 pages, the other one with 4 pages. Both sources did not provide evidence that these letters were printed and published for the permanent scientific record, which would qualify them as published under ICZN Art. 8 and 9. Menke (1848: 56) mentioned a name “*Cincinna* (= *Valvata*)” without providing evidence that *Cincinna* was made available by meeting the conditions in ICZN Art. 11 and 12. Menke (1848) did not make it available”.

***Cincinna*** Mörch, 1864

Original source: Mörch 1864: 321 (referring to Hübner).

Original classification: subgenus of *Valvata*.

Type species: not specified. Mörch included *Valvata piscinalis* Müller, 1774, *Valvata Pusilla* (Müller, 1774) and *Valvata antiqua* (Morris, 1843).

Remarks: As outlined above *Cincinna* attributed to Hübner (1810) or Menke (1848) is not available. On the other hand, *Cincinna* has not been used elsewhere in zoology. According to Wenz (1928b: 2422) Mörch (1864: 321) first used the name *Cincinna* with a reliable diagnosis and thus made it available. I have not found an earlier diagnosis of *Cincinna*.

**“*Cincinnaa*”, “*Cincuma*”** in Schlesch (1925: 91, 92).

Misspelling of *Cincinna*.

**“*Concinna*** Hubn.” mentioned in Adams and Adams (1854: 343).

“*Concinna* Hübner, 1810“ mentioned in Lindholm (1906: 190, footnote).

Misspelling of *Cincinna* in both cases.

***Costovalvata*** Poliński, 1929

Original source: Poliński 1929: 65, 133 (name only), 137 (definition).

Original classification: Subgenus of *Valvata*.

Type species: *Valvata (Costovalvata) hirsutecostata* Poliński, 1929 by original designation.

***Cristatiana*** Servain, 1888

Original source: Servain 1888: 309.

Type species: *Valvata planorbulina* Paladilhe, 1867 by monotypy.

Remarks: Servain (1888) cited *Valvata cristata* Müller, 1774, but did not give a direct and unambiguous indication that *Valvata cristata* was to be included in *Cristatiana*.

+ ***Fascivalvata*** Pan, 1982

Original source: Pan 1982b: 89 [Chinese text], 108 [English text].

Original classification: Genus of **Euomphalidae**.

Type species: Fascivalvata minuta Pan, 1982 by original designation.

Remarks: Although the name suggests valvatid affinities, this genus belongs to the Euomphalidae and has never be classified among the Valvatidae.

***Globuliana*** Paladilhe, 1866

Original source: Paladilhe 1866–1869: 26 (resp. Paladilhe 1866: 169).

Original classification: Genus of Valvatidae.

Type species: *Valvata moquiniana* Dupuy, 1851 by subsequent designation under ICZN Art. 70.3 by Kadolsky (2008: 116).

***Gyrorbis*** Fitzinger, 1833 (non *Gyrorbis* Moquin-Tandon, 1856: 423).

Original source: Fitzinger 1833: 117.

Type species: *Valvata cristata* Müller, 1774 by subsequent designation by Herrmannsen (1847: 495).

Remarks: (1) Neither a diagnosis of the genusname nor a type species were provided by Fitzinger (1833), but since the name was established before 1931 with species included, the name is available. In addition, *Gyrorbis* Moquin-Tandon, 1856 was replaced by *Wüstia* Honigmann, 1909: 297) because of the earlier name by Fitzinger (1833).

(2) *Valvata spirorbis* Draparnaud, 1809 is often regarded as junior synonym of *Valvata cristata* Müller, 1774, but has been considered as separate species by recent Russian authors, e.g. Anistratenko and Chernogorenko (1989), Anistratenko and Anistratenko (2001: 135) and Kantor et al. (2011: 74).

(3) According to Dall (1905: 85) “This subgenus [*Gyrorbis*] was called ‘*Gyrorbis* Agassiz’, by Moquin Tandon [1856: 423], but Agassiz never proposed any such genus or group”.

+ ***Heterovalvata*** Munier-Chalmas, 1879

Original source: Munier-Chalmas 1879: 34.

Original classification: Genus of Valvatidae.

Type species: + *Heterovalvata disjuncta* Dollfus, 1877 by monotypy (*Heterovalvata delessei* Munier-Chalmas, 1879 was originally included as a second species, but this was a nomen nudum).

+ ***Jekeliusiana*** Gozhik, 2002

Original source: Gozhik 2002: 49.

Original classification: Subgenus of *Valvata*.

Type species: + *Valvata (Jekeliusiana) oecsensis halavatsi* Gozhik, 2002 by original designation.

***Jelskia*** Bourguignat, 1877

Original source: Bourguignat 1877: 92.

Original classification: Genus of Valvatidae.

Type species: *Valvata jelskii* Crosse, 1863 by original designation.

Remarks: Not valid, because preoccupied by *Jelskia* Taczanowski, 1871: 97 resp. 128 (Chelicerata: Araneae), replaced by *Borysthenia* Lindholm, 1914.

***Liratina*** Lindholm, 1906: 190

Original source: Lindholm 1906: 190, 191.

Original classification: Subgenus of *Valvata* Müller, 1773.

Type species: *Valvata baicalensis* Gerstfeld, 1859 by original designation.

+ ***Loriolina*** Huckriede, 1967

Original source: Huckriede 1967: 170.

Original classification: Genus of Valvatidae.

Type species: + *Valvata loryana* Loriol, 1865 by monotypy.

***Lyogyrus*** Gill, 1863

Original Source: Gill 1863: 34 (footnote).

Original classification: Genus of Valvatidae.

Type species: *Valvata pupoidea* Gould, 1841 by original designation.

Remarks: According to Walker (1918: 33), Berry (1943: 57) or Kabat and Hershler (1993) to be classified in the **Amnicolidae** or **Hydrobiidae**.

“***Magovalvata***“ mentioned in Dybowski 1911: 965.

Misspelling of *Megalovalvata* Lindholm, 1906.

***Megalovalvata*** Lindholm, 1906

Original source: Lindholm 1906: 190.

Original classification: Subgenus of *Valvata* Müller, 1773.

Type species: *Valvata grubei* Dybowski, 1875 by original designation.

+ ***Mesovalvata*** Wei, 1984 (in Xinjiang Dizhi Ju 1984)

Original source: Xinjiang Dizhi Ju (1984: 83) *fide* Zoological Record (126(9): #3985).

Original classification: Subgenus of *Valvata*.

Type species: + *Valvata (Mesovalvata) karameilica* Wei, 1984 by original designation.

+ ***Michaudia*** Locard, 1883 (non Pallary 1926: 15, **Helicidae**)

Original source: Locard 1883a: 82.

Original classification: Subgenus of *Valvata*.

Type species: *Valvata falsani* Locard, 1883 by monotypy (see there).

***Microcincinna*** Starobogatov, 1983

Original source: Sitnikova 1983: 34.

Original classification: Subgenus of *Valvata*.

Type species: *Valvata geyeri* Menzel, 1904 by original designation.

+ ***Microcyclas*** Raspail, 1909

Original source: Raspail 1909: 197.

Type species: *Microcyclas lamellosus* Raspail, 1909 by original designation.

Remarks: Cossmann (1921: 174) and Pacaud and Le Renard (1995: 170) considered this genus to be classified in the Valvatidae.

***Minutiana*** Fagot, 1892

Original source: Fagot 1892: 32.

Type species: *Valvata turgidula* Bourguignat, 1889 by monotypy.

Remarks: Kadolski (2008: 116) considered this genus as synonym to *Globuliana* Paladhile, 1866 (**Hydrobiidae**).

***Ochridotropidina* / *Ohridotropidina*** (nomen nudum)

Original source: Hadžišce 1955: 176, legend of textfigure.

Remarks: As outlined by Hadžišce (1956: footnote at page 58), the name in the legend, i.e. “*Valvata (Ochridotropidina) polinskii*”, was based on an error and according to the author should be *Valvata (Ohridotropidina) relicta* Poliński, 1929. However, also the name *Ohridotropidina* never was diagnosed and thus is not an available name.

+ ***Oncostoma*** Brusina, 1894 (not Sclater 1862: 208 [Aves: **Tyrranidae**])

Original source: Brusina 1894: 185.

Original classification: Genus of Valvatidae.

Type species: + *Valvata marginata* Michaud, 1855 by monotypy by Sandberger (1875).

Remarks: Replacement name for preoccupied *Pachystoma* Sandberger, 1875. Likewise invalid, because a junior objective synonyme of *Stiphrostoma* Oppenheim, 1892 and *Oncostoma* Sclater, 1862.

+ ***Pachystoma*** Sandberger, 1875

Original source: Sandberger 1875: 711.

Original classification: Genus of Valvatidae.

Type species: + *Valvata marginata* Michaud, 1855 by monotypy.

Remarks: *Pachystoma* Sandberger, 1875 is preoccupied by *Pachystoma* Guilding, 1828 [**Ampullariidae**]. Oppenheim (1892: 776) proposed *Stiphrostoma* as a replacement name, whereas Brusina (1894: 185) proposed *Oncostoma* as a replacement name, the latter is again preoccupied, however.

***Pamirocincinna*** Sitnikova & Starobogatov, 1983 (in Sitnikova 1983)

Original source: Sitnikova 1983: 34.

Original classification: Subgenus of *Cincinna*.

Type species: *Valvata pamirensis* Starobogatov, 1972 by original designation.

***Piscinaliana*** Paladilhe, 1866 (non Bourguignat 1881: 333 [**Bivalvia** – **Unionidae**])

Original source: Paladilhe 1866–1869: 26 (resp. Paladilhe 1866: 169).

Original classification: Genus of Valvatidae.

Type species: *Nerita piscinalis* Müller, 1774 by original designation.

Remarks: An objective junior synonym of *Cincinna* with the same type species.

***Planella*** Schlüter, 1838

Original source: Schlüter 1838: 13.

Original classification: Genus of Valvatidae.

Type species: *Valvata cristata* Müller, 1774 by monotypy.

Remarks: An objective junior synonym of *Valvata* with the same type species (ICZN Art. 61.3.3).

***Planorbiana*** Paladilhe, 1866

Original source: Paladilhe 1866–1869: 26 (resp. Paladilhe 1866: 169).

Original classification: Genus of Valvatidae.

Type species: *Valvata cristata* Müller, 1774 by original designation.

Remarks: An objective junior synonym of *Valvata* with the same type species (ICZN Art. 61.3.3).

***Planorbitina*** De Betta, 1870

Original source: De Betta 1870a: 127.

Original classification: Subgenus of *Valvata*.

Type species: not clearly designated. De Betta's (1870) paper contained *Valvata cristata* (with synonym *Valvata planorbis*) and *Valvata spirorbis*.

Remarks: An objective junior synonym of *Valvata* with the same type species (ICZN Art. 61.3.3).

***Pleurovalvata*** Haas, 1939

Original source: Haas 1939: 101.

Original classification: Subgenus of *Valvata*.

Type species: *Valvata sincera* Say, 1824 by original designation.

+ ***Polytropis*** Sandberger, 1875 (non Koninck, 1881: 107)

Original source: Sandberger 1875: 697.

Original classification: Subgenus of *Valvata*.

Type species: + *Valvata balatonica* Rolle, 1861 by subsequent designation by Cossmann (1921: 172). Based on SEM of the protoconch Bandel (2010: 105, pl. 9: figs 111–114) designated the species as type species of *Jekeliella* Bandel, 2010 (**Hydrobiidae**).

“***Protovalvata*”** mentioned in Pană, 2000

Original source: Pană 2000: 88.

Original classification: Genus of Valvatidae.

Type species not explicitly designated: “Genotype: LPB IIIg no.; Pl. V, figs 26–27“. This term is listed as the holotype of *Protovalvata naticiformis* n.sp. described at page 89 in the same paper as first species after the genus diagnosis.

Remarks: A specimen cannot serve as type of a genus–group name, only a nominal species can (ICZN Art. 61.1.2, 67.1.). Accordingly, the genus name is not available.

+ ***Provalvata*** Bandel, 1991

Original source: Bandel 1991: 21.

Original classification: Genus of Provalvatidae.

Type species: + *Valvata helicoides* Loriol & Jaccard, 1865 by original designation.

***Pseudomegalovalvata*** Kozhov, 1936

Original source: Kozhov 1936: 29.

Original classification: Sectio (“sect.n.”) of subgenus *Megalovalvata*.

Type species: *Valvata (Megalovalvata) bathybia* W. Dybowski, 1886 by monotypy.

Remarks: Although introduced with an infra–subgeneric rank, the name is available at subgeneric rank (ICZN Art 10.4; Welter-Schultes 2012b: 14).

**“*Pseudovalvata*“** mentioned in Bekman and Starobogatov (1975: 95).

Misspelling of *Pseudomegalovalvata* at the description of *Valvata (Pseudovalvata) profundicola* Bekman & Starobogatov, 1975. The name has never been diagnosed.

***Sibirovalvata*** Starobogatov & Streletzkaja, 1967

Original source: Starobogatov and Streletzkaja 1967: 223.

Original classification: Subgenus of *Cincinna*

Type species: *Valvata confusa* Westerlund, 1897 by original designation.

+ ***Sinorificium*** Guo, 1982 (in Guo, Yü and Pan 1982)

Original source: Guo, Yü and Pan 1982: 33.

Original classification: Genus of Valvatidae.

Type species: *Sinorificium yumenensis* Guo, 1982 by original designation.

Remarks: According to Sepkoski (2002: 99) a member of Caenogastropoda (**Neotaenioglossa**).

+ ***Stiphrostoma*** Oppenheim, 1892

Original source: Oppenheim 1892: 776.

Original classification: Genus of Valvatidae.

Type species: + *Valvata marginata* Michaud, 1855 by monotypy by Sandberger (1875).

Remarks: Expressly established as a new replacement name for *Pachystoma* Sandberger, 1875, by Oppenheim treated as a junior homonym of *Pachytoma* Swainson, 1840 (**Helicinidae**), the latter was incorrectly spelled *Pachystoma* by Fischer (1885, Manuel de Conchyliologie). Accordingly, *Stiphrostoma* was correctly established as a new replacement name, the act was just incorrectly justified. But this has no influence on the status of *Stiphrostoma* as a new replacement name.

***Tropidina*** Adams & Adams, 1854 (non Mörch, 1864)

(often cited as Tropidina H. & A. Adams, 1854)

(not Tropidina Martins, 1968 (p. 331), a subtribe of cerambycid Coleoptera).

Original source: Adams and Adams 1854, Vol. 1: 344.

Original classification: Subgenus of *Valvata*.

Type species: *Cyclostoma tricarinata* Say, 1817 by subsequent designation by Lindholm (1906: 191).

Remarks: *Tropidina* in Mörch (1864: 320) is explicitly attributed by Mörch to Adams & Adams and is therefore not a separately available name.

+ ***Turrivalvata*** Papp, 1953

Original source: Papp 1953: 111.

Original classification: Subgenus of *Valvata*

Type species: + *Valvata politioanei* Jekelius, 1944 by original designation.

Remarks: Not listed in Nomenclator Zoologicus (Neave 1939–1940/2005).

***Valvata*** Müller, 1773

Original source: Müller 1773: unpaginated first part [“n29” of archive.org] http://archive.org/stream/vermivmterrestri01mle#page/n29/mode/1up

Original classification: Genus of Valvatidae

Type species: *Valvata cristata* Müller, 1774 by subsequent monotypy by Müller (1774: 198).

Remarks: Placed on the Official List by Opinion 335, 1955, Opinions and Declarations rendered by the ICZN, 10(2): 50, 51. Senior objective synonym of *Valvearius* De Duméril, 1805, of *Planella* Schlüter, 1838, of *Planorbiana* Paladilhe, 1866, and of *Planorbitina* De Betta, 1870.

***Valvatinella*** De Betta, 1870

Original source: De Betta 1870: 127.

Original classification: Subgenus of *Valvata*.

Type species: *Nerita piscinalis* Müller, 1774.

Remarks: An objective junior synonym of *Piscinalia* Paladilhe, 1866 and *Cincinna* Mörch, 1864 with the same type species.

***Valvearius*** Duméril, 1805

Original source: Duméril 1805: 164–165.

Original classification: Genus of Valvatidae (erroneously cited as “*Valvearius* Müller”).

Type species: *Valvata cristata* Müller, 1774 by subsequent monotypy by Froriep (1806: 165).

Remarks: 1805 is the correct year of publication as shown by Gregory (2010) and Bour (2010). A German translation of Duméril’s work was authored by Froiep (1806: “Duméril’s analytische Zoologie”), but the original description by Duméril (without involvement of Froriep) was in 1805.

An objective junior synonym of *Valvata* Müller, 1773 with the same type species.

“***Valvulata***“ mentioned in Bowler-Kelly (1928: 454), also Hémery (1956: 426)

Misspelling of *Valvata* Müller, 1773.

**“*Volvata*“** mentioned in Berge (1847: pp. 17, 20, 26)

A repeated and constant misspelling of *Valvata* Müller, 1773.

## Part II: (Infra-) Specific names described in Valvatidae
General remarks

All taxa (species, subspecies, named varieties) are listed alphabetically here in their original version (only spelling corrected according to ICZN rules) regardless of applicability, current taxonomic status and synonyms. Strong et al. (2008) considered 71 valid extant species worldwide, the Worldwide Mollusc Species Data Base (WMSDB: www.bagniliggia.it/WMSD/) by Bagni Liggia currently (last update: 28^th^ April 2013) lists 55 extant taxa (species, subspecies, and named varieties), whereas the present search has revealed more than 210 extant valvatid taxon names. Accordingly the synonymy rate is close to 1:3, i.e. an average of 3 names for each species. In addition, about 40 nominal *Valvata* species are currently assigned to other familes and more than 100 other taxon names exist, which are solely based on various types of error.

I also add available data sources (types, on radula, SEM of protoconch, anatomy, etc.), which may be useful for future species delineation.

As a historical curiosity, certain shells of trichopteran insects (genus *Hydropsyche*) were considered to be *Valvata* species by early authors (e.g. *Valvata agglutinans*, *Valvata arenifera*, *Valvata crispata*).

Most of the erroneously classified nominal valvatids belong to the Hydrobiidae with often very similar, so–called “valvatiform” teleoconchs. Protoconch morphology is quite different between the two families, however, and the valvatid protoconch with longitudinal riffles is very typical (Binder 1967b; Hadžišce et al. 1976; Falniowsky 1989, etc....). This makes also the classification of many fossil nominal *Valvata* species doubtful, and SEM-studies of the protoconch are necessary to clear up doubtful cases (e.g. Harzhauser and Kowalke 2002; Bandel 2010).

Several species of nominal *Valvata* species have been transferred to the family Planorbidae (Panpulmonata, Hygrophila). The single nominal extant *Valvata* from the southern hemisphere, *Valvata pedderi*, has been now placed in the euthyneuran, pan–pulmonate Glacidorbidae with Gondwana distribution, thus making at least the extant Valvatidae mostly a taxon nearly exclusively found only in the northern hemisphere.

Considering the many non–valvatids (mostly hydrobiids) among the formal descriptions of extant “Valvatidae”, it is more than likely that this is also the case among the fossil species, since the diagnostic fine–structure of the protoconch has only been rarly studied by SEM (e.g. by Harzhauser and Kowalke 2002; Harzhauser et al. 2002; Harzhauser and Binder 2004; Bandel 2010). The stratigraphy for European Tertiary sources follows Harzhauser and Mandic (2008).

Throughout the decades a high number of valvatid taxon names in the literature or in the internet have been created by misspellings, confusions or other errors. Previously, these errors were mostly due to simple errors of authors or publishers, whereas in more recent times the electronic versions and copies add to the problem: There is no doubt that electronic libraries such as the Biodiversity Heritage Library www.biodiversitylibrary.org/, Gallica http://gallica.bnf.fr/, the Digitizing Center in Göttingen http://gdz.sub.uni-goettingen.de/ or DigiLit in Linz www.landesmuseum.at/datenbanken/digilit/ are of substantial value for current taxonomic research. Indeed, the current study could not be conducted in a reasonable time–frame without the help of these resources. However, the automatic text recognition software used by some websites provides a new source of misspellings and errors, in particular, if old papers or books in Gothic typescript or in poor print condition are used. Many of these mistakes were found in the *Global Name Index* (GNI) or in the *Index of Organismic Names* (ION), some have also been copied by the *Global Biodiversity Information Facility* (GBIF) or by the World Mollusc Species Data Base (WMSDB). In the following catalog such names are listed and the most probable explanation is provided, obvious errors have not been listed herein. The most impressive example probably is “*Valvata ouscubakus* Nykk., 1895” for *Valvata piscinalis* (Müller, 1774) attributed at http://content.lib.washington.edu/ to fig. 66 for Cooke (1895). I fully agree with the warnings of http://www.wikipeetia.org/Valvata, yet there is little doubt that the following list is not complete and will grow in the future. I will be grateful for explanations of any names with currently unknown source.

+ *Valvata (Cincinna) abavia* Huckriede, 1967

Original source: Huckriede 1967: 165, pl. 23: figs 1–14, pl. 24: fig. 6.

Type horizon: Upper Jurassic, Lower Kimmeridge.

Type locality: Hannover-Linder, Lindener Berg, Germany.

*Valvata (Cincinna) sorensis* var. *abbreviata*: Lindholm, 1909

Original source: Lindholm 1909: 72–73, pl. 1: figs 66–67.

Type locality: Lake Baikal, Prorwinskji Ssor, Russia.

Remarks: In the original description Lindholm misspelled the name as *Valvata (Cincinna) ssorensis* var. *abbreviata*.The latter misspelling has been often repeated in particular by Russian authors.

+ *Valvata abdita* Brusina, 1902

Original source: Brusina 1902: pl. 13: figs 54–56.

Type horizon: Lower-Middle Miocene, Burdigalian-Langhian.

Type locality: Lower-Middle Miocene, Burdigalian-Langhian; Dugoselo, Croatia.

+ *Valvata (Aphanotylus) aberrans* Bukowski, 1895

Original source: Bukowski 1895: 24, 33–35, 63, pl. 9: figs 1–2.

Type horizon: Middle Pliocene.

Type locality: near Monastery Skhiádi, Rhodus Island, Greece.

Remarks: Lectotype figured by Willmann (1981: 75, fig. 23B).

*Valvata piscinalis acuminata* Jeffreys, 1862

Original source: Jeffreys 1862, Vol. 1: 73 (n383 of digitized volume at archive.org).

Type locality: Avon River, Bristol, England and North of Ireland.

+ *Valvata adeorboides* Fuchs, 1870

Original source: Fuchs 1870a: 347, pl. 17: figs 5–7.

Type horizon: Upper Miocene, Pannonian (Transdanubian).

Type locality: Rădmănești, near Lugos in Banat, Romania.

+ *Valvata aegaea* in Neumayr (1880: 284) (nomen nudum with locality)

Horizon: Pliocene−Pleistocene.

Locality: Kos Island, Greece.

*Valvata aegyptiaca* Innes, 1884

Original source: Innes 1884: 350

Type localities: “Canaux d’Alexandrie et lac Mariout; lac du jardin khédivial à Ghizeh près de Cairo; bords du lac Moeris, au Fayoun; rives du lac Timsah et marais près de Ramses”, all Egypt.

Remarks: Innes (1884: 350) refers to “Bourguignat, Spec. Moll. n^o^. 195, 1878”.

However, as outlined by Connolly (1934), the latter works was never published.

“*Valvata aequanica* Locard, 1883” and “*Valvata aequanica* Locard, 1889“ mentioned in Settepassi and Verdel (1965: 380)

Misspelling of + *Valvata sequanica* Locard, 1883.

*Valvata agglutinans* Tassinari, 1858 (non Lechmere Guppy, 1864).

Original source: Tassarini 1858: 2.

Type locality: Italy.

Remarks: This is the larval shell of a **trichopteran insect** (genus *Helicopsyche*) (see Bourguignat 1859: 545, Hagen 1864: 130, Bland 1865; Westerlund 1879).

*Valvata agglutinans* Lechmere Guppy, 1864 (not Tassarini, 1858)

Original source: Lechmere Guppy 1864: 245.

Type locality: Northern part of Island Trinidad.

Remarks: A junior homonym of *Valvata agglutinans* Tassarini, 1858. Also *Valvata agglutinans* Lechmere Guppy, 1864 is considered a synonym under the **trichopteran** *Helicopsyche maculisternum* Botosaneanu in Botosaneanu and Alkins-Koo 1993 (Johanson 2002: 59).

“*Valvata aleina* Westerlund, 1877” (GNI)

Misspelling of *Valvata aliena* Westerlund, 1877.

*Valvata aliena* Westerlund, 1877

Original source: Westerlund 1877: 63, pl. 1: fig. 15 (fide Westerlund 1886: 136, the original plate lacks a legend).

Type locality: Nizhnij Inbatsk, Yenisej region, central Siberia, Russia (“Jenissei, Nischnij Inbatsk (lat. 63° 50’)”).

Lectotype designated by Starobogatov and Streletzkaya (1967) and deposited at Zoological Institut of the Russian Academy of Sciences, St. Petersburg, No. 8 in systematic catalogue under the name. Paralectotypes in Natural Museum Göteborg, No. 4664 (2 specimens) and Swedish Museum of Natural History, No. 14:89 (2 specimens) (see Vinarski et al. 2013b).

*Valvata naticina* f. *alligans* Lindholm, 1927

Original source: Lindholm 1927: 22–23.

Type locality: Weichsel river near Plock, Poland.

Holotype figured by Sitnikova et al. (1986) and Anistratenko and Anistratenko (2001: 133, fig. 97), deposited at Zoological Institute of the Russian Academy of Sciences, St. Petersburg, Nr. 1 in systematic catalogue under the name.

+ *Valvata almerai* Almera, 1894

Original source: Almera 1894: 187, pl. 5: fig. 25 (attributed to Brusina, but ICZN Art. 50.1.1 is not satisfied). Expressively referred to *Valvata almerae* [sic] in Almera et al. (1892: 45) (nomen nudum with same locality).

Type horizon: Middle Pliocene.

Type locality: Papiol, province Barcelona, Spain.

*Valvata alpestris* Küster, 1853

Original source: Küster 1852–1853: 86, pl. 14: fig. 7, 8. (for publication years see Welter-Schultes 1999: 170).

Type locality: “in kleinen Seen an der Quelle der Giessbaches unweit des Faulhorns bei Grindelwald in der Schweiz” [in small lakes close to the fontain of Giessbach near the Faulhorn at Grindelwald in Switzerland] (Küster 1853: 87), Switzerland.

Types are figured by Boeters and Falkner (2002: pl. 15: figs 8–10).

Remarks: Often seen as subspecies of *Valvata piscinalis* (Müller, 1774), but Glöer and Zettler (2005: p. 15) consider this a species proper: “*Valvata alpestris* unterscheidet sich deutlich von *Valvata piscinalis*, so dass wir von einer eigenständigen Art ausgehen, auch wenn der Artstatus noch nicht eindeutig geklärt ist.” [*Valvata alpestris* differs significantly from *Valvata piscinalis* so that we accept it is a species proper, although the species status is not yet cleared up unequivocally].

Radula and anatomy were described by Starmühlner (1952).

+ *Valvata alta* Deshayes, 1862

Original source: Deshayes 1861–1863, Vol. 2: 524, pl. 36: figs 3–5.

Type horizon: Tertiary lignites.

Type locality: Sainceny, Bassin de Paris, France.

+ *Valvata (Cincinna) altaica* Popova & Starobogatov, 1981 (in Popova 1981)

Original source: Popova 1981: 18, pl. 5: figs 1–2.

Type horizon: Neogene.

Type locality: Chuisk Basin (Irkutsk Region) and Altai Mountains, Russia.

Remarks: “*Valvata* (*Cincinna*) cf. *altaica* Popova & Starobogatof, 1981” is figured by Zhu X–G (1985: 673, pl. 1: figs 1–6).

+ *Liratina altispiralis* Yü, 1982

Original source: Yü 1982: 262, pl. 3: figs 5–7 (not seen; Zoological Record 1982(A2): #4208).

Type horizon: Cretaceous.

Type locality: Xizang, China.

+ *Pentagoniostoma altispiratum* Branson, 1935

Original source: Branson 1935: 520–521, pl. 57: figs 1–3.

Type horizon: Jurassic, Morrison Formation.

Type locality: 3 miles south of Mayoworth, Wyoming, USA.

*Valvata ambigua* Westerlund, 1873 (under heading of subgenus *Tropidina*)

Original source: Westerlund 1873: 439.

Type locality: “Sverige vid Göteborg” [Sweden near Göteborg] (see Vinarski et al. 2013a).

Types: Naturalhistoriska Museet Göteborg, #7242 (for identification see Vinarski et al. 2013a, b).

Remarks: European authors mostly considered this taxon as a synonym to *Valvata piscinalis* (Müller, 1774). However, recently Glöer and Diercking (2010: 79) and Vinarski et al. (2013a) strongly argued for genuine species status because of the lower shell, wider umbilicus and differing ecology, whereas Welter-Schultes (2012a: 44) still considered synonymy with *Valvata piscinalis*.

“*Valvata ambiqua*” (GNI)

Misspelling of *Valvata ambigua* Westerlund, 1873.

*Cincinna (Sibirovalvata) amurensis* Moskvicheva, 1985 (in Starobogatov and Zatravkin 1985)

Original source: Starobogatov and Zatravkin 1985: 1156: fig. 1 (shell).

Type locality: Kutumanda River (Amur basin), Russia.

Holotype: According to Kantor et al. (2011: 65) in Zoological Institut of the Russian Academy of Sciences, St. Petersburg. Nr. 1 in systematic catalogue under the name.

*Valvata macrostoma* var. *anapensis* Westerlund, 1883

Original source: Westerlund 1883: 169 (cited as “*Valvata macrostoma* var. *anapensis* Cafici), but ICZN Art. 50.1.1 is not satisfied.

Type locality: river Anapo, Sicily, Italy.

+ *Valvata anconae* De Stefani, 1877

Original source: De Stefani 1877: 305, pl. 18 (2): fig. 5.

Type horizon: Pliocene.

Type locality: Arno Valley, near Montecarlo, Italy.

*Valvata andreaei* Menzel, 1904

Original source: Menzel 1904b: 96.

Type locality: Diluvial (interglacial) freshwater sediments of Wallensen south of Hannover and in older alluvial calcareous tuff of Alfeld and er Leine, Niedersachsen, Germany.

Types not traced.

Remarks: Replacement name for *Valvata (Cincinna) andreana* Menzel, 1904 by the author himself. *Valvata andreaei* is also (solely) used by Menzel (1904c: 286, pl. 14: figs 1–10, 15–24, 26, 29–31, 35–36, 39), recently also by Vinarski et al. (2008: 177).

+ *Valvata (Cincinna) andreana* Menzel, 1904

Original source: Menzel 1904a: 77 with textfigure.

Type horizon: Diluvial (interglacial) freshwater sediments.

Type locality: Wallensen south of Hannover and in older alluvial calcareous tuff of Alfeld and er Leine, Niedersachsen, Germany.

Remarks: Replaced by Menzel (1904b: 96) himself to *Valvata andreaei* Menzel, 1904 in the same issue of the journal. *Valvata andreaei* is also (solely) used by Menzel (1904c: 286, pl. 14: figs 1–10, 15–24, 26, 29–31, 35–36, 39), recently also by Vinarski et al. (2008: 177).

“*Valvata andrezowski*” (GNI, GBIF)

According to Dr. Gary Rosenberg from the Academy of Natural Sciences of Drexel University (Philadelphia), the digitized label of a single sample says “*Valvata andrezowski* Jelsch”; the back of the label says “Kiew”. There is also a penciled note “These 2 spp. mixed by breakage of bottles”, the second species being *Valvata spirorbis*, for which there is also a label. There are three specimens in the lot; one of them is too large to be *Valvata spirorbis* (= *cristata*). The larger specimen is similar to *Valvata naticina*. The lot came from the C. M. Wheatley Collection deposited by the University of Pennsylvania. “Jelsch” means “Jelski”, who published *Valvata menkeana* [see there]. Perhaps Jelski distributed specimens under the manuscript name “*andrezowski*” and then decided to give it a different name.

+ *Amplovalvata suturalis anjipingensis* Yü, 1980 (in Yü and Pan 1980)

Original source: Yü and Pan 1980: 149, figs ?? (not seen, Zool. Rec. 117 / 1980(A2): #3883).

Type horizon: Mesozoic.

Type locality: Zhejiang, Southern Anhui, China.

*Valvata piscinalis annandalei* Preston, 1916

Original source: Preston 1916: 162, pl. 9: figs 5, 5a, 5b.

Type locality: Lake Biwa, Japan.

*Valvata annelata* Menke, 1845

Original source: Menke 1845: 127.

Type locality: Swamp (paludosis) at the basis of Mount Sinai, Egypt.

+ *Valvata anomala* Moore, 1867

Original source: Moore 1867, 23: 556, pl. 15: figs 7–9.

Type horizon: Lower Jurassic.

Type locality: Charterhouse Mine, South Wales, U.K.

“*Valvata antigua*” (GNI)

Misspelling of *Valvata antiqua* Morris, 1838.

*Valvata antiqua* Morris, 1838

Original source: Morris 1838: 544 (with reference to the figure by Sowerby (1838: 547, figure 26).

Type locality: Grays in the Thames valley [from the publication titles], England.

Remarks: Mostly considered as an ecomorph of *Valvata piscinalis* (Müller, 1774), but Russian authors (e.g. Anistratenko and Anistratenko 2001: 141; Starobogatov et al. 2004) believe that *Cincinna antiqua* sensu Anistratenko, 1998 is probably a separate species.

+ *Amplovalvata antiqua* Pan, 1980 (in Yü and Pan 1980)

Original source: Yü and Pan 1980: 148, figs 21–24 (not seen; Zool. Rec. 1980(A2): #3883).

Holotype: http://159.226.74.248:8000/viewSpeciDetailsNormal.jsp?bbbh=36286

Type horizon: Middle Jurassic.

Type locality: Zhejiang, Southern Anhui, China.

Remarks: Cited as + *Amplovalvata antiqua* “sp.nov.” Pan in Guo et al. (1982: 32–33, pl. 12: figs 30–32). However, according to Zhu (1994: 94) both namings refer to the same specimens.

+ *Costovalvata antiqua* Zhu, 1994

Original source: Zhu 1994: 96–97 (Chinese) /103–104 (English), pl. 4: figs 1–7.

Type horizon: Middle Jurassic, Toutunhe Formation.

Type locality: Ziniquanzi, Janggar Basin, Northern Xinjiang, China.

*Valvata antiquilina* Mozley, 1934

Original source: Mozley 1934: 1–2, pl. 1: fig. 4.

Type locality: Lake Khomotenoye, Aj-Bulat drainage basin, Siberia, approximately 370 km southeast of Omsk (= Khomutinoe Lake, Burla Lake system, Altay).

Holotype: U.S. National Museum #469212.

Remarks: The holotype was designated in the original publication as “type”. The lot in the type collection of USNM with the catalogue number 469212 contains a single specimen, which matches the original illustration and measurements. The specimen is labeled as syntype on the later printed label.

“*Valvata anvandalei*” (GNI)

Misspelling of *Valvata piscinalis annandalei* Preston, 1916.

+ *Valvata aphanotylopsis* Brusina, 1902

Original source: Brusina 1902: pl. 14: figs 18–21.

Type horizon: Lower Miocene, Pontium.

Type locality: Begalijica, Serbia.

+ *Valvata (Cincinna) applanata* Youluo, 1978 (name of author according to the type catalogues of Nanjing Institute)

Original source: Youluo 1978: 30, pl. 3: fig. 22–28.

Type horizon: Lower Tertiary.

Type locality: coastal region of Bohai, China.

+ *Planorbis arcelini* Bourguignat, 1870 (in Ferry 1870)

Original source: Appendice by Adrien Arcelin in Ferry 1870: 109. Name and entire description were attributed by Arcelin to Bourguignat in the original source, this meets the conditions of ICZN Art. 50.1.1 for Bourguignat’s authorship.

Type horizon: Pliocene (“Marnes bleues”).

Type locality: Berges de la Saóne, France.

Remarks: cited as *Valvata arcelini* in Locard (1881: 181) and in Bourguignat (1891: 30).

“*Valvata arenaria*” (GNI, GBIF)

Misspelling of *Valvata arenifera* Lea, 1834.

*Valvata arenifera* Lea, 1834

Original source: Lea 1834: 104, pl. 15: figs 36a–b.

Type locality: Cumberland river near Nashville, Tennessee, U.S.A.

Remarks: the larval shell of an **trichopteran insect** (*Helicopsyche* resp. *Phryganea*) (see Bourguignat 1859, Hagen 1864: 129, Bland 1865, Westerlund 1879). Considered a synonym under *Helicopsyche borealis* (Hagen, 1861) by Johanson (2002: 32), but *arenifera* Lea, 1834 has priority.

“*Valvata ausonia*” (GNI, GBIF/SysTax).

Possibly an erroneous spelling of *Omalogyra ausonia* Palazzi, 1988 (similar shell as *Valvata cristata*), since GBIF/SysTax mentioned “Atlantic Ocean, Mediterranean Sea, Italy” as locality. *Omalogyra ausonia* has been transferred to *Palazzia* (Archaeogastropoda, incertae sedis) by Warén (1991: 75).

+ *Valvata (Cincinna) austrina* Pan, 1977

Original source: Pan 1977: 117, pl. 5: fig. 21.

Type horizon: ?? (whole text in Chinese).

Type locality: Yunnan, China.

***Cristatiana*** Servain, 1888

Original source: Servain 1888: 309.

Type species: *Valvata planorbulina* Paladilhe, 1867 by monotypy.

Remarks: Servain (1888) cited *Valvata cristata* Müller, 1774, but did not give a direct and unambiguous indication that *Valvata cristata* was to be included in *Cristatiana*.

+ *Valvata avilianensis* Pollonera, 1888

Original source: Pollonera 1888b: 50, pl. 1: figs 16–18.

Type horizon: Post-Pliocene.

Type locality: Lago di Aviliana, Italy.

*Valvata baicalensis* Gerstfeldt, 1859

Original source: Gerstfeldt 1859: 514–515 (in issue of journal, 10–11 in reprint): fig. 25a,b,c.

Type locality: Lake Baikal, Russia.

Lectotype esignated by Sitnikova et al. (1983) and deposited at at Zoological Institut of the Russian Academy of Sciences, St. Petersburg, Nr. 104.

Remarks: (1) Genital details depicted by Sitnikova (1983: fig. 1/6).

(2) Ostrovskaya et al. (1996, 2004) showed that the species is at least partly polyploid.

(3) Live photos (as “*Megalovalvata baicalensis* Gerstf.”) at: http://www.geol.irk.ru/baikal/rep_2008/pdf/baikal2008_apx5.pdf (Fig. 13) and: www.underwaterphotography.com/Photo-Contest/underwater-photo.aspx?ID=74736

(4) Spawn data at: http://userpage.fu-berlin.de/~rpeter/deutsch/repro/ei_valva.html

“*Megalovalvata baikalensis* Gerstfeldt, 1859“ (WMSDB)

http://www.bagniliggia.it/WMSD/HtmSpecies/2280450269.htm

Misspelling of *Valvata baicalensis* Gerstfeldt, 1859.

*Valvata tricarinata bakeri* Fluck, 1932

Original source: Fluck 1932: 20.

Type locality: Oneida Lake, New York, USA.

Holotype: Academy of Natural Sciences of Drexel University, Philadelphia (ANSP) #169016.

Remarks: Probably one of the many lake–specific forms of *Valvata tricarinata*.

+ *Valvata balatonica* Rolle, 1861 (?non Servain, 1881)

Original source: Rolle 1861: 209, pl. 1: fig. 5

Type horizon: Upper Miocene, Pannonian (Transdanubian).

Type locality: near Tihany, Lake Balaton, Hungary.

Remarks: (1) It is unclear, whether *Valvata balatonica* in Servain (1881: 94–95) refers to the same species: Type localities of both authors are very close, but Servain (1881) did not mention Rolle’s description and provided a full diagnosis.

(2) Based on SEM of the protoconch Bandel (2010: 105, pl. 9: figs 111–114) designated *Valvalta balatonica* Rolle, 1861 as type species of *Jekeliella* (**Hydrobiidae**).

*Valvata balatonica* Servain, 1881 (?non Rolle, 1861)

Original source: Servain 1881: 94.

Type horizon: Collected from detritus, thus possibly (sub)fossil.

Type Locality: “entre les bains de Füred et la presqu’île de Tihany”, Lake Balaton, Hungary.

Remarks: It is unclear, whether *Valvata balatonica* Rolle, 1861 refers to the same species: Type localities of both authors are very close, but Servain (1881) did not mention Rolle’s description and provided a full diagnosis.

+ *Pseudoamnicola balizacensis* Degrange-Touzin, 1892

Original source: Degrange-Touzin 1892: 189, pl. 5: fig. 8, 8a-d. Figured as *Valvata balizacensis* by Cossmann (1921: pl. 4), described and figured as *Valvata (Cincinna) balizacensis* (Degrange-Touzin) by Wenz (1936: 235-236, textfig. 4).

Type horizon: Miocene.

Type locality: Calcarie blanc de l’Agenais, à Balizac, France.

+ *Valvata balteata* Brusina, 1878

Original source: Brusina 1878: 352. Figured by Stefănescu (1896: 125, pl. 10: figs 137–138) and Brusina (1897: 25, pl. 13: figs 28–31).

Type horizon: Pliocene-Pleistocene, Romanian.

Type locality: Čaplja, Gromačnik, Slavonia, Croatia.

+ *Valvata banatica* Brusina, 1902

Original source: Brusina 1902: pl. 13: figs 50–53.

Type horizon: Upper Miocene, Pannonian (Transdanubian).

Type locality: Rădmănești, Romania.

+ *Liratina basicarinata* Youluo, 1978 (name of author according to the type catalogues of Nanjing Institute)

Original source: Youluo 1978: 32, pl. 4, figs 15–17.

Type horizon: Lower Tertiary.

Type locality: coastal region of Bohai, China.

*Valvata tricarinata* var. *basalis* Vanatta, 1915

Original source: Vanatta 1915: 105: figs 3–4.

Type locality: Hudson River, NY, USA.

*Valvata bathybia* Dybowski, 1886

Original source: W. Dybowski 1886: 119–120, pl. 4: figs 2a–c (shell), 4a–b (radula).

Type locality: Kultuk (southern Lake Baikal) (restricted in Sitnikova et al. 2004 by designation of a lectotype, stored at Zoological Institut of the Russian Academy of Sciences), Russia.

Lectotype designated by Sitnikova et al. (2004) and deposited at Zoological Institut of the Russian Academy of Sciences, St. Petersburg.

Remarks: SEM photos of shell, protoconch, and radula are depicted by Röpsdorf and Riedel (2004: fig. 7J–M).

+ *Tropidina bellireticulata* Youluo, 1978 (name of author according to the type catalogues of Nanjing Institute)

Original source: Youluo 1978: 33, pl. 4: figs 4–5, pl. 31, fig. 5.

Type horizon: Lower Tertiary.

Type locality: coastal region of Bohai, China.

“*Valvata beltrami* Contreras-Arquieta, 1993” (WMSDB).

Misspelling of *Valvata beltrani* Contreras-Arquieta, 1993 at page 194 at www.flmnh.ufl.edu/malacology/mexico-central_america_snail_checklist/part1.htm

*Valvata beltrani* Contreras-Arquieta, 1993

Original source: Contreras-Arquieta 1993: 1, figs 1A–F (shell), 2A–C (SEM of protoconch).

Type locality: Charco Azul, San Juan de Aviles, Aramberri, Nuevo León, México.

Holotype: U.S. National Museum #860587.

+ *Valvata (Cincinna) benoisti* Cossmann, 1899

Original source: Cossmann 1899b: 142, textfig. 5.

Type horizon: Jurassic – Bathonien.

Type locality: Département de l’Indre, France.

Remarks: Considered as *Cryptonerita benoisti* (Cossmann, 1899) (**Neritidae**) by Fischer (1964: 45, textfigs 17a–c, 18, 19; 65).

“*Valvata piscinaloides* var. *berthoni*“ mentioned in Fontannes (1883: 441) (nomen nudum with locality)

Locality: Middle Pliocene; Saint-Laurent–de-Arbres, Département Gard, France.

Remarks: Cited by Durand (1913: 348) and Wenz (1928b: 2446), both without diagnoses or figure.

“*Valvata (Cincinna) besançoni* de Laub. & Carez“ mentioned in Cossmann and Pissarro (1910–1913: legend of pl. 13, figs 84–86).

Misspelled for + *Valvata bezanconi* Laubrière & Carez, 1880.

+ *Valvata beysehirensis* Glöer & Girod, 2013

Original source: Glöer and Girod 2013: 26, figs 4–6.

Type horizon: Pleistocene.

Type locality: A hillock to the west of the national road D695, at the latitude of Çiftlikköy, just south of the turning for this village (37°43'58.38"N, 31°42'08.76"E).

“*Valvata (Cincinna) bezancani* Laubriere & Carez” in Yu (1965: 32).

Misspelling of + *Valvata bezanconi* Laubrière & Carez, 1880.

+ *Valvata bezanconi* Laubrière & Carez, 1880

Original source: Laubrière and Carez 1880: 405–406, pl. 15: figs 15–17.

Type horizon: Upper Pliocene.

Type locality: Brasles near Château-Thierry, Département de Aisne, France.

*Valvata bicarinata* Lea, 1841 (non Willmann, 1981)

Original source: Lea 1841: 83 (vernacular name: “two–ridge valvata”).

Type locality: Schuylkill River, PA, West side, below Permanent Bridge, USA.

Holotype: U.S. National Museum #121098.

+ *Valvata heidemariae bicarinata* Willmann, 1981 (non *Valvata bicarinata* Lea, 1841)

Original source: Willmann 1981: 158–159, textfig. 56D–F.

Type horizon: Lower Pleistocene, Middle Iraki-Formation.

Type locality: Vokasia-Tal, Kos, Greece.

Remarks: A junior homonym of *Valvata bicarinata* Lea, 1841.

+ *Valvata bicincta* Fuchs, 1870 (non Whiteaves, 1885)

Original source: Fuchs 1870b: 535, pl. 21: figs 7–9.

Type horizon: Upper Miocene, Pannonian (Transdanubian).

Type locality: near Tihany at Lake Balaton, Hungary.

Remarks: Bandel (2010: 103–104, pl. 7: figs 82–85) designated this species as type species of *Muellerpalia* Bandel, 2010 (**Hydrobiidae**).

+ *Valvata bicincta* Whiteaves, 1885 (non Fuchs, 1870)

Original source: Whiteaves 1885: 25, pl. 3: figs 3, 8, 8a,b.

Type horizon: Cretaceous.

Type locality: mouth of the Blind Man River, North–west Territory, Canada.

Remarks: A junior homonym of *Valvata bicincta* Fuchs, 1870.

+ *Valvata biformis* Sinzov, 1876

Original source: Sinzov 1876: 2.

Type horizon: Upper Miocene.

Type locality: Novorossian, Ukraine.

Remarks: Figured by Sinzov (1877: 68, pl. 5, figs 11–17) and Gozhik (2007: 76, pl. 67: figs 4–6 as *Cincinna biformis*).

+ *Valvata (Tropidina) bifrons* Neumayr, 1875

Original source: Herbich and Neumayr 1875: 426 (in issue of journal, page 26 in reprint), pl. 17: fig. 3.

Type horizon: Lower Pliocene.

Type locality: Dacian/Lower Romanian; Vârghiș (= Vargyas), Siebenbürgen (Transsilvania), Romania.

“*Valvata bivarinata*” at www.discoverlife.org

Misspelling of *Valvata bicarinata* I. Lea, 1841.

*Valvata (Cincinna) biwaensis* Preston, 1916

Original source: Preston 1916: 161–162, pl. 9: fig. 4, 4a.

Type locality: Lake Biwa, Japan.

*Valvata bocconi* Calcara, 1842

Original source: Calcara 1842: 32. Cited as “Calcara in litt.” and figured in Küster (1852–1853: 90–91. Pl. 14: figs 16–19).

Type locality: Pantano di Mondello, region of Termini, Sicilia, Italy.

+ *Valvata (Tropidina) bojanovski* Pavlović, 1932

Original source: Pavlović 1932: 245/250, pl. 1: figs 17–19.

Type horizon: Pliocene−Pleistocene, Metohija Series.

Type locality: Topličane (“blue marne Levantines near Topelić”), Metohija basin, Kosovo.

Remarks: Miloshevich (1984) regards this species a synonym of *Valvata kochi* Pavlović, 1932.

+ *Valvata bonelliana* Pollonera, 1888

Original source: Pollonera 1888b: 50, pl. 1: figs 13–15.

Type horizon: Post-Pliocene.

Type locality: Contorno di Torino, Italy.

*Valvata borealis* Milachevich, 1881

Original source: Milachevich 1881: 236 (page 22 in reprint).

Type locality: near Moscow, Russia.

Remarks: Kantor et al. (2011: 67) considered the name a synonym of *Cincinna depressa* (Pfeiffer, 1821) resp. *Valvata piscinalis* (Müller, 1774).

+ *Valvata bouei* Pavlović, 1903

Original source: Pavlović 1903: 186, pl. 6: figs 18–20. Also figured by Miloshevich (1984: pl. 1: figs 18–20).

Type horizon: Pliocene−Pleistocene, Metohija Series.

Type locality: Orahovac region, Metohija basin, Kosovo.

Remarks: + *Valvata bouei* Pavlović 1911“ is listed in GNI und ION, the species is indeed mentioned by Pavlović (1911: 600), but without description. Its introduction in 1903 obviously was overlooked.

+ *Valvata bourdoti* Cossmann, 1899

Original source: Cossmann 1899a: 349 (in issue of journal, 43 in reprint), textfîg. 4.

Type horizon: Upper Eocene.

Type locality: Bois-Gouët, Lower Loire, Bretagne, France.

*Valvata bourguignati* Letourneux, 1869

Original source: Letourneux 1869: 197 (issue of journal), resp. 37 (in reprint).

Type locality: “Fontaine, prés du Moulin-Gachet (commune de Pissotte)” , Département deVendée, France.

Remarks: (1) Germain (1931: 675) stated synonymy to *Valvata globulina* Paladilhe, 1866, now under *Neohoratia* Schütt, 1961 (**Hydrobiidae**). 

(2) Bodon et al. (2001: 175): *Islamia globulina* (**Hydrobiidae**).

(3) According to Vimpère (2003) synonymous to *Islamia moquiniana* (Dupuy 1851) (**Hydrobiidae**). (4) Falkner and Boeters (2003: 21) designated a lectotype and considered the species as *Islamia bourguignati* (Letourneux, 1869).

+ *Valvata bouryi* Cossmann, 1888

Original source: Cossmann 1888: 209, pl. 8: figs 10–12. Also figured by Cossmann and Pissarro (1910–13, pl. 13: figs 84–87).

Type horizon: Upper Eocene.

Type locality: Neauphlette, west of Paris, France.

*Valvata branchiata* Gruithuisen, 1821

Original source: Gruithuisen 1821: 437, pl. 38: figs 1–15.

Type locality: not provided in the original source, but presumably near München, Germany, where the author lived.

Remarks: Menke (1845: 125) considered the taxon as a synonym of *Valvata cristata* Müller, 1774.

*Valvata (Cincinna) brandti* Westerlund, 1897

Original source: Westerlund 1897b: 129.

Type localities: “See Goktscha, Umgebung von Lagodechi” [Lake Goktscha, surrounding of Lagodechi]; both Kaukasus, Georgia.

Remarks: Considered a synonym of *Caspicyclotus sierversi* (Pfeiffer, 1771) (**Cyclophoridae**) by Kantor et al. (2011).

*Valvata (Cincinna) aliena* var. *brevicula* Kozhov, 1936

Original source: Kozhov 1936: 18, pl. 1: fig. 30.

Type locality: Bogushanskaya Inlet, Lake Baikal, Russia.

Lectotype (Sitnikova et al. 2004): Zoological Institute, Academy of Sciences, St. Petersburg, Russia.

“*Valvata brisenoi* Contreras” (GNI, GBIF)

· at Discover Life: http://www.discoverlife.org/mp/20q?search=Valvata+brisenoi

· Cited as submitted paper “Contreras-Arquieta, A. *Valvata briseñoi* n. sp. from Aramberri, State of Sierra León, México. The Veliger (Sometido)“ in Contreras-Arquieta (1993: 4), but the paper has never been published.

+ *Valvata bronni* Ancona, 1867 (in Cocchi 1867: ICZN Art. 50.1.1 is fully satisfied)

Original source: Cocchi 1867: 27 (page 371 of the volume in the digitized version).

Type horizon: Plio-Pleistocene.

Type locality: central Italy.

Remarks: (1) Refers to *Valvata obtusa* sensu Bronn (1831: 583 [= page 75 of the respective book chapter as stated by Ancona 1867], no. 394).

(2) Type species of *Stephania* Esu & Girotti, 1975: 222 (?**Ampullariidae**)

“*Amnicola brownie*“ mentioned in Carpenter (1872: Central Falls Weekly Visitor end of March 1872 [not seen])

Remarks: it is questionable, whether or not this newspaper is published work in the sense of ICZN Art. 8.1.1. If not, than *Valvata brownii* Carpenter, 1889 would be the original source of the name.

*Valvata brownii* Carpenter, 1889

Original source: Carpenter 1889: 67 (original description of 1872 copied).

Type locality: Cunliff’s Pond, at Elmville, south of Providence, Rhode Island, USA.

Remarks: Walker (1918: 147) referred the species to *Lyogyrus* Gill, 1863. Currently again considered as *Amnicola brownii* Carpenter, 1872 (**Hydrobiidae**) (GNI).

+ *Cincinna (Cincinna) bugense* [sic: should be *bugensis*, since *Cincinna* is feminine] Gozhik, 2007

Original source: Gozhik 2007: 76, pl. 66: figs 6–9.

Type horizon: Upper Miocene, Maeotian.

Type locality: near the town Michailowka, district Volgograd, Russia.

+ *Valvata bukowskii* Brusina, 1897

Original source: Brusina 1897: 25, pl. 14: figs 1–3.

Type horizon: Pliocene–Pleistocene (Dacian–Romanian).

Type locality: Bečić, Slavonia, Croatia.

*Cincinna (Sibirovalvata) bureensis* Starobogatov & Zatravkin, 1985

Original source: Starobogatov and Zatravkin 1985: 1158: fig. 6.

Type locality: lake on the left bank of the River Bureja, near Chekunda (old) settlement, Khabarovsk Territory, Sibiria, Russia.

Holotype: Zoological Institut, Russian Academy of Sciences, St. Petersburg, Nr. 1 in systematic catalogue under the name.

+ *Valvata hellenica* var. *cabeolensis* Fontannes, 1880

Original source: Fontannes 1880: 181, pl. 1: fig. 19.

Type horizon: Lower Pliocene.

Type locality: basin du Crest, south of Valence, France.

*Valvata humeralis californica* Pilsbry, 1908

Original source: Pilsbry 1908: 82.

Type locality: Bear Lake, San Bernardino Mountains, California, USA.

Remarks: Hovingh (2004) assumed that all specimens of *Valvata humeralis* Say, 1829 with localities in Mexico should be classified as *Valvata californica*.

+ *Valvata calli* Hanniball, 1910

Original source: Hanniball 1910: 107.

Type horizon: Marl–deposit, Upper Lahontan Quaternary.

Type locality: Summer Lake, Oregon, USA.

*Valvata callista* Innes, 1884

Original source: Innes 1884: 350

Type localities: “Marais à l’est de canal Mahmoudieh; bords du lac Moeris, au Fayoun”, all Egypt.

Remarks: Innes (1884: 350) refers to “Bourguignat, Spec. Moll. n^o^. 196, 1878”. However, as outlined by Connolly (1934), the latter works was never published.

“*Valvata cancellata*“ (GNI, ION)

Possibly an erroneous spelling or confusion of *Spirorbis valvata* Berger, 1859 and *Spirorbis cancellata* Fabricius, 1780 (Annelida, Polychaeta).

+ *Valvata cangshanensis* Pan, 1982

Original source: Pan 1982: p. ??, figs 24–27 (not seen, not listed in Zoological Record, data from the online type catalogue of Nanjing Institute).

Type horizon: Jurassic – Penglaizhen.

Type locality: Zhongjiang County, Sichuan Basin, China.

Holotype: http://159.226.74.248:8000/viewSpeciDetailsNormal.jsp?bbbh=53436

+ *Valvata carasiensis* Jekelius, 1944

Original source: Jekelius 1944: 120–121.

Type horizon: Middle Miocene, Sarmat.

Type locality: Soceni (Banat), Romania.

*Valvata carinata* Sowerby, 1834 (non Fuchs, 1870)

Original source: Sowerby 1834: pl. 41: fig. 2.

Locus typicus: not provided.

Remarks: Concerning the publication year of Sowerby’s work Petit (2006) should be considered.

+ *Valvata carinata* Fuchs, 1870 (not Sowerby, 1834)

Original source: Fuchs 1870b: 535, pl. 21: figs 10–12.

Type horizon: Upper Miocene, Pannonian (Transdanubian).

Type locality: near Tihany at Lake Balaton, Hungary.

Remarks: According to Bandel (2010: 104, pl. 7: figs 86–89) this species should be classified as *Muellerpalia carinata* (Fuchs, 1870) (**Hydrobiidae**). A junior homonym of *Valvata carinata* Sowerby, 1834.

“*Valvata caterinae*“ mentioned in Siodiropoulou (2003: 40–41, fig. 15).

Locality: Pliocene-Pleistocene; Ptolemaida, West-Macedonia, Greece.

Remarks: This PhD-Thesis, in which 35 species are ostendibley described, does not meet the conditions of ICZN Art. 8.1.3 and 9.9. Accordingly, the name is not available.

+ *Valvata (Aphanotylus) chalinei* Schlickum & Puisségur, 1978

Original source: Schlickum and Puisségur 1978: 4, pl. 1: fig. 5.

Type horizon: Tertiary.

Type locality: Montagny les Beaume, Département Côte d’Or, France.

Remarks: See also Esu (1983).

+ *Valvata changzhouensis* Yü, 1977 (in Yü and Wang 1977)

Original source: Yü and Wang 1977: 15, pl. 1: figs 8–12, pl. 3: figs 15–18.

Type horizon: Upper Cretaceous.

Type locality: Jiangsu Province, China.

*Cincinna chereshnevi* Bogatov, Zatravkin & Starobogatov, 1992 (in Bogatov and Zatravkin 1992)

Original source: Bogatov and Zatravkin 1992 (“1990”): 35–36: fig. 8. a–b [a–v].

Type locality: southern Chukchi Peninsula, basin of Khatyrka River, Lake Egergytgyn, Russia.

Holotype: Zoological Institut, Russian Academy of Sciences, St. Petersburg, Nr. 1 in systematic catalogue under the name.

*Cincinna chersonica* Chernogorenko & Starobogatov, 1987

Original source: Chernogorenko and Starobogatov 1987: 150.

Type locality: mouth of Dnieper river into Black Sea, Ukraine.

Holotype figured by Anistratenko and Anistratenko (2001: 144, fig. 116), According to Kantor et al. (2011: 66) in Zoological Institute of Russian Academy of Sciences, St. Petersburg, Nr. 1 in systematic catalogue under the name.

Remarks: A much more detailed description of the species was provided by Chernogorenko (1991).

*Cincinna chishimana* Prozorova & Starobogatov, 1999

Original source: Prozorova and Starobogatov 1999: 54–55, figs 2B, 3.

Type locality: Iturup Island, Reidovoe Lake, 1–5 m, Russia.

Holotype: Zoological Institut, Russian Academy of Sciences, St. Petersburg, Nr. 1 in systematic catalogue under the name.

Remarks: According to Kantor et al. (2011: 66) a synonym to *Cincinna kizakikoensis* Fujita & Habe, 1991

“*Valvata chohnokyi* Schlosser 1906“ (GNI, ION)

Misspelled and confused with + *Bithynia* (?) *cholnokyi* Schlosser, 1906.

*Valvata choristogyra* Hagenmüller, 1884

Original source: Hagenmüller 1884: 215 (cited as “*Valvata choristogyra* (Servain)”).

Type locality: River Elbe at Hamburg, Germany.

Remarks: Hagenmüller (1884) himself suspected that this is an open coiled form of *Valvata cristata* Müller, 1774. Also cited by Westerlund (1885: 143: “*Valvata choristogyra* (Serv.) Loc. Bull. Soc. Mal. Fr. 1884”), but could not be verified in Locard (1894), but in the directly following paper by Hagenmüller.

+ *Valvata (Cincinna) costatus cinctus* Taner, 1973 [should be *costata cincta*, since *Valvata* and *Cincinna* both are feminine]

Original source: Taner 1973: 97, pl. 4 (photos): figs 1–3, pl. 3 (drawings): figs 2–2a.

Type horizon: Neogene.

Type locality: Altintepe near the town of Fatsa close to the Black Sea, Turkey.

+ *Valvata circinata* Sandberger, 1871

Original source: Sandberger 1870–1875: pl. 18: fig. 5a–c (published 1871), described by him later at page 324 (published 1873). The name is attributed by some authors (e.g. Wenz 1928b: 2427) to Merian (1849: 34, mentioned as *Paludina circinata*, a nomen nudum), but ICZN Art. 50.1.1 is not satisfied.

Type horizon: Oligocene.

Type locality: “Kalke von Kleinkems”, north of Basel, Germany.

+ *Valvata cobalcescui* Brusina, 1885

Original source: Brusina 1885: 162. Figured by Stefanescu (1896: 124, pl. 10: figs 129–132); and as *Cincinna (Atropidina) cobalcescui* Stefanescu by Gozhik (2007: 80, pl. 69: figs 8–11).

Type horizon: Pliocene–Pleistocene, (Dacian–Romanian; *Paludina*–layers).

Type locality: Barboși (= Barboschi), Galati, Romania.

Remarks: As outlined by Brusina (1885: 162) a replacement name for *Valvata sulekiana* sensu Cobălcescu (1883: 142, pl. 13, fig. 18), non *Valvata sulekiana* Brusina, 1874.

*Valvata colbeaui* Roffiaen, 1869

Original source: Roffiaen 1869: 81, pl. 1: fig. 1a–d.

Type locality: Brienzersee near Iseltwald, Switzerland.

“*Valvata colli*” (GNI)

Misspelling of + *Valvata calli* Hanniball, 1910.

+ *Valvata comes* Hudleston, 1896

Original source: Hudleston 1896: 489–490, pl. 43: fig. 27, pl. 44: figs 2a–b.

Type horizon: Middle-Jurassic, Inferior Oolite; *Paludina*–beds.

Type locality: at Langton Bridge, Lincolnshire, England, U.K.

*Valvata compressa* Locard, 1889

Original source: Locard 1889: 38–40.

Type locality: first locality mentioned is “Poligny dans le Jura”, France.

*Valvata (Cincinna) confusa* Westerlund, 1897 (not *Valvata tricarinata* var. *confusa* Walker, 1902)

Original source: Westerlund 1897b: 130.

Type locality: “Sibirien. Thal des Olenek” [Siberia, valley of Olenjok], Russia.

Lectotype designated by Starobogatov and Streletzkaya (1967): Zoological Institute of the Russian Academy of Science, St. Petersburg, No. 32 in systematic catalog, figured by Vinarski et al. (2013b: fig. 3A) (17 paralectotypes, No. 33). Paralectotypes (5 specimens) in Naturalhistoriska Museet Göteborg, Sweden, No. 4656. (cf. Vinarski et al. 2013b).

Remarks: Genital details are depicted by Sitnikova 1983: fig. 1–2.

*Valvata tricarinata* var. *confusa* Walker, 1902 (non *Valvata (Cincinna) confusa* Westerlund, 1897)

Original source: Walker 1902: 124, fig. 2.

Type locality: not provided.

Remarks: Replaced by *Valvata tricarinata* var. *perconfusa* Walker, 1917 (see there).

+ *Valvata connectens* Brusina, 1892

Original source: Brusina 1892: 166 (issue of journal, 54 of reprint) (footnote).

Type horizon: Upper Pliocene, Pannonian.

Type locality: Markuševec (Zagreb), Croatia.

Remarks: Replacement name for *Valvata gradata* Brusina (1874: 135), *non* Fuchs, 1870.

+ *Valvata conoidalis* Michaud, 1855

Original source: Michaud 1855: 49–50 (issue of journal, 17–18 in reprint), pl. 5: fig. 19.

Type horizon: Lower Pliocene, Zanclean.

Type locality: Hauterive (Drôme), France.

Remarks: Considered as *Craspedopoma conoidale* (Michaud, 1855) (**Maizaniidae**) by Locard (1887: 243) and Sandberger (1875: 726).

*Valvata (Cincinna) consors* Westerlund, 1886

Original source: Westerlund 1886: 136.

Type locality: Klagenfurt in Kärnten, Austria.

*Nerita contorta* Müller, 1774

Original source: Müller 1774: 187–188 (Nr. 374).

Type locality: Fridrichsdal, Denmark.

Types possibly in Zoological Museum Copenhagen (Kantor et al. 2011: 66).

Remarks: Glöer (2002) considered this taxon as a synonym of *Valvata piscinalis antiqua*. Anistratenko and Anistratenko (2001: 139) cited the opinion of Ya.I.Starobogatov, that *Valvata piscinalis* var. *fluviatilis* Colbeau, 1859 is a synonym of *Cincinna contorta*. Most European malacologists under the name *Valvata fluviatilis* identified separate species, for which Starobogatov Starobogatov in Anistratenko and Anistratenko (2001: 139) proposed a new name *Cincinna (Cincinna) falsifluviatilis* (see there).

Radula figured by Hensche (1866).

*Helix contortaplicata* Gmelin, 1791

Original source: Gmelin 1791: 3661 (#144) (cited as *Helix contorta–plicata*).

Type locality: “Galliae” (France).

Remarks: Gmelin provided a short description, a reference to Müller’s (1774: 187–188) description of *Nerita contorta* (see there), and to the work of (not named) Dézallier d’Argenville of 1742 (Pl. 8: fig. 5). Synonymy with *Nerita contorta* Müller, 1774 remains unclear.

*Valvata coronadoi* Bourguignat, 1870

Original source: Bourguignat 1870b: 169 (in issue of journal, 51 in reprint), pl. 17 (resp. 4 in reprint): figs 5–8.

Type locality: “en los alrededores de Madrid, o, al menos, en algunos manantiales o arroyos de la provincia de Castilla La Nueva” [in Madrid’s surroundings or, at least, in some springs or strans of the New Castille Province], Spain.

Types: Lectotype Museum de l’Histoire Naturelle de Geneve (MHNG, figured by Arconada and Ramos 2006: figs 76, 80, 83, 86) and paralectotypes MHNG (figured ibid. figs 77, 79, 81, 84, 87, 89).

Remarks: Now *Neohoriatia* (?) *coronadoi* (Hydrobiidae) see Arconada and Ramos (2006: 101) (**Hydrobiidae**).

+ *Aphanotylus cossmanni* Brusina, 1894

Original source: Brusina 1894: 185.

Type horizon: Upper Miocene, Upper Pannonian, horizon of *Lyrcæa* (*Melanopsis*) / *Congeria*–layers.

Type locality: Kúp, Komitat Veszprém, Western Hungary.

*Valvata piscinalis* var. *costulata* Drouët, 1867

Original source: Drouët 1867: 93.

Type locality: Ouche, France.

+ *Valvata (Cincinna) costatus* [sic] Taner, 1973 [should be *costata*, since *Valvata* and *Cincinna* both are feminine]

Original source: Taner 1973: 96, pl. 3 (photos): figs 3–6, pl. 3 (drawnings): figs 1–1a.

Type horizon: Pliocene.

Type locality: Tabakalari, Tahir, Eastern Turkey.

+ *Valvata craterella* Russell, 1938

Original source: Russell 1938: 505, textfigs 1–3.

Type horizon: Oligocene.

Type locality: Park County, Colorado, USA.

*Valvata cressidana* Locard, 1889

Original source: Locard 1889: 42 (attributed to Letourneux, but ICZN Art. 50.1.1 is not met).

Type locality: Marais de Cressida, Corfu, Greece.

“*Valvata crisata* (Mull.)” (GNI)

Misspelling of *Valvata cristata* Müller, 1774.

“*Valvata crispata*” mentioned in Westerlund (1879: 18)

Remarks: (1) Name attributed by Westerlund to Benoit with a bibliographical reference to the un–labelled plates in “Benoit 1857: Tav. 7 Fig. 32–33”, not used by Westerlund 1879 (cf. ICZN Art. 11.5.2) and probably not made available elsewhere. Also De Gregorio (1895: 185) did not provide a name for these two figures.

(2) This name refers to the shell of a **hydropsychid trichopteran** (Hexapoda: *Phryganea*) (cf. Westerlund 1879: 18).

*Valvata cristata* Müller, 1774

Original source: Müller 1774: 198–199.

Type locality: Fridrichsdal (NW of Copenhagen), Denmark.

Types possibly in Zoological Museum Copenhagen (Kantor et al. 2011: 73).

Remarks: (1) SEM photos of shell and radula are provided by Falniowski (1990), genital details are depicted by Sitnikova (1983: fig. 1/1), general anatomy is described by Rath (1986: 82ff), sperm ultrastructure has been described by Healy (1990), for life cycle see Myzyk (2002). (2) Live photos at: http://www.allesumdieschneck.de/html/valvata_cristata.html

*Valvata cristatella* “f. Biguet” (i.e. Faure-Biguet) mentioned in Férussac and Férussac (1807: 128/129, nr. 170) (nomen nudum)

Remarks: Cited by Menke (1845: 126) as a synonym for a variety of *Valvata cristata* Müller, 1774 denoted with the letter b, but without making it available.

+ *Valvata piscinalis* var. *crusitensis* Fontannes, 1886

Original source: Fontannes 1886: 344–345, pl. 26: figs 45–46.

Type horizon: Pliocene–Pleistocene, Romanian.

Type locality: Crușeț (Crusita, valley of Amaradii), Dolj, Romania.

Remarks: figured as *Valvata (Cincinna) crusitensis* Fontannes by Kapan Yesilyurt and Taner (2002: 102, pl. 1: fig. 4).

*Valvata cumingii* Reeve, 1859

Original source: Reeve 1859(1): 171, pl. 17, fig. 88.

Type locality: England.

Remarks: Present status unknown, cf. Petit (2007: 91).

“*Valvata inflata* var. *curta* Tournouër” mentioned in Delafond and Depéret (1893: 152, pl. 9: figs 52–54)

Locality: Middle Pliocene; Saint-Amour, Département Jura, Region Franche-Comté, France.

Remarks: Delafond and Depréret (1893: 152) referred the name to specimens of the collection of Tournouër and consider these specimens from Bresse, France identical to *Valvata interposita* de Stefani, 1880.

*Valvata cyclomphala* Westerlund, 1889

Original source: Westerlund 1889: 1316 (diagnosis in the footnote).

Type locality: “Norvegia in Finmarkia oriental ad Koskiniavi (Flumen Pasvig)”, Norway.

“*Valvata (Valvata) cyclistomoides* Raspail“ mentioned in Wenz (1928b: 2467).

Consistent misspelling of *Valvata cyclotusoides* Raspail, 1909.

+ *Amplovalvata cyclostoma* Yen, 1952

Original source: Yen 1952: 39, pl. 6: figs 1a–f.

Type horizon: Upper Jurassic, Morrison Formation.

Type locality: Felch’s Ranch, Garden Park, 9 miles north of Canon City, Fremont County, Colorado, USA.

+ *Planorbis cyclostomus* Brusina, 1902

Original source: Brusina 1902: pl. 3: figs 10–12.

Type horizon: Pliocene-Pleistocene, Dacian-Romanian.

Type locality: Gromačnik, Slavonia, Croatia.

Remarks: Considered as a *Valvata* by Wenz (1928b: 2466).

+ *Valvata cyclostrema* Brusina, 1892

Original source: Brusina 1892: 167–168 (in issue of journal, 55–56 in reprint).

Type horizon: Upper Miocene, Pannonian.

Type locality: Markuševec (Zagreb), Croatia.

+ *Valvata cyclotusoides* Raspail, 1909

Original source: Raspail 1909: 196, pl. 4: figs 21–23. Also cited and figured in Cossmann and Pissarro (1910: 10, pl. 63: figs 84–88 (3 figs)). Wenz (1928b: 2467) misspelled this as “*Valvata (Valvata) cyclistomoides* Raspail“.

Type horizon: Middle Eocene, Upper Bartonian.

Type locality: Le Vouast near Montjavoult, north–west of Paris, Département Oise, France.

+ *Valvata cyrenophila* Andreae, 1884

Original source: Andreae 1884: 272, pl. 12: figs 1a–c.

Type horizon: Upper Oligocene, Chattien.

Type locality: Kolbsheim, Département Bas-Rhin, France.

“*Valvata cythropomatia* Hemph.” (GNI)

Probably corrupted by erroneous text recognition software for *Valvata erythropomatia* Hauffen, 1856.

+ *Cincinna dakangensis* Yü, 1974

Original source: Yü 1974: 372–373, pl. 198: figs 1–3.

Type horizon: Lower Jurassic.

Type locality: Jiangyou, Sichuan Province, Southwest China.

+ *Aphanotylus dakangensis* Pan, 1982

Original source: Pan 1982: ??, figs 5–8 (Not seen, not in Zoological Record, data from the online type catalogue of Nanjing Institute).

Type horizon: Mesozoic.

Type locality: artesian springs, Sichuan Jiangyou Grafschaft Kang, Sichuan province, China.

Holotype: http://159.226.74.248:8000/viewSpeciDetailsNormal.jsp?bbbh=53439

+ *Valvata dalaziensis* Zhu, 1980

Original source: Zhu 1980: 38, figs ?? (not seen; listed in Zoological Record 1980(A2): #3886).

Type horizon: Lower Cretaceous.

Type locality: Northwest China.

*Valvata sincera danielsi* Walker, 1906

Original source: Walker 1906: 28, pl. 1: figs 9–10.

Type locality: Cannon Lake, Rice Co. Minnesota, USA.

*Valvata danubiana* Put & Polyszcuk [sic!], 1969

Original source: Put and Polyszcuk 1969: 652, fig. б (b) at page 651.

Type locality: Right bank of Ochakov–mouth in the Danube delta, Ukraine.

+ *Valvata debilis* Fuchs, 1870

Original source: Fuchs 1870b: 535, pl. 21: figs 1–3.

Type horizon: Upper Miocene, Pannonian (Transdanubian).

Type locality: near Tihany at Lake Balaton, Hungary.

Remarks: According to Bandel (2010: 105) this species should be classified in the **Planorbidae**.

+ *Valvata decollata* Hislop, 1859

Original source: Hislop 1859: 171, pl. 5: figs 16a–b.

Type horizon: Tertiary.

Type locality: Tákli, Aurangabad, Maharashtra, East India.

Remarks: The valvatid nature of this taxon was doubted by Hrubesch (1965: 98)

+ *Valvata deflexa* Sandberger, 1863

Original source: Sandberger 1858–1863: 86, pl. 6: figs 11, 11a, 11b.

Type horizon: Lower Miocene, Littorinellenkalk.

Type locality: Kästrich in Mainz, Germany.

Remarks: Sandberger himself (1875: 493) considered this taxon as identical to *Planorbis crassilabris* Sanderberger, 1875.

Remarks: Types of Sandberger (1863) have been re–investigated by Kuster-Wendenburg (1973).

+ *Amplovalvata deformis* Pan, 1980 (in Yü and Pan 1980)

Original source: Yü and Pan 1980: 149, figs ?? (not seen, listed in Zoological Record 1980(A2) / 117: #3883).

Type horizon: Mesozoic.

Type locality: Zhejiang Zhuji, China.

Holotype: http://159.226.74.248:8000/viewSpeciDetailsNormal.jsp?bbbh=36290

+ *Valvata deisteri* Struckmann, 1880

Original source: Struckmann 1880: 86, pl. 2: figs 23a–c.

Type horizon: Lower Cretaceous.

Type locality: Coal–mine Egsdorf near Hannover, Germany.

+ *Valvata (Cincinna) delaunayi* Cossmann, 1907

Original source: Cossmann 1907: 232–233, textfig. 2.

Type horizon: Jurassic, Dogger, Bathonien.

Type locality: Saint-Gaultier (Indre), France.

Remarks: Judging from the figure of the shell (non–circular aperture) nearly with certainty no valvatid.

“*Heterovalvata delessei*“ mentioned in Munier-Chalmas (1879: 33) (nomen nudum)

Locality: Stratum ?; Saint Cyr, Tripoli, Libanon.

*Valvata delevieleusae* Hagenmüller, 1884

Original source: Hagenmüller 1884: 213–214.

Type locality: not provided, North Africa.

Remarks: Boettger (1905: 120) considered the taxon to belong to the hydrobiid genus *Daudebardiella* Boettger, 1905 because of the oblique aperture.

*Valvata cristata* var. *delpretiana* Paulucci, 1878

Original source: Paulucci 1878: 20 (name only), 51 (description).

Type locality: near Viareggio, Toscana, Italy.

*Valvata (Liratina) baicalensis* var. *demersa* Lindholm, 1909

Original source: Lindholm 1909: 79.

Type locality: Lake Baikal, “beim Uluss [type error: Fluss] Byrkin” [at river Byrkin], Russia.

Lectotype designated by Sitnikova et al. (1983) and deposited at Zoological Institut of the Russian Academy of Sciences, St. Petersburg; Nr. 1 in systematic catalogue under the name.

Remarks: (1) SEM of radula figured by Röpstorf et al. (2003: fig. 6B).

(2) Live photo (under “*Megalovalvata demersa* Ldh.”): http://www.geol.irk.ru/baikal/rep_2008/pdf/baikal2008_apx5.pdf (Fig. 3).

(3) Spawn data: http://userpage.fu-berlin.de/~rpeter/deutsch/repro/ei_valva.html

+ *Valvata densestriata* Pilsbry, 1934

Original source: Pilsbry 1934: 16.

Type horizon: Pliocene.

Type locality: 23 miles southwest of Hanford, Kettleman Hills, California, USA.

*Valvata depressa* Pfeiffer, 1821

Original source: Pfeiffer 1821: 100, pl. 4: fig. 33.

Type locality: „in einem schlammigen Wassergraben, unweit Hanau, bey dem Dorfe Enkheim“. [in a muddy ditch not far from Hanau, at the village Enkheim – today a part of Bergen-Enkheim in Frankfurt/Main], Germany.

Types: not traced. Pfeiffer’s collection was dispersed after his death (Dance 1986).

+ *Valvata deshayesi* Denainvilliers, 1875

Original source: Denainvilliers 1875: 68, pl. 3: fig. 1.

Type horizon: Lower Miocene, Aquitanian.

Type locality: Segrais near Piethiviers, Départment de Loiret, France.

*Paludina dilatata* Eichwald, 1830

Original source: Eichwald 1830: 219.

*Valvata dilatata* in Eichwald (1853: 292, pl. 10: fig. 35).

Type locality: Quaternary (!) deposits near Grodno (Hrodna), Belarus.

Lectotype designated by Starobogatov et al. (2004), figured by Anistratenko and Anistratenko (2001: 142, fig. 114), deposited in Zoological Institute of Russian Academy of Science, St. Petersburg, Nr. 1 (Kantor et al. 2011: 67) under the name.

Remarks: Russian authors (e.g. Anistratenko and Anistratenko 2001; Kantor et al. 2011: 67) regularly place this taxon in *Cincinna*.

*Valvata discors* Westerlund, 1886

Original source: Westerlund 1886, Part VI: 133.

Type locality: “Sweden Lake Ringsjön”. According to Vinarski et al. (2013b) this is Lake Ringsjön in the province Skania (Sweden).

Syntypes (2 specimens, AN 4649) in the Naturalhistoriska Museet Göteborg, figured by Vinarski et al. (2013b: figs 3B–C) and in the Swedish Museum of Natural History (AN 14:78).

Remarks: Glöer (2002: 184, 193) regarded *Valvata discors* as a subspecies of *Valvata piscinalis* (Müller, 1774). Glöer and Zettler (2005: 15) discussed whether this taxon is a true subspecies or only a reaction form.

+ *Valvata disjuncta* Dollfus, 1877

Original source: Dollfus 1877: 27.

Type horizon: Upper Oligocene.

Type locality: Bessancourt (Seine-et-Oise), France.

+ *Valvata (Tropidina) donghucunensis* Pan, 1977

Original source: Pan 1977: 118, pl. 5: figs 19–20.

Type horizon: ?? (whole text in Chinese).

Type locality: Yunnan, China.

“*Valvata (Tropidina) dongshucuanensis* Pan, 1977“ mentioned in Chinese online–catalogues.

Misspelling of + *Valvata (Tropidina) donghucunensis* Pan, 1977.

+ *Valvata (Tropidina) drimensis* Pavlović, 1903

Original source: Pavlović 1903: 185–186, pl. 6: fig. 21–23. Also figured by Pavlović (1911: 605, pl. 4: figs 21–23; as “nov.spec.”!) and by Miloshevich (1984: pl. 1: figs 21–27).

Type horizon: Pliocene-Pleistocene, Metohija Series.

Type locality: Orahovac region, Metohija Basin, Southwest Kosovo.

+ *Valvata dromica* Fontannes, 1880

Original source: Fontannes 1880: 182, pl. 1: fig. 20.

Type horizon: Upper Miocene.

Type locality: bassin du Crest, Drome, France.

“*Valvata piscinalis* var. *dujardini*” mentioned in Dollfus and Dautzenberg (1886: 140) (nomen nudum).

Listed or mentioned by Lecointre (1908: 83), Dollfus (1916: 368) and by Wenz (1928b: 2445), but all without description or figure.

Locality: Middle Miocene, Faluns de la Touraine (for geography see http://www.geocaching.com/seek/cache_details.aspx?guid=4ae1307b-f29f-4a9e-a7ef-71bc1299dfbd); Départements d’Indre-et-Loire and Maine-et-Loire and an extension in the region of Rennes (Département d’Ille et Vilaine). France.

“*Valvata ecarinata* Say” (GNI)

Possibly misspelled for *Valvata tricarinata* Say, 1817.

“*Valvata elatior*” Menzel, 1904 [often cited as “*Valvata elatior* Menzel, 1900”] (Egorov 2006 Kantor et al. (2010: 73), EOL, GTI)

Error pro *Valvata (Cincinna) andreana* var. *latior* Menzel, 1904 (see there).

*Valvata erythropomatia* Hauffen, 1856

Original source: Hauffen 1856: 465, pl. 7: fig. 1.

Type locality: Cave of Görtschach / Goriče (i.e. (=”Babja Luknja” cave), Slovenia.

Remarks: Type species of *Erythropomatiana* Radoman, 1978 (see Kabat and Hershler 1993), now *Hauffenia* (**Hydrobiidae**) see Binder (1967a) and Bodon et al. (2001: 114).

+ *Valvata (Tropidina) eugeniae* Neumayr, 1875 (in Herbich and Neumayr 1875)

Original source: Herbich and Neumayr 1875: 426(26), pl. 17: fig. 1.

Type horizon: Lower Pliocene, Dacian / Lower Romanian (*Congeria*–layers).

Type locality: Vârghi (= Vargyas), Siebenbürgen (Transsilvania), Romania.

+ *Valvata euomphalus* Fuchs, 1877

Original source: Fuchs 1877: 38, pl. 4: figs 11–15.

Type horizon: Pleistocene, Chaudian (Günz glaciation).

Type locality: Livanates near Talanti, Greece.

Remarks: Type species of *Graecamnicola* Willmann, 1981 (**Hydrobiidae**), lectotype described and figured by Willmann (1981: 209–210, fig. 71A–C).

+ *Valvata euristoma* Brusina, 1902

Original source: Brusina 1902: pl. 13: fig. 43. Listed as “*Valvata eurystoma* [sic] Brusina“ in Wenz (1928b: 2468).

Type horizon: Upper Miocene, Pannonian.

Type locality: Begaljica, Serbia.

“*Valvata eurystoma*“ mentioned in Braun (1843: 123) (nomen nudum with locality)

Locality: Tertiary; Mainzer Becken, Germany.

+ *Borysthenia naticina euxinica* Gozhik, 2007

Original source: Gozhik 2007: 74–75, pl. 65: figs 2–4.

Type horizon: Upper Pleistocene (Evksinsk layer).

Type locality: near Ozernoye, district Stawropol, Russia.

*Valvata exigua* Schmidt, 1856

Original source: Schmidt 1856: 160 (footnote).

Neotype: Senckenberg Museum Frankfurt #262352.

Type locality (according to the neotype designation by Schütt (1980)): Springs near Tempetal, near railway station Agia Paraskeui, Thessaloniki, Greece.

Remarks: Considered as *Horatia (Horatia) exigua* (Schmidt, 1857) by Schütt (1962: 164); type species of *Daphniola* Schütt, 1980 (**Hydrobiidae**), see Kabat and Hershler (1993: 20), Bodon et al. (2001: 111, 175).

*Valvata exilis* Paladilhe, 1867

Original source: Paladilhe 1867b: 50–51, pl. 3: figs 27–30.

Type locality: “dans les alluvions de Lez”, Département Hérault, France.

Remarks: Type species of *Heraultia* Bodon, Manganelli & Giusti, 2001, being replaced by *Heraultiella*, see Bodon et al. (2001: 175), Bodon et al. (2002: 681), Prié (2005), or Callot-Girardi and Girardi (2013) (**Hydrobiidae**).

*Valvata eximia* Servain, 1880

Original source: Servain 1880: 155.

Type locality: Badajoz, Spain.

Remarks: Cited by Nevill (1884: 16).

“*Valvata exinia* Servain, 1880” in Vidal and Suarez (1985: 8)

Misspelling of *Valvata eximia* Servain, 1880.

+ *Valvata (Valvata) exotica* Papp, 1954

Original source: Papp 1954: 24, pl. 4: figs 5a–b.

Type horizon: Middle Miocene, Sarmatian.

Type locality: Wiesen, Eisenstadt (Sopron) Basin, Burgenland, Austria.

Remarks: Introduced for *Valvata pseudoadeorbis* Sinzow, 1880 sensu Papp (1939: 352).

*Valvata fagoti* Fagot, 1881

Original source: Fagot 1881: 141 (cited as *Valvata fagoti* Bourguignat).

Type locality: “Saint-Pardoult (Charente-Inférieure), France.

+ *Liratina fahaniuensis* Youluo, 1978 (name of author according to the type catalogue of Nanjing Institute)

Original source: Youluo 1978: 32–33, pl. 4: figs 6–8.

Type horizon: Lower Tertiary.

Type locality: coastal region of Bohai, China.

+ *Valvata falsani* Locard, 1883

Original source: Locard 1883a: 80, pl. 3: figs 12–14. The name is referred to “*Valvata ? Falsani* (*Lithoglyphus*), Tournouër, 1876, Mss. in Falsan: Introd. Faune Meximieux, p. 34” (i.e. Falsan 1875: 164), a nomen nudum. Listed as *Valvata falsani* Locard, 1888 (type error?) with further references by Wenz (1928b: 2457).

Type horizon: Middle Pliocene, Plaisencien.

Type locality: Pérouges, Département de Ain, France.

Remarks: Cited as “*Valvata falsani* (*Lithoglyphus*) Tournouër, 1876“ in Locard (1883a: 80) with detailed description and as type species for *Michaudia* Locard, 1883 (page 81 and 82) (**Lithoglyphidae**).

*Cincinna falsifluviatilis* in Anistratenko and Anistratenko (2001) (nomen nudum ?)

Original source: Anistratenko and Anistratenko 2001: 139–140, fig. 110 (name attributed to Starobogatov, but ICZN Art. 50.1.1 is not satisfied).

Type locality: not provided “Belgien, England, Deutschland, Russland”.

Types: Not specified.

Remarks: Established as a replacement name for “*Valvata fluviatilis* sensu Westerlund, 1886 (non Colbeau, 1859) “. Kantor et al. (2011: 67) stated that this name is erroneously attributed to 1996 by Starobogatov et al. (2004). Kantor and Sysoev (2005) overlooked the description of the species in Anistratenko and Anistratenko (2001) and thus concluded that the name is not available.

However, a “sensu” name is not an available name, and only an available name can be replaced for nomenclatural reasons (see Glossary of the Code, new replacement name, “a name established expressly to replace an already established name”). Westerlund (1886) just used Colbeau’s name and misapplied it to a species that later turned out to belong to a different species, thus Westerlund (1886) did not establish a new name. Consequently there was no name for which a new replacement name could be established. If Starobogatov or Anistratenko and Anistratenko (2001) intended to do that, the act was unsuccessful and it must be looked if the name was established as a regular new name. However, types were not specified (*fide* Kantor et al. 2011: 67), thus the conditions of ICZN Art. 16.4 probably were not met (after 1999 name–bearing types must be specified in the original source). This means that the name *Cincinna falsifluviatlis* is a nomen nudum and not available.

*Helix fascicularis* Gmelin, 1791

Original source: Gmelin 1791: 3641 (based on Schröter 1779: pl. 6: fig. 11, the work is not avaible for nomenclatorical purposes, since it does not use binomen).

Type locality: Thüringen, Germany.

Remarks: Menke (1845: 120) considered the name as a synonym to *Valvata piscinalis* (Müller, 1774).

*Valvata fennica* Westerlund, 1897

Original source: Westerlund 1897a: 197 (no figure, see Gude 1912).

Type locality: “Fennia ad Vosnessenje prope Onega”, Finland.

“*Valvata tricarinata* f. *fercomfuss*” (GNI)

Misspelling of *Valvata tricarinata* var. *perconfusa* Walker, 1917.

*Valvata (Tropidina) fezi* Altimira, 1960

Original source: Altimira 1960: 14, fig. 5.

Type locality: Fuente Roble, Yémeda, Cuenca, Spain

Variability figured by Callot-Girardi and Girardi (2013).

Remarks: Boeters (1988), Bodon et al. (2001: 175), Arconada and Ramos (2006), and Callot-Girardi and Girardi (2013) agree that this is a **hydrobiid** taxon.

+ *Valvata filosa* Whiteaves, 1885

Original source: Whiteaves 1885: 25, pl. 3: figs 7, 7a.

Type horizon: Cretaceous.

Type locality: Pincher Creek, North–west Territory, Canada.

*Valvata piscinalis* var. *fluviatilis* in Colbeau (1859: 11) (nomen nudum)

Remarks: Drouët (1867: 94) cited the name as a synonym of a name presented as *Valvata contorta* as published by Menke (1849: 116), without description for *fluviatilis*, and with a question mark concerning the synonymy. Accordingly, Drouët (1867) did not make the name available.

*Valvata fluviatilis* Colbeau, 1868

Original source: Colbeau 1868: 93, 110, pl. 2: fig. 16).

Type locality: Belgium.

Remarks: According to ICZN Art. 12.2.7 Colbeau (1868) made the name available in combination with a figure, and no reference was given to Drouët (1867).

Anistratenko and Anistratenko (2001: 139–140) cited the opinion of Ya.I. Starobogatov, that *Valvata piscinalis* var. *fluviatilis* Colbeau, 1859 is a synonym of *Cincinna contorta*. Most of European malacologists under the name *Valvata fluviatilis* identified separate species, for which Anistratenko & Anistratenko proposed a new name *Cincinna (Cincinna) falsifluviatilis* attributed to Starobogatov, but the name is probably not available (see there).

*Turbo fontinalis* Pulteney, 1799

Original source: Pulteney 1799: 45.

Type locality: Dorsetshire, England.

Remarks: “*Turbo fontinalis*, Pult.” in Montagu (1803: 348–351, pl. 22: fig. 4) listed as a junior synonym of *Valvata piscinalis* (Müller, 1774). “*Valvata fontinalis*, Mont.“ mentioned in Leach 1852: Moll. Brit. Synop., p. 206.

“*Valvata foraminis* Braun, 1843“ mentioned in Behrendt (1863: 41).

Remarks: see Friedl (1871: 74: “Prof Alexander Braun in Berlin theilt mir bezüglich der von Dr. G. Behrendt in der Schrift: Die Diluvial-Ablagerungen der Mark Brandenburg, Berlin, 1863, S. 41 erwähnte *Valvata foraminis* (*Valvata macrostoma*) mit, dass er zwar eine *Valvata eurystoma* und *paludinaeformis*, niemals aber eine *Valvata foraminis* aufgestellt habe, dieser Name werde wohl nur durch irriges Entziffern einer von ihm undeutlich geschriebenen Namensetiquette in die Wissenschaft, aus welcher er hiermit definitiv ausgemerzt wird, eingeschlichen sein.” [Prof. Alexander Braun in Berlin informs me concerning *Valvata foraminis*, which is mentioned in the paper “Die Diluvial-Ablagerungen der Mark Brandenburg, Berlin, 1863, p. 41“ by Dr. G. Behrendt. He has indeed described *Valvata eurystoma* and *paludinaeformis*, but never a *Valvata foraminis*. The latter name has been introduced to science probably by an erroneous reading of an unclear name label and thus should be now finally omitted.].

+ *Valvata fossaruliformis* Brusina, 1902

Original source: Brusina 1902: pl. 13: figs 9–13.

Type horizon: Upper Miocene, Pannonian.

Type locality: Kenese (at Lake Balaton), Hungary.

+ *Valvata fragilis* Yü, 1965

Original source: Yü 1965: 46, pl. 1: figs 8–10.

Type horizon: Middle - Upper Eocene, upper part of the Yuanchü Chun.

Type locality: Yuanchü, Shansi, China.

*Valvata frigida* Westerlund, 1873

Original source: Westerlund 1873: 436–437.

Type locality: “Från Naustejaur I Pite Lappmark” (near Naustejaur in the Samen region), Sweden.

Syntypes: Naturalhistoriska Museet Göteborg (AN 4677), figured by Vinarski et al. (2013b: fig. 3D–E).

Remarks: Falkner et al. (2001) and Glöer (2002) considered this species as a synonym of *Valvata sibirica* Middendorff, 1851.

+ *Aphanotylus fuchsi* Brusina, 1894

Original source: Brusina 1894: 186–187.

Type horizon: Upper Miocene, Pannonian (horizon of *Lyrcæa* (*Melanopsis*)).

Type locality: Kenese at Lake Balaton, Hungary.

“*Valvata piscinalis antiqua* var. *fuhrmanni*” mentioned in Piaget (1914)

Original source: Piaget 1914: 164, pl. 1: fig. 11.

Locality: Lac de Neuchatel, Switzerland.

Remarks: Name proposed as an infrasubspecific rank unavailable and thus not available under ICZN Art. 10.4, 45.5.

“*Valvata furhrmanni*” (GNI, ION)

Misspelling of *Valvata fuhrmanni* Piaget, 1914.

+ *Valvata furlici* Brusina, 1897

Original source: Brusina 1897: 25, pl. 13: figs 23–25.

Type horizon: Upper Miocene - Pliocene, Pannonian (Portaferrian).

Type locality: Okrugljak in Zagreb, Croatia.

+ *Valvata (Cincinna) fuxinensis* Yü, 1987

Original source: Yü X. 1987: 50, figs ?? (not seen; listed in Zoological Record 134(9): #4045).

Type horizon: Lower Cretaceous.

Type locality: Western Liaoning Province, China.

*Valvata (Cincinna) gafurovi* Izzatullaev, 1977

Original source: Izzatullaev 1977: 948–949, 1 textfig.

Type locality: Gorno-Badakhmanskaya Autonomous Republic, Lake Sylykty-Sai (areal about 300 m^2^), 10 km south–westward from Kyzil-Rabat, on depth 2–3 m, Tajik SSR.

Holotype: Zoological Institute of the Russian Academy of Sciences, St. Petersburg, Nr. 1 in systematic catalogue under the name.

*Valvata (Cincinna) gaillardoti* Germain, 1911

Original source: Germain 1911: 66.

Type locality: Environs de Saida, Syria.

Remarks: Currently *Islamia gaillardoti* (Germain, 1911) (**Hydrobiidae**), see Bodon et al. (2011: 175).

*Valvata gallica* Locard, 1889

Original source: Locard 1889: 23–25.

Type localities: “Canal de la Marne au Rhin”; Boulogne–sur-Seine, près de paris; Argenteuil, Versailles, etc. (Seine-et-Oise); bar–sur-Seine (Aube); Canal du Nivernais, Moulins (Allier); Auxonne (Côte–d’Or); Chalon–sur-Saône (Saône-et-Loire); les alluvions du Rhône, au nord de Lyon (Rhône, Ain); le lac du Bourget, près d’Aix–les-Bains (Savoie); l’ile de Trontemoult, à Nantes (Loire-Inférrierue) [col. Bourguignat]; les environs e Bayonne (Basses Pyrénées) [col. Bourguignat]; etc., all France.

+ *Valvata piscinalis* var. *gaudryana* Mortillet, 1863

Original source: Mortillet 1863: 295 and 592: textfig. 2.

Type horizon: Middle Pliocene.

Type locality: Joinville–le-Pont, sablière Deligny, Montreuil, France.

Remarks: Often cited as “*Valvata piscinalis* var. *gaudryana* Tournouër, 1866» (e.g. Wenz 1928b: 2434), but Mortillet described this species before Tournouër (1866: 791), who only listed the species name.

“*Valvata genuina* Ziegler“ mentioned in Jan (1830: 6) (nomen nudum)

“*Valvata genuina* Ziegler“ mentioned in Menke (1830: 46) (nomen nudum)

*Valvata genuina* Menke, 1845

Original source: Menke 1845: 128 (attributed to Ziegler, but ICZN Art. 50.1.1 is not met).

Type locality: Island Martinique, Caribbean (according to Menke 1845: 128).

Remarks: As outlined by Welter-Schultes (2012b: 96) Ziegler was a shell dealer from Vienna (Austria) and sent labeled shells with new names to researchers, who then described the new species and attributed the names to him. After 1905 the malacologists agreed that he (and other shell dealers as well) should not be regarded as authors of names, because they had in most cases not done any scientific work.

*Valvata (Cincinna) geyeri* Menzel, 1904 (often cited as Menzel 1900)

Original source: Menzel 1904a: 78–79, with textfigure. Also described in detail and figured by Menzel (1904c: 288, pl. 14: figs 41–48).

Type locality: “Weissen See” (Weißensee) near Füssen in Bavaria, Germany.

Specimens from the type locality were figured bei Glöer (2002: 192, fig. 221: 1–4), Syntypes (Senckenberg Museum Frankfurt 140672/7) were figured by Boeters and Falkner (1998: pl. 8: figs 11–12).

Remarks: According to Menzel (1904a, 1904c) and Steusloff (1922) only subfossil. Uhl (1926) considered continuity between the “forms” *piscinalis, alpestris, geyeri*, and *antiqua*. Glöer (2002: 184, 192) regarded *Valvata geyeri* as a subspecies of *Valvata piscinalis* (Müller, 1774). So–called *Cincinna geyeri* from the Aussensee in Schwerin (and those of Russian authors too) are probably varieties (ecomorphs) of *Valvata piscinalis*.

+ *Valvata gibbulaeformis* Brusina, 1902

Original source: Brusina 1902: pl. 13: figs 14–15.

Type horizon: Upper Pliocene, Dacian.

Type locality: Aita Seacă, Romania.

“*Valvata antiqua gigas* Goretzki, 1956” (GNI, ION) – unknown.

+ *Valvata giraudi* Dollfus, 1908

Original source: Dollfus 1908: 20, textfig. 2.

Type horizon: Lower Miocene, Aquitanian.

Type locality: Montaigu–de-Blin, Département de Allier, France.

+ *Valvata glacialis* Westerlund, 1881

Original source: Westerlund 1881: 48 (name only), 67–68 (description).

Type horizon: subfossil.

Type locality: in glacier of Scania, a province in Sweden.

*Valvata globulina* Férussac, 1807 (in Férussac and Férussac 1807)

Original source: Férussac and Férussac 1807: 128.

Type locality: France (Férussac’s specimens) and Germany: Weimar (specimens from Müller 1774: 179)

Remarks: (1) Mentioned by Férussac and Férussac (1807) without description, only with a name “*Ner. Minuta*” given in the column headed “Muller”. The names of the authors in the “Concordance” list were meant to refer to single works each name (Welter-Schultes and Audibert 2013: 21, *Sphaerium ovale*). “Muller” referred to Müller’s 1774 work, and “*Ner. Minuta*” to the description of *Nerita minuta* Müller, 1774 on p. 179. This was a bibliographical reference that made the name *Valvata globulina* available under ICZN Art. 12.2.1.

(2) Paladilhe (1866: 170) did not seem to have understood the bibliographical nature of the citation “*Ner. Minuta*” by Férussac and Férussac (1807). Paladilhe cited this name as “*Valvata minuta*” (this should have said “*Nerita minuta*”), because Paladilhe 1866 gave the number 167, not *Valvata minuta* as used by Férussac and Férussac (1807: 128 under No. 171), which had nothing to do with the name Paladilhe (1866) had in mind - by stating “..not “*Valvata minuta* Draparnaud“ mentioned in the footnote.

(3) In addition, Paladilhe (1866) provided several more citations, for example Gassies (1849: 183), where the name *Valvata minuta* was also presented with a descrption, so the name was clearly made available before 1866.

(4) Nevertheless, starting with Germain’s (1931: 675) citation as “*Valvata globulina* Paladilhe, 1866“, most subsequent authors used this version (e.g. Kadolsky 2008).

(5) According to Bodon et al. (2001: 175, 202) and Kadolsky (2008: 116) this taxon (finally cited as “*Islamia globulina* (Paladilhe, 1866)”) is a **hydrobiid**.

+ *Valvata gradata globulosa* Jekelius, 1944

Original source: Jekelius 1944: 117, pl. 43, figs 18–20.

Type horizon: Middle Miocene, Sarmatian.

Type locality: Soceni, Banat, Romania.

Remarks: Harzhauser et al. (2002: 103, pl. 8: figs 3–8) still listed this taxon as a valvatid, whereas Bandel (2010: 107) considered synonymy with *Jekeliella gradata* (Fuchs, 1870) (**Hydrobiidae**) (see also below).

“*Valvata glohulina* Paladilhe, 1866” (among Valvatidae) mentioned in Bech i Taberner and Altimiras I Roset (2003: 841).

Misspelling of *Valvata globulina* Paladilhe, 1866 = *Islamia globulina* (Paladhile, 1866) (**Hydrobiidae**).

+ *Valvata (Cincinna) goldfussiana* Wüst, 1901

Original source: Wüst 1901: 236, pl. 1: figs 43–46.

Type horizon: Pliocene – Pleistocene.

Type locality: Thüringen, Germany.

Remarks: Currently considered as *Borysthenia goldfussiana* (Wüst, 1901).

“*Valvata goryi*” mentioned in Van Damme (1984: 13)

“*Valvata nilotica* is closely related to the S.W. Asian *Valvata goryi*”.

Mismatch with *Bithynia goryi* Bouguignat, 1856 (p. 185; type locality: Nile valley).

*Valvata gracilis* Locard, 1889

Original source: Locard 1889: 36 (cited as *Valvata gracilis* Locard, 1886).

Type localities: Environs de Cherbourgh, dans la Manche; Brest, dan le Finisère; Issoudun, dans l’Indre (Loc.); la Maine, à Angers, dan le Maine-et-Loire (col. Bouguignat), all France.

+ *Valvata gradata* Fuchs, 1870

Original source: Fuchs 1870b: 536, pl. 21: figs 13–16.

Type horizon: Upper Miocene, Pannonian (Transdanubian).

Type locality: near Tihany at Lake Balaton, Hungary.

Remarks: Bandel (2010: 107, pl. 10, figs 115–119) classified the species among *Jekeliella* (**Hydrobiidae**).

+ *Valvata graeca* Fuchs, 1877

Original source: Fuchs 1877: 38, pl. 5: figs 6–10.

Type horizon: Pleistocene, Chaudian (cf. Gillet et al. 2003).

Type locality: Livanates near Talanti, Greece.

Remarks: Classified among *Graecamnicola* by Willmann (1981: 211) and lectotype figured (figs 72A–B), a view confirmed by Bandel (2010: 106, pl. 9: figs 108–110, pl. 11: figs 130–134) (**Amnicolidae**).

“*Valvata granifera*”.

Cited by Leunis (1860: 637) and Westerlund (1879: 18) as an example of confusion of a larval envelop of a phryganid **trichopteran** insect with a valvatid shell. Probably a misspelling of *Valvata arenifera* Lea, 1834, where this is the case, indeed (see there).

*Valvata contorta gratiosa* Drouët, 1867

Original source: Drouët 1867: 94.

Type locality: Côte d’Or, France.

+ *Valvata gregaria* Bukowski, 1895

Original source: Bukowski 1895: 25, pl. 8: fig. 7–8.

Type horizon: Marl and/or clay.

Type locality: south of Rhodos Island, Greece.

Remarks: Lectotype designated and figured by Willmann (1981: 77, fig. 25).

+ *Valvata gregorii* Robinson, 1915

Original source: Robinson 1915: 649, textfigs 1d–e.

Type horizon: Jurassic, Morrison Formation.

Type locality: 4 miles NE of Black Falls, Wand Terrace, Tuba, Arizona, USA.

Holotype: Yale Peabody Museum 17849, a plastoholotype at National Museum of Natural History (http://collections.si.edu/search/record/nmnhpaleobiology_3307304).

Remarks: Cossmann (1921) considered the species a member of *Ampullaria* (**Ampullariidae**, but Henderson (1935: 189) still listed it among the Valvatidae.

+ *Valvata fluviatilis groeberi* Fuhrmann, 1990

Original source: Fuhrmann 1990: 152, pl. 1: figs 1–7, pl. 2: figs 1, 5.

Type horizon: Interglacial.

Type locality: Gröbern (Kreis Gräfen-Hainichen), north of Leipzig, Germany.

*Valvata grubei* mentioned in B. Dybowski and W. Godlewski (1870: 199) (nomen nudum).

*Valvata grubei* Dybowski, 1875

Original source: W. Dybowski 1875: 28, 31–33, pl. 2: fig. 6–10 (shell), pl. 8: figs 9–12 (radula). Name attributed to Benedykt Dybowski, nut ICZN Art. 50.1.1 is not met.

Type locality: Lake Baikal.

“*Valvata grubii* Dyb.“ mentioned in Dybowski (1886: 107)

Misspelling of *Valvata grubei* Dybowski, 1875.

“*Valvata guatamalensis*” (GNI)

Misspelling of *Valvata guatemalensis* Morelet, 1851 (which is not listed in GNI).

*Valvata guatemalensis* Morelet, 1851

Original source: Morelet 1851: Vol. II: 22, no. 138.

Type locality: Río Michatoya, near the port of Istapa [Puerto Ixstapa], Dept. Esquintla, Guatemala.

Remarks: Considered as *Cochliopa guatemalensis* (Morelet, 1849) (**Hydrobiidae**) by Murray and Roy (1968).

+ *Cincinna (Atropidina) guriana* Gozhik, 2007

Original source: Gozhik 2007: 79, pl. 71: figs 1–2.

Type horizon: Pleistocene (Guriy Sediment).

Type locality: near Syvash (shallow lagoons on the west coast of the Sea of Azov) northeastern coast of the Crimean Peninsula, Ukraine.

*Valvata hagenmulleri* Hagenmüller, 1884 (non Caziot, 1902) [as spelled in the headline, no subsequent use] or *Valvata hagenmuelleri* Hagenmüller, 1884 [as cited in line 2 and in the index/page 383 of the volume; also GNI and ION]. According to ICZN Art. 50.1.1 the correct spelling depends on the usage by the first reviser.

Original source: Hagenmüller 1884: 214 (often cited as “*Valvata hagenmülleri* Bourguignat”, but ICZN Art. 50.1.1 is not satisfied).

Type locality: Seyabouse, Algeria.

Remarks: Boettger (1905: 120) considered the taxon to belong to the **hydrobiid** genus *Daudebardiella* Boettger, 1905 because of the oblique aperture.

*Valvata hagenmulleri* [sic] Caziot, 1902 (non Hagenmüller, 1884)

Original source: Caziot 1902: 325.

Type locality: valley of Tavignano, Corsica, France.

Remarks: often cited as *Valvata hagenmuelleri* Caziot, 1902 (e.g. WMSDB).

+ *Valvata (Jekeliusiana) oecsensis halavatsi* Gozhik, 2002

Original source: Gozhik 2002: 49–50, pl. 2: figs 5–11.

Figured by Gozhik (2007: 81, pl. 70: figs 1–4).

Type horizon: Miocene, Pontian?? (paper not seen).

Type locality: ?? (paper not seen).

*Valvata (Cincinna) halopea* Westerlund, 1894

Original source: Westerlund 1894: 199.

Type locality: Lake Palavesi near Kuopio, Finland.

+ *Valvata (Cincinna) hanjianggensis* Yü, 1977 (in Yü and Wang 1977)

Original source: Yü and Wang 1977: 14, pl. 1: fig. 13.

Type horizon: Upper Cretaceous.

Type locality: Jiangsu Province, China.

*Cincinna hankensis* Prozorova, 1988

Original source: Prozorova 1988: 1736–1738.

Type locality: Small lake near the shore of Khanka Lake, (= Xīngkǎi Hú, Lake Xingkai) is located in East Russia/Northeast China.

Holotype: Zoological Institute of the Russian Academy of Sciences, St. Petersburg, Nr. 1 in systematic catalogue under the name.

*Valvata hebraica* Lesson, 1832

Original source: Lesson 1832: 347, pl. 13: fig. 8.

Type locality: New Guinea.

Remarks: Baird (1850: 7) and also currently (GNI) considered as *Cyclotus hebraicus* (Lesson, 1832) (**Cyclophoridae**).

+ *Liratina hedobia* Youluo, 1978 (name of author according to the type catalogues of Nanjing Institute)

Original source: Youluo 1978: 32, pl. 4: figs 9–14.

Type horizon: Lower Tertiary.

Type locality: coastal region of Bohai, China.

+ *Valvata heidemariae* Willmann, 1981

Original source: Willmann 1981: 158, textfig. 56A–C.

Type horizon: Pliocene, Middle Vokasia-Formatin.

Type locality: Vokasia Tal, Island of Kos, Greece.

+ *Valvata (Cincinna) helicelloides* Huckriede, 1967

Original source: Huckriede 1967: 164, pl. 23: figs 32a-41.

Type horizon: Lower Cretaceous.

Type locality (according to Arkell 1941): Middle Purbeck of Ridgeway, England, U.K.

Remarks: Replacement name of *Valvata helicoides* Loriol & Jaccard, 1865.

*Valvata lewisi* var. *helicoidea* Dall, 1905

Original source: Dall 1905: 123, pl. 2: figs 1–2.

Type locality: Lake Bennett, Yukon Territory; Old Fort Yukon, Alaska.

Syntype: US National Museum No. 180304.

Remarks: Also occurring in Siberia (Kantor et al. 2011: 68). It is possible that this American species is identical with the Siberian ones, since there was a land–bridge during ice–ages (or by transport via water birds). Accordingly, the generic classification needs to be tested.

“*Valvata sincera* var. *helicoidea* Dall, 1905” (WMSDB)

Erroneous rendition of *Valvata lewisi* var. *helicoidea* Dall, 1905.

“*Valvata helicoides*“ mentioned in Fischer (1855: 581) (nomen nudum)

+ *Valvata helicoides* Stoliczka, 1862

Original source: Stoliczka 1862: 535, pl. 17: fig. 5.

Type horizon: Upper Miocene, Pannonian.

Type locality: north of Eszterthal, region of Lake Balaton, Hungary.

Remarks: SEM of shell and (typical valvatid) protoconch figured by Harzhauser and Binder (2004: Pl. 3, Figs 1–3, 4–5). However, according to Bandel (2010: 98, pl. 3: figs 32–37; pl. 4: figs 38–39) the species (cited as “*Valvata helicinoides* (Stoliczka, 1862)” should be classified among the heterobranch **Omalogyridae**. The latter view appears improbable, however, since Omalogyridae is a purely marine family, whereas valvatids are freshwater inhabitants (and the fossil samples from which this species was described are freshwater deposits).

+ *Valvata helicoides* Loriol & Jaccard, 1865

Original source: Loriol and Jaccard 1865: 93 (in issue of journal, 33 in reprint), pl. 2: fig. 21–24. Attributed to Forbes, but ICZN Art. 50.1.1 is not satisfied.

Type horizon: Lower Cretaceous.

Type locality (according to Arkell 1941): Middle Purbeck of Ridgeway, England, U.K.

Remarks: (1) Refigured and synonymy by Arkell (1941: 89, figs 14 a, b, 60, 61, 62). He explained: “Edward Forbes was preparing a monograph on the invertebrate fauna of the Purbeck Beds when his work was cut short in 1854 by his death at the age of 39. All that appeared was a preliminary account, in which the genera *Viviparus*, *Valvata*, *Lymnaea*, *Planorbis*, *Hydrobia*, *Physa*, *Melania*, *Cyclas*, and *Unio* were recorded, and also many marine genera, but no species were mentioned. A number of MS. names were introduced by him on labels and in the Survey catalogues, and some of them have been used by other authors, but if any manuscript or type specimens existed they have disappeared“.

(2) *Valvata helicoides* Loriol & Jaccard, 1865 is a junior homonym of *Valvata helicoides* Stoliczka, 1862, thus Huckriede (1967: 164) provided a replacement name, *Valvata helicelloides*.

(3) However, “*Valvata helicoides* de Loriol, 1865“ was designated as type species of *Provalvata* Bandel, 1991, the type genus of Provalvatidae Bandel, 1991.

(4) Zhu (1994: 92, pl. 3: figs 8–15) used “*Valvata helicoides* (Forbes)” for a taxon in the genus *Valvata*.

+ *Valvata kupensis* var. *hellenica* Tournouër, 1877 (non *Valvata (Cincinna) hellenica* Westerlund, 1898)

Original source: Tournouër 1877: 55.

Type horizon: Pliocene, Sarakos Formation.

Type locality: Island of Rhodos, Greece.

Remarks: SEM photos of the protoconch (cf. Bandel 2010: 100, pl. 4: figs 49–51) confirm this species as a true valvatid.

*Valvata (Cincinna) hellenica* Westerlund, 1898 (non *Valvata kupensis* var. *hellenica* Tournouër, 1877)

Original source: Westerlund 1898: 179.

Lectotype (#4667a) in the Naturhistoriska Museet Göteborg, Sweden, together with two paralectotypes (#4667b), designated by Reischütz and Sattmann (1993: Heldia 2: 51–52).

Type locality: Vyteria in Arkadia, Greece.

Remarks: Currently regarded as junior synonym of *Daphniola exigua* (Schmidt, 1856) (**Hydrobiidae**), see Bodon et al. (2001: 111).

*Valvata helvetica* Locard, 1889

Original source: Locard 1889: 41–42 (attributed to Bourguignat; but ICZN Art. 50.1.1 not met).

Type locality: Lac Morat, Switzerland.

+ *Valvata hidasensis* Kókay, 1967

Original source: Kókay 1967: 84 (Hungarian), 90 (German), pl. 8: figs 3–6.

Type horizon: Middle Miocene, Badenian.

Type locality: Hidas (Komitat Baranya), Bakony mountains, Hungary.

Holotype: Hungary National Museum Natural History #M.66.965.

Remarks: Kókay (2006: 43) himself later considered this taxon as *Sandbergerina hidasensis* (**Truncatellidae**).

*Valvata (Costovalvata) hirsutecostata* Poliński, 1929

Original source: Poliński 1929: 138.

Type locality: Lake Ohrid, 20–30 m, Macedonia (only this side of the lake was studied).

Remarks: Anatomy has been studied by Rath (1986: 103ff). According to molecular data of Hauswald et al. (2008) this taxon is a variety (ecomorph) of *Valvata rhabdota*, whereas Welter-Schultes (2012a: 43 – Hauswald et al. was not cited in that paper) still considers a valid taxon.

“*Valvata histricus*” (GNI)

Probably confused with *Paludina histrica* Gould, 1861; currently *Viviparus histricus* (Gould, 1861).

+ *Valvata hoernesi* Penecke, 1886

Original source: Penecke 1886: 38, pl. 10 (resp. 7 in reprint): fig. 3 (as *Valvata hörnesi* dedicated to Rudolf Hörnes, an Austrian from Vienna; cf. ICZN Art. 32.5.2.1).

Type horizon: Pliocene – Pleistocene, Dacian – Romanian.

Type locality: Repušnica, Slavonia, Croatia.

*Valvata cristata hokkaidoensis* Miyadi, 1935

Original source: Miyadi 1935: 61–62, pl. 3: fig. 4.

Type locality: Tôro–ko, Hokkaido, Japan.

Holotype: According to Kantor et al. (2011: 68) deposited at Ôtsu Hydrobiological Station, now known as the Center for Ecological Research, Kyoto University.

Remarks: Live movie at http://www.youtube.com/watch?v=f6p3w4WWgG4

+ *Valvata homalogyra* Brusina, 1874: 90.

Original source: Brusina 1874: 90. Figured by Brusina (1897: 25, pl. 14: figs 7–9) and Kókay (2006: 32, pl. 3: figs 11–12).

Type horizon: Neogene, layer of grey marl.

Type locality: Ruduša, Dalmatia, Croatia.

+ *Valvata utahensis horatii* Baily & Baily, 1951

Original source: Baily and Baily 1951: 50, pl. 4: figs 5–5a.

Type horizon: Pleistocene.

Type locality: Lifton, Ideal Beach, Great Basin, Idaho, USA.

Holotype: Academy of Natural Sciences of Drexel University, Philadelphia #187689.

+ *Valvata (Valvata) huailinensis* Yü & Pan, 1982

Original source: Yü and Pan 1982: 191, figs 9–13 (not seen, listed in Zoological Record 1982(A2): #1479).

Type horizon: Eocene.

Type locality: Uhuo Xian, Hebei Province, China.

Holotype depicted at: www.nimrf.net.cn/ept/eptDataDetail.action?ptzyh=2332C0001000005344

*Valvata humeralis* Say, 1829

Original source: Say 1829: 244 (in issue of journal, 22 in reprint) (vernacular name: “glossy valvata”).

Type locality: Mexico.

Types: not traced.

Remarks from www.iucnredlist.org/apps/redlist/details/189646/0: Recent surveys of this species across western USA found no morphologically similar specimens to the holotype, which is from Mexico. This suggests that individuals thought to be *Valvata humeralis* from the US may be a different species, and *Valvata humeralis* may actually be restricted to Mexico (Hovingh 2004, Miller et al. 2006). Hovingh (2004) proposed that US specimens should be classified as *Valvata californica*. However, this assessment follows the previously established view of a US/Mexican distribution until further confirmation of the taxonomic status.

For genetic differences to *Valvata utahensis* see Miller et al. (2006).

+ *Aphanotylus humeratus* Youluo, 1978 (name of author according to the type catalogues of Nanjing Institute)

Original source: Youluo 1978: 34, pl. 3, figs 7–9.

Type horizon: Lower Tertiary.

Type locality: coastal region of Bohai, China.

“*Valvata humerosa* Say, 1829“ mentioned in Menke (1834: 128).

Misspelling of *Valvata humeralis* Say, 1829.

+ *Valvata humilis* Fritzsche, 1924

Original source: Fritzsche 1924: 23, pl. 2: fig. 6.

Type horizon: Cretaceous.

Type locality: limestone of Miraflores, near Potosi, Bolivia.

+ *Valvata (Cincinna) hydrobiaeformis* Cossmann, 1919

Original source: Cossmann 1919: 119–120, pl. 4: figs 54–55.

Type horizon: Eocene.

Type locality: Bois-Gouët, Lower Loire, Bretagne, France.

+ *Valvata idahoensis* Taylor, 1981 (in Taylor and Smith 1981)

Original source: Taylor and Smith 1981: 358, pl. 4: fig. 1–12.

Type horizon: Pliocene.

Type locality: Honey Lake, Lassen County, California, USA.

Remarks: Replacement name for *Valvata multicarinata* Yen, 1946 (non Hislop, 1860).

+ *Valvata ilici* Brusina, 1894

Original source: Brusina 1894: 181 (footnote 2). Figured by Brusina (1897: 26, pl. 13: figs 26–27) and Brusina (1902: pl. 13: figs 24–27).

Type horizon: Upper Miocene – Pliocene, Pannonian (Portaferrian).

Type locality: Okrugljak, near Zagreb, Croatia.

+ *Valvata sincera illinoisensis* Baker, 1930

Original source: Baker 1930: 189, textfig. 1.

Type horizon: Pleistocene.

Type locality: near the west end of Crystal McHenry County, Illinois, USA.

*Valvata imhofi* Clessin, 1887

Original source: Clessin 1887: 776–777: fig. 510 (among section *Tropidina*).

Type locality: Lake Garda, Trentino, Italy.

“*Valvata imperialis* Bourguignat, 1884” (GNI, ION)

Probably confused with *Viviparus imperialis* Bourguignat, 1884.

“*Valvata impura* Stentz / Ziegler“ (GNI: “*Valvata impura* Ziegl.”)

cited in Villa, A. and Villa, J.B (1841: 33) and by Brusina S. (1870: 38)

cited by Systax data–base for the Löbbecke Museum Düsseldorf with locality “Austria, Carinthia, Klagenfurt”.

*Paludina impura obtusa* Menke, 1830 was regarded as synonym to *Valvata contorta subovata* Menke, 1845 by Menke (1845: 116)

Remarks: As outlined by Welter-Schultes (2012b: 96) Ziegler was a shell dealer from Vienna (Austria) and sent labeled shells with new names to researchers, who then described the new species and attributed the names to him. After 1905 the malacologists agreed that he (and other shell dealers as well) should not be regarded as authors of names, because they had in most cases not done any scientific work.

+ *Valvata incerta* Yen, 1947

Original source: Yen 1947: 272, pl. 43: figs 4a–c.

Type horizon: Pliocene.

Type locality: Salt Lake Group,” about 14 miles northwest of Logan, Northern Utah, USA.

*Valvata inconspicua* Adams, 1850

Original source: Adams 1850: 131–132.

Type locality: Jamaica.

Remarks: The types from the Museum of Camparative Zoology (Harvard) were studied and the holotype (#185089) was figured by Johnson and Boss (1972: 205–206; pl. 41: fig. 7)

+ *Valvata indecisa* Cossmann, 1924

Original source: Cossmann 1924: 8, pl. 5: figs 36–37. Referred to “*Valvata indecisa*” mentioned in Cossmann (1921: 170) (nomen nudum)

Type horizon: Paleocene, Danian.

Type locality: Belgium.

Remarks: Esu (1984: 31) considered this species closely related to *Islamia sarda* Esu, 1984 (**Hydrobiidae**).

+ *Valvata inflata* Sandberger, 1875

Original source: Sandberger 1875: 746.

Type horizon: Upper Pliocene.

Type locality: Bligny, Département de Merne, France.

+ *Valvata inflexa* Deshayes, 1862

Original source: Deshayes 1861–1863, Vol. 2: 527, pl. 36: figs 19–22.

Type horizon: Eocene.

Type locality: Bernon, près Epernay Basin de Paris, France.

*Valvata tricarinata* var. *infracarinata* Vanatta, 1915

Original source: Vanatta 1915: 104–105: figs 1–2.

Type locality: White Pond, NJ (USA).

Holotype: Academy of Natural Sciences of Drexel University, Philadelphia (ANSP) No. 12087.

*Valvata (Cincinna) innesi* Pallary, 1901

Original source: Pallary 1901a: 243, pl. 1: figs 9–11.

Type locality: l’Ouady Feiran, Sinai Peninsula, Egypt.

*Valvata (Ohridotropidina) relicta interlithonis* Hadžišce, 1956

Original source: Hadžišce 1956: 59–62, Abb. 1 (3 variants of shell) Abb. 2 (radula).

Type locality: Lake Ohrid, Macedonia.

*Valvata intermedia* Locard, 1889

Original source: Locard 1889: 50 (attributed to Bourguignat, cf. ICZN Art. 50.1.1).

Type locality: Lago di Como near Bellagio, Lombardia, Italy.

Remarks: The WMSDB regards the taxon as synonym to *Valvata cristata* Müller, 1774.

+ *Borysthenia intermedia* Kondrashov, 2007

Original source: Kondrashov 2007: 513, pl. 5, figs 1–2.

Type horizon: Middle Pleistocene of Oka-Don Plain.

Type locality: Novokhopersk, right bank of Khoper river, Voronezh region, Russia.

+ *Valvata interposita* De Stefani, 1880

Original source: De Stefani 1880: 48, 136, pl. 3(4): fig. 13.

Type horizon: Upper Pliocene.

Type locality: Pacciona, Val de Tresa (Verri) near Lago Lugano, Italy.

Remarks: Delafond and Depréret (1893: 152) considered “*Valvata inflata* var. *curta* Tournouër” from Bresse, France identical to *Valvata interposita* de Stefani, 1880.

+ *Pachystoma involutum* Tausch, 1886

Original source: Tausch 1886: 14, pl. 2: fig. 9.

Type horizon: Upper Cretaceous, “Gosaumergel”.

Type locality: Csinger valley near Ajka, Bakony, Hungary.

Remarks: Bandel and Riedel (1994: 23, pl. 14: figs 4–7) figured the shell and protoconch by SEM.

+ *Cincinna (Atropidina) cobalcescui ismailense* [sic: should be *ismailensis*, since *Cincinna* is feminine] Gozhik, 2007

Original source: Gozhik 2007: 80, pl. 72: fig. 5–6.

Type horizon: Upper Miocene, Maeotian.

Type locality: Izmajil Region, Ukraine.

*Cincinna iturupensis* Prozorova, 1898 (in Prozorova and Starobogatov 1998)

Original source: Prozorova and Starobogatov 1998: 61, 63: fig. 3D.

Type locality: Lake Dobroye, Iturup Island, Russia.

Holotype: Zoological Institute of the Russian Academy of Sciences, St. Petersburg, Nr. 1 in systematic catalogue under the name.

+ *Valvata jaccardi* Locard, 1893

Original source: Locard 1893a: 212–213, pl. 12: fig. 5.

Type horizon: Upper Miocene.

Type locality: Le Locle (Canton Neuchatel) and midi de Vicques (Canton Bern), both Switzerland.

+ *Cincinna (Cincinna) bugense jagorliticus* [sic: should be *bugensis jagorlitica*, since *Cincinna* is feminine] Gozhik, 2007

Original source: Gozhik 2007: 76, pl. 67: figs 1–3.

Type horizon: Upper Miocene – Maeotian.

Type locality: bay of Jagorlyk, district of Pridnestrowje, Moldawia.

+ *Borysthenia jalpuchense* [sic: should be *jalpuchensis*, since *Borysthenia* is feminine] Gozhik, 2002

Original source: Gozhik 2002: 48, pl. 2: figs 15–17. Also figured by Gozhik (2007: 77, pl. 66: fig. 4–5)

Type horizon: Miocene, Pontian.

Type locality: ?? (not seen).

*Valvata japonica* Martens, 1877

Original source: Martens 1877: 116–117.

Type locality: Lake Hakone, Japan.

Types not traced. Possibly in Museum für Naturkunde, Berlin (fide Dance 1986).

Remarks: Live movie at http://www.youtube.com/watch?v=Z-GvcF8kOco

*Valvata jelskii* Crosse, 1863

Original source: Crosse 1863: 382–383, pl. 13: fig. 3 (3 views).

Type locality: River Dnieper, near Kiev, Ukraina.

Holotype: Museum National d’Histoire Naturelle, Paris.

+ *Valvata (Cincinna) jiangsuensis* Yü, 1977 (in Yü and Wang 1977)

Original source: Yü and Wang 1977: 14, pl. 1: figs 15–16.

Type horizon: Upper Cretaceous.

Type locality: Jiangsu Province, China.

+ *Amplovalvata jingguensis* Pan, 1977

Original source: Pan 1977: 118, pl. 5: fig. 18.

Type horizon: Middle Jurassic.

Type locality: Jinggu County, Yunnan Province, China.

+ *Valvata (Cincinna) joncheryensis* Wenz, 1930

Original source: Wenz 1930: 65.

Type horizon: Upper Pliocene.

Type locality (according to Deshayes, 1862): Jouchery, Gueux, Rilly, Basin de Paris, France.

Remarks: Replacement name for *Valvata parvula* Deshayes, 1862 (not Meek & Hayden, 1856).

“*Valvata judaica*“ mentioned in Innes (1884: 348) (nomen nudum)

Remarks: Innes (1884) referred to “Bourg., Spec. Moll. n^o^. 192, 1878”. However, as outlined by Connolly (1934), the latter work was never published.

+ *Valvata juliae* Scholz & Glaubrecht, 2010

Original source: Scholz and Glaubrecht 2010: 997 ff, textfigs 2–3.

Type horizon: Pliocene, Koobi Fora Formation.

Type locality: Turkana Basin, Northern Kenya.

+ *Valvata (Cincinna) circinata* var. *jurana* Jodot, 1954

Original source: Jodot 1954: 538, 548, pl. 23b, figs 5a,b, 6a,b.

Type horizon: Ludién, Eocene.

Type locality: 9 km north of Lélex, Département Ain, France.

+ *Valvata jurassica* Branson, 1935

Original source: Branson 1935: 519, pl. 57: figs 7–8.

Type horizon: Jurassic, Morrison Formation.

Type locality: 3 miles south of Mayoworth, Wyoming, USA.

+ *Pentagoniostoma jurassicum* Branson, 1935

Original source: Branson 1935, 520, pl. 57: figs 4–6. Classified as *Tropidina jurassicum* (Branson) in Yen (1952: 40).

Type horizon: Jurassic, Morrisson Formation.

Type locality: 3 miles south of Mayoworth, Wyoming, USA.

+ *Aphanotylus jurassicus* Pan, 1980 (in Yü and Pan 1980)

Original source: Yü and Pan 1980: 150, figs ?? (not seen, listed in Zoological Record 1980(A2): #3883).

Type horizon: Middle Jurassic.

Type locality: Zhejiang, Southern Anhui, China.

+ *Valvata (Borysthenia) juxi* Schlickum & Strauch, 1979

Original source: Schlickum and Strauch 1979: 13, pl. 1: fig. 7.

Type horizon: Pliocene - brown coal area.

Type locality: Bergheim (abandoned opencast mine Fortuna-Garsdorf), Nordrhein-Westfalen, Germany.

+ *Valvata kamirensis* Willmann, 1981

Original source: Willmann 1981: 114–115, textfig. 42A–B, 43A–C.

Type horizon: Pliocene, Salakos Formation.

Type locality: 2 km west of Kamiros ruins, Island of Rhodos, Greece.

Remarks: Note the high polymorphism of this species.

*Cincinna kamchatica* Prozorova & Starobogatov, 1998

Original source: Prozorova and Starobogatov 1998: 63, 65: fig. 3A.

Type locality: Eastern Kamchatka, small pond on Bolshoi Kamchatskyi Island in the valley of Kamchatka River, Russia.

Holotype: Zoological Institute of the Russian Academy of Sciences, St. Petersburg.

+ *Valvata (Mesovalvata) karameilica* Wei, 1984

Original source: Xinjiang Dizhi Ju 1984, 84, figs ?? (not seen, title in Zoological Record 126(9): #3985).

Type horizon: ??

Type locality: Xinjiang Province, China.

+ *Valvata kavusani* Schütt & Kavusan, 1984

Original source: Schütt and Kavusan 1984: 220, textfig.

Type horizon: Upper Miocene.

Type locality: top of the pass Kozluca, between Bali and Harmancık near Kütahya – Bursa in northwestern Anatolia, Turkey.

*Valvata khedivialis* Innes, 1884

Original source: Innes 1884: 348.

Type localities: “Bords du lac Timsah, dans l’isthme de Suez; sur les rives du lac Moeris, au Fayoun, et sur celles du lac Mariout, près Alexandrie”; all Egypt.

*Cincinna kizakikoensis* Fujita & Habe, 1991

Original source: Fujita and Habe 1991: 25 (English 26), figs 1–2.

Type locality: Lake Kizaki (also found in Lake Nakazuna), Nagano Prefecture, Honshu, Japan.

Holotype and paratypes deposited at National Science Museum Tokyo (NSMT–Mo 69606).

*Valvata (Costovalvata) klemmi* Schütt, 1962

Original source: Schütt 1962: 158–159: fig. 1 (shell): fig. 8 (radula). SEM photos of shell and radula provided by Falniowiski et al. (2007: specimens from the type locality cited as *Valvata piscinalis*).

Type locality: South border of Lake Trigonis/Trichonida near Agrinio (Bodina), Aetolia, Greece.

Holotype: Senckenberg Museum Frankfurt/Main #166762.

Remarks: Hauswald et al. (2008) showed that this taxon is a reaction form (ecomorph) of their *Valvata* “sp1.”, a species lacking the characteristic ridges of the shell and resembling *Valvata piscinalis*, whereas Welter-Schultes (2012a: 43; no citation of Hauswald et al. 2008 in this paper) considered a valid species. It appears possible that *Valvata klemmi* is a polymorphic species.

“*Valvata (Atropidina) klinensis* Milaschewitch, 1881“ mentioned in Anistratenko and Anistratenko (2001: 145) and Gozhik (2007: 77).

Misspelling of *Valvata fluviatilis* var. *kliniensis* Milachevich, 1881.

*Valvata fluviatilis* var. *kliniensis* Milachevich, 1881

Original source: Milachevich 1881: 236 (in issue of journal, page 22 in reprint).

Figured by Gozhik (2007: pl. 68: figs 5–7).

Type locality: “Moujevo” (vicinities of Moscow), Russia.

Syntypes: Zoological Institute of the Russian Academy of Sciences, St. Petersburg.

*Cincinna (Sibirovalvata) klucharevae* Starobogatov, 1985

Original source: Starobogatov and Zatravkin 1985: 1158, fig. 5.

Type locality: Southern Sakhalin Island, near Bousset lagoon, Bolshoj Vavaj Lake, depth 2.4 m, between Kamschatka Peninsula and Japan, Russia.

Holotype: Zoological Institute of the Russian Academy of Sciences, St. Petersburg, Nr. 1 in systematic catalogue under the name.

+ *Valvata (Tropidina) kochi* Pavlović, 1932

Original source: Pavlović 1932: 245, pl. 1: figs 14–16.

Type horizon: Pliocene−Pleistocene, Metohija Series.

Type locality: Topličane (“blue marne Levantines near Topelić”), Metohija basin, Kosovo.

Also figured by Miloshevich (1984: pl. 1: figs 48–53).

*Valvata (Cincinna) korotnevi* Lindholm, 1909

Original source: Lindholm 1909: 73, pl. 1: figs 63–65.

Type locality: Angarskyi sor (northern Baikal), Russia.

Lectotype designated by Starobogatov and Streletzkaya (1967), deposited at Zoological Institute of the Russian Academy of Sciences, St. Petersburg, Nr. 1 in systematic catalogue under the name.

*Megalovalvata kozhovi* Sitnikova, 1983

Original source: Sitnikova 1983: 35–37: fig. 2.

Type locality: Zavorotnaya Bay (western coast of northern Baikal), Russia.

Holotype at Zoological Institut of the Russian Academy of Sciences, St. Petersburg, Nr. 1 in systematic catalogue under the name.

*Valvata kugleri* Forcart, 1948

Original source: Forcart 1948: 50.

Holotype and paratypes in Naturmuseum Basel, Switzerland #2631.

www.nmb.bs.ch/typenkatalog_mollusken_internetversion_korrekturen.xls

Type locality: Pota Juela, Cumarebo, Falcon, Venezuela

Remarks: Currently (GNI) considered as *Nanivitrea kugleri* (Forcart, 1948) cf. Nuttall (1989: 175, 213) (**Hydrobiidae** - Cochliopinae), or as *Tudora (Tudoata) williamsoni kugleri* (Forcart, 1948) (**Annulariidae**).

*Valvata kukunorica* Sturany, 1900

Original source: Sturany 1900: 39 (issue of journal, page 23 in reprint), pl. 3: figs 7–9.

Type locality: Lake Kuku–nor (today Qinghai Lake) in Nan-Shan mountains, Tibet.

“*Valvata kunkunorica*” (GNI)

Misspelling of *Valvata kukunorica* Sturany, 1900 (not listed in GNI).

+ *Valvata kupensis* Fuchs, 1870

Original source: Fuchs 1870b: 543, pl. 22: figs 23–25.

Type horizon: Upper Miocene, Upper Pannonian.

Type locality: near Kúp, Pápa, Hungary.

*Valvata lacustris* Clessin, 1877

Original source: Clessin 1877: 177.

Type locality: Lake Geneva, 50–100 m, Switzerland.

Remarks: According to Clessin (1877) a deep water morph being derived from *Valvata piscinalis antiqua* Morris, 1838.

*Valvata (Cincinna) piscinalis* var. *ladogaensis* Lindholm, 1912

Original source: Lindholm 1912: 289 (name only), 298 (description).

Type locality: Lake Ladoga (1) entry of river Kabona, between the channels; (2) Bay of Wolchow (vis–à-vis of entry of river Sjäss), northwestern Russia.

“*Valvata piscinalis* var. *ladogensis* Lindholm, 1912“ mentioned in Anistratenko and Anistratenko (2001: 139, 142).

Misspelling of *Valvata (Cincinna) piscinalis* var. *ladogaensis* Lindholm, 1912.

*Valvata (Pseudomegalovalvata) laethmophila* Bekman & Starobogatov, 1975

Original source: Bekman and Starobogatov 1975: 95: fig. 1D.

Type locality: near Listvennichnoe, Baikal Lake, depth 1380 m, Russia.

Holotype: Zoological Institute of the Russian Academy of Sciences, St. Petersburg, Nr. 1 in systematic catalogue under the name.

+ *Microcyclas lamellosus* Raspail, 1909

Original source: Raspail 1909: 197, pl. 4: figs 27–29.

Type horizon: Eocene.

Type locality: Vouast, près Montjavoult, Dept. Oise, France.

Remarks: Type species (by monotypy) of genus *Microcyclas* Raspail, 1909.

+ *Valvata landereri* Hermite, 1879

Original source: Hermite 1879: 184, 190, 199 (name only), 320 (description), pl. 5: figs 21–22.

Type horizon: Lower Eocene.

Type locality: Sineu, Mallorca, Spain.

Remarks: According to the (not round) aperture probably not a valvatid.

+ *Valvata larteti* Bourguignat, 1881

Original source: Bourguignat 1881: 153, pl. 8: figs 297–299.

Type horizon: Middle Miocene - calcaire marneux.

Type locality: colline de Sansan, west of Toulouse, France.

Remarks: with 9 mm shell size probably no *Valvata*.

*Valvata (Cincinna) andreana* var. *latior* Menzel, 1904

Original source: Menzel 1904a: 77–78, textfig. Cited as *Valvata andreaei* var. *latior* in Menzel (1904c: 287, pl. 14: figs 11–14, 25, 27–28, 32–34, 37–38, 40).

Type horizon: post–glacial.

Type locality: Wallensen near Salzhemmendorf, Kreis Hameln, Niedersachsen; and Alfeld / Leine, Niedersachsen, Germany.

Remarks: Based on fossil types, but considered as extant by Starobogatov et al. (2004) and Kantor et al. (2011) occurring in the lakes of the basin of the Baltic Sea.

“*Valvata piscinalis* var. *latius umbilicata*“ or „*late umbilicata*“ mentioned in Schmidt (1856: 160) (nomen nudum, presented in two spellings without description or indication, only with locality).

Locality: Ebenthaler Allee (avenue) near Klagenfurt, Carinthia, Austria.

Remarks: Note the similar (available) name *Valvata umbilicata* Servain, 1881.

*Valvata (Atropidina) lauta* Lindholm, 1909

Original source: Lindholm 1909: 74–75, pl. 1: figs 68–70. Attributed to Milaschewitsch, but ICZN Art. 50.1.1 is not satisfied.

Type localities: Tschiwyrkuikji Saliw; opposite to entry of river Angara (Dagarskaja Guba); bay of Besimennaja, 10 Werst (10,6 km) from village Gorjatschinskoje, all Lake Baikal, Russia.

Lectotype designated by Sitnikova et al. (1983) and deposited at Zoological Institut of the Russian Academy of Sciences, St. Petersburg; Nr. 17.

+ *Valvata leduensis* Li, 1988

Original source: Li 1988: 157, pl. 1: figs 1–12.

Type horizon: Upper Cretaceous, Minhe Formation.

Type locality: Xining-Minhe Basin of Qinghai, China.

+ *Valvata leei* Logan, 1900

Original source: Logan 1900: 133, pl. 31: figs 1–3. Cited and figured as + *Amplo-valvata scabrida leei* (Logan) in Yen (1952: 40, pl. 6: figs 3a–c).

Type horizon: Jurassic, Morrison Formation.

Type locality: Freeze–out Hills, Wyoming, USA.

*Valvata lenticularis* Küster, 1856.

Original source: Küster 1856: 78.

Type locality: Sediment of river Regnitz near Bamberg, Germany.

+ *Valvata leopoldi* Boissy, 1848

Original source: Boissy 1848: 284, pl. 6: fig. 25a,b (as a nomen nudum in Boissy 1846: 178).

Type horizon: Lower Paleocene, Thanetian.

Type locality: Calcaire de Rilly–la–Montagne: near Reims, France.

+ *Valvata leptonema* Brusina, 1892

Original source: Brusina 1892: 167–168 (in issue of journal, 55–56 in reprint). Figured in Brusina (1902: pl. 14: figs 5–7).

Type horizon: Upper Miocene, Pannonian.

Type locality: Markuševec (Zagreb), Croatia.

+ *Valvata leptopomoides* Reuss, 1868

Original source: Reuss 1868: 83, pl. 1: fig. 4.

Type horizon: Lower Miocene, Eggenburgian.

Type locality: freshwater limestone of Tuchořice, Bohemia, Czech Republic.

Remarks: currently *Craspedompoma leptopomoides* (Reuss, 1868) (**Pomatiasidae**).

+ *Valvata lessonae* Sacco, 1886

Original source: Sacco 1886: 177, pl. 1: figs 8a,b,c.

Type horizon: Upper Pliocene.

Type locality: Fossano, Piemont, Italy.

“*Valvata letourneuxi*“ mentioned in Innes (1884: 349) (nomen nudum with localities)

Remarks: Innes (1884: 349) refers to “Bourguignat, Spec. Moll. n^o^. 194, 1878”.

However, as outlined by Connolly (1934), the latter works was never published. Innes (1884) listed two localities in Egypt, but did not provide a description of the species.

+ *Valvata (Tropidina) levantica* Halaváts, 1889

Original source: Halaváts 1889: 228, pl. 34: fig. 6a,b.

Type horizon: Pliocene–Lower Pleistocene.

Type locality: “Nagy András János” - fountain (artesic) in Hódmezővásárhely, Hungary.

Remarks: The large size (9 mm high, 10 mm wide) and the shell shape both make a valvatid nature unlikely.

*Valvata lewisi* Currier, 1868

Original source: Currier 1868: 9.

Type locality: Grattam, Kent (Michigan), USA.

Types: not traced.

Remarks: Vernacular name “fringed valvata”. Replacment name for *Valvata striata* Lewis, 1856, not Philippi, 1836 (p. 147, pl. 8: fig. 3a–c; **Tornidae**). It is improbable that this American species is identical with the Siberian ones reported by Starobogatov et al. (2004) (cf. Kantor et al. 2011: 69). For reproduction see Lang and Dronen (1970).

Radula (rhachis tooth) figured by Baker (1928: 28).

+ *Valvata (Atropidina) liaoxiensis* Yü, 1987

Original source: Yü X. 1987: 51, figs ?? (not seen, listed in Zoological Record 134(9): #4045).

Type horizon: Lower Cretaceous.

Type locality: Western Liaoning Province, China.

*Valvata lietuvensis* Chernogorenko & Starobogatov, 1987

Original source: Chernogorenko and Starobogatov 1987: 149.

Type locality: Vyshtitis Lake, at the border of Kaliningrad district and Lithuania. Holotype: Zoological Institut, Russian Academy of Sciences, St. Petersburg, Nr. 1 in systematic catalogue under the name.

*Valvata lilljeborgi* Westerlund, 1897

Original source: Westerlund 1897a: 137.

Type locality: “Suecia in fluvio Fyrisån ad Upsala”, Sweden.

Syntypes in Naturalhistoriska Museet Göteborg (AN 4654) (Kantor et al. 2011: 69) figured by Vinarski et al. (2013b: figs 4A–B) and Zoological Institut, Russian Academy of Sciences, St. Petersburg, Nr. 1 in systematic catalogue under the name.

“*Valvata limpida*” (GNI, ION)

Possibly an error for *Vitrina limpida* Gould, 1850.

+ *Valvata (Cincinna) lorentheyi* Wenz, 1928

Original source: Wenz 1928a: 120 (as *lörentheyi*, dedicated to the Hungarian Emerich Lörenthey; cf. ICZN Art. 32.5.2.1).

Type horizon: Upper Miocene, Pannonian.

Type locality: Szegzárd, Kom. Tolna, Hungary.

Remarks: According to Wenz (1928b: 2438) a replacement name for *Vivipara unicarinata* Lörenthey, 1894, non *Valvata unicarinata* Lörenthey, 1894.

+ *Valvata loryana* Loriol, 1865

Original source: de Loriol and Jaccard 1865: 33, pl. 2: fig. 20.

Type horizon: Jurassic / Lower Cretaceous.

Type locality: Villers-Le-Lac, northwest of Neuchâtel, France.

Remarks: Designated by monotypy as type species of valvatid genus *Loriolina* Huckriede, 1967.

+ *Valvata lucici* Brusina, 1902

Original source: Brusina 1902: pl. 13: figs 31–32.

Type horizon: Pliocene-Pleistocene, Dacian-Romanian.

Type locality: Čerević (Fruška Gora), Serbia.

*Paludina lustrica* Say, 1821

Original source: Say 1821: 175.

Type locality: Shore of Cayuga Lake, New York, U.S.A.

Remarks: (1) Listed as “*Valvata lustrica* m. (*Padulina lustrica* Say)” by Menke (1830: 46). Menke (1845: 130) stated that his *Valvata lustrica* is identical to *Paludina lustrica* Say, lacks the typical valvatid gill and should be placed in **Amnicolidae** or **Hydrobiidae**.

(2) Pilsbry (1943) designated *Paludina lustrica* Say, 1821 as type species of *Euamnicola* Crosse & Fisher, 1891: 262 (Kabat and Hershler 1993: 22), the latter is now the objective synonmy of *Amnicola* Gould & Haldeman, 1840 (see ICZN 1973: Opinion 1108).

(3) Interestingly, Hagen (1864: 130) and Lindholm (1906: 193) both considered this taxon as the larval shell of a **trichopteran insect** (*Hydropsyche*).

“*Valvata maackei* Gerstf.“ mentioned in Westerlund (1877: 109).

As stated by Dybowski (1886: 108, footnote) and Lindholm (1909: 79) Gerstfeldt (1859) did not describe this species. Westerlund (1877) probably confused the taxon with *Choanomphalus maackei* Gerstfeld, 1859 (**Planorbidae**).

*Valvata macei* Locard, 1884

Original source: Locard 1884: 208 (attributed to Bourguignat, but ICZN Art. 50.1.1 is not satisfied).

Type locality: Saint-Martin de Varreville (Departement de la Manche), France.

*Valvata macrostoma* Mörch, 1864

Original source: Mörch 1864: 321 (diagnosis) (attributed to Steenbuch, but ICZN Art. 50.1.1 is not satisfied).

Type locality: “Talrig i en Mosegröft paa sognefogdens Lod i Rudi (Stb.); Sorösö [Sorø] (Stp.); Viborgsö [Viborg] (Fedd.)” Midtjylland, Danmark.

Types: According to Dance (1986), Mörch’s collection is dispersed between Zoological Museum Copenhagen and British Museum of Natural History (London).

Remarks: “*Valvata macrostoma* Steenbuch” in Beck (1847: 123) is a nomen nudum.

For life cycle see Myzyk (2004). Live photos at: http://www.allesumdieschneck.de/html/valvata_macrostoma.html

+ *Amplovalvata magna* Pan, 1980

Original source: Yü and Pan 1980: 149, figs ?? (not seen, listed in Zoological Record 1980(A2): #3883).

Type horizon: Middle Jurassic.

Type locality: Zhejiang, Southern Anhui, China

Holotype: http://159.226.74.248:8000/viewSpeciDetailsNormal.jsp?bbbh=36293

+ *Valvata magniumbilicata* Youluo, 1978 (name of author according to the type catalogues of Nanjing Institute)

Original source: Youluo 1978: 29, pl. 3: figs 1–3.

Type horizon: Lower Tertiary.

Type locality: coastal region of Bohai, China.

“*Valvata alpestris* var. *major*” in Favre (1922: 50) (nomen nudum with locality)

Locality: Lac de Joux, Kanton Waadt, Switzerland.

“*Valvata major* Gredl.” http://zipcodezoo.com/Animals/V/Valvata_major/ (also GNI).

There is a *Valvata piscinalis* var. *minor* in Gredler (1859: 250), but not a var. *major*. Westerlund (1886: 132f) mentioned a “forma *major*” for each of several *Valvata* species without any diagnosis except the size.

+ *Amplovalvata manasensis* Zhu, 1994

Original source: Zhu 1994: 94 (Chinese) / 102–103 (English), pl. 4: figs 9–14.

Type horizon: Middle Jurassic.

Type locality: Ziniquanzi, Janggar Basin, Northern Xinjang, China.

+ *Borysthenia mankeanaformis* Gozhik, 2007

Original source: Gozhik 2007: 75, pl. 65: fig. 1. Expressivly meant as “similar to *Borysthenia mankeana* [sic]” (i.e. *Borysthenia menkeana* (Jelski, 1863)).

Type horizon: Pleistocene (Alluvium VIII. Terrace).

Type locality: river Dnjestr near village Velikaya Kosnitza, Ukraine.

+ *Amplovalvata mansueta* Pan, 1982

Original source: Pan 1982: ??, figs 3–4 (not seen, not listed in Zoological Record).

Type horizon: Jurassic.

Type locality: Artesian springs, Sichuan Jiangyou County Kang, Sichuan Basin, China.

Holotype: http://159.226.74.248:8000/viewSpeciDetailsNormal.jsp?bbbh=53438

+ *Valvata marginata* Michaud, 1855

Original source: Michaud 1855: 50 (in issue of journal, 18 in reprint), pl. 5: figs 16–18.

Type horizon: Lower Pliocene, Zanclean («Plaisancien»).

Type locality: Hauterive, Département de Drôme, France.

Remarks: Type species of *Pachystoma* Sandberger, 1875 and of *Oncostoma* Brusina, 1894 (see there).

“*Valvata mariae*“ mentioned in Siodiropoulou (2003: 38–39, fig. 14).

Locality: Pliocene - Pleistocene, Ptolemaida, West-Macedonia, Greece.

Remarks: This PhD–Thesis, in which 35 species are ostendibley described, does not meet the conditions of ICZN Art. 8.1.3 and 9.9. Accordingly, this name is not available.

“*Valvata margine columellari*” (GNI)

Probably misinterpreted from a Latin description, not a binominal name.

“*Valvata marmorata*”, “*Valvata marmorea*”, “*Valvata marmoreus*” (GNI, ION)

Probably all errors for *Cochlea mormorea* Swammerdam, 1737 (p. 185, pl. 10: fig. 2), according to Martini (1767: 104) and Müller (1774: 194) a synonym of *Nerita fluviatilis* (currently *Theodoxus fluviatilis* (Linné, 1758), Neritidae). Note that *Nerita piscinalis* Müller, 1774 is currently *Valvata piscinalis*.

*Valvata maroccana* Pallary, 1904

Original source: Pallary 1904: 41, pl. 3, figs 6–7.

Type locality: Morocco, north–west Africa.

Remarks: According to the figures with certainty not a *Valvata*, but I could not find any subsequent determination of current systematic position of this species.

+ *Valvata lewisi mccolli* LaRocque, 1932

Original source: LaRocque 1932: 199, pl. 1: figs 1a–3c.

Type horizon: Pleistocene - marl of Upper Wisconsin age.

Type locality: Shallow Lake, Ontoario, USA.

“*Valvata media*“ mentioned in Férussac and Férussac (1807: 128/129, no. 167) (nomen nudum)

*Valvata tricarinata mediocarinata* Baker, 1928

Original source: Baker 1928: 17, pl. 1: fig. 7.

Type locality: Lower Asylum Bay, Lake Winnebago, Wisconsin, USA.

*Valvata menkeana* Jelski, 1863

Original source: Jelski 1863: 136–137, pl. 6: fig. 4.

Type locality: “Profonds du Dnieper”, Ukraina.

Lectotype figured in Anistratenko and Anistratenko (2001: 133, fig. 96), deposited at Zoological Institute of the Russian Academy of Sciences, St. Petersburg, Nr. 2. Paralectotypes in Museum National d’Histore Naturelle, Paris.

Remarks: Currently (e.g. Kantor et al. 2011: 74) considered as belonging to genus *Borysthenia*. Radula and parts of genital system are figured by Sitnikova et al. (1986: figs 1, 2); SEM of protoconch by Anistratenko et al. (2010).

*Valvata meretricis* Locard, 1889

Original source: Locard 1889: 26 (attributed to Bourguignat, but ICZN Art. 50.1.1 is not met).

Type localities: (1) Marais du Boucau near Bayonne, Pyrénées-Atlantiques, (2) “lac de la Négresse” near Bayonne, Pyrénées-Atlantiques, (3) “Moulins”, Allier, France.

*Valvata meridionalis* Locard, 1889

Original source: Locard 1889: 59 (attributed to Bourguignat, but ICZN Art. 50.1.1 is not met).

Type locality: Viareggio, Toscana, Italy.

*Valvata mergella* Westerlund, 1883

Original source: Westerlund 1883: 166; also figured by Westerlund (1885: 209, pl. 5: figs 22, a–d).

Type locality: Port Clarence, Alaska, U.S.A.

Syntypes (16 specimens) located in Swedish Museum of Natural History (AN 1640), figured by Johannes (2010).

Remarks: Vernacular name “rams–horn valvata”. This taxon is also listed by Kantor et al. (2010) for Russia, but it is uncertain, whether the American and the Russian specimens are conspecific.

+ *Valvata menyinensis* Yen, 1969

Original source: Yen 1969: 42, pl. 1: figs 10–11.

Type horizon: “Higher than Men-Ying of Lower Cretaceous”, Kuan-Chuang-Series.

Type locality: Meng-Yin–valley (Kuan-Chuang), Shantung, North China.

+ *Valvata michaudi* Deshayes, 1862

Original source: Deshayes 1861–1863, Vol. 2: 525, pl. 36: figs 6–8.

Type horizon: Middle Eocene.

Type locality: Caumont, Mareul–en-Dole, Basin de Paris, France.

*Valvata michleri* Kuščer, 1932

Original source: Kuščer 1932: 56–57, pl. 5: fig. 3.

Type locality: Ljubljanica spring, Mocilnik, Slovenia.

Remarks: According to Binder (1967a) synonymous to *Valvata minuta* Draparnaud, 1807 (see there; **Hydrobiidae**); according to Bodon et al. (2001: 124) a junior synonym to *Hauffenia tellini* Pollonera, 1898 (**Hydrobiidae**), whereas Callot-Girardi and Girardi (2013) considered two separate species.

*Valvata micra* Pilsbry & Ferriss, 1906

Original source: Pilsbry and Ferriss 1906: 172, pl. 9: figs 7–9.

Type locality: Guadeloup River, Texas, USA.

Syntypes at Academy of Natural Sciences of Drexel University, Philadelphia #91322. Syntypes from locality 6 are figured by Hershler and Longley (1986: 133; Fig. 3J, N).

Remarks: Pilsbry (1916) himself considered the species a hydrobiid (*Horatia*). Later on, *Valvata micra* was designated as type species of *Phreatodrobia* Hershler & Longley, 1986 (**Hydrobiidae**) by original designation, see also Nuttall (1989: 222), Kabat and Hershler (1993: 43).

*Valvata micrometrica* Locard, 1889

Original source: Locard 1889: 56–57.

Type Locality: «Fontaine du Camarde, près de Valence dans Gers», France.

Remarks: Esu and Girotti (1975: 235) classified *Valvata micrometrica* in *Horatia* (**Hydrobiidae**). Falkner and Boeters (2003: 21) designated a lectotype (now Museum National de l’Histoire Naturelle, Paris).

*Valvata microscopica* Nevill, 1877

Original source: Nevill 1877: 21.

Type locality: brackish–water pond near Port Canning, India.

Remarks: According to Abbott (1949: original description repeated) considered as *Clenchiella microscopica* (Nevill, 1877) (**Hydrobiidae**).

“*Valvata (Tropidina) microstoma*” mentioned in Kolářova et al. (2010)

Misspelling of *Valvata macrostoma* Mörch, 1864.

*Cincinna (Sibirovalvata) sibirica middendorffi* Starobogatov & Zatravkin, 1985

Original source: Starobogatov and Zatravkin 1985: 1158–1159: fig. 7 (attributed to Moskvichera, but ICZN Art. 50.1.1 is not satisfied).

Type locality: Amur river, Siberia, Russia.

Holotype: Zoological Institute of the Russian Academy of Sciences, St. Petersburg, Nr. 1 in systematic catalogue under the name.

“*Valvata miliaria* Ziegler“ mentioned in Frauenfeld (1863: 1027).

One of the many label names of Ziegler and Parreys (see above at *Valvata impura*) for *Amnicola miliaria* Parreys, today *Pseudoamnicola miliaria* (Frauenfeld, 1863) (**Amnicolidae**).

+ *Valvata minima* Hislop, 1859 (non Fuchs, 1877)

Original source: Hislop 1859: 170, pl. 5: fig. 13.

Type horizon: Tertiary.

Type locality: Little Tisti, Karwad, Butárá, East India.

+ *Valvata minima* Fuchs, 1877 (non Hislop, 1859)

Original source: Fuchs 1877: 14, pl. 1: figs 25–27.

Type horizon: Lower Pliocene.

Type locality: Megara, Greece.

Remarks: This species is a junior homonym of *Valvata minima* Hislop, 1859. Wenz (1928b: 2439) listed *Valvata serbica* Brusina, 1902 as a junior synonym, which may be the next available name for *Valvata minima* Fuchs, 1877.

“*Valvata alpestris* var. *minor*“ mentioned in Favre (1922: 50) (nomen nudum with locality)

Locality: Postglacial; Vallée de Joux, Kanton Waadt, Switzerland.

+ *Valvata piscinalis* var. *minor* De Stefani, 1877

Original source: De Stefani 1877: 305, pl. 18, fig. 4.

Type horizon: Pleistocene.

Type locality: Spoleto (Pananelli), Umbria, Italy.

*Valvata baicalensis* forma *minor* Lindholm, 1909

Original source: Lindholm 1909: 78.

Type locality: Lake Baikal, Russia.

Types unknown.

+ *Valvata minuscula* Yen & Reeside, 1946

Original source: Yen and Reeside 1946: 53: figs 1a–b.

Type horizon: Jurassic, Morrison Formation.

Type locality: Sublette County, Wyoming, USA.

Holotype: U.S. National Museum #103.799.

*Valvata minuta* Draparnaud, 1805 (non *Valvata (Atropidina) minuta* Yü, 1987)

Original source: Draparnaud 1805: 42, pl. 1: figs 36–38.

Type material: Naturhistorisches Museum Wien #1820/XXVI/21 7.33; cf. Binder (1967a), Bodon et al. (2001: 195), ICZN Opinion 2035 (2003).

Type locality: Source de l’Ain, France (cf. Bodon et al. 2001: 195).

Remarks: Detailed comment by Westerlund (1879). For shell variability cf. Binder (1967a). According to Binder (1967a), Bodon et al. (2000, 2001, ICZN Case 3146), and Callot-Girardi and Girardi (2013) a species of **Hydrobiidae** (*Hauffenia*, *Neohoratia*, *Islamia*).

+ *Valvata (Atropidina) minuta* Yü, 1987 (non *Valvata minuta* Draparnaud, 1805)

Original source: Yü XH 1987: 51, figs ?? (not seen, listed in Zoological Record 134(9): #4045).

Type horizon: Lower Cretaceous.

Type locality: Western Liaoning Province, China.

+ *Costovalvata minuta* Youluo, 1978 (name of author according to the type catalogues of Nanjing Institute)

Original source: Youluo 1978: 31, pl. 4: figs 18–20.

Type horizon: Lower Tertiary.

Type locality: coastal region of Bohai, China.

*Valvata minutissima* Wattebled, 1884

Original source: Wattebled 1884: 131, pl. 6, fig. 8.

Type locality: “L’arroyo de Long-Xuyen”, Mekong Delta, Vietnam.

“*Valvata mischleri* Kuščer, 1933“ mentioned in Binder (1967a: 375).

Misspelling of *Valvata michleri* Kuščer, 1932.

+ *Valvata moesiensis* Jekelius, 1944

Original source: Jekelius 1944: 55, pl. 7: fig. 11–15.

Type horizon: Middle Miocene, Sarmatian.

Type locality: Soceni (Banat), Romania.

Remarks: According to Bandel (2010: 94) this species should be placed in the heterobranch family **Cornirostridae** (also Valvatoidea/Ectobranchia) because of the slightly hyperstrophic protoconch.

+ *Amnicola moguntina* Boettger, 1884

Original source: Boettger 1884: 276. Considered a *Valvata* by Cossmann (1921: 110), cited and figured as + *Valvata moguntina* (Boettger, 1884) by Kókay (2006: 31, pl. 3: figs 6–7).

Type horizon: Lower Miocene, Burgidalian.

Type locality: Niederrad (part of Frankfurt/Main), Hessen, Germany.

+ *Valvata (Cincinna) molnarae* Soós, 1955

Original source: Bartha and Soós 1955: 58, pl. 5: figs 5–7.

Type horizon: Upper Miocene – Lower Pliocene.

Type locality: Kocs (Szendi-Street), Hungary.

+ *Valvata monachorum* Bukowski, 1895

Original source: Bukowski 1895: 24 (name only), 31–33 (description), pl. 8: fig. 12.

Type horizon: Upper Miocene.

Type locality: marl and/or chalk near Monastery Skhiadi, Rhodus Island, Greece.

Remarks: Lectotype figured by Willmann (1981: 75, fig. 24A).

“*Valvata mongazoniana* Servain” (nomen nudum)

Remarks: Mentioned in Hagenmüller (1884: 216 “cette coquille est inedité”) and Westerlund (1886: 143), but never formally described.

+ *Valvata montanaensis* Meek, 1876

Original source: Meek 1876: 591, textfigs 81–83.

Type horizon: Latest Cretaceous.

Type locality: mouth of Judith River on Upper Missouri, Montana, USA.

*Valvata montenegrina* Glöer & Pesic, 2008

Original source: Glöer and Pesic 2008: 346–347: fig. 2/1–8) (shell, operculum, soft body).

Types: Holotype and 3 paratpyes in the Museum für Naturkunde, Berlin (#37584), paratypes also in private collection of P. Glöer.

*Valvata monterosati* Westerlund, 1883

Original source: Westerlund 1883: 170 (attributed to Cafici, but ICZN Art 50.1.1 is not satisfied).

Type locality: Sicily, Italy.

“*Valvata moquini*” mentioned in Germain 1931 (fig. 745; p. 676)

Misspelling of *Valvata moquiniana* Dupuy, 1851 (no reasoning for a replacement name).

*Valvata moquiniana* Dupuy, 1851

Original source: Dupuy 1851: 586–587, pl. 28: fig. 15. The name was not attributed to Reyniés in the headline, only in a note in the synonymy. Description was also at least in parts by Dupuy. In footnote 2 Dupuy stated that Reyniés had provided a description, so maybe the Latin and French description could have been based on Reynies’s notes. However in the past paragraph Dupuy compared the species with others, this was also part of the description and clearly written by Dupuy. Since Reyniés was not “alone” responsible for both, the name and the description, the authorship for the new name must be attributed to Dupuy alone under ICZN Art. 50.1.1.

Type locality: “les alluvions du Lot, près de Mende, Département de Lozère, France.

Remarks: (1) Kobelt (1883: 20) stated that “*Valvata moquiniana* Reyn., abgebildet bei Moq. Tandon t. 41 f. 29–31, ist nach Fagot et de Malafosse (1878 Catalogue des Mollusques terrestres et fluviatiles vivants observés dans le département de la Lozère, p. 28) eine sehr verdächtige Art, nach einem Exemplar beschrieben - und seitdem nicht wieder gefunden worden” [*Valvata moquiniana* Reyn., depicted by Moq. Tandon t. 41 f. 29–31, is according to Fagot et de Malafosse (1878 Catalogue des Mollusques terrestres et fluviatiles vivants observés dans le département de la Lozère, p. 28) a suspicious species, based on a single specimen - and since then never found again].

(2) Bodon et al. (2001: 202) suspected and Falkner and Boeters (2003: 21) considered the taxon as a synonym of *Valvata globulina* Férussac, 1807 (but see there) and stated that syntypes are not available and that a re–description of *Valvata moquiana* and the designation of a neotype is necessary to clear up this often cited stygobiont taxon.

(3) Type species of *Globuliana* Paladilhe, 1866 (**Hydrobiidae**)by subsequent designation under ICZN Art. 70.3. by Kadolsky (2008: 116).

“*Amplovalvata morrisonensis*“ mentioned in Yen (1952: 27) (nomen nudum)

*Valvata mucronata* Menke, 1830

Original source: Menke 1830: 46 (nomen nudum), 139 (description).

Type locality: Island of Madeira, Portugal.

Remarks: Erroneously attributed to “Menke, 1845” at animal base www.animalbase.uni-goettingen.de/zooweb/servlet/AnimalBase/home/speciestaxon?id=15908

*Valvata muelleri* Leach, 1852

Original source: Leach 1852: 205–206 (original spelling *mülleri*; cf. ICZN Art. 32.5.2.1).

Type localities: “common in ponds around London and Bristol… some of the pond near Edinbough” and many others throughout Europe based on the synonymy list. Specification requires type selection.

Remarks: According to the original description “diameter 3/16 of an inch [= 4.7 mm]; animal black, tentacles, lateral appendages and lobes of the foot pale bluish–black, terminating with hyaline, eyes very black”, probably *Bithynia tentaculata* (Linnaeus, 1758) (**Bithyniidae**).

+ *Valvata multicarinata* Hislop, 1859 (non Yen, 1946)

Original source: Hislop 1859: 170, pl. 5: figs 15a–b.

Type horizon: Tertiary.

Type locality: Little Tisti, Karwad, Butárá, East India.

Remarks: The valvatid nature of this taxon was doubted by Hrubesch (1965: 98).

+ *Valvata multicarinata* Yen, 1946 (non Hislop, 1859)

Original source: Yen 1946: 487–488, pl. 76: fig. 1.

Type horizon: Pliocene.

Type locality: Honey Lake, Lassen County, California, USA.

Remarks: A junior homonym of *Valvata multicarinata* Hislop, 1859, thus replaced by *Valvata idahoensis* Taylor, 1981 (see there).

+ *Liratina multicarinata* Yü, 1974

Original source: Yü 1974: 373, pl. 198: figs 7–9.

Type horizon: Lower Jurassic.

Type locality: Jiangyou, Sichuan Province, southwest China.

+ *Paludina multiformis* Zieten, 1830

Original source: Zieten 1830: 40, pl. 30: figs 7–10. Attributed to Bronn, but ICZN Art. 50.1.1 is not satisfied.

Type horizon: Middle Miocene.

Type locality: Steinheim Basin; Baden-Württemberg, Germany.

Remarks: (1) + *Valvata multiformis* according to Ludwig v. Buch (1837: 98). Currently regarded as synonym to *Gyraulus trochiformis* (Stahl, 1824) (**Planorbidae**). This is the famous species, on which Hilgendorf’s (1863, 1866) descendence theory was established (e.g. Nützel and Bandel 1993; Glaubrecht 2012).

(2) Nomenclatural situation unclear: The name was made available (ICZN Art. 12.2.7, Glossary “taxon: a taxon encompasses al included taxa of lower rank”), but not based on types (Art 72.4.1), so without identity. Same situation as in *Helix draparnaldi* (ICZN Opinion 336 and 1924, name was regarded as available) and *Helix barbata* (Op. 1691, name was not regarded as available). It is recommended that this gap should be closed in the next edition of the Code, preferably in a sense that ICZN Art. 74.2.1 will be amended to rule that such names should be based on all types of the included subordinate variants.

*Helix nana* Megerle von Mühlfeld, 1824 (non *Helix nana* Pennant, 1777 from Britain; non *Helix spiriplana* var. *nana* Mousson, 1861: 125)

Original source: Megerle von Mühlfeld 1824: 220, pl. 8 (resp. 2 in reprint): figs 10a–b. “.... ebenfalls zur Gattung *Valvata* gehörige Schnecke” [likewise a snail belong to the genus *Valvata*], meant in the sense of a subgenus of *Helix*.

Type locality: not provided.

Remarks: According to figures identical to *Helix tricarinata* Megerle von Mühlfeld, 1824 (see there) and probably a synonym to *Valvata cristata*.

+ *Valvata nana* Meek, 1873 (non Westerlund, 1886; non Li, 1984)

Original source: Meek 1873: 507–508.

Type horizon: Cretaceous.

Type locality: Carleton’s coal mine, Coalville, Utah, USA.

*Valvata nana* Westerlund, 1886 (non Meek, 1873, non Li, 1984) (under subgenus *Tropidina*)

Original source: Westerlund 1886: 141.

Type locality: Zealand, Denmark.

Syntype (1 specimen) in Naturalhistoriska Museet Göteborg, Sweden (AN 4679), and (1 specimen) in Swedish Museum of Natural History (AN 14: 96) figured by Vinarski et al. (2013b: fig. 4C).

Remarks: A junior homonym of *Valvata nana* Meek, 1873.

+ *Valvata nana* Li, 1984 (non Meek, 1873; non Westerlund, 1886)

Original source: Li 1984: 7, pl. 1: figs 23–28.

Type locality: Lower Tertiary; Lingboa Basin of Henan Province, China.

Remarks: A junior homonym of *Valvata nana* Meek, 1873.

*Valvata naticina* Menke, 1845

Original source: Menke 1845: 129.

Type locality: “Hungaria, ad Pestinum” [Danube at Budapest], Hungary.

Types: Menke’s collection was dispersed after his death (Dance 1986).

Remarks: Currently considered as *Borysthenia naticina* (Menke, 1845). For anatomy, histology, and reproduction biology see Niero and Bodon (2011) and Hawe et al. (2013).

+ *Protovalvata naticiformis* Pană, 2000

Original source: Pană 2000: 89, pl. 5: figs 22–29.

Type horizon: Lower Creatceous, Barremian and Berriasian.

Type locality: Ostrov – the southern border of Bugeac Lake, Romania.

+ *Valvata neglecta* Brusina, 1902

Original source: Brusina 1902: pl. 13: figs 35–38.

Type horizon: Upper Miocene, Pannonian (Transdanubian).

Type locality: Rădmănești, Romania.

+ *Valvata nevadensis* Taylor, 1981

Original source: Taylor and Smith 1981: 357, pl. 3: fig. 7–12.

Type horizon: Pliocene.

Type locality: Honey Lake, Lassen County, California, USA.

+ *Valvata vanciana* var. *neyronensis* Locard, 1883

Original source: Locard 1883a: 22.

Type horizon: Middle Pliocene, Plaisancien.

Type locality: Bas Neyron, Département de Ain, France.

*Valvata nilotica* Jickeli, 1874

Original source: Jickeli 1874: 233–235, pl. 7: fig. 29a–c.

Type locality: Mahmudi Canal, Nile river near Alexandria, Egypt.

“*Valvata nitens* Westerlund, 1877” (GNI, ION)

Possibly confused with *Helix nitens* Michaud, 1831 in Westerlund (1886: 64), currently regarded as *Aegopinella nitens* (Michaud, 1831) (**Zonitidae**).

“*Valvata nitida*” (GNI)

Probably an erroneous combination of *Planorbis nitida* Müller, 1774, currently considered as *Segmentina nitida* (Müller, 1774) (**Planorbidae**).

*Valvata bicarinata normalis* Walker, 1902

Original source: Walker 1902: 125: fig. 6.

Type locality: Muscatine, Iowa and Utica, Illinois, USA.

Remarks: Radula shown in Baker (1928: 20).

*Valvata nowshahrensis* Glöer & Pešic, 2012

Original source: Glöer and Pešic 2012: 38, figs 14a–c.

urn:lsid:zoobank.org:act:944E6EE3-B23C-43FB-A305-882A4D4CF3D9

http://species–id.net/wiki/Valvata_nowshahrensis

Type locality: Mazandaran Province, Nowshahr City, pond near the Caspian Sea, 51°31'E, 36°38'N, 18 June 2005; Iran.

Holotype Zoological Museum Hamburg #79376.

*Valvata (Liratina) piligera* var. *nudicarinata* Lindholm, 1924

Original source: Lindholm 1924: 217.

Type locality: Lake Baikal, Russia.

Lectotype designated by Sitnikova et al. (1983) and deposited at Zoological Institut of the Russian Academy of Sciences, St. Petersburg; Nr. 96 in systematic catalogue.

*Valvata micra* var. *nugax* Pilsbry & Ferriss, 1906

Original source: Pilsbry and Ferriss 1906: 173, pl. 9: figs 6.

Type locality: Guadeloup River, Texas, USA.

Remarks: On basis of anatomy Hershler and Longley (1986) classified this taxon in *Phreatodrobia* Hershler & Longley, 1986 (**Hydrobiidae**).

*Valvata sincera* var. *nylanderi* Dall, 1905

Original source: Dall 1905: 122, pl. 1: figs 7–9.

Type locality: Aroostook Co., Maine, USA.

Holotype: U.S. National Museum #150617.

Remarks: Radula shown by Baker (1928a: 26).

+ *Amplovalvata obliqua* Pan, 1982 (in Guo et al. 1982)

Original source: Guo et al. 1982: 33, pl. 13: figs 4–5.

Type horizon: Middle Jurassic.

Type locality: Atlas Shanganning, China.

*Nerita obtusa* “Müll.” Studer, 1789

Original source: Studer 1789: 391 (presented as “*Nerita obtusa* MULL. 358 sive *Piscinalis*”).

Remarks: Studer 1789: 391 established the new name and provided a bibliographical reference to Müller (1774: 172; No. 358 in Müller’s list). Müller (1774) described *Nerita piscinalis* (= currently *Valvata piscinalis* (Müller, 1774)). Studer’s (1789) name *Nerita obtusa* was made available under ICZN Art. 12.2.1. The types could be those of Müller’s (1774) presentation (from Fridrichsdal, Denmark) or those from Switzerland to which Studer had had access.

+ *Valvata (Cincinna) obtusaeformis* Lörenthey, 1906

Original source: Lörenthey 1906: 174, pl. 3: figs 20a, b.

Type horizon: Upper Miocene, Pannonian.

Type locality: Öcs, north of Lake Balaton, Hungary.

*Cyclostoma obtusum* Draparnaud, 1801

Original source: Draparnaud 1801: 39–40; figured as *Valvata obtusa* by Brard (1815: p. 190, pl. 6: fig. 17).

Type locality: “France septemtrionale”.

Types possibly in Naturhistorisches Museum Vienna (Kantor et al. 2011: 69).

Remarks: Draparnaud (1805: 101) cited *Nerita piscinalis* as a senior synonym of *Cyclostoma obtusum*, among other synonyms.

*Valvata (Atropidina) ochridana* Poliński, 1929

Original source: Poliński 1929: 136–137.

Type locality: According to Radoman (1985: 115): “Lake Ohrid, in the *Chara* zone in the Ohrid gulf”, Macedonia.

Remarks: Type species of *Pseudohoratia* Radoman, 1967 (**Hydrobiidae**), see Kabat and Hershler (1993: 46) and Bodon et al. (2001: 150).

+ *Valvata octonaria* Brusina, 1902

Original source: Brusina 1902: pl. 13: figs 5–8. Referred to Brusina (1894: 181), a nomen nudum.

Type horizon: Upper Miocene, Pannonian, horizon of Lyrecaea (Melanopsis).

Type locality: Lake Balaton, Tihany, Hungary.

+ *Valvata (Valvata) simplex oecsensis* Soós, 1934

Original source: Soós 1934: 189, fig. 1 (as *Valvata öcensis*)

Type horizon: Upper Miocene, Upper Pannonian.

Type locality: Öcs, north of Lake Balaton, Remarks: SEM of shell and (typically valvatid) protoconch were depicted by Harzhauser and Binder (2004: 10, pl. 3: figs 9–11).

+ *Valvata ogerieni* Locard, 1883

Original source: Locard 1883a: 131, pl. 4: figs 1–3.

Type horizon: Middle Pliocene.

Type locality: Le Villard, Domsure, Département de l’Ain, France.

Remarks: Cited as “*Valvata ogerieni* Loc.” in Chaignon (1893: 611), showing that the paper by Locard (1883a) was indeed published in 1883 rather than in 1888 as stated by Wenz (1923: 115).

“*Valvata olgae*“ mentioned in Siodiropoulou (2003: 34–35, fig. 11)

Horizon: Pliocene-Pleistocene.

Locality: Ptolemaida, West Macedonia, Greece.

Remarks: This PhD–Thesis PhD–Thesis, in which 35 species are ostendibley described, does not meet the conditions of ICZN Art. 8.1.3 and 9.9. Accordingly, this name is not available.

*Valvata (Pseudomegalovalvata) olkhonica* Bekman & Starobogatov, 1975

Original source: Bekman and Starobogatov 1975: 94: fig. 1B.

Type locality: Kharin-Irgi Bay (Oikhon Gates) (Baikal Lake), depth 32–39 m, Russia.

Holotype: Zoological Institute of the Russian Academy of Sciences, St. Petersburg, Nr. 1 in systematic catalogue.

*Valvata lewisi* var. *ontariensis* Baker, 1931

Original source: Baker 1931: 119–121.

Type locality: Shakespeare Island Lake, Ontario, Canada.

Types: Museum of Natural History University of Illinois #Z31241; Academy of Natural Sciences of Drexel University, Philadelphia #153471.

Remarks: According to Hanson et al. (2002) a loosely coiled form of *Valvata lewisi* Currier, 1868.

“*Valvata sincera ontariensis* Baker, 1931” (WMSDB).

Erroneous spelling of *Valvata lewisi* var. *ontariensis* Baker, 1931

“*Valvata opaca*” www.naturamediterraneo.com/forum/topic.asp?TOPIC_ID=40300 at the “Check–list Pen. Iberica con sinonimie”

Probably confused with *Paludinella opaca* M. von Gallenstein, 1848, currently regarded as a *Bythinella* (**Hydrobiidae**).

+ *Valvata oregonensis* Hanna, 1922

Original source: Hanna 1922: 11–12, pl 3: figs 1–18, pl. 4: figs 1–4.

Type horizon: Pliocene.

Type locality: Pliocene; Warner Lake Beds, Oregon, USA.

Holotype figured at http://en.wikipedia.org/wiki/Valvata_oregonensis

Remarks: Taylor (1966: 132) synonymized the taxon with *Valvata whitei* Hannibal, 1910, whereas Pierce (1993) considered two species.

+ *Valvata orientalis* Fischer, 1866

Original source: Fischer 1866: 345, pl. 6: fig. 7.

Type horizon: Upper Miocene.

Type locality: Sarayköy (= Saraïkoï) near Denizli (vallée du Méandre), western Asian, Turkey.

+ *Valvata ottiliae* Penecke, 1886

Original source: Penecke 1886: 37, pl. 10 (resp. 7 in reprint): figs 1–2.

Type horizon: Pliocene - Pleistocene.

Type locality: Dacian-Romanian; Repušnica, southeast of Zagreb, Croatia.

Remarks: Stefanescu (1896: 125) considered this species a junior synonym of *Valvata balteata* Brusina, 1897.

“*Valvata ouscubakus* Nykk., 1895”.

Error probably caused by text recognition software pro “*Valvata piscinalis* Müll. 1774” attributed at http://content.lib.washington.edu/ to fig. 66 for Cooke (1895).

+ *Valvata (Costovalvata) pagana* Bulić & Jurišić, 2009

Original source: Bulić and Jurišić 2009: 143, pl. 3, figs 9–10.

Type horizon: Lower Miocene.

Type locality: Crnika, Island of Pag, Croatia.

+ *Valvata palmotići* Brusina, 1902

Original source: Brusina 1902: pl. 13: figs 1–4.

Type horizon: Pliocene – Pleistocene, Dacian-Romanian.

Type locality: Mali Poganac near Lepavina, Croatia.

“*Valvata piscinalis* var. *paludinaeformis*“ mentioned in Braun (1843, 144) (nomen nudum with locality)

Horizon: Tertiary.

Locality: Mainzer Becken; Germany.

“*Valvata palustris*” (GNI)

Possibly an erroneous combination for *Lymnaea palustris* Müller, 1774, currently considered as *Stagnicola palustris* (Müller, 1774) (**Lymnaeidae**).

*Valvata (Cincinna) pamirensis* Starobogatov, 1972

Original source: Starobogatov 1972: 170–171: fig. 9.

Type locality: Gorno-Badakhshan Autonomous Region, Shaimak, 7 km from Kyzyl-Ravat, warm spring on right bank of the Aksu River, Tajik SSR.

Holotype: Zoological Institute of the Russian Academy of Sciences, St. Petersburg, Nr. 1 in systematic catalogue under the name.

Remarks: Genital details are shown by Sitnikova (1983: fig. 1/4).

“*Valvata panagile*” mentioned in Siodiropoulou (2003: 41–42, fig. 16).

Locality: Pliocene – Pleistocene; Ptolemaida, West-Macedonia, Greece.

Remarks: This PhD–Thesis PhD–Thesis, in which 35 species are ostendibley described, does not meet the conditions of ICZN Art. 8.1.3 and 9.9. Accordingly, this name is not available.

*Valvata panormitana* Locard, 1889

Original source: Locard 1889: 51 (footnote: attributed to Bourguignat, but ICZN Art. 50.1.1 is not satisfied).

Type locality: near Palermo, Sicily, Italy.

*Valvata parva* Locard, 1889

Original source: Locard 1889: 50 (footnote: attributed to Bourguignat, but ICZN Art. 50.1.1 is not satisfied).

Type locality: on la trouve à Viareggio, Italy.

+ *Valvata parviumbilicata* Wang, 1977 (in Yü and Wang 1977)

Original source: Yü and Wang 1977: 15–16, pl. 1: figs 17–19.

Type horizon: Upper Cretaceous.

Type locality: Jiangsu Province, China.

+ *Valvata parvula* Meek & Hayden, 1856 (non Deshayes, 1862)

Original source: Meek and Hayden 1856: 123, also listed by Meek and Hayden (1876: 591 with a detailed description of the species but with clear reference to Meek and Hayden 1856).

Type horizon: Tertiary.

Type locality: “3 miles below Fort Union” (a historical post-side) on the Missouri River at the North Dakota/Montana border, USA.

Remarks: Seemingly replaced by + *Valvata subparvula* Cossmann, 1921. Cossmann (1921: 170, first line and footnote) refers to *Valvata parvula* as listed by Meek and Hayden (1876: 591) and thus later than *Valvata parvula* Deshayes, 1862 – which is not the case, however. *Valvata parvula* Meek & Hayden, 1856 still is a valid name.

+ *Valvata parvula* Deshayes, 1862 (non Meek & Hayden, 1856)

Original source: Deshayes 1861–1863, Vol. 2: 526, pl. 36: figs 12–14.

Type horizon: Upper Pliocene.

Type locality: Jonchery, Gueux, Rilly, Basin de Paris, France.

Remarks: Replaced by + *Valvata (Cincinna) joncheryensis* Wenz, 1930: 65 (see there).

*Valvata (Megalovalvata) lauta* var. *parvula* Kozhov, 1936 (not + *Valvata parvula* Meek & Hayden, 1856, not + *Valvata parvula* Deshayes, 1862).

Original source: Kozhov 1936: 25.

Type locality: Davsha Inlet, 9 fathoms (eastern coast of northern Baikal), Russia.

Lectotype designated by Sitnikova et al. (1983) and deposited at Zoological Institut of the Russian Academy of Sciences, St. Petersburg; Nr. 3 in systematic catalogue under the name.

*Valvata humeralis* var. *patzcuarensis* Pilsbry, 1899

Original source: Pilsbry 1899: 392.

Type locality: Lago de Patzcuaro, Michoacán, Mexico.

Remarks: Probably a lake variety of *Valvata humeralis* Say, 1829. Pilsbry (1903) himself admitted that the taxon is a junior synonym (published Oct. 1899) of *Valvata humeralis pilsbryi* Martens, 1899 (published Sept. 1899).

“*Valvata humeralis* var. *patzcuaroensis* Pilsbry, 1899”

Misspelling of *Valvata humeralis* var. *patzcuarensis* Pilsbry, 1899 at page 195 at www.flmnh.ufl.edu/malacology/mexico-central_america_snail_checklist/part1.htm

+ *Valvata paula* Pierce, 1993

Original source: Pierce 1993: 980, figs 1.1–1.4.

Type horizon: Upper Oligocene – Lower Cabbage.

Type locality: Powel County, Montana, USA.

Holotype at http://invertebratepaleontology.biodiversity.ku.edu/galleries/kumip-holotypes-pierce-1993#photo-1 (first photo)

+ *Valvata (Cincinna) paviai* Schlickum & Strauch, 1979

Original source: Schlickum and Strauch 1979: 11–12, pl. 1: fig. 5.

Type horizon: Pliocene, brown coal area.

Type locality: Bergheim (abandoned opencast mine Fortuna-Garsdorf), Nordrhein-Westfalen, Germany.

*Valvata pedderi* Smith, 1973

Original source: Smith 1973: 430.

Type locality: Lake Edgar (part of Lake Pedder), Tasmania.

Holotype: Tasmanian Museum #E8543.

Type species of *Striadorbis* Ponder & Avern, 2000 (Euthyneura - **Glacidorbidae**), see Ponder and Avern (2000: 339).

+ *Valvata peneckei* Brusina, 1892

Original source: Brusina 1892: 167. Figured by Brusina (1902: pl. 13: figs 18–21).

Type locality: Pliocene, Paludina–layers; Repusnica, Slavonia.

Remarks: Replacement name for *Valvata bifrons* Neumayr, 1875 sensu Penecke (1886: 37).

+ *Cincinna penglaizhenensis* Pan, 1982

Original source: Pan 1982: ??, figs 22–23 (not seen, data from online type catalogue of Nanjing Institute).

Type horizon: Jurassic, Penglaizhen.

Type locality: Penglai town, Sichuan Basin, China.

Holotype: http://159.226.74.248:8000/viewSpeciDetailsNormal.jsp?bbbh=53435

“*Valvata penthica*” (GNI, ION)

Probably an erroneous combination for *Viviparus fasciatus* var. *penthica* Servain, 1884.

*Valvata tricarinata perconfusa* Walker, 1917

Original source: Walker 1917: 36.

Remarks: Replacement name for the preoccupied *Valvata confusa* Walker, 1902 , from North America, not *Valvata confusa* Westerlund, 1897, from Siberia.

“*Valvata tricarinata perconfuxa* Walker” (google)

Misspelling of *Valvata tricarinata perconfusa* Walker, 1917.

*Valvata bicarinata perdepressa* Walker, 1906

Original source: Walker 1906: 130, pl. I: figs 15–16.

Type locality: South shore (at Michigan City) of Lake Michigan, Indiana, USA.

Remarks: Vernacular name: purplecap valvata.

+ *Liratina peronata* Pan, 1980 (in Yü and Pan 1980)

Original source: Yü and Pan 1980: 148, pl. 2: figs 11–17.

Type horizon: Middle Jurassic.

Type locality: Junggar Basin, Fukang County Dahonggou, China

Holotype: http://159.226.74.248:8000/viewSpeciDetailsNormal.jsp?bbbh=36285

“*Valvata perroquini*” mentioned in Hagenmüller (1884: 216)

Remarks: clearly refers to *Heterocyclus perroquini* Crossé, 1872 (**Hydrobiidae**)

“*Valvata persimilis*“ mentioned in Férussac and Férussac (1807: 128–129) (nomen nudum)

*Valvata petiti* Crosse, 1872

Original source: Crosse 1872: 157, 353–354, pl. 16, fig. 7.

Type locality: Lac de la Grande, Vallée de Kaoris, New Caledonia.

Remarks: According to Solem (1961: 429) a synonym of *Heterocyclus perroquini* Crosse, 1872: 156 (**Hydrobiidae**)

*Valvata petrettinii* Innes, 1884

Original source: Innes 1884: 349 (attributed to Bouguignat, but ICZN Art. 50.1.1 is not satisfied).

Type localities: “…dans les canaux d’Alexandrie et de Rosette, …… dans les sables de Mandarah, entre Ramleh et la cap Aboukir”, all Egypt.

+ *Valvata (Cincinna) petronijevici* Miloshevich, 1973

Original source: Miloshevich 1973: 143, textfig. 1. Also figured by Miloshevich (1984: pl. 1: figs 11–17).

Type horizon: Pliocene, Kosovo Series (topmost horizon).

Type locality: Pećka Bistrica stream outlet, Drsnik region, Metohija Basin, Kosovo.

*Valvata pharaonum* Innes, 1884

Original source: Innes 1884: 351

Type localities: “Bords du lac Moeris, au Fayoun”, Egypt.

“*Valvata phialensis*“ mentioned in Şereflişani et al. (2009: 291/fig. 2)

Probably an erroneous combination of *Bithynia phialensis* (Conrad, 1852) and *Valvata piscinalis* (Müller, 1774).

+ *Valvata (Aegaea) philippsoni* Oppenheim, 1891

Original source: Oppenheim 1891: 473, pl. 28, figs 6a–d.

Type horizon: Pleistocene, Chaudian (cf. Gillet et al. 1979).

Type locality: Arkitsa near Livanates, Greece.

+ *Valvata alpestris* var. *piattii* Adami, 1881

Original source: Adami 1881: 198.

Type horizon: Post-Pliocene.

Type locality: Torbiera di Polada, Northern Italy.

“*Valvata piacinalia*” (GNI, ION)

Error caused by text recognition software pro *Valvata piscinalis* (Müller, 1774).

+ *Valvata (Atropidina) pileiformis* Youluo, 1978 (name of author according to the type catalogues of Nanjing Institute)

Original source: Youluo 1978: 29–30, pl. 3: figs 10–15.

Type horizon: Lower Tertiary.

Type locality: coastal region of Bohai, China.

*Valvata (Liratina) baicalensis* var. *piligera* Lindholm, 1909

Original source: Lindholm 1909: 78.

Type locality: Lake Baikal, around Island Olchon, Russia.

Lectotype designated by Sitnikova et al. (1983) and deposited at Zoological Institut of the Russian Academy of Sciences, St. Petersburg; Nr. 29 in systematic catalogue.

Remarks: Kantor et al. (2011: 73) considered the species as *Megalovalvata piligera piligera* (Lindholm, 1909). Spawn depicted at: http://userpage.fu-berlin.de/~rpeter/deutsch/repro/ei_valva.html

*Valvata humeralis* var. *pilsbryi* Martens, 1899

Original source: Martens 1899: 427.

Type locality: Lago de Patzcuaro, Michoacán, Mexico.

*Nerita piscinalis* Müller, 1774

Original source: Müller 1774: 172 (Nr. 358).

Type locality: “In piscina horti Fridrichsdalenjis frequens, nec unquam alibi reperi” [frequent in fish ponds of Fridrichsdal], i.e. probably near Copenhagen, Denmark.

Types possibly in Zoological Museum Copenhagen (Kantor et al. 2011: 70).

Remarks: (1) Anatomy studied by Bernard (1888, 1890) and Cleland (1954).

(2) Ontogeny studied by Rath (1986), for life cycle see Myzyk (2007).

(3) SEM photos of shell and radula provided by Falniowski (1990) and Anistratenko et al. (2010: fig. 5A–D).

(4) Live photos at: http://www.allesumdieschneck.de/html/valvata_piscinalis_piscinalis.html

+ *Valvata piscinaloides* Michaud, 1855

Original source: Michaud 1855: 48 (in issue of journal, 16 in reprint), pl. 5: figs 20–21.

Type horizon: Lower Pliocene, Zanclean.

Type locality: Hauterive, Département de Drôme, France.

“*Valvata piscinnalis* Müll.” mentioned in Férussac and Férussac (1807: 75).

Misspelling of *Valvata piscinalis* (Müller, 1774).

+ *Valvata pisidica* Oppenheim, 1918

Original source: Oppenheim 1918: 206–207, pl. 7: figs 4–6.

Type horizon: Upper Pliocene – Lower Pleistocene.

Type locality: Eflatun Pınar (= Efflatum-Bunar) at Beysehir Gölü, West-Turkey.

+ *Protovalvata plana* Pană, 2000

Original source: Pană 2000: 89, pl. 5: figs 20–21.

Type horizon: Lower Cretaceous, Barremian.

Type locality: Ostrov – the southern border of Bugeac Lake (Lake Gârlita), Northern Dobruja, Romania.

+ *Valvata planconcava* Pavlović, 1928

Original source: Pavlović 1928: 62.

Type horizon: Upper Miocene, Pannonian (Serbian).

Type locality: Karagača stream (Vrčin SSE Belgrade), Serbia.

“*Valvata plaiti*” (GNI)

Error caused by text recognition software pro + *Valvata alpestris* var. *piattii* Adami, 1881.

+ *Valvata planibasis* Cossmann, 1899

Original source: Cossmann 1899a: 349–350 (in issue of journal, 44–45 in reprint), textfîg. 5.

Type horizon: Eocene.

Type locality: Bois-Gouët, Lower Loire, Bretagne, France.

*Valvata planorbis* Draparnaud, 1801

Original source: Draparnaud 1801: 42.

Type locality: France.

Remarks: Generally (e.g. Menke 1845) regarded as synonym to *Valvata cristata* Müller, 1774.

*Valvata planorbulina* Paladilhe, 1867

Original source: Paladilhe 1867b: 49–50, pl. 3: figs 23–26.

Type locality: «dans des alluvions du Lez», France.

Types not traced.

*Valvata planulata* Innes, 1884

Original source: Innes 1884: 351.

Type locality: “Bords du lac Moeris”, Egypt.

Remarks: Innes (1884: 351) refers to “Bourguignat, Spec. Moll., n^o^. 197, 1878”.

However, as outlined by Connolly (1934), the latter works was never published.

+ *Valvata virens platyceps* Pilsbry, 1935

Original source: Pilsbry 1935: 565, text fig. 2c–d.

Type horizon: Pliocene.

Type locality: Kettleman Hills, California, USA.

+ *Valvata saulcyi pliocaenica* Schütt, 1988

Original source: Schütt 1988: 133, pl. 3: fig. 26.

Type horizon: Lower Pleistocene (?).

Type locality: left border of Orontes river (Nahr al-Asi), 12 km south of Ğisr aš-Šugur, Syria.

+ *Cincinna (Cincinna) piscinalis pliocaenicus* [sic] Gozhik, 2007 [should be *pliocaenica*, since *Cincinna* is feminine]

Original source: Gozhik 2007: 78, pl. 68: figs 1–4.

Type horizon: Pliocene (Alluvium XI. terrace).

Type locality: river Pruth near Kuzhbovka; Moldavia.

“*Cincinna (Cincinna) pliocalnicus*“ mentioned in Gozhik (2007: 122).

Misspelling of + *Cincinna (Cincinna) piscinalis pliocaenicus* Gozhik, 2007.

“*Valvata* (*Ochridotropina*) [sic] *polinskii*” in Hadžišče (1955: 176).

As outlined by Hadžišče 1956: 58 (footnote), this name is based on an error and should be *Valvata (Ohridotropina) relicta* Poliński, 1929.

+ *Valvata politioanei* Jekelius, 1944

Original source: Jekelius 1944: 55, pl. 7: figs 20–23.

Type horizon: Middle Miocene, Sarmatian.

Type locality: Soceni (Banat), Romania.

+ *Valvata simplex* var. *polycincta* Lörenthey, 1906

Original source: Lörenthey 1906: 167, pl. 1: fig. 17, pl. 3: fig. 21.

Type horizon: Upper Miocene, Upper Pannonian.

Type locality: Tihany, Lake Balaton, Hungary.

+ *Valvata polystriata* Pavlović, 1928

Original source: Pavlović 1928: 61.

Type horizon: Upper Miocene, Pannonian (Serbian).

Type locality: Karagača stream (Vrčin SSE Belgrade), Serbia.

*Valvata pornae* Locard, 1889

Original source: Locard 1889: 25 (attributed to Bourguignat, but ICZN Art. 50.1.1 is not met).

Type localities: (1) Toscana; (2) near St. Germano, Campania, both Italy.

+ *Cincinna (Atropidina) cobalcescui porrecta* Gozhik, 2007

Original source: Gozhik 2007: 80, pl. 72: figs 1–2.

Type horizon: Upper Miocene – Lower Maeotian.

Type locality: Lower Dnepr, Ukraine.

+ *Valvata praecursor* Tate, 1873

Original source: Tate 1873: 348, pl. 12: fig. 9.

Type horizon: Jurassic, Infra-Oxfordian.

Type locality: Prince Charles Cave, Portree, Skye Island, Scotland, U.K.

+ *Planorbis praecursoris* White, 1895

Original source: White 1895: 46, pl. 6: figs 4–7. As + *Valvata praecursoris* (White) cited and figured in Yen (1950a: 14, pl. 2: figs 1a–g).

Type horizon: Cretaceous.

Type locality: 20 miles north of Cokeville, Wyoming, USA.

+ *Cincinna (Cincinna) praepiscinalis* Gozhik, 2007

Original source: Gozhik 2007: 75. Was before listed and figured as “*Valvata* ex gr. *piscinalis*” in Gozhik and Prysjazhnjuk (1978: 65, pl. 1: figs 5–6).

Type horizon: Middle or Upper Miocene – Sarmatian.

Type locality: Mykhailivka (= Michailovka), Ukraine.

+ *Valvata lewisii precursor* Baker, 1928

Original source: Baker 1928: 136–137.

Type horizon: Pleistocene, Lower Wisconsin.

Type locality: Fulton County, east of Havana, Illinois, USA.

“*Valvata priscinalis* Wood 1848” (EOL: Location Great Britain).

Misspelling of *Valvata piscinalis* (Müller, 1774).

+ *Valvata (Cincinna) proavia* Huckriede, 1967

Original source: Huckriede 1967: 167, pl. 23: figs 15a–27b, 42a–43b, pl. 24: figs 2a–4b.

Type horizon: Upper Jurassic, Middle Kimmeridge.

Type locality: Kahlberg in the Harz, Germany.

+ *Gyraulus procerus* Russell, 1952

Original source: Russell 1952: 131–132, textfig. 10, pl. 19, figs 3–13. Cited as *Valvata procera* (Russell, 1952) in Ross (1960: 70: “*Gyraulus procerus* Russell is apparently a *Valvata*,..” ) and in Pierce et al. (2001).

Type horizon: Upper Eocene / Lower Oligocene, Kishenehn formation (Ross 1960: 69).

Type locality: valley of North Fork of the Flathead River in southeastern British Columbia, Canada.

*Valvata profunda* Clessin, 1887

Original source: Clessin 1887: 776: fig. 509.

Type locality: Lake Garda, Trentino, Italy.

*Valvata (Pseudovalvata) profundicola* Bekman & Starobogatov, 1975

Original source: Bekman and Starobogatov 1975: 95: fig. 1C.

Type locality: near Bolsodej Cape [Baikal Lake], depth 300 m, Russia.

Holotype: Zoological Institute of the Russian Academy of Sciences, St. Petersburg, Nr. 1 in systematic catalogue under the name.

Remarks: Genital details are depicted by Sitnikova (1983: fig. 1/5), SEM of shell, protoconch, and radula is shown by Röpsdorf and Riedel (2004: fig. 7A–I).

+ *Valvata (Megalovalvata) protopiligera* Martinson, 1961

Original source: Martinson 1961: 247, pl. 22: figs 9–10.

Type horizon: ??

Type locality: Tarbagatay, Zabaykalsky Krai (=Trans-Baikal Territory), Eastern Sibiria, Russia.

“?*Valvata proxima* (Fuchs)“ mentioned in Müller (1989: 576, legend to plate 6: fig. 12).

Confusion with ?*Bithynia proxima* Fuchs (1870b: 534, pl. 20: figs 34–36).

+ *Cincinna (Cincinna) prutulense* [sic] Gozhik, 2007 [should be *prutulensis*, since *Cincinna* is feminine]

Original source: Gozhik 2007: 77, pl. 73: figs 1–4.

Type horizon: Pliocene (Alluvium).

Type locality: river Yalpug near the village Kotlovina (Bolboaka), district of Odessa, Ukraine.

+ *Valvata pseudoadeorbis* Sinzov, 1880

Original source: Sinzov 1880: 14, pl. 8: 53–55 (for author spelling of author see reference list).

Type horizon: Upper Miocene, Sarmatian.

Type locality: Simbirsk and Saratov governments, Russia.

Remarks: (1) + *Valvata pseudoadeorbis* Sinzov, 1880 from the Eastern Paratethys (like the type locality) has been recently considered to be a hydrobiid by Anistratenko O. and Anistratenko V. (2012: 121 footnote).

(2) “*Valvata (Aphanotylus) pseudo-adeorbis* Sinz. (?)” used in Papp (1939: 352) (Sarmatian, Vienna Basin), also figured by Papp (1974: 334, pl. 3: figs 3–4) has been described as + *Valvata (Valvata) exotica* Papp, 1954 (see there) and shows the typical valvatid protoconch (cf. Harzhauser and Kowalke 2002: 74 under *Valvata pseudoadeorbis* Sinzov).

“*Valvata* (?*Aphanotylus*) *pseudoadeorboides* Sinzow“ mentioned in Wenz (1928b: 2463)

Consistent misspelling of *Valvata pseudoadeorbis* Sinzow, 1880, but cited to “Sinzow, 1883: pages, 78 and 93”.

+ *Valvata pseudoalpestris* Brusina, 1902

Original source: Brusina 1902: pl. 13: fig. 33.

Type horizon: Pliocene – Pleistocene, Dacian-Romanian.

Type locality: Rešetari, Slavonia, Croatia.

+ *Valvata (Cincinna) piscinalis* var. *pseudoantiqua* Settepassi, 1965 (in Settepassi and Verdel 1965)

Original source: Settepassi and Verdel 1965: 382.

Type horizon: Quaternary.

Type locality: C. Sisti (Pontecorvo), Southern Latium, Romania.

*Valvata pulchella* Studer, 1789 (in Coxe 1789)

Original source: Coxe 1789: 391.

Type locality: “Umgebung des Bielersee’s in Wassergräben”, Switzerland (Studer’s specimens) and Rivière des Gobelins, Paris, France (specimens from Geoffroy 1767).

Remarks: *Valvata pulchella* Studer, 1789 was established without description, but with a reference to “Geofr. Ner. 4” (= Geoffroy 1767: 115) that made the name available under ICZN Art. 12.2.1. (although the latter work has been rejected for nomenclatorical purposes by ICZN Opinion 362). Boeters and Falkner (1998) considered this name as a junior synonym of *Nerita piscinalis* Müller, 1774. The name was subsequently used by Studer (1820: 91) and misapplied to a species that later turned out to be different (i.e. *Valvata studeri* Boeters & Falkner, 1998; see there). Forcart’s (1957: pl. 4: fig. 22) lectotype designation for *Valvata pulchella* in Studer (1820) (also figured by Boeters and Falkner 1998: pl. 14: fig. 1) is invalid, because an unavailable name has no types at all.

*Valvata pupoidea* Gould, 1841

Original source: Gould 1841: 226: fig. 155.

Type locality: Fresh Pond, Cambridge, Middlesex County, Massachusetts, U.S.A.

Remarks: Type species of *Lyogyrus* Gill, 1863 (**Hydrobiidae** or **Amnicolidae**), cf. Walker (1918: 33), Berry (1943: 57) or Kabat and Hershler (1993).

*Nerita pusilla* Müller, 1774

Original source: Müller 1774: 171 (Nr. 357).

Type locality: “in lacu Ruppinensi”, Ruppiner See, Brandenburg, Germany.

Types unknown, possibly in Zoological Museum Copenhagen (Kantor et al. 2011: 73).

Remarks: Considered a *Valvata* by Menzel (1904a: 116).

*Valvata pusilla* Piersanti, 1951 (non Martinson, 1961)

Original source: Piersanti 1951: 1–3, pl. 1.

Type locality: Frasassi cave system near San Vittore, Genga Ancona, Italy.

Remarks: According to Giusti et al. (1982) and Bodon et al. (2001: 176) now *Islamia pusilla* (Piersanti, 1951) (**Hydrobiidae**).

+ *Valvata pusilla* Martinson, 1961 (non Piersanti, 1951)

Original source: Martinson 1961: 247, pl. 22: figs 12–13.

Type horizon: ??

Type locality: Nemegt uul, Mongolia.

*Valvata pygmaea* C.B. Adams, 1849 (non Noulet, 1854; non Moore, 1867)

Original source: C.B. Adams 1849: 42–43.

Type locality: Island Jamaica.

Remarks: The types of the Museum of Camparative Zoology (Harvard) were studied and a lectotype was designated and figured by Johnson and Boss (1972: 213–214, pl. 41: fig. 6).

+ *Valvata pygmaea* Noulet, 1854 (non Adams, 1849; non Moore, 1867)

Original source: Noulet 1854: 55–56.

Type horizon: Middle Eocene.

Type locality: Molasse de Castellnaudary; Département de Tarn, France.

Remarks: An objective junior homonym of *Valvata pygmaea* Adams, 1849.

+ *Valvata pygmaea* Moore, 1867 (non Adams, 1849; non Noulet, 1854)

Original source: Moore 1867: 557, pl. 15: figs 5–6.

Type horizon: Lower Jurassic.

Type locality: Charterhouse Mine, South Wales, U.K.

Remarks: An objective junior homonym of *Valvata pygmaea* Adams, 1849.

+ *Liratina qikouensis* Youluo, 1978 (name of author according to the type catalogues of Nanjing Institute)

Original source: Youluo 1978: 33, pl. 4, figs 1–3.

Type horizon: Lower Tertiary.

Type locality: coastal region of Bohai, China.

“*Valvata raboi* Anon 1889” (GNI)

Misspelling of *Valvata raboti* Westerlund, 1889.

*Valvata piscinalis raboti* Westerlund, 1889

Original source: Westerlund 1889: 1316 (footnote).

Type locality: Norvegia in Finmarkia orientali ad Klostervand (Flumen Pasvig.), Finland.

+ *Valvata radiatula* Sandberger, 1875

Original source: Sandberger 1875: 576, pl. 30: figs 7–7c.

Type horizon: Middle Miocene, Tortonian.

Type locality: Schwenditobel near Pfrungen, Baden-Württemberg, Germany.

+ *Valvata radovanovići* Pavlović, 1931

Original source: Pavlović 1931: 26, pl. 11: figs 27–30.

Type horizon: Upper Miocene- Pliocene (“Pontian *Congeria*–layers”).

Type locality: Basin of Skopje, Macedonia.

+ *Valvata (Turrivalvata) ranjinai* Brusina, 1902

Original source: Brusina 1902: pl. 13: figs 39–41.

Type horizon: Upper Pliocene–Lower Pleistocene, Romanian.

Type locality: Kindrovo, Slavonia, Croatia.

*Valvata regalis* Locard, 1889

Original source: Locard 1889: 38 (attributed to Bourguignat, but ICZN Art. 50.1.1 is not met).

Type localities: (1) Königsee in Bavaria, Germany, (1) Lake Tristach near Lienz, East-Tyrol, Austria.

+ *Valvata (Cincinna) rehetaiensis* Youluo, 1978 (name of author according to the type catalogues of Nanjing Institute)

Original source: Youluo 1978: 30–31, pl. 3: figs 16–21.

Type horizon: Lower Tertiary.

Type locality: coastal region of Bohai, China.

*Gyraulus (Carinogyraulus) relictus* Poliński, 1929

Original source: Poliński 1929: 164.

Type locality: Lake Ohrid, Albania/Macedonia.

Remarks: German translation of Poliński (1929) by Poliński (1932): Valvatid nature has been shown by anatomy (Rath 1986: 89ff) and by molecular data (Hauswald et al. 2008).

*Valvata revoili* Bourguignat, 1889

Original source: Bourguignat 1889: 189, pl. 8: figs 5–6.

Type locality: Market of Moguedoushou (Mogadishu), Somalia.

Remarks: Considered to be a **terrestrial species** (Mandahl-Barth in Brown 1965: 46). According to Van Damme (1984) this is probably a distinct species, but taxonomic status needs to be confirmed.

*Valvata rhabdota* Sturany, 1894

Original source: Sturany 1894: 381, pl. 19(II): figs 18–20.

Type locality: near Ohrida in Lake Ohrid, 30 m (only dead shells), Macedonia.

Remarks: Valvatid nature has been shown by anatomy (Rath 1986: 111ff) an molecular data (Hauswald et al. 2008).

+ *Valvata ringentis* Youluo 1978 (name of author according to the type catalogues of Nanjing Institute)

Original source: Youluo 1978: 29, pl. 3: figs 4–6.

Type horizon: Lower Tertiary.

Type locality: coastal region of Bohai, China.

+ *Aphanotylus ristici* Pavlović, 1931

Original source: Pavlović 1931: 26, pl. 11: figs 42–47. As *Valvata (Aphanotylus) ristići* (Pavlović, 1931) cited and figured in Milosević (1984: 10, pl. 1: fig. 1–5).

Type horizon: Pliocene−Pleistocene, Metohija Series (Levantin de Topelić).

Type locality: Topličane, Metohija basin, Kosovo.

+ *Valvata robusta* Martinson, 1982

Original source: Martinson 1982: 70, pl. 16: figs 23–24.

Type horizon: Upper Cretaceous.

Type locality: exact location ??, Mongolia.

*Vallonia rosalia* Risso, 1826

Original source: Risso 1826: 102 (#237)

Type locality: Alpes maritimes.

Remarks: According to Küster (1852–1853: 85) a synonym of *Valvata piscinalis* (Müller, 1774). However, Küster cited Risso (1826: Pl. 3: fig. 30), where *Helix pulchella* Müller, 1774, currently *Vallonia pulchella* (Müller, 1774), was figured. Accordingly, *Vallonia rosalia* Risso, 1826 remains as the type species of *Vallonia* Risso, 1826, type genus of **Valloniidae**.

*Valvata rothi* Innes, 1884

Original source: Innes 1884: 347 (attributed to Bourguignat, but ICZN Art. 50.1.1 is not met).

Type localities: “Bords du lac Mariout, entre Ramleh et Alexandrie”, Egypt.

+ *Valvata rothleitneri* Bittner, 1884

Original source: Bittner 1884: 514, pl. 10: fig. 15.

Type horizon: Oligocene, Chattian.

Type locality: Trifail-Sagor (=Trbovlje-Zagorje), Slovenia.

+ *Valvata rugaoensis* Wang, 1977 (in Yü and Wang 1977)

Original source: Yü and Wang 1977: 15, pl. 1: fig. 14.

Type horizon: Upper Cretaceous.

Type locality: Jiangsu Province, China.

+ *Valvata sabaudiensis* Maillard, 1884

Original source: Maillard 1884: 68–69, pl. 2: figs 12–13a,b.

Type horizon: Jurassic – Purbeckian.

Type locality: Villers–le–lac, Département Doubs, France.

Remarks: According to Huckriede (1967: 168), fig. 12 of the original description of Maillard (1884) shows another species. Bandel (1991: 22, pl. 3: figs 16–20) considered this species belonging to *Provalvata* (**Provalvatidae**).

“*Valvata* var. *sabnaticina* Piaget, 1913” (ION, GNI)

Misspelling of *Valvata piscinalis* var. *subnaticina* Piaget, 1913.

*Valvata pulchella saghalinensis* Miyadi, 1935

Original source: Miyadi 1935: 61, pl. 3: fig. 3.

Type locality: “along the shore of a lakelet Tyatya–numa on the western coast of South Sakhalin”, Japan.

Holotype: According to Kantor et al. (2011: 70) deposited at Ôtsu Hydrobiological Station, now known as the Center for Ecological Research, Kyoto University.

+ *Valvata (Valvata) salebrosa* Meijer, 1990

Original source: Meijer 1990: 110, pl. 1: figs 3a–c.

Type horizon: Lower Pleistocene.

Type locality: Pit Maalbeek, Belfeld, province of Limburg, The Netherlands.

+ *Valvata salina* Leonard, 1972

Original source: Leonard 1972: 1–2, figs 1–3.

Type horizon: Pleistocene.

Type locality: Saline river banks near Equality, Galatin County, Southern Illinois, USA.

+ *Valvata (Turrivalvata) sarmatica* Papp, 1954

Original source: Papp 1954: 24, pl. 4: figs 3, 4a–b.

Type horizon: Middle Miocene, Sarmatian.

Type locality: Wiesen, Eisenstadt-Sopron Basin, Burgenland, Austria.

+ *Valvata satira* Fritzsche, 1924

Original source: Fritzsche 1924: 25, pl. 2: figs 8a, b.

Type horizon: Cretaceous.

Type locality: limestone of Yavi, North of province of Jujuy, Argentina.

Remarks: With a size of 12 mm and an oblique aperture this species is unlikely to be a valvatid.

*Valvata saulcyi* Bourguignat, 1853

Original source: Bourguignat 1853: 68, pl. 2: fig. 41, 42.

Type locality: near Damascus, Syria.

Remarks: It remains to be verified, whether the specimens identified as *Valvata saulcyi* reported from Sicily (e.g. Welter-Schultes 2012a: 45) truly is the same species.

+ *Valvata sayni* Delafond & Depéret, 1893

Original source: Delafond and Depéret 1893: 47, pl. 4: fig. 3, 3a.

Type horizon: Lower Pliocene.

Type locality: Marnes de Saint-Jean–de Vieux, Departément de Ain, France.

Remarks: Attributed to an unpublished manuscript by Fontannes (“Diagnos. esp. nouv. p, IV, fig. 10”), who mentioned *Valvata sayni* without description or figure as a nomen nudum (Fontannes 1883: 440), but did not make it available. Listed with several further references by Wenz (1928b: 2449).

“*Valvata scabrida*” mentioned in Meek and Hayden (1860: 418) (nomen nudum)

*Valvata scabrida* Meek & Hayden, 1865 (under subgenus *Tropidina*)

Original source: Meek and Hayden 1865: 113, pl. 4: figs 2a–b. Referred as *Amplovalvata scabrida* in Yen (1952: 39, pl. 6: figs 2a–o).

Type locality: Jurassic beds near the sout–west base of Black Hills, USA.

Holotype: U.S. National Museum #316 (see Yen 1952: 39).

*Valvata (Cincinna) piscinalis* var. *scharffi* Westerlund, 1894

Original source: Westerlund 1894: 198.

Type locality: Dublin, Ireland.

+ *Valvata (Valvata) schlosseri* Royo Gómez, 1922 (repeatedly erroneously cited in www, e.g. WMSDB for 1992)

Original source: Royo Gómez 1922: 159, textfig. 19, pl. 11: figs 17–20.

Type horizon: Miocene.

Type locality: surroundings of Teruel, Spain.

Remarks: Considered synonymous to = *Valvata* cf. *vallestris* Fontannes by Schlosser (1907: 24, pl. 1: fig. 22), other authors think it probably a **hydrobiid**, *Islamia schlosseri* or *Neohoratia schlosseri*, cf. Albesa and Robles (2006).

*Valvata schmidtii* Menke, 1849

Original source: Menke 1849: 166–167.

Type locality: near Töplitz (Toplice), Unterkrain (Dolenjska), Slovenia.

Remarks: Radoman (1985: 44) placed the species as *Sadleriana schmidtii* (Menke, 1849) (**Hydrobiidae**).

*Valvata schweinfurthi* Innes, 1884

Original source: Innes 1884: 352 (attributed to Bourguignat, but ICZN Art. 50.1.1 is not met).

Type locality: “Bords du lac Moeris”, Egypt.

*Valvata nilotica* var. *scioana* Pollonera, 1888

Original source: Pollonera 1888a: 82.

Type locality: Cimbisi district near Debra, Erhan, Ethiopia.

+ *Valvata semigradata* Pavlović, 1928

Original source: Pavlović 1928: 61.

Type horizon: Upper Miocene, Pannonian (Serbian).

Type locality: Karagača stream (Vrčin SSE Belgrade), Serbia.

*Valvata sequanica* Locard, 1883

Original source: Locard 1883b: 49, detailed description by Locard (1889: 18–19; 1893b: 124).

Type locality: Rouen, France.

+ *Valvata serbica* Brusina, 1902

Original source: Brusina 1902: pl. 13: figs 44–49.

Type horizon: Upper Miocene – Lower Pliocene.

Type locality: Visoka (Negotin), Serbia.

Remarks: Wenz (1928b: 2439) listed *Valvata serbica* Brusina, 1902 as a junior synonym of *Valvata minima* Fuchs, 1877, the latter is a junior homonym of *Valvata minima* Hislop, 1859.

+ *Valvata serpens* Stefanescu, 1896

Original source: “Sabba” Ştefănescu 1896: 8, 122ff, pl. 10: figs 139–144.

Type horizon: Pliocene – Pleistocene, Romanian.

Type locality: Milcov (“Un seul gisement connu, dans les couches levantines de Roumanie, à Milcov, près de Slaltina, dans la vallée de l’Oltu”), Romania.

Remarks: The author quoted himself as the author of taxa “Sabba”, but the last name on the title page is Stefanescu. Wenz (1928b: 2430) listed this name as a junior synonym of *Valvata crusitensis* Fontannes, 1886.

*Valvata servaini* Locard, 1889

Original source: Locard 1889: 15–16.

Type localities: Sur lees bordds des grands lacs ou étangs: La Maine à Angers; la Seine, à Marly, dans Seine-et-Oise; Argenteuil, près de paris; le délaissés de las Seine, près de Rouen; la canal de la Marne au Rhin; les environs de Lille, dans le Nord; les alluvions du Rhône, au nord de Lyon; le lac de la Négresse, près de Bayonne.

+ *Valvata (Cincinna) shakengensis* Yü & Zhang, 1982

Original source: Yü and Zhang 1982: 45, pl. 1: fig. 13–15.

Type horizon: Eocene.

Type locality: Zhuo Xian, Hebei, China.

+ *Valvata (Cincinna) shansiensis* Yü, 1965

Original source: Yü 1965: 45–46, pl. 1: figs 1–7.

Type horizon: Middle – Upper Eocene.

Type locality: upper part of the Yuanchü Chun, Yuanchü, Shansi, China.

“*Valvata shanxiensis* Yu“ mentioned in Yü (1984: 328)

Misspelling of + *Valvata shansiensis* Yü, 1965.

+ *Valvata sibinensis* Neumayr, 1875

Original source: Neumayr and Paul 1875: 78, pl. 9: figs 19a–d.

Type horizon: Pliocene – Pleistocene, Dacian-Romanian (*Paludina*–layer).

Type locality: Gromačnik, Croatia.

*Valvata cristata* var. *sibirica* Middendorff, 1851

Original source: Middendorff 1851: 299.

Type locality: Barnaul, Altai (on the basis of the lectotype), Western Siberia, Russia.

Lectotype was designated by Prozorova and Starobogatov (1986) and deposited in the Zoological Institute of the Russian Academy of Sciences, St. Petersburg.

Remarks: Falkner et al. (2001) and Glöer (2002) considered *Valvata sibirica* Middendorf, 1851 and *Valvata frigida* Westerlund, 1873 to be conspecific. Genital details were provided by Sitnikova (1983: fig. 1/3).

+ *Valvata sichuanensis* Yü, Pan & Wang, 1974

Original source: Yü et al. 1974: 325, pl. 169: figs 9–10.

Type horizon: Upper Triassic.

Type locality: Sichuan Emei lotus leaf bend, Sichuan Province, China.

Holotype at: http://159.226.74.248:8000/viewSpeciDetailsNew.jsp?bbbh=22758

Remarks: In the Chinese online–catalogues listed as *V.s.* Pian & Wang, 1975 or *V.s.* Yu, Pian & Wang, 1975.

+ *Cyclostoma simile* Draparnaud, 1805

Original source: Draparnaud 1805: 34, pl. 1: fig. 15.

Type horizon: ??

Type locality: France.

Remarks: Cited as *Valvata simile* by Férussac and Férussac (1807: 128, nr. 2 resp. Nr. 164), and also often (incorrectly) cited as *Valvata simile* Daudebard, 1807.

*Valvata tricarinata* var. *simplex* Gould, 1841 (non *Valvata simplex* Fuchs, 1870)

Original source: Gould 1841: 226, fig. 156 (right hand figure).

Type locality: Vermont, North-Eastern USA.

+ *Valvata simplex* Fuchs, 1870 (non *Valvata tricarinata* var. *simplex* Gould, 1841)

Original source: Fuchs 1870b: 535, Taf. 21: figs 4–6.

Type horizon: Upper Miocene, Pannonian (Transdanubian).

Type locality: near Tihany at Lake Balaton, Hungary.

Remarks: Bandel (2010: 105, pl. 8: figs 95–98) was unaware of the junior homonymy and classified the species as *Muellerpalia simplex* (Fuchs, 1870) (**Hydrobiidae**).

*Valvata piscinalis simusyuensis* Miyadi, 1935

Original source: Miyadi 1935: 60–61, pl. 3: fig. 2.

Type locality (from lectotype): Kitabettobu–numa (a lake), “Shumshir Island” [Kurile Islands], Russia.

Lectotype (“neoholotype”) and paratypes selected from the syntypes by Fujita and Habe (1991: figs 5–6) and deposited at Ôtsu Hydrobiological Station, now known as the Center for Ecological Research, Kyoto University (Kantor et al. 2011: 70).

*Valvata sincera* Say, 1824

Original source: Say 1824: 264, pl. 15: fig. 11.

Type locality: North–west Territory, Canada.

Type specimens: lost (Walker 1906).

Remarks: Vernacular names: “mossy valvata” or “boreal turret snail”.

“*Valvata sinensis* Yü et Lee“ mentioned in Yü (1979: 191, Abstract) (nomen nudum)

+ *Valvata sinensis* Yü & Lee, 1983 (in Yü 1983; author names spelled according to Pan 1983: 211 and online type catalogue of Nanjing Institute)

Original source: Yü 1983: 338, pl. 1: figs 15–17.

Type horizon: Upper Cretaceous.

Type locality: Songliao Basin, China.

Holotype data: http://159.226.74.248:8000/searchbycontentnew.jsp?curPage=1&showdetail=1&szlm=%3Ci%3EValvata%20sinensis%20%3C/i%3EYu%20et%20Lee,%201983

+ *Cincinna (Atropidina) singularis* Gozhik, 2007

Original source: Gozhik 2007: 79, pl. 69: figs 4–7.

Type horizon: Miocene, Upper Sarmatian.

Type locality: near the village Michailowka, district of Wolgograd, Russia.

*Cincinna (Sibirovalvata) sirotskii* Starobogatov & Zatravkin, 1985

Original source: Starobogatov and Zatravkin 1985: 1157: fig. 2.

Type locality: Near Novyj Mir settlement, Komsomolskij district, Khabarovsk Territory (Far East), depth 0.2 m, Russia.

Holotype: Zoological Institute of the Russian Academy of Sciences, St. Petersburg, Nr. 1 in systematic catalogue under the name.

+ *Valvata (Aphanotylus) skhiadica* Bukowski, 1895

Original source: Bukowski 1895: 24, 28–31 (description), 32, 34, 63, pl. 8: figs 9–11.

Type horizon: Upper Miocene (*Paludina*–layer).

Type locality: near Monastery Skhiadi, Rhodus Island, Greece.

Remarks: Lectotype designated by Willmann (1981: 76, textfig. 22)

*Valvata (Cincinna) skorikovi* Lindholm, 1912

Original source: Lindholm 1912: 299–300.

Type locality: 5 stations in “Newabucht bei Kronstadt”, near St. Petersburg, Russia.

Syntype: Zoological Institute of the Russian Academy of Sciences, St. Petersburg, Nr. 1 in systematic catalogue under the name.

*Valvata depressa* var. *soluta* Boettger, 1883

Original source: Boettger 1883: 343.

Type locality: near Athens, Greece.

+ *Valvata (Cincinna) soceni* Jekelius, 1944: 117, pl. 43: fig. 11–13.

Original source: Jekelius 1944: 117, pl. 43: fig. 11–13.

Type horizon: Middle Miocene, Sarmatian.

Type locality: Soceni (Banat), Romania.

*Valvata (Cincinna) sorensis* Dybowski, 1886

Original source: W. Dybowski 1886: 113–118, pl. 4: figs 1a–b (shell), 1^a^ (operculum), 3a, a’, b, c, d (radula), 5 (spawn).

Type locality: Posolskyj sor (eastern coast of Baikal), Russia.

Lectotype designated by Sitnikova et al. (2004) and deposited in Zoological Institute of the Russian Academy of Sciences, St. Petersburg.

+ *Valvata procera spatiosa* Pierce, 2001 (in Pierce and Constenius 2001)

Original source: Pierce and Constenius 2001: 64, figs 14L-14N.

Type horizon: Oligocene.

Type locality: Northern Kishenehn Basin, Montana and British Columbia, Canada.

*Valvata spelaea* Hauffen, 1856

Original source: Hauffen 1856b: 702, pl. 7: fig. 2.

Type locality: Cave of Glaven, Slovenia.

Remarks: Falkner et al. (2001) considered the taxon as a junior synonym of *Bythinella opaca* (M. von Gallenstein, 1848) (**Hydrobiidae**).

*Nerita sphaerica* Müller, 1774

Original source: Müller 1774: 170–171 (Nr. 356).

Type locality: Not directly provided, but probably Denmark, since a Danish vernacular name (kugle neriten) is given by Müller (1774: 170).

Remarks: According to Küster (1852–1853: 84) a synonym of *Valvata contorta* Müller, 1774.

*Valvata spirorbis* Draparnaud, 1805

Original source: Draparnaud 1805: 41, pl. 1: figs 32–33.

Type locality: France.

Types not traced. Possibly, in Natural History Museum Vienna (fide Dance 1986).

Remarks: Sometimes (see e.g. for “*Valvata andrezowski*”) regarded as synonym to *Valvata cristata* Müller, 1774, but considered as separate species by recent Russian authors, e.g. Anistratenko and Chernogorenko (1989), Anistratenko and Anistratenko (2001: 135), or Kantor et al. (2011: 74).

“*Valvata ssorensis*” (GNI, ION)

Misspelled for *Valvata sorensis* Dybowski, 1886, probably based on *Valvata (Cincinna) ssorensis* [sic!] var. *abbreviata*: Lindholm 1909, with a long tradition among Russian authors based on Lindholm’s papers.

*Valvata stelleri* Dybowski, 1903

Original source: Dybowski 1903: 46, textfig. 2–3.

Type locality: Lake Chalaktir, Kamtschatka, Russia.

Remarks: Kantor et al. (2011: 66) considered this taxon as synonym of *Valvata confusa* Westerlund, 1897.

*Valvata (Cincinna) stenotrema* Poliński, 1929

Original source: Poliński 1929: 135.

Type locality: Lake Ohrid, Macedonia.

Remarks: Anatomy (Rath 1986: 98ff) and molecular data (Hauswald et al. 2008) confirmed the valvatid nature of this taxon.

“*Valvata stenotrenta* “(GNI), found also at GBIF_Portal for Lund-Museum L934/3816

Misspelling of *Valvata (Cincinna) stenotrema* Poliński, 1929.

+ *Valvata stevanovici* Ilyina (Iljina in GNI and ION), 1982 (in Stevanovich and Ilyina 1982)

Original source: Stevanovich and Ilyina 1982: 127–128, pl. 3: figs 6a–c.

Type horizon: Miocene.

Type locality: exact location ?? (data in Cyril / Serbian), eastern Serbia.

+ *Valvata stiriaca* [sic] Rolle, 1860

Original source: Rolle 1860: 34, pl. 2: figs 9–10.

Type horizon: Lower Pliocene, lignit (brown coal) layers.

Type locality: Schallthal, basin of Schönstein (now Šoštanj), “Lower Styria”, Slovenia.

*Valvata stoliczkana* Nevill, 1878

Original source: Nevill 1878: 12, pl. 1: figs 34–36. Already mentioned as nomen nudum in Nevill (1877: 20).

Type locality: Yarkand, Xinjiang Uyghur autonomous region, China.

*Valvata strebeli* Fischer & Crosse, 1891

Original source: Fischer and Crosse 1880–1902 (Livraison 12, feuille 38: 1891): 304–305.

Type locality: “l’Etat du Mexico” = Estado Libre y Soberano de México, México.

Remarks: Replacement name for *Valvata humeralis* Strebel, 1873 (non Say, 1824).

*Valvata striata* Philippi, 1836 (non Lewis, 1856)

Original source: Philippi 1836: 147, pl. 8: fig. 3a–c;

Type locality: off Sicily, Mediterranean Sea.

Remarks: A marine species and according to its anatomy (Fretter 1956) classified as *Circulus striatus* in **Vitrinellidae** or **Tornidae**, although species identity in Fretter’s paper appears doubtful.

*Valvata striata* Lewis, 1856 (in Lea 1856) (non Philippi, 1836)

Original source: Lea 1856: 260. Contents were entirely attributed to Lewis, so Lewis’s authorship is correct under ICZN Art. 50.1.1.

Type locality: “Little Lakes”, Otsego County, New York, USA (Lea 1856: 259).

Remarks: Name preoccupied and being replaced by *Valvata lewisi* Currier, 1868 (see there).

+ *Valvata striolata* Pavlović, 1928

Original source: Pavlović 1928: 61.

Type horizon: Upper Miocene, Pannonian (Serbian).

Type locality: Karagača stream (Vrčin, SSE Belgrade), Serbia.

*Valvata studeri* Boeters & Falkner, 1998

Original source: Boeters and Falkner 1998: 115, textfigs 1–4 (sketches of shell and head), pl. 14: figs 2–5 (shells), pl. 16: figs 13–14 (spawn).

Type locality: Schützing (nature reservate “Untere Alz”), South Bavaria, Germany.

Holotype: Senckenberg-Museum Frankfurt SMF 311193.

Remarks: Introduced as a replacement name for *Valvata pulchella* sensu Studer (1820), but in fact a new species (see for *Valvata pulchella* Studer, 1789). Live photo by G. Falkner in Strong et al. (2008: 156).

*Valvata subangulata* Boettger, 1909 (in Wohlberedt 1909: authorship according to ICZN Art. 50.1.1)

Original source: Wohlberedt 1909: 697 (issue of journal) resp. 113 (in reprint), pl. 54: fig. 193.

Type localities: (1) River Zem near Angesta, (2) Bokumirska jezero = Lake Bukumirska (Podogorika), both Montenegro.

+ *Amplovalvata subantiqua* Yakushina (ION: Jakuschina), 1991

Original source: Yakushina 1991: 66, pl. 2: fig. 8.

Type horizon: Lower Cretaceous.

Type locality: North Choibalsan region, Mongolia.

+ *Valvata subbiformis* Gozhik, 1978 (in Gozhik and Prysjazhnjuk 1978)

Original source: Gozhik and Prysjazhnjuk 1978: 63, pl. 36: figs 22–24.

Type horizon: Middle or Upper Miocene, Sarmatian.

Type locality: Mykhailivka (= Michailovka = Mihalpol), Ukraine.

+ *Valvata subcarinata* Brusina, 1878

Original source: Brusina 1878: 352. Figured by Porumbaru (1881: 39, pl. 9: fig. 10), Brusina (1897: 25, pl. 13: fig. 32–39), and by Miloshevich (1984: pl. 1: figs 8–10).

Type horizon: Pliocene – Pleistocene, Dacian-Romanian.

Type locality (Neumayr 1869): Černik (=St. Leonhardt), near Nova Gradiska, Croatia.

Remarks: Replacement name for *Valvata piscinalis* as used by Neumayr (1869: 378, pl. 13: fig. 11).

“*Valvata subdepressa*“ mentioned in Bielz (1864: 78) (nomen nudum with locality)

Locality: Upper Miocene – Lower Pliocene, Pontian (“schwarzer Thon”, *Congeria*–layers); Krajova, Walachei (Tara Românească), Romania.

“*Valvata subfasciata*” (GNI)

Probably confused with *Vivipara subfasciata* Bourguignat, 1870.

*Valvata contorta* var. *subglobosa* Menke, 1845

Original source: Menke 1845: 116.

Type localities: “in lacubus Daniae, Galliae, Helvetiae (Hartmann), Germaniae; in Borussiae provinciae Brandenburgu lacu Ruppinensi (Feldmann, Martini), lac Müggelsee, ad Berlolinum (Ehrenberg), Vratislaviam (Scholtz), in Hannoverae lacu Seeburgensis (W. Dunker)”.

Types: Not traced. Menke’s collection was dispersed after his death (Dance 1986). Besides Menke’s types also the cited specimens of the bibliographically given sources were syntypes.

+ *Valvata subgradata* Lörenthey, 1902

Original source: Lörenthey 1902: 283, pl. 20: figs 9a–c.

Type horizon: Upper Miocene, Pannonian (Transdanubian).

Type locality: Budapest – Köbánya, Hungary.

*Valvata piscinalis* var. *submucronata* Schmidt, 1856

Original source: Schmidt 1856: 160. Without description, but with bibliographic reference to Stein (1850: pl. 2: fig. 28; legend at page 125 as *Valvata piscinalis*), thus available under ICZN Art. 12.2.1.

Type locality: Creek of Godesberg near Bonn, Germany.

+ *Valvata subnaticina* Lomnicki, 1886 (not *Valvata piscinalis* var. *subnaticina* Piaget, 1913)

Original source: Lomnicki 1886: 423.

Type horizon: Middle Miocene, Badenian.

Type locality: Goncharivka (= Wyczólki), Ukraine.

Remarks: “...der lebenden *Valvata naticina* Menke ähnlich” [similar to the extant *Valvata naticina* Menke] and thus probably belonging to genus *Borysthenia*.

*Valvata piscinalis* var. *subnaticina* Piaget, 1913 (not *Valvata subnaticina* Lomnicki, 1886)

Original source: Piaget 1913: 87, figs 6, 9.

Type locality: River Areuse, Canton Neuchatel, Switzerland.

Remarks: A junior homonym of + *Valvata subnaticina* Lomnicki, 1886.

*Valvata contorta* var. *subovata* Menke, 1845

Original source: Menke 1845: 115–116.

Type localities: The name was based on a description by Menke and on various bibliographical references, in many of which localities were given. All these are type localities.

Types: Not traced. Menke’s collection was dispersed after his death (Dance 1986). Besides Menke’s types also the cited specimens of the bibliographically given sources were syntypes.

“*Valvata subparvula*“ mentioned in Cossmann (1921: 170 (footnote).

Remarks: Cossmann (1921: 170, first line and footnote) refers to *Valvata parvula* as listed by Meek and Hayden (1876: 591) and thus later than *Valvat parvula* Deshayes, 1862 – which is not the case, however. *Valvata subparvula* is not an available name.

Locality: Tertiary; 3 miles below Fort Union, Nebraska, USA.

“*Valvata piscinalis* var. *subpiscinalis* Tournouër, 1866” mentioned in Wenz (1928b: 2435)

An error by Wenz, the name is not mentioned in the cited paper (Bull. Soc. Geol. France ser. 2, Vol. 23: page 790), but Delafond and Depéret (1893: 152) referred the name to specimens of the collection of Tournouër.

“*Valvata inflata* var. *subpiscinalis*” mentioned in Tardy (1883: 571) (nomen nudum).

+ *Valvata inflata* var. *subpiscinalis* Delafond & Depéret, 1893

Original source: Delafond and Depéret 1893: 87 (name only with locality), 152 (description), pl. 9: figs 49–51.

Type locality: Middle Pliocene; Saint-Amour, Département Jura, Region Franche-Comté, France.

Remarks: The name is referred to specimens of the collection of Tournouër.

*Valvata subpiscinalis* Kuščer, 1932

Original source: Kuščer 1932: 51–53, pl. 5: fig. 1.

Type locality: “Der Rak-Bach unweit der jugoslavisch–italienischen Grenze” [the rak–creek near the Jugoslavian-Italian border], Slovenia.

Paratypes (Biological Institute, Scientific Research Centre of Ljublijana, No 1862) were figured by Arconada and Ramos (2006: 78).

Remarks: Type species of *Neohoratia* Schütt, 1961 (**Hydrobiidae**), see Kabat and Hershler (1993: 38), Bodon et al. (2001: 141), and Callot-Girardi and Girardi (2013).

+ *Cincinna (Atropidina) subpulchella* Gozhik, 2007

Original source: Gozhik 2007: 78, pl. 69: fig. 3.

Type horizon: Pleistocene or Holocene (Alluvium V. Terrace).

Type locality: river Danube near the village Nagornoye, district of Odessa, Ukraine.

+ *Liratina subtilostriata* Pan, 1980 (in Yü and Pan 1980)

Original source: Yü and Pan 1980: 149, figs ?? (not seen, listed in Zoological Record 1980(A2): #3883).

Type horizon: Mesozoic?

Type locality: Zhuji same Shan, province Zhejiang, China.

Holotype: http://159.226.74.248:8000/viewSpeciDetailsNormal.jsp?bbbh=36281

+ *Planorbis subumbilicatus* Meek & Hayden, 1856

Original source: Meek and Hayden 1856: 120. Cited as *Valvata subumbilicata* in Meek and Hayden (1876: 590, pl. 43: fig. 13a–c).

Type horizon: Lower Palaeocene, Thanétien.

Type locality: 3 miles below Fort Union, Nebraska, USA.

*Cincinna sujfunensis* Prozorova, 1998 (in Prozorova and Starobogatov 1998)

Original source: Prozorova and Starobogatov 1998: 65, 67, fig. 4H.

Type locality: Razdolnaya River near Razdolnoye settlement, Far East Russia.

Holotype: Zoological Institute of the Russian Academy of Sciences, St. Petersburg, Nr. 1 in systematic catalogue under the name.

*Valvata sulcata* Eydoux & Souleyet, 1852

Original source: Eydoux and Souleyet 1852, 2: 547, pl. 31: figs 19–21.

Type locality: Pondichéry = Puducherry, South–east India.

Remarks: Vernacular name “Valvée sillonnée”. Considered as *Bithynia sulcata* (**Bithyniidae**) since Gray (1855: 20).

“*Valvata sulechiana* Cob. non Brus.”, cited at page 53 in Senoner (Vienna) Cenni Biograpfici. Giornale di Scienze Naturali il Naturalisti Siciliano, 1884–85, vol. 4: pp. 45–60.

Misspelling of + *Valvata sulekiana* Cobălcescu, 1883, non Brusina, 1874 (see there).

+ *Valvata sulekiana* Brusina, 1874 (non Cobălcescu, 1883)

Original source: Brusina 1874: 89, pl. 6: figs 11–12. Also figured by Stefanescu (1896: 123, pl. 10: figs 145–149) and Wenz (1942: 42, pl. 11: figs 136–138).

Type horizon: Pliocene-Pleistocene, Dacian-Romanian.

Type locality: Marinac (near Varoš), Slavonia, Croatia.

+ *Valvata sulekiana* Cobălcescu, 1883 (non Brusina, 1874)

Original source: Cobălcescu 1883: 142, pl. 13, fig. 18.

Type horizon: Pliocene–Pleistocene, Dacian–Romanian; *Paludina*–layers.

Type locality: Barboși (= Barboschi), Galati, Romania.

Remarks: An objective junior homonym of *Valvata sulekiana* Brusina, 1874 and thus replaced by *Valvata cobalcescui* Brusina, 1885 (see there).

+ *Valvata tricarinata supracarinata* Baker, 1921

Original source: Baker 1921: 24.

Type horizon: Pleistocene.

Type locality: near Morris, Grundy County, Illinois, USA.

Topotype: Museum of Natural History of the Univesity of Illinois, #P928.

*Valvata (Tropidina) macrostoma* var. *suturalis* Westerlund, 1886 (non *Valvata suturalis* Grabau, 1923)

Original source: Westerlund 1886: 140.

Type locality: Galicia near Przemysi: Kotula, Southeast Poland.

+ *Valvata suturalis* Grabau, 1923 (non *Valvata (Tropidina) macrostoma* var. *suturalis* Westerlund, 1886)

Original source: Grabau 1923: 161, pl. 2: figs 7e–g.

Type horizon: Upper Jurassic, Meng-Yin formation.

Type locality: Ning Chia Kou, Shandong, China.

+ *Planorbis symmetricus* Ludwig, 1865

Original source: Ludwig 1865: 96, pl. 21: figs 16, 16a, 16b. Cited as *Valvata symmetrica* (Ludwig) in Wenz (1928b: 2478).

Type horizon: Lower Miocene, Aquitanien.

Type locality: Kleinkarben in Hessen, Germany.

*Valvata syracusana* Locard, 1889: 35.

Original source: Locard 1889: 35 (attributed to Bourguignat; but ICZN Art. 50.1.1 is not satisfied).

Type locality: Assapo near Syracus in Sicily, Italy.

“*Valvata syriaca*“ mentioned in Innes (1884: 347) (nomen nudum with localities)

Localities: “… environs de Sayda en Syrie, a été constatée en Égypte sur les bords du lac Mariout; dans un marias à l’est de la Mahmoudieh; dans le lac du jardin khedivial de Ghizeh; sur les bords du lac Moeris, au Fayoun, et sur les rives de l’ancien lac Timsah.”, all Egypt.

Remarks: Innes (1884: 347) refers to “Bourguignat, Spec. Moll. n^o^. 191, 1878”.

However, as outlined by Connolly (1934), the latter works was never published. Innes (1884) presented with various localities (Syria, Egypt), he did not provide a description.

*Valvata tacitiana* Locard, 1889: 42.

Original source: Locard 1889: 42 (attributed to Letourneux, but ICZN Art. 50.1.1 is not met).

Type locality: Marshes of Cressida, Corfu, Greece.

*Valvata tasmanica* Tennison Woods, 1876

Original source: Tennison Woods 1876: 82+ (additional note).

Type locality: Gould’s County, north–easternTasmania.

Remarks: Type species of *Valvatasma* Iredale, 1943 (p. 203, **Hydrobiidae**), see Kabat and Hershler (1993: 56). *Beddomeia tasmanica* (Tennison-Woods, 1876) in the OBIS Indo-Pacific Database http://clade.ansp.org/obis/search.php/4996

“*Valvata tasolana*” (GNI, ION)

As already noticed by Kobelt (1893: 20) this taxon is misspelled for *Valvata tolosana* Saint-Simon, 1870 (the latter taxon is lacking in GNI and ION; Sept. 2013).

*Valvata (Pseudomegalovalvata) tenagobia* Bekman & Starobogatov, 1975

Original source: Bekman and Starobogatov 1975: 93, fig. 1A.

Type locality: Kharin-Irgi Bay (Oikhon Gates) [Baikal Lake], depth 32–39 m, Russia.

“*Valvata (Ohridotropidina) relicta relicta* f. *tetracarinata*“ mentioned in Hadžišče (1956: 62–63, fig. 3).

Locality: Lake Ohrid, Macedonia.

Remarks: Originally proposed at infrasubspecific rank, thus not available under ICZN Art. 45.6.4. However, the name would be available, if an author before 1985 used it and gave it subspecific or specific rank (ICZN Art. 45.6.4.1). This was not fully checked and remains to be verified.

+ *Valvata tenuistriata* Fuchs, 1870

Original source: Fuchs 1870b: 536, pl. 21: figs 19–20.

Type horizon: Upper Miocene, Pannonian (Transdanubian).

Type locality: near Tihany at Lake Balaton, Hungary.

Remarks: Classified by Bandel (2010: 107, pl. 10: figs 120–125) as *Jekeliella* (**Hydrobiidae**).

“*Valvata theocleti*” mentioned in Siodiropoulou (2003: 36–37, fig. 13).

Locality: Pliocene – Pleistocene; Ptolemaida, West-Macedonia, Greece.

Remarks: This PhD–Thesis PhD–Thesis, in which 35 species are ostendibley described, does not meet the conditions of ICZN Art. 8.1.3 and 9.9. Accordingly, the name is not available.

*Valvata theotokii* Locard, 1889

Original source: Locard 1889: 42 (attributed to Letourneux, but ICZN Art. 50.1.1 is not met).

Type localities: (1) Fountain of Kardachi, (2) Marshes of Cressida, both Korfu, Greece.

+ *Valvata tihanyensis* Lörenthey, 1906

Original source: Lörenthey 1906: 171, pl. 1: figs 18–19.

Type horizon: Upper Miocene, Pannonian (Transdanubian).

Type locality: near Tihany at Lake Balaton, Hungary.

*Valvata tilhoi* Germain, 1909

Original source: Germain 1909: 376.

Type locality: Lake Chad, Algerie.

*Valvata tolosana* Saint-Simon, 1870

Original source: Saint-Simon 1870: 31–33.

Type locality: Toulouse, channel of Midi, France.

+ *Valvata (Valvata) toplicani* Miloshevich, 1984

Original source: Miloshevich 1984: 177, pl. 1: figs 41–47.

Type horizon: Pliocene−Pleistocene, Metohija Series.

Type locality: Topličane, Metohija basin, Kosovo.

+ *Valvata tournoueri* Capellini, 1880

Original source: Capellini 1880: 410–411, pl. 5: figs 7–12.

Type horizon: Upper Miocene (*Congeria*–layers).

Type locality: valley of Sterza di Laiatico, province of Pisa, Italy.

+ *Valvata transbaicalensis* Martinson, 1961

Original source: Martinson 1961: 244–245, pl. 22: figs 4–5.

Type horizon: Lower Cretaceous.

Type locality: exact locality??, Eastern Sibiria, Russia.

*Cyclostoma tricarinata* Say, 1817

Original source: Say 1817: 13, cited as *Valvata tricarinata* by Say (1821: 173).

Type locality: river Delaware, USA.

Remarks: Verancular name: “three–ridge valvata”. Anatomy, histology and development of the genital system were outlined by Furrow (1931, 1935). Live photo at: http://animaldiversity.ummz.umich.edu/site/accounts/information/Valvatidae.html

*Helix tricarinata* Megerle von Mühlfeld, 1824 (not Müller, 1774)

Original source: Megerle von Mühlfeld 1824: 220, pl. 8 resp. 2: figs 9a,b. “...da diese Schnecke zur Gattung *Valvata* des Draparnaud gehört...” [...since this snail belongs to genus *Valvata* of Draparnaud....].

Type locality: beach of Rimini (shells transported by rivers), Italy.

Remarks: According to figures identical (personal judgement) to *Helix nana* Megerle von Mühlfeld, 1824 (see there) and thus probably a synonym of *Valvata cristata* Müller, 1774.

+ *Valvata trigeri* Deshayes, 1862

Original source: Deshayes 1861–1863, Vol. 2: 525, pl. 36: figs 9–11.

Type horizon: Middle Eocene, Bartonian.

Type locality: Nantheuil–sur-Marne, Saint Aubin, pres le Mans, Basin de Paris, France.

*Valvata contorta* var. *trochoidea* Menke, 1845

Original source: Menke 1845: 116.

Type localities: The name was based on a description by Menke and on various bibliographical references, in many of which localities were given. All these are type localities.

Types: Not traced. Menke’s collection was dispersed after his death (Dance 1986). Besides Menke’s types also the cited specimens of the bibliographically given sources are syntypes.

“*Valvata troglobia*“ mentioned in Bole and Velkovrh (1986)

Remarks: According to Bodon et al. (2001: 177) mismatched because of the misleading title of Piersanti (1952: Una nuova specie italica di *Valvata* troglobia, *Valvata pusilla*, Mihi.), a nomen nudum.

+ *Valvata trouessarti* Brusina, 1902

Original source: Brusina 1902: pl. 13: figs 28–30.

Type horizon: Pliocene-Pleistocene, Romanian (*Paludina*–layers).

Type locality: Szentes, Hungary.

+ *Valvata truckeensis* Yen, 1950

Original source: Yen 1950b: 185, pl. 1: fig. 3.

Type horizon: Miocene, Truckee formation.

Type locality: Desert Queen Mine, northeastern corner of the Hot Spring Mountains, Western Nevada, USA.

+ *Valvata truncatella* Li, 1984

Original source: Li 1984: 7, pl. 1: figs 20–22.

Type horizon: Lower Tertiary.

Type locality: Lingboa Basin of Henan Province, China.

*Valvata umbilicata* var. *tubula* Westerlund, 1886

Original source: Westerlund 1886: 140.

Type locality: Siberia, Russia.

+ *Valvata tuostaiensis* Wei, 1984

Original source: Xinjiang Dizhi Ju 1984: 84, figs ? (not seen, title in Zoological Record 126(9): #3985).

Type horizon: ??

Type locality: Xinjiang Province, China.

+ *Valvata tuozhuangensis* Youluo, 1978 (name of author according to the online type catalogues of Nanjing Institute)

Original source: Youluo 1978: 31–32, pl. 4: figs 21–23.

Type horizon: Lower Tertiary.

Type locality: coastal region of Bohai, China.

+ *Valvata (Cincinna) turgensis* Martinson, 1961

Original source: Martinson 1961: 245, pl. 22: figs 1–3. Also cited and figured in Zhu 1994: 93, pl. 2: figs 13–19.

Type horizon: Lower Cretaceous.

Type locality: not clear, since Martinson only listed several records: West Trans-Baikal region – Tarbagatay, Ulan-Ude Region, Kizhinga Region (Buryatia); Ost Trans-Baikal Region – Arbagar, Turga; Vilyuysk, Baysa; south–east Mongolia.

*Valvata turgidula* Locard, 1889

Original source: Locard 1889: 53 (attributed to Bouguignat, but ICZN Art. 50.1.1 is not satisfied).

Type locality: Lac de Négresse, Bayonne, Pyrénées-Atlantiques, France.

Remarks: Probably synonymous to *Valvata minuta*, now *Islamia globulina* (see Bodon et al. (2001: 177, 202).

“*Valvata (Atropidina) turislavica*“ mentioned in Floriu (2011: 32).

Probably confused with + *Caspia turislavica* Jekelius, 1944.

+ *Atropidina turpanensis* Zhu, 1994

Original source: Zhu 1994: 96 (Chinese) / 103 (English), pl. 1, figs 7–12.

Type horizon: Middle Jurassic, Qiktim Formation.

Type locality: Xiabakan, Shanshan County, Turpan Basin, Northern Xinjiang, China.

Holotype: http://www.nimrf.net.cn/ept/eptDataDetail.action?ptzyh=2332C0001000004184

*Cincinna (Sibirovalvata) tymiensis* Starobogatov, 1985 (in Starobogatov and Zatravkin 1985)

Original source: Starobogatov and Zatravkin 1985: 1157: fig. 3.

Type locality: Sakhalin Island, right bank of the River Tym’ near Nogliki settlement, Russia.

Holotype: Zoological Institute of the Russian Academy of Sciences, St. Petersburg, Nr. 1 under the name.

“*Valvata umbilicata*” mentioned in Fitzinger (1833: 117) (nomen nudum with locality)

Locality: Gosau-See, Upper Austria.

*Valvata umbilicata* Servain, 1881: 94

Original source: Servain 1881: 94 (attributed to “Parreyss mss.”, but ICZN Art. 50.1.1 is not satisfied).

Type locality: “dans la vallée du Siô dans les endroits marécagenx”, Lake Balaton, Hungary.

Remarks: It is beyond the scope of this contribution to check, whether the specimens of Servain (1881) are identical to those of Westerlund (1886) – if this is possible at all on shells alone. It is possible that Fitzinger communicated the name to Parreyss, and Parreyss labelled the name, and Servain thought that the name must be attributed to Parreyss.

*Valvata umbilicata* Westerlund, 1886

Original source: Westerlund 1886: 140. Cited and figured first by Rossmässler and Kobelt (1910: 15, pl. 399, fig. 2289) with bibliographic reference to Westerlund (1886).

Type localities: various countries between Sweden and Austria.

Remarks: Westerlund expressively attributed the name to the nomen nudum of Fitzinger (1833), which is not available, however. Accordingly, the naming by Servain (1881) has priority, if these are two different species.

*Valvata unicarinata* De Kay, 1843 (non Lörenthey, 1894)

Original source: De Kay 1843: 118–119, pl. 6: fig. 129a–b.

Type locality: Lake Champlain and Erie Channel (New York), USA.

+ *Valvata unicarinata* Lörenthey, 1894 (non De Key, 1843)

Original source: Lörenthey 1894: 120 (50), pl. 5: fig. 1.

Type horizon: Upper Miocene – Pliocene, Pannonian (Portaferrian).

Type locality: Séd–creek, Bálint–bridge, Szekszárd, Hungary.

Remarks: a junior homonoym of *Valvata unicarinata* De Kay, 1843.

+ *Valvata unicarinifera* Hislop, 1859

Original source: Hislop 1859: 170, pl. 5: fig. 14.

Type horizon: Tertiary.

Type locality: Little Tisti, Karwad, Butárá, East India.

+ *Valvata simplex* var. *unicincta* Lörenthey, 1906

Original source: Lörenthey 1906: 165, pl. 1: fig. 16.

Type horizon: Upper Miocene, Pannonian (Transdanubian).

Type locality: Fehérpart near Tihany, Hungary.

*Valvata sincera* var. *utahensis* Call, 1884

Original source: Call 1884: 22, 24, 25, 44, pl. 6: figs 1–3.

Type locality: Utah Lake, USA.

Holotype: U.S. National Museum # 31277.

Remarks: Vernacular names: Utah roundmouth snail, desert valvata. Hovingh (2004) stated that *Valvata utahensis* is a polymorphic species exhibiting a wide range of forms. Miller et al. (2006) and Hauswald et al. (2008) provided DNA-sequences for unequivocal identification.

+ *Amplovalvata valareslebensis* Huckriede, 1967

Original source: Huckriede 1967: 169, pl. 23: figs 29a–31c.

Type horizon: Upper Jurassic, Middle Kimmeridgian.

Type locality: southeast of Sülfeld, Fallersleben, Germany.

+ *Valvata vallestris* Fontannes, 1876

Original source: Fontannes 1876: 52, pl. 1: fig. 3.

Type horizon: Upper Miocene.

Type locality: Moulin de Fully near Saint-Quentin-Fallavier (= La Fuly); Bas Dauphine, southeast of Lyon, France.

Remarks: “*Valvata* cf. *vallestris* Fontannes“ mentioned in Schlosser (1907: 24, pl. 1: fig. 22) has been named *Valvata (Valvata) schlosseri*, Royo Gómez 1922 (see there).

“*Valvata (Valvata) valvestris* Fontannes” by Locard (1878: 62).

Misspelling of + *Valvata vallestris* Fontannes, 1876.

+ *Valvata (Tropidina) vanciana* Tournouër, 1875

Original source: Tournouër 1875: 742 (name only), 744–746 (description), pl. 28: figs 3–4.

Type horizon: Upper Miocene.

Type locality: Fort de Vancia near Lyon, Département de Ain, France.

+ *Valvata variabilis* Fuchs, 1870

Original source: Fuchs 1870a: 346, pl. 14: fig. 10–12, 17–19.

Type horizon: Upper Miocene, Pannonian (Transdanubian).

Type locality: Rădmănești, near Lugos in Banat, Romania.

Remarks: Considered as *Muellerpalia varians* (Fuchs, 1870) (**Hydrobiidae**) by Bandel (2010: 104, pl. 8: figs 90–94).

+ *Valvata varians* Lörenthey, 1902

Original source: Lörenthey 1902: 281, pl. 20: figs 6–8.

Type horizon: Upper Miocene, Pannonian (Transdanubian).

Type locality: Budapest – Köbánya; Hungary.

+ *Pachystoma varicatum* Tausch, 1886

Original source: Tausch 1886: 13, pl. 2: figs 6a–d, 7a–b, 8a–b.

Type horizon: Upper Cretaceous – “Gosaumergel”.

Type locality: Csinger valley near Ajka in Bakony, Hungary.

Remarks: Type species of *Ariomphalus* Bandel & Riedel, 1994 (p. 22), who also figured the shell and protoconch by SEM (23, pl. 14: figs 1–3).

+ *Valvata sabaudiensis* var. *varicosa* Koert, 1898

Original source: Koert 1898: 42, lower textfigure.

Type horizon: Jurassic/Cretaceous border.

Type locality: southwest of Selter hill, Lower Sachsian, Germany.

“*Valvata (Tropidina) vauciana*“

Misspelling of + *Valvata (Tropidina) vanciana* Tournouër, 1875 in the catalogue of the Museum National d’Histoire Naturelle (Paris) at http://coldb.mnhn.fr/ScientificName/Valvata/vauciana

+ *Valvata (Atropidina) velitzelosi* Schütt & Velitzelos, 1991

Original source: Schütt and Velitzelos 1991: 5–6, pl. 2: fig. 22.

Type horizon: Upper Miocene.

Type locality: Kerasia, Island of Euboea, Greece.

+ *Cincinna vetusta* Kormos, 1911

Original source: Kormos 1911b: ?? (not seen).

Type horizon: Upper Miocene, Pannonian (Transdanubian).

Type locality: Mencshely, Lake Balaton, Hungary.

+ *Cincinna (Cincinna) vinogradovskense* [sic] Gozhik, 2002 [should be *vinogradovskensis*, since *Cincinna* is feminine]

Original source: Gozhik 2002: 48, pl. 2: fig. 15–17. Also figured by Gozhik (2007: pl. 66: figs 1–3).

Type horizon: Miocene, Pontian.

Type locality: Vinograd, Ukraina.

*Valvata virens* Tryon, 1863

Original source: Tryon 1863: 148, pl. 1: fig. 2.

Type locality: Clear Lake, California, USA.

Remarks: Vernacular name: “emerald valvata”. A recent species considered to be extinct by human activities (pollution), however.

“*Valvata viridana* Stentz”

Cited (with doubts) by Strobel (1851: 98) and by Gredler 1859: 250 (#144) but unknown, probably another shell–dealer name (see above for *Valvata impura*).

+ *Valvata (Aegaea) vivipariformis* Oppenheim, 1891

Original source: Oppenheim 1891: 462–463, pl. 26: fig. 1, 1a–c.

Type horizon: Lower Pleistocene.

Type locality: Koumaris (= Kumari) near Aegion, Greece.

Remarks: According to the size (10 mm) and aperture (not round) probably not a valvatid, but a species of **Viviparidae** (certainly not a “missing link” between the two families as assumed by Oppenheim, since both families are very distantly related). Type species of *Aegaea* Oppenheim, 1891.

“*Valvata viviparum*”

Cited by Liess and Hillebrandt (2005) and Liess et al. (2006) from Lake Erken (Sweden), but most probably confused with *Viviparus viviparus* (Linnaeus, 1758).

*Valvata (Microcincinna) vystitiensis* Chernogorenko & Starobogatov, 1987

Original source: Chernogorenko and Starobogatov 1987: 148.

Type locality: Vyshtitis Lake, at the border of Kaliningrad district and Lithuania.

Holotype: Zoological Institute of the Russian Academy of Sciences, St. Petersburg, Nr. 1 in systematic catalogue under the name.

+ *Valvata vrabceana* Gorjanović-Kramberger, 1890

Original source: Gorjanović-Kramberger 1890: 156, pl. 6: fig. 8.

Type horizon: Upper Miocene, Pannonian (Slavonian).

Type locality: Vrapče (Zagreb), Croatia.

*Valvata wagneri* Kuščer, 1928

Original source: Kuščer 1928: 50, fig. 1.

Type locality: “Vranja peč” cave near Boštanj, Svenica, Krso, Slovenia.

Remarks: Type species of *Vrania* Radoman, 1978: 35 (**Hydrobiidae**), see Binder (1967), Kabat and Hershler (1993: 57), and Bodon et al. (2001: 154, figs 163–160).

*Valvata perdepressa walkeri* Baker, 1930

Original source: Baker 1930: 188

Type locality: Southern part of Lake Michigan, USA.

+ *Valvata (Atropidina) wenzi* Papp, 1953

Original source: Papp 1953: 110, pl. 4: figs 4–5.

Type horizon: Upper Miocene, Pannonian.

Type locality: Eichkogel bei Mödling, Lower Austria.

Remarks: SEM of shell and (typically valvatid) protoconch were depicted by Harzhauser and Binder (2004: 11, pl. 3: figs 7–8).

+ *Valvata whitei* Hannibal, 1910

Original source: Hannibal 1910: 107.

Type horizon: Marl–deposit, Upper Lahontan Quaternary.

Type locality: near Summer Lake, Oregon, USA.

+ *Aphanotylus whitei* Dall, 1924

Original source: Dall 1924: 115, pl. 26: fig. 7.

Type horizon: Pliocene – Pleistocene, Idaho Formation.

Type locality: Castle Creek, Owyhee County, Idaho, USA.

+ *Valvata (Turrivalvata) soceni wiesenensis* Papp, 1954

Original source: Papp 1954: 25, pl. 3: figs 23–24.

Type horizon: Middle Miocene – Sarmatian.

Type locality: Wiesen, Eisenstadt-Sopron Basin; Burgenland, Austria.

+ *Valvata windhauseni* Parodiz, 1961

Original source: Parodiz 1961: 16–18, pl. 1: figs 1–6.

Type horizon: Lower Tertiary.

Type locality: Nahuel Niyeu (25 miles west of Valvheta), Rio Negro province, Argentina.

*Valvata winnebagoensis* Baker, 1928.

Original source: Baker 1928: 475–476, pl.1: figs 11–13.

Type locality: Miller Bay, Lake Winnebago, Wisconsin, USA.

Remarks: Vernacular name: “flanged valvata”.

+ *Valvata woodwardi* Kennard, 1911

Original source: Kennard 1911: 324–325, 1 textfig.

Type horizon: Pleistocene, Cromerian Stage.

Type locality: West Runton, Norfolk, England.

Remarks: Considered as a synonym of *Valvata goldfussiana* Wüst, 1901 by Wüst (1912).

+ *Valvata yaviana* Fritzsche, 1924

Original source: Fritzsche 1924: 23, pl. 2: figs 7a–c.

Type horizon: Cretaceous.

Type locality: limestone of Yavi, North of province of Jujuy, Argentina.

+ *Valvata yongkangensis* Yü, 1980

Original source: Yü and Pan 1980: 147, figs ?? (not seen, listed in Zoological Record 1980(A2): #3883).

Type horizon: Mesozoic?

Type locality: Zhejiang, southern Anhui, China.

Holotype: http://159.226.74.248:8000/viewSpeciDetailsNormal.jsp?bbbh=18572

+ *Valvata piligera yukonensis* Clarke & Harington, 1978

Original source: Clarke and Harington 1978: 47, fig. 2A–D.

Type horizon: Pleistocene.

Type locality: Old Crow Basin, Yukon Territory, Canada.

+ *Sinorificium yumenensis* Guo, 1982 (in Guo et al. 1982)

Original source: Guo et al. 1982: 34, pl. 13: figs 9–14.

Type horizon: Lower Cretaceous.

Type locality: Shaanxi, Gansu and Ningxia, North–west China.

Remarks: Currently regarded (Paleobiology Database) as a neotaenioglossan species.

+ *Valvata zhongbaensis* Yü, 1974

Original source: Yü 1974: 372, pl. 198: figs 18–19.

Type horizon: Lower Jurassic.

Type locality: Sichuan Jiangyou, China.

+ *Valvata zhongjiangensis* Pan, 1982

Original source: Pan 1982: ??, figs 18–21 (not seen, not in Zoological Record, data from online type catalogue of Nanjing Institute).

Type horizon: Upper Jurassic, Penglaizhen formation.

Type locality: Zhongjiang County Cangshan, Sichuan Basin, China.

Holotype: http://159.226.74.248:8000/viewSpeciDetailsNormal.jsp?bbbh=53434

+ *Valvata zhouqingzhuangensis* Youluo, 1978 (name of author according to the online type catalogues of Nanjing Institute)

Original source: Youluo 1978: 28–29, pl. 4, figs 24–25.

Type horizon: Lower Tertiary.

Type locality: coastal region of Bohai, China.

+ *Valvata (Cincinna) zhuchengensis* Pan, 1983

Original source: Pan 1983: 213, pl. 1: figs 7–9.

Type horizon: Lower Cretaceous – Xiazhuang Formation.

Type locality: Shichang-Zhonglou Basin in North China.

+ *Valvata zschokkei* Bollinger, 1921

Original source: Bollinger 1921: 8, textfig. 2.

Type horizon: Pleistocene, Interglacial “Schieferkohle”.

Type locality: Dürnten, Kanton Zürich, Switzerland.

## Remarks

If authors were originally written in Cyril lettering or Chinese wording I either use the Latin version found in the original paper or (sometime several) the Latin version(s) found in the later literature citing that paper. Usually the original title and an English or French translation of the title or journal are provided.

As far as possible I provide here direct www-links to the first page of the respective papers and books. Mostly they have been found in the Biodiversity Library respectively at www.archive.org , via Gallica http://gallica.bnf.fr/ respectively in the Jubilothece http://jubilotheque.upmc.fr/, and in the digital library “DigiLit” of the Oberösterreichisches Landesmuseum Linz (please, provide an English introduction for international users!) at <http://www.landesmuseum.at/datenbanken/digilit/>, reflecting the extremely high value and help provided by these institutions. On the other hand the substantial content of the digital library of the Haiti Trust http://catalog.hathitrust.org is of very limited use, since it is available only for few US-colleagues. It is particularly hard to understand, why contributions prior to 1923 cannot be offered online “due to copyright restrictions”.

If not stated otherwise these e-libraries are free for download or view. This also is true for Google-books, where registration, but no fee is required. Note that the digital page numbers often do not correspond with the printed page numbers. All Roman numbers of pre-pages or plates are listed in Arabic numbers for convenience.

I add here the urgent ask for all responsible persons and societies for journals or books not yet digitized to do so as soon as possible at least for the elder volumes before 1923 (no copyright restrictions) in order to facilitate and accelerate taxonomy as science. Concerning the present work the “most wanted” journals are the *Archiv für Molluskenkunde* and the *Mitteilungen der Deutschen Malakozoologischen Gesellschaft*. In addition, I (and the whole malacological community as well) will be grateful for any tip concerning a new or overlooked source of electronic copy of any reference not yet covered herein.

## Final remarks

Despite much time and effort (and the truly substantial input by all the reviewers), the present contribution is neither complete nor entirely free of errors. The main reason for these shortcomings is the non-availability of certain papers, in particular those of Russian and Chinese paleontologists. Accordingly, for a future update I ask all readers and colleagues to send me corrections, additions (e.g. sources of type material), and (preferably electronic) copies of papers not yet or insufficiently indexed.

## References

[B1] Abbott RT (1949) An Indian species of *Clenchiella*. The Nautilus 63(2): 62 http://archive.org/stream/nautilus63amer#page/62/mode/2up

[B2] Adami GB (1881) Molluschi postpliocenici della Torbiera di Polada presso Lonato. Bullettino della Società Malacologica Italiana 7: 18–202 (no pls.) http://archive.org/stream/bullettino07soci#page/188/mode/2up

[B3] Adams CB (1849–52) Contributions to Conchology. Vol. 1. (1849) Descriptions of supposed new species of freshwater shells which inhabit Jamaica. H. Baillière, New York, 129–140. http://archive.org/stream/contributionstoc00adam#page/128/mode/2up

[B4] Adams H Adams A (1854) The genera of recent Mollusca: arranged according to their organization. 3 Volumes. J. Van Voorst, London, 40 + 484 pp. http://www.biodiversitylibrary.org/bibliography/4772

[B5] Albesa J Robles F (2006) Síntesis de los estudios sobre moluscos continentales neógenos del sector septentrional de la Depresión de Teruel: período 1775–1998. Estudios Geológicos 62(1): 183–198. http://estudiosgeol.revistas.csic.es/index.php/estudiosgeol/article/view/18/17

[B6] Almera J (1894) Memoria sobre los depósitos pliocénicos de la cuenca del bajo Llobregat y llano de Barcelona. Memorias de la Real Academia de Ciencias y Artes de Barcelona / Memòries de la Reial Academia de Ciencies i Arts de Barcelona, epoca Tercera, Vol. III (segunda parte: Paleontologia) 3(2): 355 pp., 7 pls http://archive.org/stream/descripcindelos00angegoog#page/n9/mode/2up (text); http://archive.org/stream/descripcindelos00angegoog#page/n373/mode/2up (plates)

[B7] Almera J Bofill Y Poch A (1892) Catálogo de los Moluscos fósiles Pliocenos de Cataluña. Publicaciones de la Crónica científica de Barcelona 1892, Barcelona, 108 pp. http://simurg.bibliotecas.csic.es/viewer/image/CSIC000074873/1/

[B8] Altimira C (1960) Notas malacológicas. Contribución al conocimiento de los moluscos terrestres y de agua dulce de Cataluña. Miscelánea Zoologica 1(3): 9–15. www.raco.cat/index.php/Mzoologica/article/download/93528/168699

[B9] Andreae A (1884) Ein Beitrag zur Kenntnis des Elsässer Tertiärs. Abhandlungen zur geologischen Specialkarte von Elsass-Lothringen 2(3), R. Schultz & Co., Strassburg, 331 pp., 12 pls. http://archive.org/stream/abhandlungenzur04lotgoog#page/n365/mode/2up (text only, plates lacking)

[B10] Anistratenko OYa Anistratenko VV (2012) Зоогеография и экология среднесарматских гастропод Восточного Паратетиса [Zoogeography and ecology of the Middle Sarmatian Gastropods of the Eastern Paratethys]. Ruthenica 22(2): 115–134. [in Russian, English summary] www.ruthenica.com/documents/Vol22_Anistratenko_Anistratenko_115-134.pdf

[B11] Anistratenko OYa Degtyarenko E Anistratenko VV (2010) Сравнительная морфология раковины и радулы брюхоногих моллюсков семейства Valvatidae из Северного Причерноморья [Shell and radula comparative morphology of the gastropod mollusc family Valvatidae from the North Black Sea coast]. Ruthenica 20(2): 91–101. [in Russian, Engl summary] www.ruthenica.com/documents/Vol20_Anistratenko_etal_91-101.pdf

[B12] Anistratenko VV (1998) Определитель гребнежаберных моллюсков (Gastropoda, Pectinibranchia) фауны Украины. Часть 2. Пресноводные и наземные [Guide to pectinibranch molluscs (Gastropoda, Pectinibranchia) of the fauna of Ukraine. Part 2. Freshwater and land species]. Vestnik Zoologii, Supplement 8: 67–117. [in Russian]

[B13] Anistratenko VV Anistratenko OYa (2001) Фауна Украины в сорока томах. Том 29. Моллюски. Выпуск 1. Книга 1. Класс панцирные или хитоны, класс брюхоногие [Fauna Ukraine: Vol. 29: Mollusca. Fasc. 1, book 1: Class Polyplacophora or Chitons, Class Gastropoda - Cyclobranchia, Scutibranchia i Pectinibranchia taschast]. National Academy of Sciences of the Ukraine, Institute of Zoology, Kiev 29(1): 1–240. [in Russian, English abstract]

[B14] Anistratenko VV Chernogorenko EV (1989) Фауна и экология брюхоногих моллюсков бассейна Среднего Днепра [Fauna and ecology of gastropod mollusks of the middle Dnieper river basin Ukrainian SSR USSR]. Vestnik Zoologii 1989(2): 3–6. [in Russian]

[B15] Arconada B Ramos MA (2006) Revision of the genus *Islamia* Radoman, 1973 (Gastropoda, Caenogastropoda, Hydrobiidae), on the Iberian Peninsula and description of two new genera and three new species. Malacologia 48(1–2): 77–132. http://archive.org/stream/malacologia482006inst#page/76/mode/2up

[B16] Arkell WJ (1941) The gastropods of the Purbeck Beds. The Quarterly Journal of the Geological Society of London 97: 79–128. http://jgslegacy.lyellcollection.org/content/97/1-4.toc (free abstract, pdf-download requires subscription)

[B17] Baily JLjr Baily RI (1951) Further observations on the Mollusca of the relict lakes in the Great Basin. The Nautilus 65(2): 46–53, pl. 4. http://archive.org/stream/nautilus65amer#page/46/mode/2up

[B18] Baird W (1850) Nomenclature of molluscous animals and shells in the collection of the British Museum. Pt. I. Cyclophoridae. London, by the Trusties, 69 pp. http://archive.org/stream/nomenclatureofmo00brit#page/n1/mode/2up

[B19] Baker FC (1921) New forms of Pleistocene Mollusks from Ilinois. The Nautilus 35: 22–24. http://archive.org/stream/nautilus35amer#page/22/mode/2up

[B20] Baker FC (1928a) The Fresh Water Mollusca of Wisconsin. Part I. Gastropoda. Wisconsin Geological, Natural History Survey Bulletin 70: 20 + 507 pp. + 28 pls. http://digicoll.library.wisc.edu/cgi-bin/EcoNatRes/EcoNatRes-idx?id=EcoNatRes.WGB70PartI

[B21] Baker FC (1928b) Descriptions of new varieties of land and freshwater mollusks from Pleistocene deposits in Illinois. The Nautilus 41: 132–137. http://archive.org/stream/nautilus41amer#page/132/mode/2up

[B22] Baker FC (1930) The molluscan fauna of the southern part of Lake Michigan and its relationship to old glacial Lake Chicago. Transactions of the Illinois State Academy of Science 22: 186–194. http://www.idaillinois.org/cdm/compoundobject/collection/isac/id/6675/rec/1

[B23] Baker FC (1931) Description of a new variety of *Valvata lewisi* Currier. The Nautilus 44: 119–121. http://archive.org/stream/nautilus44amer#page/118/mode/2up

[B24] Baker HB (1961) *Lustrica* (*Paludina*) Say, 1821 (Gastropoda): proposed suppression under the Plenary Powers. Z.N.(S.) 730. Bulletin of Zoological Nomenclature 18(1–3): 146–148. http://biostor.org/reference/2218

[B25] Bandel K (1991) Gastropods from brackish and fresh water of the Jurassic - Cretaceous transition (a systematic reevaluation). Berliner geowissenschaftliche Abhandlungen (A) 134: 9–55, 7 pls. (pdf-file available on request from the author)

[B26] Bandel K (2010) Valvatiform Gastropoda (Heterostropha and Caenogastropoda) from the Paratethys Basin compared to living relatives, with description of several new genera and species. Freiberger Forschungshefte C536: 91–155. (pdf-file available on request from the author)

[B27] Bandel K Riedel F (1994) The Late Cretaceous gastropod fauna from Ajka (Bakony Mountains, Hungary): a revision. Annalen des Naturhistorischen Museums Wien 96A: 1–65. www.landesmuseum.at/pdf_frei_remote/ANNA_96A_0001-0065.pdf

[B28] Bank RA (1989) Die Veröffentlichungsdaten der Rossmässler‘schen‚ Iconographie der Land- und Süßwasser-Mollusken’ Europas (1835–1920). Mitteilungen der Deutschen Malakozoologischen Gesellschaft 44/45: 49–53.

[B29] Bank RA (2013) *Islamia pusilla* (Piersanti, 1951). Fauna Europaea. http://www.faunaeur.org/full_results.php?id=427808 [accessed July 2013]

[B30] Bank RA Menkhorst HPMG (2009) A revised bibliography of the malacological papers of Paul Pallary. Zoologische Mededelingen Leiden 83(5): 537–546. http://dpc.uba.uva.nl/cgi/t/text/get-pdf?c=zoomed;idno=8303a05

[B31] Bartha F Soós L (1955) Die pliozäne Molluskenfauna von Balatonszentgyorgy. Annales historico-naturalis Musei nationalis hungarici, (new series 6) 47: 51–72, pls. 4–5 http://publication.nhmus.hu/pdf/annHNHM/Annals_HNHM_1955_Vol_47_51.pdf

[B32] Bech i Taberner M Altimiras i Roset J (2003) Nuevas aportaciones al conocimiento de los moluscos actuales y del cuaternario en Extremadura: I. Malacofauna dulceacuícola. Revista de estudios extremeños 59(2): 837–870. www.dip-badajoz.es/cultura/ceex/reex_digital/reex_LIX/2003/T.%20LIX%20n.%202%202003%20mayo-ag/RV11435.pdf

[B33] Beck H (1847) Verzeichniss einer Sammlung von Landconchylien aus den Dänischen Staaten in Europa, bestehend aus 2,058 Individuen, darstellend 158 Arten, die zu 44 Geschlechtern gehören, eingesandt in Folge Allerhöchsten Befehls zur Versammlung Deutscher Naturforscher und Aerzte in Kiel im Jahre 1846 aus dem Königlichen particulären zoologischen Musäum. Amtlicher Bericht über die Versammlung Deutscher Naturforscher und Ärzte 24: 122–124. http://archive.org/stream/amtlicherbericht23gese#page/122/mode/2up

[B34] Behrendt GM (1863) Die Diluvial- Ablagerungen der Mark Brandenburg, insbesondere der Umgegend von Potsdam. Mittler, Sohn, Berlin, 85 pp., 1 pl., 1 map https://play.google.com/store/books/details?id=-NcAAAAAcAAJ&rdid=book--NcAAAAAcAAJ&rdot=1 (requires registration at google-books)

[B35] Bekman / Beckman MYu Starobogatov YaI (1975) Байкальские глубоководные моллюски и родственные им формы. В кн.: Новое о фауне Байкала. Часть 1. [Baikalian deep-water molluscs and forms related to them. In: New data on the fauna of Baikal. Part I.] Trudy Limnologicheskogo Instituta SO AN SSSR [Travaux de la Station limnologique du Lac Bajkal / Transactions of the Limnological Station, Siberian Branch of the USSR Academy of Sciences] 18(38): 92–111. [in Russian].

[B36] Benoist E-A (1873) Catalogue synonymique et raisonné des testacés fossiles des faluns miocènes de la Brède et de Saucats (2 parts). Actes de la Société Linnéenne de Bordeaux 29: 5–78, 265–452. http://archive.org/stream/actesdelasocit29soci#page/n9/mode/2up (Part 1); http://archive.org/stream/actesdelasocit29soci#page/n289/mode/2up (Part 2)

[B37] Benoit L (1857) Illustrazione sistematica critica iconografica de’ testacei estramarini della Sicilia ulteriore e delle isole circostanti. Cav. Gaetano Nobile, Napoli, 16 + 248 pp., 12 pls. http://archive.org/stream/illustrazionesis00beno#page/n5/mode/2up (text) http://archive.org/stream/illustrazionesis00beno#page/248/mode/2up (plates)

[B38] Berge F (1847) Conchylienbuch oder allgemeine und besondere Naturgeschichte der Muscheln und Schnecken: nebst der Anweisung, sie zu sammeln, zuzubereiten und aufzubewahren. 263 pp., 46 pls. http://archive.org/stream/conchylienbuchod00berg#page/n5/mode/2up (new edition of 1855)

[B39] Bernard F (1888) Recherches anatomiques sur la *Valvata piscinalis*. Comptes rendus de l’ Academie des Sciences, Paris 107: 191–194. http://archive.org/stream/comptesrendusheb1071888acad#page/190/mode/2up

[B40] Bernard F (1890) Recherches sur *Valvata piscinalis*. Bulletin scientifique de la France et de la Belgique 22: 253–361, pls. 12–20. http://archive.org/stream/bulletinbiologiq22univ#page/252/mode/2up (text); http://archive.org/stream/bulletinbiologiq22univ#page/n453/mode/2up (plates)

[B41] Berry EG (1943) The Amnicolidae of Michigan. Miscellaneous Publications. Museum of Zoology, University of Michigan 57: 68 pp., 9 pls. http://deepblue.lib.umich.edu/bitstream/handle/2027.42/56302/MP057.pdf?sequence=1

[B42] Bielz EA (1864) Die jungtertiären Schichten nächst Krajova in der Walachei. Verhandlungen und Mittheilungen des siebenbürgischen Vereins für Naturwissenschaften zu Hermannstadt 15: 76–78, 243–247. www.landesmuseum.at/pdf_frei_remote/VerhMittNaturwissHermannstadt_15_0076-0078.pdf

[B43] Binder E (1967a) Position systématique de *Valvata minuta* Drap., *Valvata globulina* Palad. et d’autres petetes espèces attribuées au genre *Valvata*. Atti della Società Italiana di Scienze Naturali e del Museo Civico di Storia Naturale di Milano 105(4): 371–376, 4 figs. (pdf-file available on request from the author)

[B44] Binder E (1967b) La coquille embryonaire des Valvatidae (Mollusca, Gastropoda). Archiv für Molluskenkunde 96: 21–24.

[B45] Bittner A (1884) Die Tertiär-Ablagerungen von Trifail und Sagor. Jahrbuch der kaiserlich königlichen Geologischen Reichsanstalt 34: 433–596, pl. 10 http://www.landesmuseum.at/pdf_frei_remote/JbGeolReichsanst_034_0433-0596.pdf; http://archive.org/stream/jahrbuchderka341884unse#page/432/mode/2up (text); http://archive.org/stream/jahrbuchderka341884unse#page/n639/mode/2up (plates)

[B46] Bland T (1865) Note on certain insect larvae described as species of Valvatidae. Annals of the Lyceum of Natural History of New York 8: 144–149. http://archive.org/stream/annalsoflyceumof81867lyce#page/144/mode/2up, doi: 10.1111/j.1749-6632.1867.tb00303.x

[B47] Bodon M Manganelli G Giusti F (2000) *Valvata minuta* Draparnaud, 1805 (currently *Hauffenia*, *Neohoratia* or *Islamia minuta*; Mollusca, Gastropoda): proposed replacement of the lectotype by a neotype. Bullettin of Zoological Nomenclature 57: 144–146. http://www.biodiversitylibrary.org/page/12439220#page/166/mode/2up

[B48] Bodon M Manganelli G Giusti F (2001) A survey of the European valvatiform hydrobiid genera, with specidal reference to *Hauffenia* Pollonera, 1898 (Gastropoda: Hydrobiidae). Malacologia 43(1–2): 103–215. http://archive.org/stream/malacologia432001inst#page/102/mode/2up

[B49] Bodon M Manganelli G Giusti F (2002) *Heraultiella* new name for *Heraultia* Bodon M., Manganelli G., Giusti F., 2001 (Gastropoda, Hydrobiidae). Journal of Conchology 37(6): 681.

[B50] Bole J Velkovrh F (1986) Mollusca from continental subterranean aquatic habitats. In: Botosaneuanu L (Ed) Stygofauna mundi. A faunistic, distributional and ecological synthesis of the world fauna inhabiting subterreanean waters (including the marine interstitial). Brill, Leiden 177–208.

[B51] Boeters H-D (1988) Westeuropäische Moitessieriidae und Hydrobiidae in Spanien und Portugal (Gastropoda: Prosobranchia). Archiv für Molluskenkunde 118: 181–261.

[B52] Boeters H-D Falkner G (1998) *Valvata pulchella* S. Studer and *Valvata studeri* n.sp. (Gastropoda, Ectobranchia: Valvatidae). Heldia 2(5/6): 113–122, pls. 14–16 (pdf-file available on request from the author)

[B53] Boettger O (1883) Aufzählung der von den Herren E. Reitter und E. Brenske 1882 in Griechenland und auf den Jonischen Inseln gesammelten Binnenmollusken. Jahrbücher der Deutschen Malakozoologischen Gesellschaft 10(4): 313–344, pl. 8 http://archive.org/stream/jahrbcherderde101883deut#page/312/mode/2up

[B54] Boettger O (1884) Fossile Binnenschnecken aus den untermiocänen Corbicula-Tonen von bei Niederrand. Berichte der Senckenbergischen Naturforschenden Gesellschaft im Frankfurt am Main 1884: 258–280. http://archive.org/stream/naturundmuseum1884senc#page/258/mode/2up

[B55] Boettger O (1905) Die Konchylien aus den Anspülungen des Sarus-Flusses bei Adana in Sizilien. Nachrichtsblatt der Deutschen Malakozoologischen Gesellschaft 37(3): 97–123, pl. 2a http://archive.org/stream/nachrichtsblattd3738190516deut#page/n121/mode/2up (text); http://archive.org/stream/nachrichtsblattd3738190516deut#page/n177/mode/2up (plate)

[B56] Bogatov VV Zatravkin MN (1992) (“1990“) Брюхоногие моллюски пресных и солоноватых вод Дальнего Востока СССР / Bryukhonogie mollyuski presnykh I solonovatykh vod Dalnego Vostoka SSSR [Fresh and brackish water gastropods of the Soviet Far East] Biologo-pochvennyi institut, Vladivostok / Academy of Science of the USSR, Vladivostok: Far East Branch, 172 pp. [in Russian]

[B57] Boissy S-A de (1846) Coquilles fossiles de Rilly (Marne). Bulletin de la Société géologique de France (2) 4: 177–179. http://jubilotheque.upmc.fr/img-viewer/fonds-bulsgf/GB_000018_001/Contenu/JPEG_HD/viewer.html?ns=GB_000018_001_J3_003.jpg

[B58] Boissy S-A de (1848) Description des coquilles fossiles du calcaire de Rilly-la- Montagne: prés de Reims. Mémoirs de la Société Géologique de France (2) 3(4): 266–285, pls. 5–6 http://goo.gl/ouQNQe

[B59] Bollinger G (1921) Mollusken aus der Schieferkohle von Dürnten. Festschrift zur Feier des 60. Geburtstages (27. Mai 1920) von Friedrich Zschokke Nr. 5 Basel, Kober, 17 pp..

[B60] Botosaneanu L Alkins-Koo M (1993) The caddisflies (Insecta: Trichoptera) of Trinidad and Tobago, West Indies. Bulletin de l’Institut Royal des Sciences Naturelles de Belgique, Entomologie 63: 5–45.

[B61] Bouchet P Rocroi J-P (2005) Classification and nomenclator of gastropod families. Malacologia 47(1–2): 1–397. http://archive.org/stream/malacologia47122005inst#page/n9/mode/2up

[B62] Bour R (2010) Constant Duméril’s Zoologie Analytique was published in 1805. Bionomina 1: 56–57. http://mapress.com/bionomina/content/2010/f/bn00001p057.pdf

[B63] Bourguignat JR (1853) Catalogue raisonné des mollusques terrestres et fluviatiles recueillis par M. F. de Saulcy pendant son voyage en Orient. Gide, J. Baudry, Paris, 26 + 96 pp., pls. 1–4 http://gallica.bnf.fr/ark:/12148/bpt6k99038p

[B64] Bourguignat JR (1853–1856) Aménités Malacologiques. Recueil de travaux et notes diverses publiés pour la plupart de 1853 à 1856 dans la Revue et Magazin de Zoologique, Vol. 1: 1–247, 21 pls. http://doc.rero.ch/record/11955/files/

[B65] Bourguignat JR (1859) Mélanges et Nouvelles (IV. Remarque sur la broschure de M. Tassinari 1858: Mollusci fluviatilis italici nova species). Revue et magasin de zoologie pure et appliquée (2) 11: 545–546. http://archive.org/stream/revueetmagasinde11revu#page/544/mode/2up

[B66] Bourguignat JR (1870a) Aperçu de la faune malacologique du bas Danube. Annales de Malacologie 1: 36–76. http://archive.org/stream/annalesdemalacol1187084serv#page/36/mode/2up

[B67] Bourguignat JR (1870b) Mollusques nouveaux, litigieux ou peux connus. Revue et magasine de Zoologie pure et appliquée (2) 22(5), 166–171, pl. 17 http://archive.org/stream/revueetmagasinde222pari#page/166/mode/2up (text); http://archive.org/stream/revueetmagasinde222pari#page/n362/mode/1up (plate)

[B68] Bourguignat JR (1877) [«1875–1876»] Descriptions de deux nouveaux genres algériens, suivies d’une classification des familles et des genres de mollusques terrestres et fluviatiles du système européen. Bulletin de la Société des Sciences Physiques et Naturelles de Toulouse 3(1): 49–101. www.animalbase.uni-goettingen.de/zooweb/servlet/AnimalBase/home/digireference?id=43

[B69] Bourguignat JR (1878) Species novissimae molluscorum in Europaeo systemati detectae, notis diagnosticis succinctis breviter descriptae. Lutetiae. 1–80. http://gallica.bnf.fr/ark:/12148/bpt6k99050j; http://archive.org/stream/speciesnovissima00bour#page/n15/mode/2up

[B70] Bourguignat JR (1880–1881) Matériaux pour servir à l’histoire des *Mollusques* acéphales du système européen. Imprimerie de S. Lejay, cie, Poissy, 1–387. http://archive.org/stream/matriauxpourse00bour#page/n7/mode/2up

[B71] Bourguignat JR (1881) Histoire malacologique de la colline de Sansan, précédée d’une notice géologique et suivie d’un aperçu climatologique et topographique de Sansan, à l’époque des dépôts de cette colline. Annales des sciences géologiques (6) 11: 49–175, pls. 26–33 http://gallica.bnf.fr/ark:/12148/bpt6k4325871.langDE; http://archive.org/stream/histoiremalacol00unkngoog#page/n4/mode/2up (text); http://archive.org/stream/histoiremalacol00unkngoog#page/n190/mode/2up (plates)

[B72] Bourguignat JR (1889) *Mollusques* de l’Afrique équatoriale de Moguedouchou à Bagamoyo et de Bagamoyo au Tanganika. DuMoulin et Co., Paris, 229 pp., 8 pls. http://archive.org/stream/mollusquesdelafr00bour#page/n5/mode/2up

[B73] Bourguignat JR (1891) Oevres Scientifiques. Du Moulin, Paris, 256 pp. http://archive.org/stream/oeuvresscientifi1891bour#page/n9/mode/2up

[B74] Bowler-Kelly A (1928) Un nouveau gisement paléolithique de la forêt de Compiègne (Oise). In: Bowleer-Kelly A Kelley H (Eds) Congres de la La Rochelle. Comptes Rendus de l’Association Française pour l’Avancement des Sciences 52: 453–454.

[B75] Branson CC (1935) Fresh-water invertebrates from the Morrison (Jurassic?) of Wyoming. Journal of Paleontology 9(6): 514–522, pls. 56–57. http://www.jstor.org/stable/i255578 (free abstract, download of pdf requires subscription)

[B76] Brard C-P (1815) Histoire des coquilles terrestres et fluviatiles qui vivent aux environs de Paris. J.J. Paschoud, Paris, 239 (text) + 17 (legends) pp., 10 pls. http://archive.org/stream/histoiredescoqui00brard#page/n7/mode/2up

[B77] Braun A (1843) Vergleichende Zusammenstellung- der lebenden und diluvialen Molluskenfauna des Rheinthals mit der Tertiären des Mainzer Beckens. Amtlicher Bericht über die 20. Versammlung der Naturforscher und Aerzte in Mainz 1842: 142–150. http://archive.org/stream/amtlicherbericht20gese#page/142/mode/2up

[B78] Bronn HG (1831) Ergebnisse meiner naturhistorisch-ökonomischen Reisen. Zweyter Theil. Skizzen und Ausarbeitungen über Italien. Nach einem zweyten Besuche im Jahre 1827 entworfen. Heidelberg, Leipzig (Groos). 18 + 686 pp., 4 pls. http://babel.hathitrust.org/cgi/pt?id=mdp.39015031288619;seq=7;view=1up

[B79] Brown DS (1965) Freshwater gastropod Mollusca from Ethiopia. Bulletin of the British Museum (Natural History), Zoology 12: 39–94, pls. 1–3 http://archive.org/stream/bulletinofbritis12zoollond#page/n55/mode/2up (text); http://archive.org/stream/bulletinofbritis12zoollond#page/n115/mode/2up (plates)

[B80] Brusina S (1870) Contribution à la malacologie de la Croatie. Zagreb/Agram, Imprimé aux frais de l’auteur par C. Albrech. 40 pp. http://archive.org/stream/contributionla00brus#page/n0/mode/2up

[B81] Brusina S (1874) Fossile Binnen-Mollusken aus Dalmatien, Kroatien und Slavonien nebst einem Anhange. Deutsche vermehrte Ausgabe der kroatischen im “Rad” der südslavischen Akademie der Wissenschaften und Künste in Agram (28. Band, 1874) erschienenen Abhandlung. Actienbuchdruckerei, Agram, 144 pp., 7 pls. http://archive.org/stream/fossilebinnenmo00brusgoog#page/n5/mode/2up

[B82] Brusina S (1878) Molluscorum fossilium species novae et emendatae, in tellure tertiana Dalmatiae, Croatiae et Slavoniae inventae. Journal de Conchyliologie 26(4): 347–356. http://archive.org/stream/journaldeconchyl261878pari#page/346/mode/2up

[B83] Brusina S (1885) Bemerkungen über rumänische Paludinen-Schichten mit Bezug auf Professor G. Cobalcescu’s Werk: „Studii geologice şi palæontologice asupră unor Těrámuri Terţiare din unilc Părţĭ ale Romăneĭ. Verhandlungen der kaiserlich königlichen Geologischen Reichsanstalt 6: 157–163. www.landesmuseum.at/pdf_frei_remote/VerhGeolBundesanstalt_1885_0157-0163.pdf

[B84] Brusina S (1892) Fauna fossile terziaria di Markuševec in Croazia. Glasnik Hrvatskoga naravoslovnoga Društva 7: 113–210. http://archive.org/stream/glasnikhrvatskog1892hrva#page/n129/mode/2up

[B85] Brusina S (1894) («1893») Note preliminaire sur le groupe des *Aphanotylus*, nouveau genre de Gastropode de l’horizon à *Lyrcæa*, et sur quelques autres espèces nouvelles de Hongrie. Journal de Conchyliologie 41: 179–187. (publication date was 12th March 1894 according to Winckworth 1936) http://archive.org/stream/journaldeconchyl411893pari#page/178/mode/2up

[B86] Brusina S (1897) Gragja za neogensku malakošku faunu Dalmacije, Hrvatske i Slavonije uz neke vrste iz Bosne, Hercegovine i Srbije / Matériaux pour la faune malacologique néogène de la Dalmatie, de la Croatie, et de la Slavonie avec des espèces de la Bosnie et de l’Herzégowine et de la Serbie (Croatian/French) Djela Jugoslavenske akademije znanosti i umjetnosti 18 Actienbuchdruckerei, Zagreb/Agram, 21 + 43 pp., 21 pls. http://archive.org/stream/djelajugoslaven03umjegoog#page/n6/mode/2up (text); http://archive.org/stream/djelajugoslaven03umjegoog#page/n76/mode/2up (plates are poorly scanned)

[B87] Brusina S (1902) Iconographia molluscorum fossilium in tellure Tertiari Hungariae, Croatiae, Slavoniae, Dalmatiae, Bosniae, Herzegovinae, Serbiae et Bulgarie inventorum. Operis Gragja pars altera. Dion. Tiskara, Zagreb/Agram, 10 pp. + 30 pls. (pdf-file available on request from the author)

[B88] Buch L (1837) [Note]. Neues Jahrbuch für Mineralogie, Geognosie, Geologie und Petrefaktenkunde 1837: 98 http://archive.org/stream/neuesjahrbuchfer1837stut#page/98/mode/2up

[B89] Bukowski G (1895) (1895 as reprint; 1896 as issue of journal) Die Levantinische Molluskenfauna der Insel Rhodus. 2. Teil und Schluss. Denkschriften der mathematisch-naturwissenschaftlichen Klasse der kaiserlichen Akademie der Wissenschaften in Wien 63: 70 pp., pls. 7–11 http://archive.org/stream/dielevantinische02buko#page/n7/mode/2up (text + plates); www.landesmuseum.at/pdf_frei_remote/DAKW_63_0001-0070.pdf

[B90] Bulić J Jurišić Z (2009) Macropalaeontology and stratigraphy of lacustrine Miocene deposits at Crnika beach on the Island of Pag (Croatia). Geologia Croatica 62(3): 135–155. http://hrcak.srce.hr/file/66756, doi: 10.4154/gc.2009.16

[B91] Calcara P (1842) Cenno topografico dei dintorni di Termini. Roberti, Palermo, 1–32. http://gdz.sub.uni-goettingen.de/dms/load/img/?PPN=PPN64022282X&IDDOC=620485

[B92] Call RE (1884) On the Quaternary and Recent Mollusca of the Great Basin, with descriptions of new forms. Bulletin of the United States Geological Survey 11: 358–421, 6 pls. https://play.google.com/store/books/details?id=VZaACOyXrQsC&rdid=book-VZaACOyXrQsC&rdot=1 (needs registration at google-books)

[B93] Callot-Girardi H Girardi M (2013) Complément à l’étude de *Corbullaria celtiberica* et présentation d’espèces valvatiformes d’Espagne, de France, d’Italie et de Slovénie (Mollusca: Gastropoda Prosobranchia: Hydrobiidae). Folia Conchyliologica 21: 3–30. http://www.cernuelle.com/download.php?lng=fr

[B94] Capellini G (1880) Gli strati a congerie o la formazione gessosa-solfifera nella provincia di Pisa e nei dintorni di Livorno. Atti della Reale Accademia dei Lincei, Memorie della Classe di Scienze Fisiche, Matematiche e Naturali, Roma (3) Annata 277, Vol. 5: 375–427, 9 pls. http://emeroteca.braidense.it/beic_attacc/sfoglia_articolo.php?IDTestata=924&CodScheda=00AF&IDT=31&IDV=269&IDF=0&IDA=8763 (djvu-format)

[B95] Carpenter HF (1889) Notes on *Valvata (Lyogyrus) brownii*. The Nautilus 3: 67 http://archive.org/stream/nautilus38990amer#page/66/mode/2up

[B96] Caziot E (1902) Étude sur la faune des mollusques vivants terrestres et fluviatiles de l’île de Corse. Bulletin de la Société historique et naturelles de la Corse 22(266–269): 354 pp., 2 pls. Ollagnier, Bastia http://gallica.bnf.fr/ark:/12148/bpt6k5725738n.image.langDE.r=Soci%C3%A9t%C3%A9%20des%20sciences%20historiques%20et%20naturelles%20de%20la%20Corse

[B97] Chaignon H de (1893) Note sur le forage de quelques puits en Bresse et sur quelques affleuremens fossilifères. Bulletin de la Société géologique de France (3) 11: 610–623, pl. 17 http://jubilotheque.upmc.fr/ead.html?id=GB_000056_001&c=GB_000056_001_page610#!{«content»:[«GB_000056_001_page625»,false,»»]}

[B98] Chernogorenko EV (1991) Переописание *Cincinna chersonica* (Gastropoda, Pectinibranchia) [A redescription of *Cincinna chersonica* (Gastropoda, Pectinibranchia)]. Vestnik Zoologii 1991(4): 81–83. [in Russian]

[B99] Chernogorenko EV Starobogatov YaI (1987) Valvatidae Восточнои Eвролы [Valvatidae of East Europe] In: Starobogatov YaI Golikov AN Likharev IM (Eds) Моллюски: результаты и перспективы их исследований : Восьмое Всесоюзное совещание по изучению моллюсков, Ленинград, апрель 1987 г.: авторефераты докладов. [Molluscs, Results and Perspectives of Investigation]. Eighth Meeting on the Investigation of Molluscs, Abstracts of communications. Zoological Institute, Academy of Sciences, Leningrad, 148–150. (pdf-files of original paper and of an English translation are available on request by the author) [in Russian]

[B100] Clarke AH Harington CR (1978) Asian freshwater mollusks from Pleistocene deposits in the Old Crow Basin, Yukon Territory. Canadian Journal of Earth Sciences 15(1): 45–51. http://www.nrcresearchpress.com/doi/pdf/10.1139/e78-004, doi: 10.1139/e78-004

[B101] Cleland DM (1954) A study of the habits of *Valvata piscinalis* (Müller) and the structure and function of the alimentary canal and reproductive system. Proceedings of the Malacological Society of London 30(6): 167–203. http://mollus.oxfordjournals.org/content/30/6.toc (free abstract, download of pdf requires subscription of the Journal of Molluscan Studies)

[B102] Clessin S (1877) Die Mollusken der Tiefenfauna unserer Alpenseen. Malakozoologische Blätter 24: 159–185, pl. 3 http://archive.org/stream/malakozoologisch2324menk#page/158/mode/2up (text); http://archive.org/stream/malakozoologisch2324menk#page/n475/mode/2up (plates)

[B103] Clessin S (1887) Die Mollusken-Fauna Mitteleuropa’s. II. Die Mollusken-Fauna Oesterreich-Ungarns und der Schweiz. Bauer, Raspe, Nürnberg, 858 pp. http://archive.org/stream/diemolluskenfaun22cles#page/n7/mode/2up

[B104] Cobălcescu G (1883) Studii geologice şi palæontologice asupră unor Tăràmuri Terţiare din unele părţĭ ale Româneĭ / Geological and paleontological studies on certain Tertiary lands in some parts of Romania [Romanian and French text in parallel columns] Memoriile Geologice ale Şcolei Militare din Iaşi, Memoriile I, Bucuresci. Stabilimentul Grafic Socecu, Teclu, 1–164, table of contents and corrections (2), pls. 1–18.

[B105] Cocchi I (1867) L’uomo fossile dell’Italia centrale, studi paleontologici. Memorie della Società Italiana di Scienze Naturali e del Museo Civico di Storia Naturale in Milano 2(7): 1–80, 4 pls. https://play.google.com/books/reader?id=3m4_AAAAcAAJ&printsec=frontcover&output=reader&authuser=0&hl=de&pg=GBS.PP1 (requires registration at google-books) 10.4081/nhs.2014.60 PMC4681098 26689358

[B106] Colbeau J (1859) Matériaux pour la faune malacologique de Belgique. I. Liste des mollusques terrestres et fluviatiles de Belgique. Bruxelles, chez l’auteur, 12 pp.

[B107] Colbeau J (1868) Liste générale des Mollusques vivants de la Belgique. Annales de la Société malacologique de Belgique 3: 85–111, pls. 2–4 http://archive.org/stream/annalesdelasoci031868soci#page/n87/mode/2up

[B108] Connolly M (1934) A Bourguignatian bluff. Proceedings of the Malacological Society of London 21(2): 70–73. http://mollus.oxfordjournals.org/content/21/2/70.full.pdf+html (free abstract, download of pdf requires subscription of the Journal of Molluscan Studies)

[B109] Contreras-Arquieta A (1993) *Valvata beltrani* n. sp. (Gastropoda: Valvatidae) de Charco Azul, San Juan de Aviles, Aramberri, Nuevo León, México. Publicaciones Biológicas, Facultad de Ciencias Biológicas, Universidad Autónoma de Nuevo León, Supl 1, 1–6, figs 1–2. (pdf-file available on request from the author)

[B110] Cooke AH (1895) Molluscs. In: Harmer SF Shipley AE (Eds) The Cambridge Natural History 3 Macmillan and Co., London, England, 11 + 462 pp. http://archive.org/stream/cambridgenatural03harm#page/n7/mode/2up

[B111] Cossmann M (1888) Catalogue illustré des coquilles fossiles de l’Éocène des environs de Paris. Annales de la Société royale malacologique de Belgique 23: 3–324, pls. 1–12 http://archive.org/stream/annalesdelasoci23soci#page/n31/mode/2up (text); http://archive.org/stream/annalesdelasoci23soci#page/n557/mode/2up (plates)

[B112] Cossmann M (1899a) Mollusques Èoceniques de la Loire-Inferieure. Tome 2. Bulletin de la Société des sciences naturelles de l’Ouest de la France 9: 307–360, pls. 22–26. http://archive.org/stream/bulletindelasocif09soci#page/n437/mode/2up (text); http://archive.org/stream/bulletindelasocif09soci#page/n419/mode/2up (plates)

[B113] Cossmann M (1899b) Sur la découverte d’un gisement palustre à Paludines dans le Terrain Bathonien de l’Indre. Bulletin de la Société Géologique de France (3) 27: 136–143. http://goo.gl/hf7qUR

[B114] Cossmann M (1907) Troisième note sur le Bathonien de Saint-Gaultier (Indre). Bulletin de la Société Géologique de France (4) 7: 225–253, pls. 7–8. http://goo.gl/XApLc9; http://rogov.zwz.ru/Joliaf/Cossmann,1907.pdf

[B115] Cossmann M (1918) Essais de Paléoconchologie comparée 11. Paris, chez l’auteur, 388 pp., 11 pls. http://archive.org/stream/essaisdepaloconc12coss#page/n11/mode/2up

[B116] Cossmann M (1919) Supplément aux Mollusques éocéniques de la Loire-Inférieure. Bulletin de la Société des Sciences Naturelles de l’Ouest de la France (3) 5: 53–141, pls. 1–4. http://archive.org/stream/bulletindelasocif0305soci#page/52/mode/2up (text); http://archive.org/stream/bulletindelasocif0305soci#page/n169/mode/2up (plates)

[B117] Cossmann M (1921) Essais de Paleoconchologie comparée 12. Paris, chez l’auteur, 348 pp., pls. A-D, 1–6 http://archive.org/stream/essaisdepaloconc12coss#page/n447/mode/2up

[B118] Cossmann M (1924) Revision des Scaphopodes, Gastropodes et Céphalopodes du Montien de Belgique (Deuxième Partie). Mémoires de la Museum Royal de l’Histoire Naturelle de Liège 34: 1–35, pls. 5–6 http://archive.org/stream/revisiondesscaph1112coss#page/n93/mode/2up

[B119] Cossmann M Pissarro G (1910–1913) Iconographie complète des coquilles fossiles de l’éocène des environs de Paris. Tome 2. Scaphopodes, Gastropodes, Brachiopodes, Céphalopodes. Supplement Paris (no publisher mentioned), 21 pp. + 65 pls. http://archive.org/stream/iconographiecomp212coss#page/n199/mode/2up

[B120] Coxe W (1789) Faunula Helvetica: Or, a catalogue of the Quadrupeds, Birds, Amphibia, Fishes, and Testaceous Animals of Switzerland. Travels in Switzerland 3 T. Cadell, London, 384–392. http://resolver.sub.uni-goettingen.de/purl?PPN251818438

[B121] Crosse H (1863) Description d’espèces nouvelles. Journal de Conchyliologie 11: 379–386, pl. 13 http://archive.org/stream/journaldeconchyl111863pari#page/378/mode/2up (text); http://archive.org/stream/journaldeconchyl111863pari#page/n453/mode/2up (plate)

[B122] Crosse H (1872a) Diagnoses molluscorum Novae Caledoniae. Journal de Conchyliologie 20: 154–157, pl. 16 http://archive.org/stream/journaldeconchyl201872pari#page/154/mode/2up (text); http://archive.org/stream/journaldeconchyl201872pari#page/n431/mode/2up (plate)

[B123] Crosse H (1872b) Description d’espèces inédites provenant de la Nouvelle-Calédonie. Journal de Conchyliologie 20: 349–359, pl. 16 http://archive.org/stream/journaldeconchyl201872pari#page/348/mode/2up (text); http://archive.org/stream/journaldeconchyl201872pari#page/n431/mode/2up (plate)

[B124] Currier AO (1868) List of the shell-bearing Mollusca of Michigan, especially of Kent and adjoining counties. Miscellaneous Publications of Kent Scientific Institute (Grand Rapids) 1: 1–12.

[B125] Dall WH (1905) Land and freshwater mollusks. Harriman Alaska Expedition 13: 9 + 171 pp., 2 pls http://archive.org/stream/landfreshwatermo00dall#page/n9/mode/2up

[B126] Dall WH (1924) Discovery of a Balkan fresh-water fauna in the Idaho Formation of Snake River Valley, Idaho. In: Mendenhall WC (Ed) Shorter Contributions in general geology 1923–1924. U.S. Geological Survey Professional Paper 132(G): 109–115, pl. 26 http://pubs.usgs.gov/pp/0132/report.pdf

[B127] Dance SP (1986) A history of shell collecting. E.J. Brill – Dr. W. Backhuys, Leiden, 230 pp.

[B128] Danielopol DL Harzhauser M Piller WE Gross M Minati K (2009) A visit to Soceni (Banat, Romania) - remembering Erich Jekelius (1889–1970). In: Sedimentary Processes amd Deposits within River-Sea Systems. Geo-Exo Marina 15: 177–183. www.nhm-wien.ac.at/jart/prj3/nhm/data/uploads/mitarbeiter_dokumente/harzhauser/2009/danetalJekelius061209.pdf

[B129] De Betta E (1870a) I molluschi terrestri e fluviatili della provincia Veronese a complemento della Malacologia di L. Menegazzi. Atti dell’Accademia di Agricoltura Arti e Commercio di Verona 47: 168 pp. http://gdz.sub.uni-goettingen.de/dms/load/img/?PPN=PPN64070204X&IDDOC=646717

[B130] De Betta E (1870b) Malacologia Veneta ossia Catalogo sinottico ed analitico dei Molluschi terrestri e fluviatili viventi nelle province Venete. Extract of Atti del Reale Istituto Veneto di Scienze, Lettere ed Arti, (ser. 3) 15: 1–141. http://archive.org/stream/malacologiavenet00bett#page/n3/mode/2up

[B131] De Gregorio A (1895) Appunti su talune conchiglie estramarine di Sicilia viventi e fossili con la spiegazione delle tavole dell’opera di Benoit. Il Naturalista Siciliano 14(10/12): 183–212. Palermo http://archive.org/stream/ilnaturalistasic9495soci#page/182/mode/2up

[B132] De Kay JE (1843) Zoology of New York, or the New-York Fauna: comprising detailed descriptions of all the animals hitherto observed within the state of New York, with brief notices of those occasionally found near its borders, and accompanied by appropriate illustrations. Part 5: Mollusca. Carroll, Cook, Albany, 8 + 271 pp., 40 pls http://archive.org/stream/zoologyofnewyork05deka#page/n7/mode/2up

[B133] De Koninck LG (1881) Faune du calcaire carbonifère de la Belgique. III (Avec un atlas de 21 planches in-folio). Gastéropodes. Annales du Musée royal d’histoire naturelle de Belgique, Série Paléontologique 6, 2 vols, 170 pp., 21 pls http://bio.emodnet.eu/component/imis/?module=ref&refid=218894

[B134] De Stefani C (1877) Molluschi continentali, fino ad ora notati in Italia nei terreni plioceni, ed ordinamento di questi ultimi. (Part 2) Continuazione e fine. Atti della Società Toscana di Scienze Naturali, Residente in Pisa 3: 274–325, pls. 17–18 (1–2) http://archive.org/stream/attidellasocie3187778soci#page/274/mode/2up (text); http://archive.org/stream/attidellasocie3187778soci#page/n447/mode/2up (plates)

[B135] De Stefani C (1880) Molluschi continentali, fino ad ora notati in Italia nei terreni pliocenici, ed ordinamento di questi ultimi. (Part 3) Continuazione. Atti della Società Toscana di Scienze Naturali, Residente in Pisa 5: 9–108, pls. 3–4 (2–3) http://archive.org/stream/attidellasociet518808soci#page/8/mode/2up (text); http://archive.org/stream/attidellasociet518808soci#page/n379/mode/2up (plates)

[B136] Degrange-Touzin MA (1892) Étude sur la faune terrestre, lacustre et fluviatile de l’Oligocène supérieur et du Miocène dans le sud-ouest de la France de principalement dans la Gironde. Actes de la Société linnéenne de Bordeaux 45: 125–230, pls. 4-5 https://archive.org/stream/mobot31753003648596#page/124/mode/2up (text), https://archive.org/stream/mobot31753003648596#page/IV/mode/2up (plates)

[B137] Delafond F Depéret CJJ (1893–94) Les terrains Tertiaires de la Bresse et leur gîtes de lignites et de minerais de Fer. 2me Volume Études des gîtes minéraux de la France. Imprimerie nationale, Paris, 332 pp., 58 figs, 19 pls., 1 map http://gallica.bnf.fr/ark:/12148/bpt6k62272678

[B138] Derjavin AN (1927) Notes on the Upper Sarmatian amphipods of the Ponto-Caspian region. Bulletin de la Société des Naturalistes de Moscou, Section de Geologie (n.s.) 35(3–4): 183–196.

[B139] Deshayes GP (1861–1863) [for exact dating of parts see Bouchet, Rocroi 2005: 301] Description des animaux sans vertèbres découverts dans le bassin de Paris pour servir de supplément à la description des coquilles fossiles des environs de Paris comprenant une revue générale de toutes les espèces actuellement connues. Tome deuxième Mollusques acéphales monomyaires et brachiopodes. Mollusques céphales, première partie. J.B. Balliere et Fils, Paris, 1–968, pls. 1–64 http://archive.org/stream/descriptiondesan02desh#page/n7/mode/2up (text); http://archive.org/stream/descriptiondesan0203pldes#page/n9/mode/2up (legends); http://archive.org/stream/descriptiondesan0203pldes#page/n115/mode/2up (plates)

[B140] Dézallier d’Argenville AJ (1742) L’histoire naturelle éclaircie dans deux de ses parties principales, la lithologie et la conchyliologie: dont l’une traite des pierres et l’autre des coquillages: ouvrage dans lequel on trouve une nouvelle méthode, une notice critique des principaux auteurs qui ont écrit sur ces matiéres: enrichi de figures dessinées d’après nature. Bure l’Ainé, Paris 8 + 491 + 1 pp. + 33 pls. http://archive.org/stream/lhistoirenaturel00dza#page/n11/mode/2up; http://gdz.sub.uni-goettingen.de/dms/load/img/?PPN=PPN393596060&IDDOC=281410

[B141] Dollfus G-F (1877) *Valvata disjuncta*, G. Dollf., espèce nouvelle des meulières supérieures des environs de Paris. Annales de la Société Malacologique Belgique 12: 27–28. http://archive.org/stream/annalesdelasocit12soci#page/26/mode/2up

[B142] Dollfus G-F (1908) Revision de la feuille de Fontainebleau au 80.000e, feuille de Bourges (Terraines tertiaries) au 320.000e. Bulletin des Services de la Carte géologique de la France et des topographies souterraines, Comptes rendus des collaborateurs pour la campagne 1907 18(119): 8–20, figs 1–2.

[B143] Dollfus G-F (1916) Étude sur la Molasse de l’Armagnac. Bulletin de la Société Géologique de France (4) 15: 335–402. http://archive.org/stream/bulletindelasoci4151915soci#page/334/mode/2up

[B144] Dollfus G-F Dautzenberg P (1886) Étude preliminaire de coquilles fossiles des faluns de la Touraine (suite). La Feuille des Jeunes Naturalistes 16(192): 138–143. http://archive.org/stream/lafeuilledesjeun17pari#page/n623/mode/2up

[B145] Draparnaud J (1801) Tableau des mollusques terrestres et fluviatiles de la France. Renaud, Bossange Montpellier, Paris, 116 pp. http://archive.org/stream/tableaudesmollus00draparn#page/n9/mode/2up

[B146] Draparnaud J (1805) Histoire naturelle des mollusques terrestres et fluviatiles de la France. A. Paris, Louis Colas. 8 + 163 pp., 16 pls. http://archive.org/stream/histoirenaturell00dra#page/n9/mode/2up

[B147] Drouët H (1867) Mollusques terrestres et fluviatiles de la Côte-d’Or. Baillière et fils, Paris, 122 pp. http://archive.org/stream/mollusquesterres00drou#page/n9/mode/2up

[B148] Duméril AMC (1805) ("1806") Zoologie analytique, ou méthode naturelle de classification des animaux, rendue plus facile à l’aide de tableaux synoptiques. Allais, Paris, 32 + 344 pp. http://archive.org/stream/zoologieanalytiq00dumr#page/n7/mode/2up

[B149] Dupuy D (1847–52) Histoire naturelle des mollusques terrestres et d’eau douce qui vivent en France. V. Masson, Paris, 31 + 737 pp., 31 pls. http://archive.org/stream/histoirenaturell00dupu#page/n7/mode/2up

[B150] Durand A (1913) Le Pliocène de la région de Saint-Laurent-des Arbres (Gard). Compte rendu de la Association Francaises pour l’Avancement des Sciences. 41^e^ session Nîmes 1912: 345–352. http://archive.org/stream/compterendu41asso#page/344/mode/2up

[B151] Dybowski / Dybovskij B(enedykt) (1911) O faunie mienczakow Baykalskikh. Kosmos 36: 10–12, 945–981.

[B152] Dybowski / Dybovskij B(enedykt) Godlewski / Godlevskij W (1870) Predvaritel’nyy otchet o faunisticheskikh issledovaniyakh na Baykale [Preliminary report on the faunistic research at the Baikal] (in Russian). In: Usol’tsev AF (Ed) Otchet o deystviyakh Sibirskago otdela Imperatorskago Russkago Geograficheskago Obshchestva za 1869 g., Prilozhenie [Report on the work of the Siberian Department of the Imperial Russian Geographic Society in 1869. Supplement]. St Petersburg, Russia, 167–204

[B153] Dybowski / Dybovskij B(enedykt) Grochmalicki J (1917) Studien über die turmförmigen Schnecken des Baikalsees und des Kaspimeeres. (Turribaicaliinae - Turricaspiinae). Abhandlungen der kaiserlich-königlichen zoologisch-botanischen Gesellschaft in Wien 9(3): 1–55, pls. 1–4. http://www.landesmuseum.at/pdf_frei_remote/AZBG_9_3_0001-0055.pdf

[B154] Dybowski / Dybovskij W(ladimir) (1875) Die Gastropoden-Fauna des Baikal-Sees anatomisch und systematisch bearbeitet. Mémoires de l’Academie impériale des Sciences de St.-Pétersbourg (7) 22(8): 1–73, 8 pls. [pdf-file available on request from the author]

[B155] Dybowski / Dybovskij W(ladimir) (1886) Ueber zwei neue sibirische *Valvata* - Arten. Jahrbücher der Deutschen Malakozoologischen Gesellschaft 13: 107–121, pl. 4. http://archive.org/stream/jahrbcherderde131886deut#page/n117/mode/2up (text); http://archive.org/stream/jahrbcherderde131886deut#page/n359/mode/2up (plate) core.kmi.open.ac.uk/download/pdf/4509418

[B156] Dybowski / Dybovskij W(ladimir) (1903) Beitrag zur Kenntnis der Mollusken- Fauna Kamchatka’s. Ezhegodnik / Annuaire du Musée Zoologique de l’Académie Impériale des Sciences de St.-Pétersbourg 8: 40–55. http://archive.org/stream/ezhegodnik08zool#page/40/mode/2up

[B157] Egorov R (2006) Treasure of Russian Shells. Supplement 4. Illustrated Catalogue with Selected Keys of the Recent Fresh- and Brackish-water Pectinibranch Molluscs (Gastropoda: Pectinibranchia) of Russia and Adjacent Areas. Colus-Doverie, 88 pp. (ISBN: 5873172927).

[B158] Eichwald E (1830) Naturhistorische Skizze von Lithauen, Volhynien und Podolien in geognostisch-mineralogischer, botanischer und zoologischer Hinsicht. Joseph Zawadzki, Wilna, 256 pp., 2 pls.

[B159] Eichwald E (1853) Lethaea rossica ou Paleontologie de la Russie. Volume III. Derniere Periode. Schweizerbart, Stuttgart, 19 + 2 + 533 pp., 14 pls. http://archive.org/stream/lethaearossicaou03eich#page/n3/mode/2up; http://archive.org/stream/lethaearossicaou003eich#page/n7/mode/2up

[B160] Esu D (1983) Presence de *Valvata chalinei* Schlickum, Puissegur dans le Villafranchien superieur de Leffe (Italie) (Prosobranchia: Valvatidae). Archiv für Molluskenkunde 114(1–3): 65–68.

[B161] Esu D (1984) La Malacofauna continentale Pliocenica di Mandriola (Sardegna occidentale): sistematica e paleobiogeografia. Geologica Romana 23: 23–50. http://tetide.geo.uniroma1.it/dst/grafica_nuova/pubblicazioni_DST/geologica_romana/Volumi/VOL%2023/GR_23_23_50_Esu.pdf

[B162] Esu D Girotti O (1975) La malacofauna continentale del Plio-Pleistocene dell’Italia centrale. I. Paleontologia. Geologica Romana 13: 203–294. http://tetide.geo.uniroma1.it/dst/grafica_nuova/pubblicazioni_DST/geologica_romana/Volumi/VOL%2013/GR_13_203_293_Esu%20et%20al.pdf

[B163] Eydoux JFT Souleyet LFA (1852) Zoologie. Voyage autour du monde: executé pendant les anneés 1836 et 1837 sur la corvette La Bonite commandé par M. Vaillant Capitaine de Vaisseau publié par ordre du gouvemement sous les auspices du Département de la Marin. Arthus Bertrand, Paris Volume 2: 664 pp., 45 pls. http://archive.org/stream/zoologie2121852eydo#page/n7/mode/2up

[B164] Fagot P (1881) Diagnoses de Mollusques nouveaux pour la faune Française. Bulletin de la Société Zoologique de France 6: 137–141. http://archive.org/stream/bulletindelas1881soci#page/136/mode/2up

[B165] Fagot P (1892) Histoire malacologique des Pyrénées Françaises et espagnoles – Liste des Espèces (suite et fin de la premiere partie). Explorations Pyrénéennes - Bulletin de la Société Ramond de Bagnères-de-Bigorre 27(1): 23–41. http://gallica.bnf.fr/ark:/12148/bpt6k6535108f/f25.image

[B166] Falkner G Bank RA Proschwitz Tv (2001) Check-list of the non-marine molluscan species-group taxa of the states of Northern, Atlantic, and Central Europe (CLECOM I). Heldia 4(1/2): 1–76.

[B167] Falkner G Boeters H-D (2003) Beiträge zur Nomenklatur der europäischen Binnenmollusken. XVI. Zum Status der *Islamia bourguignati* (T. Letrourneux 1869). Heldia 5(1–2): 19–22, pl. 6b.

[B168] Falkner G Obrdlik P Castella E Speight MCD (2002) Shelled Gastropoda of Western Europe. [Book, CD-ROM] Conchbooks, 267 pp., 12 figs, 6 pls.

[B169] Falniowski A (1990) Anatomical characters and SEM structure of radula and shell in the species-level taxonomy of freshwater prosobranchs (Mollusca: Gastropoda: Prosobranchia): a comparative usefulness study. Folia Malacologica (Krakow) 4(1276): 53–142 + 346 figs.

[B170] Falniowski A Economou-Amilli A Anagnostidis K (1988) *Valvata piscinalis* O. F. Müller (Mollusca, Prosobranchia) and its epizoic diatoms from Lake Trichonis, Greece. Internationale Revue der gesamten Hydrobiologie und Hydrogeographie 73(3): 327–335. http://onlinelibrary.wiley.com/doi/10.1002/iroh.19880730308/abstract (free abstract, pdf-download requires subscription)

[B171] Falsan A (1875) Études sur la position stratigraphique des tufs de Meximeux de Pérouges et de Montluel. Archives du Muséum d’histoire naturelle de Lyon 1: 135–170. H. Georg Libraire Ed., Lyon et Genève http://dds.crl.edu/CRLdelivery.asp?tid=11262

[B172] Favre J (1922) Les *Valvata* post-glaciaires et actuelles du bassin de Genève. (Archives de Sciences et) Compte rendu des sancés de la Société de physique et d’histoire naturelle de Genève 4(5): 49–53. http://gallica.bnf.fr/ark:/12148/bpt6k299154k/f516.image.r=Archives%20Soci%C3%A9t%C3%A9%20de%20physique%20et%20d%27histoire%20naturelle%20de%20Gen%C3%A8ve.langDE

[B173] Favre J (1927) Les mollusques post-glaciaires et actuels du bassin de Genève. Mémoires de la Société de Physique et d’Histoire Naturelle de Genève 40(3): 171–434, pls. 14–27.

[B174] Favre J (1935) Études sur la partie occidentale du Lac de Genève II. Histoire malacologiques du Lac de Genève. Mémoires de la Société de Physique et d’Histoire Naturelle de Genève 41(3): 295–414, pl. 13.

[B175] Ferry H de (1870) *Maçonnais préhistorique*. Mémoire sur les âges primitifs de la Pierre, du Bronze et du Fer en Mâconnais et dens quelques contrées limitrophes. 8 + 105 pp. Appendice par Adrien Arcelin, 106–136, 42 pls. Màcon et Paris http://gallica.bnf.fr/ark:/12148/bpt6k5613795j (text only, no plates) https://play.google.com/store/books/details?id=YrNBAAAAcAAJ&rdid=book-YrNBAAAAcAAJ&rdot=1 (requires registration at google-books)

[B176] Férussac JBL d’Audebard de Férussac AEJPJF d’Audebard de (1807) Essai d’une méthode conchyliologique appliquée aux mollusques fluviatiles et terrestres d’après la considération de l’animal et de son testpar M. Daudebard de Férussac. Nouvelle édition augmentée d’une synonymie des espèces les plus remarquables, d’une table de concordance systématique de celles qui ont été décrites par Géoffroy, Poiret et Draparnaud, avec Müller et Linné, et terminée par un catalogue d’espèces observées en divers lieux de la France, par J. Daudebard fils. Delance, Paris 16 + 142 pp. http://gdz.sub.uni-goettingen.de/dms/load/img/?PPN=PPN614791812&IDDOC=585556

[B177] Férussac JBL d’Audebard de (1821–22) Tableaux systématiques des animaux Mollusques classés en familles naturelles, dans lesquels on a établi la concordance de tous les systèmes; suivis d’un prodrome général pour tous les Mollusques terrestres ou fluviatiles, vivants ou fossiles. A. Bertrand, Paris, 110 pp. http://archive.org/stream/tableauxsystma00fr#page/n7/mode/2up

[B178] Fischer J-C (1964) Contribution a l’étude de la faune bathonnienne dans le Vallée de la Creuse (Indre) Brachiopodes et Mollusques. Annales de Paléontologie 50: 19–101, 2 tables, 4 pls. http://rogov.zwz.ru/Fischer%20J.-C.,%201964.pdf

[B179] Fischer O (1855) On the Purbeck strata of Dorsetshire. Transactions of the Cambridge Philosophical Society 9(9): 555–581. http://archive.org/stream/transactionsofca09camb#page/554/mode/2up

[B180] Fischer P (1866) Faune Tertiaire Lacustre In: Tchihatcheff PA de (1866–69): Asie mineure, description physique, statistique et archéologie de cette contrée. Vol. 5: Paléontologie (par d’Archiac A, Fischer P, Verneuill E de). Ed. L. Guérin, Paris 327–351, pl. 6 http://books.google.de/books/about/Asie_Mineure.html?id=MLRBAAAAcAAJ&redir_esc=y

[B181] Fischer P (1880–87) (for exact publication dates of the various parts see page after title) Manuel de conchyliologie et de paléontologie conchyliologique. Libraire F. Savy, Paris, 24 + 1369 pp., 23 pls., 1 map http://archive.org/stream/manueldeconchyli00fisc#page/n9/mode/2up

[B182] Fischer P Crosse H (1880–1902) Etudes sur les Mollusques terrestres et fluviatiles du Mexique et du Guatemala. In: Milne-Edwards M (Ed) Recherches Zoologiques pour servir à l’histoire de la faune de l’Amérique Centrale et du Mexique. Imprimerie Nationale, Paris, (7) 2(8–17): 2 + 712 pp., pls. 32–72 http://www.animalbase.uni-goettingen.de/zooweb/servlet/AnimalBase?nav=ShowOneReference&id=7945 (detailed comment and time table) Livraison 8 (1880): feuilles 1–10, pls. 32–36 Livraison 9 (1886): feuilles 11–16, pls. 37–42 Livraison 10 (1888): feuilles 17–22, pls. 43–46 Livraison 11 (1890): feuilles 23–32, pls. 47–48 Livraison 12 (1891): feuilles 33–39, pls. 49–52 Livraison 13 (1892): feuilles 40–49, pls. 53–54 Livraison 14 (1893): feuilles 50–61, pls. 55–58 Livraison 15 (1894): feuilles 62–72, pls. 59–62 Livraison 16 (1894): feuilles 73–82, pls. 63–66 Livraison 17 (1902): feuilles 83–92, pls. 67–72 http://archive.org/details/tudessurlesmol21900fisc

[B183] Fitzinger L (1833) Systematisches Verzeichnis der im Erzherzogthume Oesterreich vorkommenden Weichthiere als Prodrom einer Fauna derselben. Beiträge zur Landeskunde Oesterreich’s unter der Enns 3: 88–122. http://archive.org/stream/systematischesve00fitz#page/88/mode/2up

[B184] Floriu A (2011) Analiza integrata (biostratigrafica, tectonica, stratigrafie seismica) a Paratethysului Oriental (Bazinul Dacic, Marea Neagra, Peninsula Taman) in timpul Pontianului. Thesis, Universitatea din Bucaresti, Facultatea de Geologie si Geofizica, 64 pp. http://www.unibuc.ro/studies/Doctorate2011August/Floroiu%20Alina%20-%20Analiza%20Integrata%20(biostratigrafica%20tectonica,%20stratigrafie%20seismica)%20a%20Paratethysului%20Oriental%20(Bazinul%20Dacic,%20Marea%20Neagra,%20Peninsula%20Taman)%20in%20Timpul%20Pontianului/Floroiu%20Rezumat%20teza.pdf

[B185] Fluck WH (1932) *Valvata simplex* Gould. The Nautilus 46(1): 19–22. http://archive.org/stream/nautilus46amer#page/n31/mode/2up

[B186] Fontannes F (1876) Le Vallon de la Fuly et les Sables à Buccins des Environs d’Heyrieu (Isere). Études stratigraphiques et paléontologiques pour servir à l’histoire de la Période Tertiaire dans le bassin du Rhône. Annales de la Société d’Agriculture (histoire naturelle et arts utiles) de Lyon (4) 8 F. Savy, Paris, 59 pp. + 2 pls. http://gallica.bnf.fr/ark:/12148/bpt6k6132401p.image

[B187] Fontannes F (1880) Le bassin de Crest, Drome. Études stratigraphiques et paléontologiques pour servir à l’histoire de la période tertiaire dans le bassin du Rhone, 6. A-C. Georg, Lyon, F. Savy, Paris, 214 pp., pls. 1–7, http://gallica.bnf.fr/ark:/12148/bpt6k5441803c

[B188] Fontannes F (1883) Diagnoses espèces et de variétés nouvelles des terrains tertiaries du Bassin du Rhone. Bulletin de la Société Géologique de France 11: 440–441. http://goo.gl/ZN3HRq

[B189] Fontannes F (1886) Contribution à la faune malacologique des terrains néogènes de la Roumanie. Archives du Muséum d’histoire naturelle de Lyon 4(1): 321–365, pls. 26–27 http://archive.org/stream/mobot31753003649081#page/n329/mode/2up

[B190] Forcart L (1948) Beschreibung neuer Schnecken von Celebes, Rotti, Neu Guinea und Venezuela. Verhandlungen der Naturforschenden Gesellschaft zu Basel 59: 45–54, pl. 1.

[B191] Forcart L (1957) Ipsa Studeri Conchylia. Professor Samuel Studer (17571834), seine Bedeutung als Naturforscher und die von ihm hinterlassene Molluskensammlung. Mitteilungen der Naturforschenden Gesellschaft in Bern N.F. 15: 157–210, 7 pls. 10.5169/seals-319492

[B192] Fougeroux de Denainvilliers A de (1875) Description de quelques espèces de coquilles fossiles des terrains Tertiaires des environments de Paris. Journal de Conchyliologie 23: 68–75, pl. 3 http://archive.org/stream/journaldeconchyl231875pari#page/68/mode/2up (text) http://archive.org/stream/journaldeconchyl231875pari#page/n403/mode/2up (plate)

[B193] Frauenfeld GV (1863) Vorläufige Aufzählung der Arten der Gattungen *Hydrobia* Htm. und *Amnicola* Gld. Hldm. in der kaiserlichen und in Cuming’s Sammlung. Verhandlungen der Zoologisch-Botanischen Gesellschaft in Wien 13: 1017–1032. www.landesmuseum.at/pdf_frei_remote/VZBG_13_1017-1032.pdf

[B194] Frauenfeld GV (1864) Verzeichnis der Namen der fossilen und lebenden Arten der Gattung *Paludina* Lam., nebst jenen der nächststehenden und Einreihung derselben in die verschiedenen neueren Gattungen. Verhandlungen der Zoologisch-Botanischen Gesellschaft in Wien 14: 561–672. www.landesmuseum.at/pdf_frei_remote/VZBG_14_0561-0672.pdf

[B195] Frest TJ Johannes EJ (1995) Interior Columbia Basin mollusk species of special concern. Final Report Interior Columbia Basin Ecosystem Management, 215 pp. www.icbemp.gov/science/frest_1.pdf

[B196] Fretter V (1956) The anatomy of the prosobranch Circulus striatus (Philippi) and a review of its systematic position. Proceedings of the Zoological Society of London 126(3): 369–381. (free abstract, download of pdf requires subscription) http://onlinelibrary.wiley.com/doi/10.1111/j.1096-3642.1956.tb00443.x/abstract

[B197] Friedl E (1871) Betrachtungen über Weichthiere der Mark Brandenburg. Nachrichtsblatt der Deutschen Malakozoologischen Gesellschaft 3: 73–77. http://archive.org/stream/nachrichtsblattd35187173deut#page/n85/mode/2up

[B198] Fritzsche CH (1924) Beiträge zur Geologie und Paläontologie von Südamerika. XXVII. Neue Kreidefaunen aus Südamerika (Chile, Bolivia, Peru, Columbia). Neues Jahrbuch für Mineralogie, Geologie und Palaeontologie 50: 1–56, pls. 1–4.

[B199] Froriep LF von (1806) Duméril’s analytische Zoologie aus dem Französischen mit Zusätzen. Verlag des Landes-Industrie-Comptoirs, Weimar, 6 + 343 pp. http://reader.digitale-sammlungen.de/de/fs3/object/display/bsb10307091_00001.html

[B200] Fuchs T (1870a) Beiträge zur Kenntnis fossiler Binnenfaunen III. Die Fauna der Congerien Schichten von Radmanesti in Banate. Jahrbuch der kaiserlich königlichen Geologischen Reichsanstalt 20(3): 344–364, pls. 14–17 www.geologie.ac.at/filestore/download/JB0203_343_A.pdf

[B201] Fuchs T (1870b) Beiträge zur Kenntnis fossiler Binnenfauna IV und V. Die Fauna der Congerien Schichten von Tihany am Plattensee und Kúp bei Pápa in Ungarn. Jahrbuch der kaiserlich königlichen Geologischen Reichsanstalt 20(4): 531–547, pls. 20–22 www.geologie.ac.at/filestore/download/JB0204_531_A.pdf https://play.google.com/store/books/details?id=kNZAAAAAcAAJ&rdid=book-kNZAAAAAcAAJ&rdot=1 (requires registration at google-books)

[B202] Fuchs T (1877) Studien über die jüngeren Tertiärbildungen Griechenlands. Denkschriften der Österreichischen Akademie der Wissenschaften, mathematisch-naturwissenschaftliche Klasse 37 II: 1–42, pls. 1–5 www.landesmuseum.at/pdf_frei_remote/DAKW_37_2_0001-0042.pdf

[B203] Fuhrmann R (1990) Die Molluskenfauna des Interglazials von Gröbern (Kreis Gräfen-hainichen). Altenburger naturwissenschaftliche Forschungen 5: 148–167, 6 pls., 1 tab.

[B204] Fujita T Habe T (1991) 長野県木崎湖・中綱湖産ミズシタダミ科の新種キザキコミズシタダミ [*Cincinna kizakikoensis* n.sp. of the family Valvatidae from Lakes Kizaki and Nakatsuna, Nagano Prefecture, Japan]. Venus, Japanese Journal of Malacology 50(1): 23–26. [in Japanese, English summary] http://ci.nii.ac.jp/naid/110004764857/en (free abstract, download of pdf requires subscription)

[B205] Furrow CL (1931) Some observations on the hermaphrodite apparatus of *Valvata tricarinata* (Say). Transactions of the Illinois State Academy of Sciences 24(2): 241–246. (pdf of paper available on request from the author)

[B206] Furrow CL (1935) Development of the hermaphroditic genital organs of *Valvata tricarinata*. Zeitschrift für Zellforschung 22(3): 282–304. http://emedia1.bsb-muenchen.de/han/858/link.springer.com/content/pdf/10.1007%2FBF02451157.pdf (free abstract, download of pdf requires subscription of Cell, Tissue Research), doi: 10.1007/BF02451157

[B207] Gallenstein M (1848) Systematisches Verzeichniss der in der Provinz Kärnten bisher entdeckten Land-, Süsswasser-Conchylien, mit Angabe der wichtigsten Fundorte, nebst einer kurzen Anleitung für angehende Conchylien-Sammler. Laibach, Blasnik, 1–28. http://www.animalbase.uni-goettingen.de/zooweb/servlet/AnimalBase/home/digireference?id=83

[B208] Garnault P (1889) Sur les organes reproducteurs de la *Valvata piscinalis*. Zoologischer Anzeiger 12: 266–269. http://archive.org/stream/zoologischeranze12deut#page/266/mode/2up

[B209] Garnault P (1890) Les organes reproducteurs de la *Valvata piscinalis*. Bulletin biologique de la France et de la Belgique 22: 496–507, pl. 26 http://archive.org/stream/bulletinbiologiq22univ#page/496/mode/2up (text) http://archive.org/stream/bulletinbiologiq22univ#page/n575/mode/2up (plate)

[B210] Gassies J-B (1849) Tableau méthodique et descriptif des mollusques terrestres et d’eau douce de l’Agenais. Paris, Agen (Ballière). 1 + 209 + 4 pp., pls. 1–4 http://archive.org/stream/tableaumthodiq00gass#page/n7/mode/2up

[B211] Geoffroy EL (1767) Traité sommaire des coquilles, tant fluviatiles que terrestres, qui se trouvent aux environs de Paris. Musier, Paris (St. Etienne), 9 + 3 + 143 pp. http://archive.org/stream/traitsommaired00geoff#page/n1/mode/2up (Remark: ICZN 0.362: rejected for nomenclatural purposes, because the author did not apply the principles of binominal nomenclature.)

[B212] Germain L (1909) Contributions à la faune malacologique de l’Afrique équatorial. XIX: Mollusques nouveaux de l’Afrique tropicale. Bulletin du Muséum Nationale d’Histoire naturelle, Paris 15(6): 375–379. http://archive.org/stream/contributionsl01germ#page/n173/mode/2up http://archive.org/stream/bulletindumusumn15musu#page/374/mode/2up

[B213] Germain L (1911) Mollusques terrestres et fluviatiles de l’Asie Antérieure (2e Note). Bulletin du Muséum National d’Histoire naturelle Paris 17: 63–67. http://archive.org/stream/bulletindumusu17mus#page/62/mode/2up

[B214] Germain L (1931) Mollusques terrestres et fluviatiles. Vol. II Faune de France 22: 520 pp., pls. 1–26 www.faunedefrance.org/BibliothequeVirtuelleNumerique

[B215] Gerstfeldt G (1859) Über Land und Süsswasser-Mollusken Sibiriens und des Amur-Gebietes. Zapiski Imperatorskoĭ akademīi nauk po Fiziko-matematicheskomu otdielenīiu = Mémoires présentés à l’Académie Impériale des Sciences de St. Pétersbourg par divers Savants et dans ses Assemblées 9: 505–548, 1 pl. http://archive.org/stream/mmoiresprsentsla09impe#page/n535/mode/2up

[B216] Gill T (1863) Systematic arrangement oft the mollusks of the family Viviparidae and others, inhabiting the United States. Proceedings of the Academy of Natural Sciences of Philadelphia 15: 33–40. http://archive.org/stream/proceedingsofaca15acad#page/32/mode/2up

[B217] Gillet H Leriocoalis G Rehault J Dinu C (2003) La stratigrafie oligo-miocène et la surface d’erosion messinienne en Mer Noire, stratigraphie sismique haute résolution. Comptes Rendus Geoscience 335(12): 907–916. http://www.sciencedirect.com/science/article/pii/S1631071303001391, doi: 10.1016/j.crte.2003.08.008

[B218] Giusti F Pezzoli E Bodon M (1982) Notulae Malacologicae, XXVIII: Primo contributo alia revisione del genre *Islamia* (Radoman, 1973) in Italia. Lavori della Società di Malacologica Italiana 19–20: 49–71, pls. 1–7.

[B219] Glaubrecht M (2012) Franz Hilgendorf’s dissertation “Beiträge zur Kenntnis des Süßwasserkalks von Steinheim” from 1863: Transcription and description of the first Darwinian interpretation of transmutation. Zoosystematics, Evolution 88(2): 231–259. http://www.naturkundemuseum-berlin.de/fileadmin/startseite/institution/mitarbeiter-publikationen/glaubrecht/Glaubrecht_ZSE_2012_Hilgendorf.pdf

[B220] Glöer P (2002) Mollusca I. Süßwassergastropoden Nord- und Mitteleuropas. Bestimmungsschlüssel, Lebensweise, Verbreitung. [ISBN 3–925919–60–0] Conchbooks, Hackenheim, 327 pp.

[B221] Glöer P Diercking R (2010) Atlas der Süßwassermollusken. Rote Liste, Verbreitung, Ökologie, Bestand und Schutz. Gutachten für die Behörde für Stadtentwicklung und Umwelt, Hamburg 180 pp. www.malaco.de/Sonderdrucke/atlas-suesswassermollusken.pdf

[B222] Glöer P Girod A (2012) A new Pleistocene *Valvata* species from Lake Beyşehir and two new *Gyraulus* species from Lake Eǧirdir (Mollusca: Gastropoda: Valvatidae, Planorbidae) in Turkey. Folia Malacologia 21(1): 25–31. www.malaco.de/Sonderdrucke/Gloeer_Girod

[B223] Glöer P Pešić V (2008) The freshwater gastropods of the Skadar Lake with the description of *Valvata montenegrina* n. sp. (Mollusca, Gastropoda, Valvatidae). In: Pavicevic D Perreau M (Eds) Advances in the studies of the subterranean and epigean fauna of the Balkan Peninsula. Monography of the Institute of Nature Conservation of Serbia 22: 341–348. Institute for Nature Conservation of Serbia, Belgrad www.malaco.de/Sonderdrucke/Gloeer-Pesic-Serbia.pdf

[B224] Glöer P Pešić V (2012) The freshwater snails (Gastropoda) of Iran, with descriptions of two new genera and eight new species. ZooKeys 219: 11–61. www.pensoft.net/J_FILES/1/articles/3406/3406-G-3-layout.pdf, doi: 10.3897/zookeys.219.3406 PMC3433696 22977349

[B225] Glöer P Zettler ML (2005) Kommentierte Artenliste der Süßwassermollusken Deutschlands. Malakologische Abhandlungen (Dresden) 23: 3–26. www.malaco.de/Sonderdrucke/Gloeer_et_Zettler-checklist.pdf

[B226] Gmelin JF (1791) Caroli a Linné, systema naturae. Tom. I Pars 6: 3021–3910. Lipsiae (Beer). http://www.biodiversitylibrary.org/item/83098#page/4/mode/2up

[B227] Gorjanović-Kramberger D (1890) Die praepontischen Bildungen des Agramer Gebirges. Glasnik hrvatskoga naravoslovnoga Družtva 5, 151–164, pl. 6 http://archive.org/stream/glasnikhrvatskog1890hrva#page/150/mode/2up

[B228] Gould AA (1841) Report on the Invertebrata of Massachusetts: comprising the Mollusca, Crustacea, Annelida, and Radiata. Thurston, Cambridge, Folsom, Wells, 6 + 373 pp., 213 figs (15 pls.) http://archive.org/stream/reportoninverteb01goul#page/n19/mode/2up

[B229] Gould AA (1961) Desciptions of new species of shells. Proceedings of the Boston Society of Natural History 7: 40–45. http://archive.org/stream/proceedingsofbos59bost#page/40/mode/2up

[B230] Gozhik / Gožyk / Gożik / Gozhyk PF (2002) Pontični prisnovodni moljuski pivdnja Ukraïni i Moldovi. [Pontian freshwater molluscs of Ukraina and Moldavia] ISBN 9667731103, 9789667731106, 95 pp.

[B231] Gozhik / Gožyk / Gożik / Gozhyk PF Datsenko LN (2007) Пресноводные моллюски позднего кайнозоя юга Восточной Европы [в 2 ч.] / П. Ф. Гожик, Л. Н. Даценко; Национальная академия наук Украины, Институт геологических наук. [Fresh-water molluscs from the Late Caenozoic in the south of Eastern Europe] Part II: Family Sphaeridae, Pisidiidae, Corbiculoidae, Neritidae, Viviparidae, Valvatidae, Bithyniidae, Lithoglyphidae, Melanopsidae. National Academy of Sciences of Ukraine, Institute of Geological Science, Kiev, 2007: 1–255 (plates included) [ISBN: 978–966–581–923–3] [in Russian]

[B232] Gozhik / Gožyk / Gożik / Gozhyk PF Prisiazhiuk / Prysjazhnjuk VA (1978) Presnovodnye i nazemnye molliuski miotsena Pravoberezhnoĭ Ukrainy. Akademiia nauk Ukrainskoi SSR, Institut geologicheskikh nauk, 178 pp., 20 pls.

[B233] Grabau AW (1923) Cretaceous fossils from Shantung. Jing ji bu zhong yang di zhi diao cha suo / Bulletin of the Geological Survey of China 5(2): 143–181, 2 pls.

[B234] Gray JE (1840) [A new edition of] A Manual of the land and freshwater shells of the British Islands by W. Turton. Longman, Orme, Brown, Green, and Longmans, London, ix + 324 pp., 12 pls. http://archive.org/stream/manualoflandfres00turtrich#page/n5/mode/2up

[B235] Gray JE (1855) List of Mollusca and shells in the collection of the British Museum, collected and described by Eydoux and Souleyet (1855). London, printed by the order of the trusties 27 pp. http://archive.org/stream/listofmolluscash00brituoft#page/n3/mode/2up

[B236] Gredler VM (1859) Tirol’s Land- und Süsswasser-Conchylien. II. Die Süsswasser-Conchylien. Verhandlungen der Zoologisch-Botanischen Gesellschaft Wien 9: 213–308, 1 pl. http://www.landesmuseum.at/pdf_frei_remote/VZBG_9_0213-0308.pdf

[B237] Gregory SMS (2010) The two ‘editions’ of Duméril’s Zoologie analytique, and the potential confusion caused by Froriep’s translation Analytische Zoologie. Zoological Bibliography 1(1): 6–8. http://www.avespress.com/featured-periodicals/

[B238] Gruithuisen F von P (1821) Die Branchienschnecke und eine aus ihren Ueberresten hervorwachsende lebendig-gebaehrende Conserve. Nova Acta physico-medica Academiae Caesareae Leopoldino-Carolino Naturae Curiosum 10: 437–452, pl. 38 http://archive.org/stream/novaactaphysicom10182kais#page/n601/mode/2up

[B239] Gude GK (1912) Explanation of the figures occurring in Westerlund’s “Sibiriens Land- och Sötvatten-Mollusker”, 1877. Journal of Molluscan Studies 10(1): 24 http://mollus.oxfordjournals.org/content/10/1.toc

[B240] Guilding L (1828) Observations on the Zoology of the Caribæan Islands. Zoological Journal 3 (Art. 61): 527–544. http://archive.org/stream/zoologicaljourna318271828sowe#page/526/mode/2up

[B241] Guo F Yü W Pan H-Zg (1982) Phylum Mollusca, Class Gastropoda. In: Shaanxi Gansu Ningxia Volume / Palaeontological Atlas of Northwest China. Part 3: Mesozoic and Cenozoic. Geological Publishing House, Bejing, 28–52, 107 pls. (pdf-file available on request from the author)

[B242] Haas F (1938) Über potenzielle Skulpturbildung bei *Valvata (Cincinna) piscinalis antiqua* (Sow.). Archiv für Molluskenkunde 70: 41–45. (pdf-file available on request from the author)

[B243] Haas F (1939) Malacological Notes: Notes on valvatids with a description of a new subgenus. Zoological Series of Field Museum of Natural History 24(8): 100–102. http://archive.org/stream/malacologicalnot248haas#page/100/mode/2up

[B244] Hadžišče S (1955) (“1953“) Prilog poznavanju Gastropoda Prespanskog i Ohridskog Jezera / Beitrag zur Kenntnis der Gastropodenfauna des Prespa- und Ohridsees. Glasnik Bioloske Sekcije [Periodicum Biologorum], Hrvatsko Prirodoslovno Drustvo (II/B) 7(1): 174–177.

[B245] Hadžišče S (1956) III. Beitrag zur Kenntnis der Gastropodenfauna des Ohridsees. Beschreibungen der bis jetzt unbekannten Schnecken und Beispiele der Speciation bei den Gastropoden des Ochridsees. Sbornik rabotite hidrobioloski sawod Ohrid / Recueil des Travaux de la Station Hydrobiologique Ohrid 4(1): 57–107.

[B246] Hadžišče S Patterson CM Burch JB LoVerde PT (1976) The embryonic shell surface sculpture of Gocea and Valvata. Malacogical Review 9: 1–14.

[B247] Hagen H (1864) Ueber Phryganiden-Gehäuse. Entomologische Zeitung 25(4–6): 113–144. http://archive.org/stream/entomologischeze251864ento#page/112/mode/2up

[B248] Hagenmüller P (1884) Clausilie et valvées nouvelles du Nord de l’Afrique. Bulletins de la Société malacologique de France 1: 209–216. http://archive.org/stream/bulletinsdelasoc11884soci#page/208/mode/2up

[B249] Halaváts J (1889) Die zwei artesischen Brunnen in Hódmezó-Vásárhely. Mittheilungen aus dem Jahrbuche der königlichen ungarischen geologischen Anstalt 8: 214–231, pls. 33–34.

[B250] Hanna GD (1922) Fossil freshwater mollusks from Oregon contained in the Condon Museum of the University of Oregon. Volume 1(12): 23 pp. including 4 pls. http://archive.org/stream/fossilfreshwater00hanniala#page/n1/mode/2up

[B251] Hanniball HA (1910) Valvatidae of the western North America. The Nautilus 23(8): 104–107. http://archive.org/stream/nautilus23amer#page/104/mode/2up

[B252] Hannibal HA (1912) A synopsis of the Recent and Tertiary freshwater Mollusca of the Californian province based upon an ontogenetic classification. Proceedings of the Malacological Society of London 10(2): 112–165, pls. 5–6; 10(3): 167–211, pls. 7–8 http://mollus.oxfordjournals.org/content/10/2.toc (free abstract, pdf-download requires subscription of the Journal of Molluscan Studies)

[B253] Hanson A Euliss NH jr Mushet DM (2002) First records of loosely coiled valve snail in North Dakota. The Prairie Naturalist 34(1/2): 63–65. http://digitalcommons.unl.edu/cgi/viewcontent.cgi?article=1151&context=usgsnpwrc

[B254] Harzhauser M Binder H (2004) Synopsis of the Late Miocene mollusc fauna of the classical sections Richardhof and Eichkogel in the Vienna Basin. Archiv für Molluskenkunde 133(1/2): 1–57, 11 pls. www.nhm-wien.ac.at/jart/prj3/nhm/data/uploads/mitarbeiter_dokumente/harzhauser/2004/Harzhauser_Binder_2004_AM_proof.pdf

[B255] Harzhauser M Kowalke T (2002) Sarmatian (Late Middle Miocene) gastropod assemblages of the Central Paratethys. Facies 46: 57–82. http://link.springer.com/article/10.1007%2FBF02668073, doi: 10.1007/BF02668073 (free abstract, download of pdf requires subscription)

[B256] Harzhauser M Kowalke T Mandic O (2002) Late Miocene (Pannonian) gastropods of Lake Pannon with special emphasis on early ontogenetic development. Annalen des Naturhistorischen Museums Wien 103A: 75–141. www.landesmuseum.at/pdf_frei_remote/ANNA_103A_0075-0141.pdf

[B257] Harzhauser M Mandic O (2008) Neogene lake systems of Central and South-Eastern Europe: Faunal diversity, gradients and interrelations. Palaeogeography, Palaeoclimatology, Palaeoecology 260: 417–434. www.yumpu.com/en/document/view/9742037/neogene-lake-systems-of-central-and-south-eastern-europe-; http://www.sciencedirect.com/science/article/pii/S0031018208000151, doi: 10.1016/j.palaeo.2007.12.013 (free abstract, download of pdf requires subscription)

[B258] Hauffen H (1856a) Zwei neue Höhlenschnecken. Verhandlungen des zoologisch-botanischen Vereins in Wien 3: 465–466. www.landesmuseum.at/pdf_frei_remote/VZBG_6_0465-0466.pdf

[B259] Hauffen H (1856b) Zwei neue Schnecken. Verhandlungen des zoologisch-botanischen Vereins in Wien 3: 701–702. www.landesmuseum.at/pdf_frei_remote/VZBG_6_0701-0702.pdf

[B260] Hauswald A-K Albrecht C Wilke T (2008) Testing two contrasting evolutionary patterns in ancient lakes: species flock versus species scatter in valvatid gastropods of Lake Ohrid. Hydrobiologia 615: 169–179. http://link.springer.com/journal/10750/615/1/page/1, doi: 10.1007/s10750-008-9556-0 (free abstract, download of pdf requires subscription)

[B261] Hawe A Heß M Haszprunar G (2013) 3D-reconstruction of the anatomy of the ovoviviparous (?) freshwater gastropod *Borysthenia naticina* (Menke, 1845) (Ectobranchia: Valvatidae). Journal of Molluscan Studies 79(3): 191–204. doi: 10.1093/mollus/eyt018; http://mollus.oxfordjournals.org/content/79/3/191.abstract

[B262] Healy JM (1990) Spermatozoa and spermiogenesis of *Cornirostra*, *Valvata* and *Orbitestella* (Gastropoda, Heterobranchia) with a discussion on valvatoidean sperm morphology. Journal of Molluscan Studies 56(4): 557–566. http://mollus.oxfordjournals.org/content/56/4.toc, doi: 10.1093/mollus/56.4.557 (free abstract, download of pdf requires subscription)

[B263] Hémery M (1956) La grévière du Carrefour d’Aumont en forêt de Compiègne (Oise). Bulletin de la Société préhistorique de France 53(7–8): 424–433. www.persee.fr/web/revues/home/prescript/article/bspf_0249-7638_1956_num_53_7_3359; www.jstor.org/discover/10.2307/27915167?uid=3737864&uid=2&uid=4&sid=21102128483067

[B264] Henderson J (1935) Fossil Non-Marine Mollusca of North America. Geological Society of America Special Papers 3: 1–290. http://specialpapers.gsapubs.org/content/3/1.abstract (free abstract pdf-download requires subscription)

[B265] Herbich F Neumayr M (1875) Beiträge zur Kenntnis fossiler Binnenfaunen. VII. Die Süsswasserablagerungen im südöstlichen Siebenbürgen. Jahrbuch der kaiserlich königlichen Geologischen Reichsanstalt 25(4): 401–431(1–31), pls. 16–17 www.landesmuseum.at/pdf_frei_remote/JbGeolReichsanst_025_0401-0431.pdf

[B266] Hensche A (1866) Dritter Nachtrag zur Mollusken-Fauna Preussen’s. Schriften der königlichen physikalisch-ökonomischen Gesellschaft zu Königsberg 7: 99–106. http://archive.org/stream/schriftenderkn79kn#page/n111/mode/2up

[B267] Hermite H (1879) Études géologiques sur les îles Baléares. Première partie, Majorque et Minorque. Pichon et Savy, Paris, 7–362, pls. 1–5 http://archive.org/stream/etudesgologiq00herm#page/n9/mode/2up (text); http://archive.org/stream/etudesgologiq00herm#page/n371/mode/2up (plates)

[B268] Herrmannsen AN (1847) Indicis generum malacozoorum primordia. Vol. 1 Casellis, Theodor Fischer, 27 + 637 pp. http://archive.org/stream/indicisgenerumma01herr#page/n3/mode/2up

[B269] Hershler R Longley G (1986) Phreatic hydrobiids (Gastropoda: Prosobranchia) from the Edwards (Balcones Fault Zone) Aquifer Region, South-central Texas. Malacologia 27(1): 127–172. http://archive.org/stream/malacologia271986inst#page/126/mode/2up

[B270] Hilgendorf F (1863) Beiträge zur Kenntnis des Süßwasserkalks von Steinheim. Unpublished Dissertation at the Universität Tübingen. Museum für Naturkunde an der Humboldt Universität zu Berlin, Historische Bild und Schriftgutsammlung, Bestand Paläontologisches Museum, Signatur II, Nachlass Hilgendorf, „Steinheim“, Diss. 1863 (reproduced by Glaubrecht (2012), see there) 42 pp.

[B271] Hilgendorf F (1866) *Planorbis multiformis* im Steinheimer Süßwasserkalk. Monatsberichte der königlich Preussischen Akademie der Wissenschaften zu Berlin 1866: 474–504. Also published as reprint (36 pp. + 1 pl.) by W. Weber, Berlin. http://archive.org/stream/monatsberichtede1866knig#page/474/mode/2up (text); http://archive.org/stream/monatsberichtede1866knig#page/n546/mode/1up (plate)

[B272] Hislop S (1859) On the Tertiary deposits, associated with trap-rock, in the East Indies. Quarterly Journal of the Geological Society of London 16: 154–182, pls. 5–10 http://archive.org/stream/quarterlyjournal161860ge#page/154/mode/2up

[B273] Honigmann HL (1909) Verzeichniss der im Zoologischen Museum der Universität Halle befindlichen Goldfuss’schen Mollusken-Lokalsammlung. Zeitschrift für Naturwissenschaften 81(4): 287–300.

[B274] Hovingh P (2004) Intermountain freshwater mollusks, USA (*Margaritifera*, *Anodonta*, *Gonidea*, *Valvata*, *Ferrissia*): geography, conservation, and fish management implications. Monographs of the Western North American Naturalist 2: 109–135. https://ojs.lib.byu.edu/ojs/index.php/wnanmonos/article/viewFile/1461/1794

[B275] Hrubesch K (1965) Die santone Gosau-Landschneckenfauna von Glanegg bei Salzburg, Österreich. Mitteilungen der Bayerischen Staatssammlung für Paläontologie und Historische Geologie 5: 83–120, pls. 5–9 + 10: Fig. 1 www.archive.org/stream/mitteilungenderb5719651967baye#page/n89/mode/2up (text); www.archive.org/stream/mitteilungenderb5719651967baye#page/n875/mode/2up (plates)

[B276] Huckriede R (1967) Molluskenfaunen mit limnischen und brackischen Elementen aus Jura, Serpulit und Wealden NW-Deutschlands und ihre paläogeographische Bedeutung. Beihefte zum Geologischen Jahrbuch 67 Niedersächsisches Landesamt für Bodenforschung, Hannover, 263 pp., 25 pls. http://rogov.zwz.ru/Huckriede,%201967.pdf

[B277] Hudleston WH (1887–1896) British Jurassic Gasteropoda. Supplement. Palaeogeographical Society, London, 453–514, pls. 43–44 http://archive.org/stream/britishjurassicg00hudl#page/n9/mode/2up

[B278] ICZN (1953) Opinion 336. Addition to the “Official List of Specific Names in Zoology” of the species names of one hundred and twenty-two non-marine species of the phylum Mollusca. Opinions and Declarations rendered by the International Commission on Zoological Nomenclature 10(3): 79–108. http://archive.org/stream/opinionsdecla10195556inte#page/78/mode/2up

[B279] ICZN (1955) Opinion 362. Rejection for nomenclatorical purposes of Geoffroy (E.L.), 1767, Traité Sommaire des Coquilles, tant fluviatiles que terrestres, qui se traouvent aux Environs de Paris. Opinions and Declarations rendered by the International Commission on Zoological Nomenclature 11(12): 175–182. http://archive.org/stream/opinionsdecla11195556inte#page/174/mode/2up

[B280] ICZN (1973) Opinon 1108. Conservation of *Marstonia* Baker, 1926 and of *Amniocola lustrica* Pilsbry, 1890 (Mollusca: Gastropoda). Bulletin of Zoological Nomenclature 35(2): 94–96. http://archive.org/stream/bulletinofzoolog35inte#page/94/mode/2up

[B281] ICZN (1992) Opinion 1690 *Helix (Helicigona) barbata* Férussac, 1807 (currently *Lindholmiola barbata*; Mollusca, Gastropoda): lectotype designation confirmed. Bulletin of Zoological Nomenclature 49: 238–239. http://archive.org/stream/bulletinofzoolog49inte#page/238/mode/2up

[B282] ICZN (1999) Opinion 1924 *Helix draparnaudi* Beck, 1837 (currently *Oxychilus draparnaudi *; Mollusca, Gastropoda): specific name conserved. Bulletin of Zoological Nomenclature 56: 152–153. http://archive.org/stream/bulletinofzoolog56inte#page/152/mode/2up

[B283] ICZN (2003) Opinion 2035 (Case 3146) *Valvata minuta* Draparnaud, 1805 (currently *Hauffenia, Neohoratia* or *Islamia minuta*; Mollusca, Gastropoda): conserved by replacement of the lectotype by a neotype. Bulletin of Zoological Nomenclature 60(2): 155–156. http://archive.org/stream/bulletinofzoolog602003int#page/154/mode/2up

[B284] Innes W (1884) Recensement des Planorbes et des Valvées de l’Égypte. Bulletins de la Société Malacologique de France 1: 329–356. http://archive.org/stream/bulletinsdelasoc11884soci#page/328/mode/2up

[B285] Iredale T (1943) A basic list of the freshwater Mollusca of Australia. The Australian Zoologist 10(2): 188–230. http://archive.org/stream/australianzool1021943roya#page/188/mode/2up

[B286] Izzatullaev Z (1977) Новые и малоизвестные пресноводные моллюски Средней Азии [New and little known freshwater molluscs of Middle Asia]. Zoologicheskii Zhurnal 56(6): 948–950. [in Russian, English summary]

[B287] Jakuschina AA

[B288] Jan G (1830) Scientiae naturalis cultoribus. Conspectus methodicus testaceorum in collectione mea exstantium, Parma, 1–8. www.animalbase.uni-goettingen.de/zooweb/servlet/AnimalBase/home/digireference?id=90

[B289] Jeffreys JG (1862) British conchology or an account of the Mollusca which now inhabit the British Isles and the surrounding seas. J. Van Voorst, London Vol. 1: 104 + 341 pp., pls. 1–8 http://archive.org/stream/britishconcholo02jeffgoog#page/n7/mode/2up

[B290] Jekelius E (1932) Die Molluskenfauna der Dazischen Stufe des Beckens von Braşov / Kronstadt. Fauna Neogena a Romaniei. Memoriile Institutului Geologic al României 2: 120 pp., 23 pls., 2 maps.

[B291] Jekelius E (1944) Sarmat und Pont von Soceni (Banat). Memoriile Institutului Geologic al României 5: 1–167, 65 pls. (documented by Danielopol et al. 2009)

[B292] Jelski C (1863) Note sur la faune malacologique des environs de Kieff (Russie). Journal de Conchyliologie 11: 129–137. http://archive.org/stream/journaldeconchyl111863pari#page/128/mode/2up

[B293] Jickeli CF (1874) Fauna der Land- und Süßwasser-Mollusken Nord-Ost Afrikas. Nova Acta der kaiserlich Leopold-Carolingischen Deutschen Akademie der Naturforscher 37(1): 1–352, 11 pls. http://archive.org/stream/faunaderlandunds00jick#page/n5/mode/2up

[B294] Jodot P (1954) Mollusques de petites dimensions des formations continentales ludienne, sannoisienne et stampienne du Jura méridional et de la Haute-Savoie. Annexe par Henri Vincienne. Bulletin de la Société géologique de France (6) 4: 537–555, pl. 23a, b.

[B295] Johannes EJ (2010) Types of *Valvata mergella* Westerlund, 1883 located at the Swedish Museum of Natural History, Stockholm. Tentacle 19: 34–36. http://www.hawaii.edu/cowielab/tentacle/Tentacle_19.pdf

[B296] Johannes EJ (2011) A rare snail lives in Paradise Lake. Water Tenders Fall/winter 2011(1): 8–9. www.watertenders.org/Newsletters/Fall%202011%20newsletter.pdf

[B297] Johanson KA (2002) Systematic revision of American *Helicopsyche* of the subgenus *Feropsyche* (Trichoptera: Helicopsychidae). Insect Systematics and Evolution Supplement 60: 1–146. [Abstract at: http://scanentom.se/publ_ess_60.html]

[B298] Johnson RI Boss KJ (1972) The fresh-water, brackish, and non-Jamaican land mollusks described by C.B. Adams. Occasional Papers on Mollusks 3(43): 193–233 (including pls. 36–42) http://archive.org/stream/occasionalpapers03harv#page/192/mode/2up

[B299] Kabat AR Hershler R (1993) The prosobranch snail family Hydrobiidae (Gastropoda: Rissooidea): Review of classification and supraspecific taxa. Smithsonian Contributions in Zoology 547: 3 +94 pp. www.sil.si.edu/smithsoniancontributions/zoology/pdf_hi/SCTZ-0547.pdf

[B300] Kadolsky D (2008) Mollusks from the Late Oligocene of Oberleichtersbach (Rhön Mountains, Germany). Part 2: Gastropoda: Neritimorpha and Caenogastropoda. Courier Forschungs-Institut Senckenberg 260: 103–138. (pdf-file available on request from the author)

[B301] Kantor YI Vinarski MV Schileyko AA Sysoev AV (2011) Catalogue of the continental mollusks of Russia and adjacent territorries. Version 2.3 330 pp. www.ruthenica.com/documents/Continental_Russian_molluscs_ver2-3.pdf; www.kz-snailhome.narod.ru/books/kantor_2010.pdf

[B302] Kantor YI Vinarski MV Schileyko AA Sysoev AV (2012) Bibliography of the continental mollusks of Russia and adjacent territorries. (Last update March 1, 2012) Version 2.3.1. 55 pp. www.ruthenica.com/documents/Bibliography_continenta_molluscs_ver2-3_1.pdf

[B303] Kapan Yeşilyurt S Taner G (2002) Datça Yarimadasinin Geçpliyosen Pelecypoda ve Gastropoda faunasi ve stratigrafisî (Mugla-Güneybati Anadolu). MTA [Maden Tetkik Arama] Tergisi 125: 89–120, pls. 1–3 [in Turkish] www.mta.gov.tr/v2.0/daire-baskanliklari/bdt/kutuphane/mtadergi/125_6.pdf

[B304] Kennard AS (1911) On *Valvata woodwardi*, n.sp., and Sphaerium bulleni, n.sp. from the Cromerian (Forest bed) of West Runton, Norfolk. Proceedings of the Malacological Society of London 9(5): 324–326. http://mollus.oxfordjournals.org/content/9/5.toc (free abstract, download of pdf requires subscription of the Journal of Molluscan Studies)

[B305] Kennard AS Woodward BB (1926) Synonymy of the British non-marine Mollusca (recent and post-Tertiary). British Museum Natural History, London, 24 + 447 pp. http://archive.org/stream/synonymyofbritis00brit#page/n5/mode/2up

[B306] Kobelt W (1873) Nachträge und Berichtigungen zu meinem Catalog der im europäischen Faunengebiet lebenden Binnenconchylien. Malakozoologische Blätter 21: 177–190. http://archive.org/stream/malakozoologisch2122menk#page/n191/mode/2up

[B307] Kobelt W (1883) Erster Nachtrag zur zweiten Auflage meines Catalogs der im europäischen Faunengebiet lebenden Binnenconchylien. Nachrichtsblatt der Deutschen Malakozoologischen Gesellschaft 15(1–2): 1–25. http://archive.org/stream/nachrichtsblattd151883deut#page/n7/mode/2up

[B308] Koert W (1898) Geologische und paläontologische Untersuchung der Grenzschichten zwischen Jura und Kreide auf der Südwestseite des Selter. Inaugural-Dissertation an der Universität Göttingen, Dieterich Verlag, Göttingen, 57 pp., 13 figs. http://onlinebooks.library.upenn.edu/webbin/book/lookupid?key=ha008423704 (US access only); http://hdl.handle.net/2027/nnc1.cu50515020

[B309] Kókay J (1967) A Bakony-hegység felsőtortonai képződményei (Obertortonische Ablagerungen des Bakonygebirges). Földtani Közlöny / Bulletin of the Hungarian Geological Society 97: 74–90, pls. 7–9 [in Hungarian and German]

[B310] Kókay J (2006) Nonmarine mollusc fauna from the Lower and Middle Miocene, Bakony Mts. W. Hungary. Geologica Hungarica, Series Palaeontologia 56: 196 pp., 14 figs, 41 pls. www.mfgi.hu/sites/default/files/files/K%C3%B6nyvtar/GeolHung_SerPal_teljes/Kokay_GeolHung_56.pdf

[B311] Kolářova L Horák P Skírnisson K (2010) Methodical approaches in the identification of areas with a potential risk of infection by bird schistosomes causing cercarial dermatitis. Journal of Helminthology 84(3): 327–335. doi: 10.1017/S0022149X09990721 (abstract free, pdf-download requires subscription) 20102677

[B312] Kolenati FA (1848) Genera et species *Trichopterorum*. Pars 1 Nouveaux Mémoires de la Société des Naturalistes de Moscou, 141–296, 4 pls. Pragae http://reader.digitale-sammlungen.de/de/fs2/object/display/bsb10231535_00001.html; www.mdz-nbn-resolving.de/urn/resolver.pl?urn=urn:nbn:de:bvb:12-bsb10231535-7

[B313] Kondrashov PE (2007) New gastropod species from the Pleistocene of the Upper Don basin. Paleontological Journal 41(5): 513–519 [Paleontologicheskii Zhurnal, 2007(5), 40–45.] http://link.springer.com/article/10.1134/S0031030107050061 (free abstract, download of pdf requires subscription)

[B314] Kormos T (1911a) Beiträge zur Kenntnis der Pleistozänfauna des Komitates Nyitra. Földtani Közlöni 41, Supplementband: 802–806. http://epa.oszk.hu/01600/01635/00097/pdf/Foldtani_kozlony_EPA01635_1911_11-12_797-850.pdf

[B315] Kormos T (1911b) Ueber die Fauna des Süsswasserkalkes von Mencshely. In: Balaton-Ausschuß der Ungarischen Geographischen Gesellschaft (Eds) Resultate der wissenschaftlichen Untersuchungen des Balatonsees. 1. Band: Physische Geographie des Balatonsees und seiner Umgebung. 1. Teil: Geographische Beschreibung der Balatonsee-Umgebung, samt deren Orographie und Geologie. Anhang: Palaeontologie der Umgebung des Balatonsees (4/9): 1–12. Edition Hölzel, Wien.

[B316] Kozhov / Кожов MM (1936) Моллюски озера Байкал: систематика, распределение, экология, некоторые данные по генезису и истории / Mollyuski ozera Baikala [Mollusks of Lake Baikal - systematic, distribution, ecology, some data on genesis and history]. Trudy Baikal’skoy limnologicheskoy Stantsii / Travaux de la Station Limnologique du Lac Bajkal / Works of the Baikal Limnological Station 8: 1–350. [in Russian, German summary] http://babel.hathitrust.org/cgi/pt?id=mdp.39015070509859 (US access only)

[B317] Kuščer L (1928) (“1926–27“) Drei neue Höhlenschnecken. Glasnik Muzejskega Društva za Slovenijo / Bulletin de l’Association du Musée de Slovenie 7–8: 50–51.

[B318] Kuščer L (1932) Höhlen- und Quellenschnecken aus dem Flußgebiet der Ljubljanica. Archiv für Molluskenkunde 64(2): 48–61.

[B319] Küster HC (1852–1853) Die Gattungen *Paludina, Hydrocaena* und *Valvata*. Systematisches Conchylien-Cabinet von Martini und Chemnitz (1) 21 Bauer und Raspe, Nürnberg, 1–96, pls. 1–77 [for bibliography see Welter-Schultes 1999: 170] http://archive.org/stream/systematischesco121mart#page/n7/mode/2up

[B320] Küster HC (1856) Nachträge und Berichtigungen zu dem Verzeichnisse der Binnenmollusken Bamberg‘s. Ueber das Bestehen und Wirken der naturforschenden Gesellschaft zu Bamberg 3: 73–78. http://archive.org/stream/ueberdasbestehen31856natu#page/72/mode/2up

[B321] Kuster-Wendenburg E (1973) Die Gastropoden aus dem Meeressand (Rupelium) des Mainzer Tertiärbeckens. Abhandlungen des Hessischen Landesamtes für Bodenforschung 67: 170 pp., 8 pls. Abstract at: http://www.hlug.de/fileadmin/shop/pics/schriften/Schriften_Geologie_233.pdf

[B322] LaRoque A (1932) A new variety of *Valvata lewisi* from the Pleistocene of Ontario. Canadian Field Naturalist 46(9): 199 http://archive.org/stream/canadianfieldnat1932otta#page/198/mode/2up

[B323] Lang BZ Dronen NO jr (1970) Eggs and attachment sites for egg capsules of *Valvata lewisi*. The Nautilus 84: 9–12. http://archive.org/stream/nautilus84amer#page/8/mode/2up

[B324] Laubriere L de Carez L (1880) Sur les sables de Brasles (Aisne). Bulletin de la Société Géologique de France (3) 8: 391–413, pls. 15–16 http://goo.gl/NzBRQd

[B325] Lea I (1834) Observations on the Naiades, and descriptions of new species of that and other families. Transactions of the American Philosophical Society (new series) 4: 63–121, pls. 3–18 http://archive.org/stream/transactionsofam04amer#page/62/mode/2up (text with plates interspersed)

[B326] Lea I (1841) Description of new freshwater and land shells. Continuation. Proceedings of the American Philosophical Society held at Philadelphia 2: 11–15, 30–35, 81–83, 224–225, 242–243. http://archive.org/stream/proceedingsofa02amer#page/n17/mode/2up

[B327] Lea I (1856) November 4th 1856, Mr. Lea, Vice-President, in the Chair. Proceedings of the Academy of Natural Sciences of Philadelphia 8: 259–260. http://archive.org/stream/proceedingsofaca08acaduoft#page/258/mode/2up

[B328] Leach WE (1852) Molluscorum Britanniæ synopsis. A Synopsis of the Mollusca of Great Britain. (foreword by J.E. Gray) John van Voorst, London, 376 pp. + 13 pls. http://archive.org/stream/molluscorumbrita00leac#page/n5/mode/2up

[B329] Lechmere Guppy RJ (1864) Descriptions of new species of fluviatile and terrestrial operculate Mollusca from Trinidad. The Annals and Magazine of Natural History: Zoology, Botany, and Geology (3) 14(3): 243–248. http://archive.org/stream/s3annalsmagazine14londuoft#page/242/mode/2up

[B330] Lecointre P Comptesse (1908) Les Faluns de la Touraine. Maison Alfred Mame et Fils, Tours, 111 pp. http://gallica.bnf.fr/ark:/12148/bpt6k881405z

[B331] Leonard AB (1972) A new *Valvata* from the Pleistocene of Southern Illinois. The Nautilus 86: 1–2. http://archive.org/stream/nautilus86amer#page/n7/mode/2up

[B332] Leschke M (1909) Mollusken. Hamburgische Elb-Untersuchung Mitteilungen aus dem naturhistorischen Museum Hamburg 26(2): 249–279. http://archive.org/stream/mitteilungenausd26natu#page/248/mode/2up

[B333] Lesson RP (1832) Histoire naturelle des Mollusques, Annélides et Vers recueillis dans le Voyage autour du Monde de la corvette de Sa Majesté la Coquille pendant les années 1822, 1823, 1824 et 1825 sous le commandement du capitaine Duperrey. Zoologie. Arthus Bertrand, Paris Vol. 2(1): 239–471; Vol. 2(2), 16 pls. https://play.google.com/store/books/details?id=uMNIAAAAcAAJ&rdid=book-uMNIAAAAcAAJ&rdot=1 (Vol. 2(2) = atlas only, requires registration at google-books)

[B334] Letourneux A (1869) Catalogue des Mollusques terrestres et fluviatiles recueillis dans le département de la Vendée et particulièrement dans l’arrondissement de Fontenay le Comte. Revue et magasin de zoologie pure et appliquée (2) 21: 49–64, 105–113, 145–148, 193–203. http://archive.org/stream/revueetmagasinde221pari#page/48/mode/2up

[B335] Leunis J (1860) Synopsis der Naturgeschichte des Thierreichs. Hahn’sche Buchhandlung, Hannover, 1014 pp. http://books.google.de/books?id=0YY9AAAAYAAJ&printsec=frontcover&hl=de&source=gbs_ge_summary_r&cad=0#v=onepage&q&f=false

[B336] Lewis J (1862) Remarks on some species of *Paludina*, *Amnicola*, *Valvata*, and *Melania*. Proceedings of the Academy of Natural Sciences of Philadelphia 14: 587–594. http://archive.org/stream/proceedingsofaca24acaduoft#page/586/mode/2up

[B337] Li YT (1984) 河南凌波盆地早第三纪非海相腹足类化石 - pinyin transcription: Hénán líng bō péndì zǎo dì sān jì fēi hǎi xiàng fù zú lèi huàshí. [Early Tertiary non-marine gastropods from the Lingbao Basin of Henan Province] Professional Papers of Stratigraphy and Palaeontology 11: 1–30, pls. 1–3. http://cpfd.cnki.com.cn/Article/CPFDTOTAL-ZGDJ198400017002.htm (free abstract, download of pdf requires subscription) [in Chinese]

[B338] Li YT (1988) Discovery of fresh-water Gastropoda from Late Cretaceous and Late Palaeocene in Xining-Minhe Basin of Qinghai. Professional Papers of Stratigraphy and Palaeontology 21: 155–169, pls. 1–2. http://en.cnki.com.cn/Article_en/CJFDTOTAL-DCGW198803010.htm (free abstract, download of pdf requires subscription)

[B339] Li YT Sun MR Sun XY (1984) 中国地层学 (13): 中国的第三系 - pinyin transcription: Zhōngguó dìcéng xué (13): Zhōngguó de dì sān xì. [Chinese Stratigraphy (13): The Tertiary System of China]. Geological Publishing House, Beijing, 362 pp. [in Chinese]

[B340] Liess A Quevedo M Olsson J Vrede T Eklöv P Helmut H (2006) Food web complexity affects stoichiometric and trophic interactions. Oikos 114: 117–125. doi: 10.1111/j.2006.0030-1299.14517.x (free abstract, download of pdf requires subscription), http://onlinelibrary.wiley.com/doi/10.1111/oik.2006.114.issue-1/issuetoc

[B341] Liess A Hillebrand H (2005) Stoichiometric variation in C:N, C:P and N:P ratios of littoral benthic invertebrates. Journal of the North American Benthological Society 24: 256–269. www.bioone.org/doi/abs/10.1899/04-015.1, doi: 10.1899/04-015.1 (free abstract, download of pdf requires subscription)

[B342] Lindholm WA (1906) Einige Bemerkungen über die Systematik der Valvatidae. Nachrichtsblatt der Deutschen Malakozoologischen Gesellschaft 38: 187–193. http://archive.org/stream/nachrichtsblattd3738190516deut#page/186/mode/2up

[B343] Lindholm WA (1909) Die Mollusken des Baikal-Sees (Gastropoda et Pelecypoda) systematisch und zoogeographisch bearbeitet. Wissenschaftliche Ergebnisse einer Zoologischen Expedition nach dem Baikal-See unter Leitung des Professors Alexis Korotneff in den Jahren 1900–1902 4: 104 pp., 2 pls. http://archive.org/stream/wissenschaftlich04koro#page/n7/mode/2up

[B344] Lindholm WA (1912) (“1911“) Über Mollusken aus dem Ladogasee und der Nevabucht. Ezhegodnik / Annuaire du Musée Zoologique de l’Académie Impériale des Sciences de St.-Pétersbourg 16: 285–310. http://archive.org/stream/ezhegodnik16zool#page/284/mode/2up

[B345] Lindholm WA (1914) (“1913“) Miszellen zur Malakozoologie des Russischen Reiches. XIII. Ueber die Namen *Dybowskia* Dall und *Jelskia* Bourguignat. Ezhegodnik Zoologicheskago Muzeja Imperatorskoi Akademii Nauk. / Annuaire du Musée zoologique de la Academie Scientifique St. Petersbourg 18(1): 167 http://archive.org/stream/ezhegodnik18zool#page/166/mode/2up

[B346] Lindholm WA (1924) Collectanea baicalia. 1. Archiv für Molluskenkunde 56(6): 217–225.

[B347] Lindholm WA (1927) *Valvata naticina* Menke und ihr Formenkreis. Eine monographische Studie. Archiv für Molluskenkunde 59(1): 20–33.

[B348] Linnaeus C (1758) Systema naturae per regna tria naturae, secundem classes, ordines, genera, species cum characteribus, differentis, synonymis, locis. Vol. 1 Laurentius Salvius, Stockholm, 823 pp. http://archive.org/stream/mobot31753000798865#page/n1/mode/2up

[B349] Locard A (1878) Description de la faune de la mollasse marine et d’eau douce du Lyonnais et du Dauphiné. Archives de Museum d’Histoire Naturelle de Lyon 2 Pitrat Aine, Lyon, 1–284, pls. 18–19 http://archive.org/stream/archivesdumusu02mus#page/n9/mode/2up (text); http://archive.org/stream/archivesdumusu02mus#page/n365/mode/2up (plates)

[B350] Locard A (1881) Études sur les variations malacologiques d’après la faune vivante et fossile de la partie centrale du bassin du Rhône. Tome 2 Henri Georg, Lyon, 560 pp. http://archive.org/stream/tudessurlesvar02loca#page/n7/mode/2up

[B351] Locard A (1883a) Recherches paléontologiques sur les Dépots tertiaires à *Milne-Edwardsia* et *Vivipara* du Pliocène inférieure du Département de l’Ain. Annales de l’Académie de Mâcon. Société des arts, sciences, belles-lettres et agriculture de Saône-et-Loire (2) 6 Macon 166 pp., 4 pls. http://gallica.bnf.fr/ark:/12148/bpt6k2136624.image Remark: Printed date at page 1 of the scanned version is 1888 (as listed by Wenz 1923: 115 and generally in Wenz 1928b), but the paper was already offered with year “1883” in the Journal de Conchyliologie 32(1) available at 1^st^ July 1884. http://archive.org/stream/journaldeconchyl321884pari#page/n114/mode/1up

[B352] Locard A (1883b) Bulletin de la Société des amis des sciences naturelles de Rouen 19 (Procès verbaux, séance du 2 aout 1883): 49 http://babel.hathitrust.org/cgi/pt?id=uc1.b3085992 (US access only) (not seen)

[B353] Locard A (1884) Matériaux pour servir à l’histoire de la malacologie française. Bulletins de la Société Malacologique de France 1: 197–208. http://archive.org/stream/bulletin11884soci#page/196/mode/2up

[B354] Locard A (1889) Contributions à la faune malacologique francaise. XV. Monographie des espéces francaises appartenant au genre *Valvata*. J.-B. Baillière, Paris, 62 pp. http://gallica.bnf.fr/ark:/12148/bpt6k5437006f

[B355] Locard A (1893a) Deuxieme Partie. In: Maillard G Locard A (Eds) Monographie des Mollusques tertiaires terrestres et fluviatiles de la Suisse ..... précédée d’une notice biographique, par M. le Prof. E. Renevier et d’un aperçu statigraphique, par le Prof. A. Jaccard. Mémoirs de la Société Paleontologique Suisse 19: 131–275, pls. 8–12 http://archive.org/stream/MaillardG.1891-MonographieDesMollusquesTertiairesTerrestres/

[B356] Locard A (1893b) Conchyliologie française. Les Coquilles des eaux douces et des eaux saumàtres de France. Description des familles, genres et espèces. Baillière et Fils, Lyon et Paris, 327 pp., 302 figs, http://archive.org/stream/lescoquillesdese00loca#page/n7/mode/2up

[B357] Logan WN (1900) The stratigraphy and invertebrate faunas of the Jurassic formation in the Freezeout Hills of Wyoming. Bulletin of the University of Kansas, Kansas University Quarterly 9(2): 109–134, pls. 25–31 http://archive.org/stream/kansasuniversity09lawr#page/n167/mode/2up (text); http://archive.org/stream/kansasuniversity09lawr#page/134/mode/2up (plates)

[B358] Lomnicki AM (1886) Die tertiäre Süsswasserbildung in Ostgalizien. Verhandlungen der kaiserlich königlichen Geologischen Reichsanstalt 1886: 412–431. www.landesmuseum.at/pdf_frei_remote/VerhGeolBundesanstalt_1886_0412-0431.pdf http://archive.org/stream/verhandlungender1885kkge#page/n873/mode/2up

[B359] Lörenthey E (1894) Die oberen pontischen Sedimente und deren Fauna bei Szegzárd, Nagy-Mányok und Àrpád. Mittheilungen aus dem Jahrbuche der königlichen Ungarischen Geologischen Anstalt 10: 71–160, pls. 3–4.

[B360] Lörenthey E (1902) Die pannonische Fauna von Budapest. Palaeontographica 48(4–5): 137–289, pls. 19–21 http://archive.org/stream/palaeontographic48cass#page/n155/mode/2up

[B361] Lörenthey E (1906) Beiträge zur Fauna und stratigraphischen Lage der pannonischen Schichten in der Umgebung des Balatonsees. In: Magyar földrajzi társaság, Budapest. Balaton-bizottsága / Resultate der wissenschaftlichen Erforschung des Balatonsees. Physische Geographie des Balatonsees und seiner Umgebung. 1. Teil Geographische Beschreibung der Balatonsee-Umgebung, samt deren Orographie und Geologie. 4. Band., Artikel 3. (Hungarian; 1911 in German: I. Band. I, Teil Paläontologie, Anhang 215 pp., pls. 1–3). http://catalog.hathitrust.org/Record/001473276 (US-access only)

[B362] Loriol P de Jaccard A (1865) Étude géologique et paléontologique de la formation d’eau douce infracretacée du Jura et en particulier de Villers-Le-Lac. Ramboz, Schuchardt, Genève, 65 pp., 3 pls. [reprinted in 1866 under the same titel in: Memoires de la Société de physique et d’histoire naturelle de Geneve 18: 63–128, pls. 1–3.] http://archive.org/stream/memoiresdelasoci1866soci#page/n95/mode/2up (text); http://archive.org/stream/memoiresdelasoci1866soci#page/n163/mode/2up (plates)

[B363] Ludwig R (1865) Fossile Conchylien aus den tertiären Süsswasser- und Meerwasser-Ablagerungen in Kurhessen, Grossherzogthum Hessen und der Bayer’schen Rhön. Palaeontographica 14: 40–97, pls. 11–22. http://archive.org/stream/palaeontographic14cass#page/40/mode/2up (text); http://archive.org/stream/palaeontographic14cass#page/n293/mode/2up (plates)

[B364] Luther A (1901) Bidrag till Kännedomen om land- och sötvattengastropodernas utbredning I Finland. Acta Societas Pro Fauna et Flora Fennica 20(3): 125 pp. + 1 map http://archive.org/stream/actasocprofaunae20soci#page/n79/mode/2up

[B365] Maillard G (1884) Invertébrés Purbeckien de Jura. Mémoires de la Société Paléontologique Suisse 11: 156 pp., 3 pls. http://dbooks.bodleian.ox.ac.uk/books/PDFs/N12104444.pdf

[B366] Martens E v (1877) Uebersicht über die von Hilgendorf und Dönitz in Japan gesammelten Binnenmollusken. Sitzungsberichte der Gesellschaft naturforschender Freude zu Berlin 1877: 97–123. http://archive.org/stream/sitzungsberichte1877gese#page/96/mode/2up

[B367] Martens E v (1899) Land and freshwater Mollusca. In: Godman FD Salvin O (Eds) Biologia Centrali-Americana: Zoology, botany and archaeology. Taylor, Francis, London, 369–472. www.sil.si.edu/DigitalCollections/bca/navigation/bca_06_00_00/bca_06_00_00select.cfm

[B368] Martini FHW (1767) Des Herrn Geoffroy ... kurze Abhandlung von den Conchylien, welche um Paris sowohl auf dem Lande, als in süssen Wassern gefunden werden. Index, Nachricht, Systematic Overview, 1 table Raspe, Nürnberg, 133 pp. http://archive.org/stream/desherrngeoffroy00geof#page/n5/mode/2up

[B369] Martins UR (1968) Monografia da tribo Ibidionini (Coleoptera, Cerambycinae). Parte II Arquivos de Zoologia, São Paulo 16(2): 321–630. http://www.revistas.usp.br/azmz/article/view/11937/13714

[B370] Martinson GG (1956) Opredelitel’ Mezozoiskikh i Kainozoiskikh presnovodnykh mollyuskov vostochnoi Sibiri [Handbook of Mesozoic and Cenozoic Freshwater Mollusca of Eastern Siberia]. Izdatel’stvo Akademiia Nauk SSSR, Moscow, 92 pp. + 16 pls. [in Russian]

[B371] Martinson GG (1961) Mezozoyskiye i kaynozoyskiye molluski kontinental’nykh otlozhenii. Sibirskoy platformy, Zabaykal’ya i Mongolii [Mesozoic and Caenozoic Mollusca of continental deposits from Siberia Platform, Zabaykalia and Mongolia]. Papers of the Baikal Limnological Station, Siberia Branch, Academy of Sciences USSR 19: 1–332, pls. 1–26 [in Russian] http://rogov.zwz.ru/Martinson,%201961.pdf

[B372] Martinson GG (1982) [The Upper/Late Cretaceous mollusks of Mongolia (systematics, stratigraphy, taphonomy)]. Sovmestnaya Sovetsko-Mongol’skaya Paleontologicheskaya Ekspeditsiya Trudy = Transactions of the Joint Soviet Mongolian Paleontological Expedition 17, Izdatstvo “Nauka”, Moscow 1–82, pls. 1–16 [in Russian]

[B373] Meek FB (1873) Paleontological report. Annual Report of the U.S. Geological and Geographic Survey of the Territories Washington 6: 429–520. http://archive.org/stream/annualreport1st106geol#page/430/mode/2up

[B374] Meek FB Hayden FV (1856) Descriptions of new species of Acephala and Gasteropoda from the Tertiary Formations of Nebraska Territory, with some general remarks on the geology of the country about the sources of the Missouri River. Proceedings of the Academy of Natural Sciences of Philadelphia 8: 111–126. http://archive.org/stream/proceedingsofaca08acad#page/n129/mode/2up

[B375] Meek FB Hayden FV (1860) Systematic catalogue, with synonyms etc., of Jurassic, Cretaceous and Tertiary fossils collected in Nebraska, by the exploring expeditions und the command of Lieut. Warren, of U.S. Topographic Engineers. Proceedings of the Academy of Natural Sciences of Philadelphia 12: 417–432. http://archive.org/stream/proceedingsofaca12acad#page/416/mode/2up

[B376] Meek FB Hayden FV (1865) Palaeontology of the Upper Missouri: A report upon collections made principally by the expedition under command of Lieut. G.K. Warren, U.S. Top. Engrs., in 1855 and 1856. Invertebrates. Smithsonian Contributions to Knowledge 14(5): 7 + 158 pp., 5 pls. http://archive.org/stream/smithsoniancontr141865smi#page/n197/mode/2up

[B377] Meek FB Hayden FV (1876) A report on the Invertebrate Cretaceous and Tertiary Fossils of the Upper Missouri Country. Report of the United States Geological Survey of the Territories, Washington 9: 15 + 629 pp., 45 pls. http://archive.org/stream/reportofunitedst09geol#page/n5/mode/2up

[B378] Megerle von Mühlfeld JC (1824) Beschreibung einiger neuen Conchylien. Fortsetzung. Verhandlungen der Gesellschaft Naturforschender Freunde zu Berlin 1(4): 205–221, pls 7–9 (in print numbered 1–3) https://play.google.com/store/books/details?id=8OoTAAAAQAAJ&rdid=book-8OoTAAAAQAAJ&rdot=1 (requires registration at google-books)

[B379] Meijer T (1990) Two new freshwater molluscan species from the early Quaternary of the Netherlands. Contributions to Tertiary and Quaternary Geology 27(4): 107–112. www.academia.edu/754929/Two_new_freshwater_molluscan_species_from_the_early_Quaternary_of_the_Netherlands

[B380] Menke KT (1830) Synopsis methodica molluscorum generum omnium et specierum earum, quae in Museo Menkeano adservantur; cum synonymia critica et novarum specierum diagnosibus. Editio altera, auctior et emendatior. G. Uslar, Pyrmonti [Germany], 16 + 169 pp. http://archive.org/stream/synopsismethodic00menk#page/n3/mode/2up

[B381] Menke KT (1845) Kritische Übersicht der lebenden *Valvata* - Arten. Zeitschrift für Malakozoologie 1845/August: 115–130. http://archive.org/stream/zeitschriftfrm15184448menk#page/n327/mode/2up

[B382] Menke KT (1848) Geographische Uebersicht der um die Molluskenfauna Deutschlands verdienten Schriften, Kenner und Sammler. Zeitschrift für Malakozoologie 54(4): 49–78. http://archive.org/stream/zeitschriftfrm15184448menk#page/48/mode/2up

[B383] Menke KT (1849) Literatur. Zeitschrift für Malakozoologie 6(11): 161–167. http://archive.org/stream/zeitschriftfrm610184953menk#page/n177/mode/2up

[B384] Menzel H (1904a) Zwei neue Arten von *Valvata* Müller (Gruppe *Cincinna* Hübner). Nachrichtsblatt der Deutschen Malakozoologischen Gesellschaft 36(2): 77–79, textfigures http://archive.org/stream/nachrichtsblattd3536190314deut#page/76/mode/2up

[B385] Menzel H (1904b) Zwei neue Arten von *Valvata* Müller (Gruppe *Cincinna* Hübner). Berichtigung. Nachrichtsblatt der Deutschen Malakozoologischen Gesellschaft 36(2): 96 http://archive.org/stream/nachrichtsblattd3536190314deut#page/96/mode/2up

[B386] Menzel H (1904c) Beiträge zur Kenntnis der Quartärbildungen im südlichen Hannover. 1. Die Interglazialschichten von Wallensen in der Hilsmulde. Mit einem Anhang: zwei neue Arten von *Valvata* Müller (Gruppe *Cincinna* Hübner). Jahrbuch der königlich preußischen Geologischen Landesanstalt und Bergakademie zu Berlin für das Jahr 1903, Vol. 24: 254–289, pl. 14 http://archive.org/stream/geologischesjah01bodegoog#page/n270/mode/2up (text); http://archive.org/stream/geologischesjah01bodegoog#page/n1004/mode/2up (plate)

[B387] Merian P (1849) Ueber die im Süsswasserkalke der Umgebungen von Mülhausen aufgefundenen Schalthiere. Bericht über die Verhandlungen der naturforschenden Gesellschaft in Basel 8: 33–35. http://archive.org/stream/berichtberdiev08natu#page/32/mode/2up

[B388] Michaud A-L-G (1831) Complément de l’histoire naturelle des mollusques terrestres et fluviatiles de la France, de J. P. R. Draparnaud. Lippmann, Verdun, 1–6, 9–15, 1–116, 1–12, pls. 14–16 http://gdz.sub.uni-goettingen.de/dms/load/img/?PPN=PPN615631045&IDDOC=560860, http://archive.org/stream/histoirenaturell00drap#page/n163/mode/2up (text); http://archive.org/stream/histoirenaturell00drap#page/n361/mode/2up (plates)

[B389] Michaud M (1855) Description des coquilles découvertes dans les environmentes de Hauterive (Drôme). Annales de la Société Linnéenne de Lyon (N.S.) 2: 33–64 (1–34), including pls. 4–5 http://archive.org/stream/annalesdelasoci54lyon#page/32/mode/2up

[B390] Milachevich / Milaschewitsch C (1881) Études sur la faune des Mollusques vivants terrestres et fluviatiles de Moscou. Bulletin de la Société impériale des naturalistes de Moscou 56(1): 215–241. http://archive.org/stream/bulletindelas56188112mosk#page/n281/mode/2up

[B391] Miloshevich / Milošević VM (1973) *Valvata (Cincinna) petronijevici* nov. spec. (Gastropoda) iz slatkovodnih sedimenata donjeg pliocena Metohijske kotline. Glasnik Prirodnjačkog muzeja u Beogradu, serija A, geološke nauke / Bulletin of Natural History Museum in Belgrade. Series A, Mineralogy, Geology and Palaeontology 28: 143–149. [in Serbian]

[B392] Miloshevich / Milošević VM (1984) Prilog poznavanju fosilnih Gastropoda iz familije Valvatidae iz slatkovodnih sedimenata Metohijske kotline (neogen). [Contribution to the fossil gastropods from the family Valvatidae from freshwater sediments, the Metohija basin (Neogene)]. Glasnik Prirodnjačkog muzeja u Beogradu, serija A, mineralogija, geologija, paleontologija / Bulletin of Natural History Museum in Belgrade. Series A, Mineralogy, Geology and Palaeontology 39: 167–184 (incl. 1 plate) [in Serbian]

[B393] Miyadi D (1935) Description of three new subspecies of *Valvata* from Nippon. Venus, Japanese Journal of Malacology 5(2–3): 59–62, pl. 3 (pdf-file available on request from the author)

[B394] Montagu G (1803) *Testacea* Britannica, or, Natural history of British shells, marine, land, and fresh-water, including the most minute systematically arranged and embellished with figures. Vol. 1 Whitels, London, 37 + 606 pp., pls. 1–16 http://archive.org/stream/testaceabritanni12mont#page/n5/mode/2up (text); http://archive.org/stream/testaceabritanni12mont#page/n663/mode/2up (plates)

[B395] Montagu G (1808) *Testacea* Britannica, or, Natural history of British shells, marine, land, and fresh-water, including the most minute systematically arranged and embellished with figures. Vol. 2 = Supplement Whitels, London, 6 + 183 pp., pls. 17–30 http://archive.org/stream/testaceabritanni12mont#page/n697/mode/2up (text); http://archive.org/stream/testaceabritanni12mont#page/n889/mode/2up (plates)

[B396] Moquin-Tandon A (1856) Histoire naturelle des mollusques terrestres et fluviatiles de la France contenant des études générales sur leur anatomie et leur physiologie et la description particulière des genres, des espèces et des variétés. Tome Second J.-B. Bailliere, Paris, 1–646, 54 pls. http://archive.org/stream/histoirenaturell02moqu#page/n7/mode/2up (text); http://archive.org/stream/histoirenaturell00moquuoft#page/n7/mode/2up (plates)

[B397] Moore C (1867) On abnormal conditions of secondary deposits when connected with the Somersetshire and South Wales Coal-basin; and on the age of the Sutton and Southerndown Series. The Quarterly Journal of the Geological Society of London 23: 449–568, pls. 14–17 http://archive.org/stream/quarterlyjourna231867geol#page/448/mode/2up

[B398] Mörch OAL (1864) (“1863”) Fortegnelse over de i Danmark forekommende land- og ferskvandsblöddyr. Videnskabelige Meddelelser fra Dansk naturhistorisk Forening i Kjöbenhavn (2) 1863(17–22): 265–367. http://archive.org/stream/mobot31753002559588#page/n269/mode/2up

[B399] Morelet A (1851) Testacea novissima insulae Cubanae et Americae Centralis. Pars II J.-B. Baillière, Paris, 30 pp. http://archive.org/stream/testaceanovissim00more#page/n7/mode/2up

[B400] Morris J (1838) On the deposits containing Carnivora and other Mammalia in the Valley of the Thames. The Magazine of Natural History (n.s.) 2: 539–546. http://archive.org/stream/magazineofnatura12loud#page/538/mode/2up

[B401] Mortillet G de (1863) Coquilles terrestres et d’eau douce des sables blancs á Eléphas primigenius et a silex taillés d’Abbeville. Bulletin de la Société Géologique de France (2) 20: 293–296, an addition (figures of shells of new species at p. 592) http://goo.gl/FHQn00

[B402] Mousson A (1861) Coquilles terrestres et fluviatiles recueillies par M. le Prof. J. R. Roth dans son dernier voyage en Orient. Vierteljahrsschrift der Naturforschenden Gesellschaft in Zürich 6: 1–34, 124–156. http://archive.org/stream/vierteljahrsschr5618601861natu#page/n461/mode/2up (part 1); http://archive.org/stream/vierteljahrsschr5618601861natu#page/124/mode/2up (part 2)

[B403] Mozley A (1934) New fresh-water mollusks from northern Asia. Smithsonian Miscellaneous Collections 92(2)(3253): 7 pp., 1 pl. http://archive.org/stream/smithsonianmisce921935smit#page/n83/mode/2up

[B404] Müller OF (1773) Vermium terrestrium et fluviatilium, seu animalium infusoriorum, helminthicorum et testaceorum, non marinorum, succincta historia. Vol. 1 Havnia et Lipsia, Heinbeck et Faber 38 + 135 + 72 + 8 pp. http://archive.org/stream/vermivmterrestri01ml#page/n3/mode/2up

[B405] Müller OF (1774) Vermium terrestrium et fluviatilium, seu animalium infusoriorum, helminthicorum et testaceorum, non marinorum, succincta historia. Vol. 2. Havnia et Lipsia, Heinbeck et Faber 36 + 214 + 10 pp. http://archive.org/stream/vermivmterrestri02ml#page/n3/mode/2up

[B406] Müller P (1989) Revised and other species of malacofauna from Tihany (Fehérpart) in Hungary. In: Stevanovic PM Nevesskaja LA Marinescu FL Sokac FL Jámbor Á (Eds) Chronostratigraphie und Neostratotypen. Neogen der Westlichen (“Zentrale”) Paratethys, VIII, pl. 1, Pontien. Jugoslawische Akademie der Wissenschaften und Künste; Serbische Akademie der Wissenschaften und Künste (JAZU, SANU), ISBN 86–407–0082–6, Zagreb-Beograd (1990). 558–581 (including plates 1–8)

[B407] Munier-Chalmas E-P-A (1879) Note In: Revue de Géologie pour l’année 1860–1877 et 1878 15: 33–34. http://books.google.fr/books/about/Revue_de_g%C3%A9ologie_pour_l_ann%C3%A9e_1860_18.html?id=nd8sAQAAIAAJ&redir_esc=y (search modus only)

[B408] Munier-Chalmas E-P-A (1884) Miscellanées Palaeontologiques. Annales de Malacologie 1: 323–340, including pls. 7–8 http://archive.org/stream/annalesdemalacol1187084serv#page/322/mode/2up

[B409] Murray HD Roy EC (1968) Checklist of fresh-water and land mollusks of Texas. Sterkiana 30: 25–42.

[B410] Myzyk S (2002) Life cycle of *Valvata cristata* O.F. Müller, 1774 (Gastropoda: Heterobranchia) in the laboratory. Folia Malacologica (Poznan) 10(2): 47–76. www.foliamalacologica.com/index.php?option=com_content&view=article&id=182&catid=39&Itemid=56 (requires registration at Folia Malacologia)

[B411] Myzyk S (2004) Life cycle of *Valvata macrostoma* Mörch, 1864 (Gastropoda: Heterobranchia) in the laboratory. Folia Malacologica (Poznan) 12(3): 111–136. www.foliamalacologica.com/index.php?option=com_content&view=article&id=147&catid=39&Itemid=56 (requires registration at Folia Malacologia)

[B412] Myzyk S (2007) Life cycle of *Valvata piscinalis* O.F. Müller, 1774 (Gastropoda: Heterobranchia) in the laboratory. Folia Malacologica (Poznan) 15(4): 145–174. www.foliamalacologica.com/index.php?option=com_content&view=article&id=94&catid=39&Itemid=56 (requires registration at Folia Malacologia)

[B413] Neave SA (Ed) (1939–1940) Nomenclator Zoologicus. A List of the Genera and Subgenera in Zoology from the Tenth Edition of Linnaeus 1758 to 2004. (I used the electronic Version 0.86, released 2005 at http://uio.mbl.edu/NomenclatorZoologicus/)

[B414] Neumayr M (1869) Beiträge zur Kenntiss fossiler Binnenfaunen. Jahrbuch der kaiserlich königlichen Geologischen Reichsanstalt 19(3): 355–382, pls. 11–14. www.landesmuseum.at/pdf_frei_remote/JbGeolReichsanst_019_0355-0382.pdf http://archive.org/stream/jahrbuchderka191869unse#page/354/mode/2up (text); http://archive.org/stream/jahrbuchderka191869unse#page/n519/mode/2up (plates)

[B415] Neumayr M (1880) Über den geologischen Bau der Insel Kos und über die Gliederung der jungtertiären Binnenablagerungen des Archipels. Denkschriften der Kaiserlichen Akademie der Wissenschaften / Mathematisch-Naturwissenschaftliche Classe 40: 213–314, 2 pls., 1 map http://archive.org/stream/mobot31753002296942#page/n241/mode/2up

[B416] Neumayr M Paul CM (1875) Congerien und Paludinenschichten Slavoniens und deren Faunen. Ein Beitrag zur Descendenz-Theorie. Abhandlungen der kaiserlich königlichen Geologischen Reichsanstalt 7(3): 111 pp., 10 pls. http://www.onread.com/fbreader/1223582/; http://archive.org/stream/diecongerienundp00neumfo#page/n5/mode/2up

[B417] Nevill G (1877) Catalogue of Mollusca in the Indian Museum, Calcutta, Fasciculus E (contains sub-class Gastropoda, order Prosobranchia, sub-order Ctenobranchia, group Taenioglossa). Indian Museum, Calcutta, 4 + 42 pp.

[B418] Nevill G (1878) Mollusca. Scientific results of the second Yarkand Mission 1–21, pl. 1 http://archive.org/stream/scientificresult01indirich#page/n303/mode/2up

[B419] Nevill G (1884) Hand List of Mollusca in the Indian Museum, Calcutta. Part II: Gastropoda, Prosobranchia-Neurobranchia (Cont.). Indian Museum, Calcutta, 10 + 306 pp. http://archive.org/stream/handlistofmollus12nevi#page/n7/mode/2up; http://www.ebooksread.com/authors-eng/indian-museum/hand-list-of-mollusca-in-the-indian-museum-calcutta-volume-2-hci.shtml

[B420] Niero I Bodon M (2011) Prima segnalazione di Borysthenia naticina (Menke, 1845) per la malacofauna italiana (Gastropoda: Heterobranchia: Valvatidae). Bollettino di Malacolgico 47: 138–149. (pdf available on request from the author)

[B421] Nuttall CP (1989) A review of the Tertiary non-marine molluscan faunas of the Pebasian and other island basins of north-west South America. Bulletin of the British Museum of Natural History, Geology 45: 165–371. http://archive.org/stream/bulletinofbritis45brit#page/n173/mode/2up

[B422] Oppenheim P (1890) Neue oder wenig bekannte Binnenschnecken des Neogen im Peloponnes und im südlichen Mittelgriechenland. Zeitschrift der Deutschen Geologischen Gesellschaft Berlin 42(3): 588–592. http://archive.org/stream/zeitschriftderde42deut#page/588/mode/2up

[B423] Oppenheim P (1891) Beiträge zur Kenntnis des Neogen in Griechenland. Zeitschrift der Deutschen Geologischen Gesellschaft Berlin 43(2): 421–487, pls. 26–28 http://archive.org/stream/zeitschriftderd190gesegoog#page/n430/mode/2up (text); http://archive.org/stream/zeitschriftderd190gesegoog#page/n1116/mode/2up (plates)

[B424] Oppenheim P (1892) Ueber einige Brackwasser- und Binnenmollusken aus der Kreide und dem Eocän Ungarns. Zeitschrift der Deutschen Geologischen Gesellschaft Berlin 44(4): 697–818, pls. 21–26 http://archive.org/stream/zeitschriftderd91gesegoog#page/n705/mode/2up (text); http://archive.org/stream/zeitschriftderd91gesegoog#page/n997/mode/2up (plates)

[B425] Oppenheim P (1918) Das Neogen in Kleinasien. I. Teil. Zeitschrift der deutschen Geologischen Gesellschaft Berlin 70: 1–210, pls. 1–12 http://www.schweizerbart.de/papers/zdgg/detail/70/67559/Das_Neogen_in_Kleinasien (Summary only)

[B426] Ostrovskaya RM Sayarova NS Shirokaya AA Sitnikova TYa (2004) The elements of karyotype instability in representatives of Baikalian malacofauna. In: Abstracts of the Conference “Mollusks of the Northeastern Asia and Northern Pacific: Biodiversity, Ecology, Biogeography and Faunal History” compiled by Semenikhina OYa, Dalnauka, Vladivostok, 106–107. http://rfems.dvo.ru/conference_2004/Malacological_Conference_2004_-_Abstracts.pdf

[B427] Ostrovskaya RM Sitnikova TYa Yakovleva YYa Finogenko YA (1996) Poliploidiya i miskoploidiya u baikal’skikh endemichnykh mollyuskov [Polyploidy and mixoploidy in endemic molluscs of Baikal]. In: Gokhman VE Kuznetsova VG (Eds) Kariosistematika bespozvonochnykh zhivotnykh [Karyosystematics of the Invertebrate Animals]. Vol. 3 Moscow Lomonosov State University, 1–88, 54–56. [in Russian, English summary]

[B428] Pacaud J-M Le Renard J (1995) Révision des Mollusques Paléogénes du Bassin de Paris. IV - Liste systématique actualisée. Cossmanniana 3(4): 155–187.

[B429] Paladilhe A (1866–69) Nouvelles miscellanées malacologiques. Savy, Paris, 144 pp. + 6 pls. [Compilation of various articles of the Revue et Magasin de Zoologie pure et appliquée.] http://archive.org/stream/nouvellesmiscell00pala#page/n9/mode/2up

[B430] Paladilhe A (1866) Nouvelles miscellanées malacologiques. II. Espèces inédites, nouvelles ou peu connues du départment de l’Herault - Suite. Revue et Magasin de Zoologie pure et appliquée (2) 18: 168–171. http://archive.org/stream/revueetmagasinde18pari#page/168/mode/2up

[B431] Paladilhe A (1867a) Nouvelles miscellanées malacologiques. III. Le genre *Assiminea*, en France. Revue et Magasin de Zoologie pure et appliquée (2) 19: 38–41. http://archive.org/stream/revueetmagasinde19pari#page/38/mode/2up

[B432] Paladilhe A (1867b) Nouvelles miscellanées malacologiques. IV. Espèces inédites, nouvelles ou peu connues du départment de l’Herault. Revue et Magasin de Zoologie pure et appliquée (2) 19: 42–53. http://archive.org/stream/revueetmagasinde19pari#page/42/mode/2up

[B433] Pallary P (1901a) Apport à la fauna malacologique de l’Egypte et l’Arabie. Bulletin de l’Institut égyptien (4) 2(5), 239–244, 1 pl. [for bibliography see Bank, Menkhorst 2009] http://archive.org/stream/s4bulletin01inst#page/238/mode/2up

[B434] Pallary P (1901b) Sur le Mollusques fossiles terrestres fluviatiles et saumâtres de l’Algérie. Mèmoires de la Société Géologique de France, Paléontologie 22: 1–213, pls. 3–6 (1–4 in reprint) [note: reprinted in 1970 by Swets, Zeitlinger, Amsterdam] [for bibliography see Bank, Menkhorst 2009] http://jubilotheque.upmc.fr/fonds-mempsgf/GM_000922_001/document.pdf?name=GM_000922_001_pdf.pdf

[B435] Pallary P (1904) Quatrième contribution a l’étude de la faune malacologique du nord-ouest de l’Afrique. Journal de Conchyliologie 52(1): 5–58, pls. 1–3 [for bibliography see Bank, Menkhorst 2009] http://archive.org/stream/journaldeconchyl521904pari#page/n11/mode/2up (text); http://archive.org/stream/journaldeconchyl521904pari#page/n419/mode/2up (plates)

[B436] Pallary P (1926) Compléments a la faune malacologique de la Berbérie. Journal de Conchyliogie 70(1): 1–50. [for bibliography see Bank & Menkhorst 2009]

[B437] Palazzi S (1988) Note sugli Omalogyridae Mediterranei e Maderensi. Bollettino Malacologico 24: 101–111.

[B438] Pan HZ (1977) [Mesozoic and Cenozoic fossil Gastropoda from Yunnan]. In: Mesozoic and Cenozoic Fossils from Yunnan 2: 83–152, pls. 1–9 Academia Sinica, Beijing Science Press [in Chinese]

[B439] Pan HZ (1982a) 四川侏罗纪淡水腹足类化石 - pinyin transcription: Sìchuān zhū luó jì dànshuǐ fù zú lèi huàshí. [Sichuan Jurassic freshwater gastropod fossils]. In: Bureau of Geology and Mineral Resources of Sichuan Province (Ed) Continental Mesozoic Stratigraphy and Paleontology in Sichuan Basin of China. Sichuan People’s Publishing House, Chengdu, 425–439. [in Chinese]

[B440] Pan HZ (1982b) [Late Triassic – early Jurassic gastropods from eastern Hunan and Northeastern Guangxi]. Chung-kuo k’o hsüeh yüan Nanching ti chih ku sheng wu yen chiu so chi k’an / Zhong guo ke xue yuan nan jing di zhi gu sheng wu yan jiu suo ji kan / Memoirs of Nanjing Institute of Geology and Palaeontology, Academia Sinica 17: 108–110. [in Chinese, English summary] https://archive.org/stream/zhongguokexueyua17zhon#page/84/mode/2up

[B441] Pan HZ (1983) Jurassic-Cretaceous non-marine gastropods from Shandong Province. Acta Palaeontologica Sinica 22(2): 210–218, pls. 1–2 http://www.doc88.com/p-78247227729.html

[B442] Pan HZ (2012) The sequence and distribution of Cretaceous non-marine gastropod assemblages in China. Journal of Stratigraphy 36(2): 214–226. http://dcxz.chinajournal.net.cn/EditorB/WebPublication/paperDigest.aspx?paperID=DCXZ201202016&isCnki=ck01 (abstract and references only, download of pdf-file requires subscription)

[B443] Pan HZ Wang HJ Yü W (1974) 三叠纪腹 - pinyin transcription: Sān dié jì fù. [Triassic Gastropoda]. In: Handbook of Stratigraphy and Palaeontology from NW-China. Nanjing Institute of Geology and Palaeontology, 454 pp., 202 pls. [in Chinese]

[B444] Pan HZ Xiang GZ (2002) Middle Jurassic - Lower Cretaceous nonmarine gastropods from Chaoshui Basin. Palaeoworld 14: 86–94. http://english.nigpas.cas.cn/sp/Palaeoworldbackup/vol14/201103/P020110303379608745053.pdf

[B445] Pan HZ Xiu L (1998) 西沙群岛软体动物 - pinyin transcription: Xishā qúndǎo ruǎntǐ dòngwù. [Molluscs from Xisha Islands]. Acta Palaeontologica Sinica 37(1): 121–132. [in Chinese]

[B446] Pan HZ Zhu XG (2007) Early Cretaceous non-marine gastropods from the Xiazhuang Formation in North China. Cretaceous Research 28(2): 215–224. http://www.sciencedirect.com/science/article/pii/S0195667107000250 doi: 10.1016/j.cretres.2006.12.001 (free abstract, pdf-download requires subscription)

[B447] Pană I (2000) (“1998”) New taxa of small-sized gastropods from the Lower Cretaceous deposits of South Dobrogea. Revue Roumaine de Géologie, Géophysique et Géographie 42: 69–90, pls. 1–5, 1 table (pdf file available on request from the author)

[B448] Papp A (1939) Untersuchungen an der sarmatischen Fauna von Wiesen. Jahrbuch der kaiserlich königlichen Geologischen Reichsanstalt, 89 (Jahrbuch der Zweigstelle Wien der Reichsstelle für Bodenforschung) 315–355, pls. 9–10 www.landesmuseum.at/pdf_frei_remote/JbGeolReichsanst_89_0315-0355.pdf

[B449] Papp A (1953) (“1951“) Die Molluskenfauna des Pannon im Wiener Becken. Mitteilungen der Geologischen Gesellschaft von Wien 44: 85–222, pls. 1–25 http://www2.uibk.ac.at/downloads/oegg/Band_44_85_222.pdf

[B450] Papp A (1954) (“1952“) Die Molluskenfauna im Sarmat des Wiener Beckens. Mitteilungen der Geologischen Gesellschaft von Wien 45: 1–112, pls. 1–20 http://www2.uibk.ac.at/downloads/oegg/Band_45_1_112.pdf

[B451] Papp A (1974) Die Molluskenfauna der Sarmatischen Schichtengruppe. In: Papp A Marinescu F Senes J (Eds) M5. Sarmatien (sensu E. Suess, 1866). Chronostratigraphie und Neostratotypen: Miozän der zentralen Paratethys 4: 318–427, 19 pls.

[B452] Parodiz JJ (1961) Notes on Valvatidae from early Tertiary of South America, with a new species. The Nautilus 75(1): 16–18. http://archive.org/stream/nautilus75amer#page/16/mode/2up

[B453] Paulucci M (1878) Materiaux pour servir a l’ètude de la Faune Malacologique de l’Italie et de les Iles. F. Savy, Paris, 4 + 51 pp., http://archive.org/stream/matriauxpourse00paul#page/n7/mode/2up

[B454] Pavlović PS (1903) Gra dia za poznavanje tercijara u. Staroj Srbiji. 3. [Contributions to the Tertiary fauna in Old Serbia]. Geološki anali Balkanskoga poluostrva / Annales géologiques de la Péninsule Balkanique 6(1): 155–189, pls. 3–7 [in Serbian] http://archive.org/stream/geoloshkianalib00zavogoog#page/n176/mode/2up (text); http://archive.org/stream/geoloshkianalib00zavogoog#page/n484/mode/2up (plates)

[B455] Pavlović PS (1911) Beiträge zur Fauna der Tertiärablagerungen in Alt-Serbien. Geološki anali Balkanskoga poluostrva / Annales géologiques de la Péninsule Balkanique 6(2): 580–608, pls. 1–6 http://books.google.de/books/about/Geoloshki_anali_Balkanskoga_poluostrva.html?id=qoARAAAAIAAJ&redir_esc=y (search function only)

[B456] Pavlović PS (1928) Donji pont okoline Beograda / Les Mollusques du Pontien inferieur des environs de Beograd Geološki anali Balkanskoga poluostrva / Annales géologiques de la Péninsule Balkanique 9(2): 1–74. [French title from the summary] http://www.rgf.bg.ac.rs/Publikacije/Geoloski%20anali/GABP%2073%20-%202013.pdf

[B457] Pavlović PS (1931) O fosilnoj fauni mekušaca iz Skopske kotline / La faune des mollusques fossiles du bassin de Skoplje Glasnik Skopskog naučnog društva / Bulletin de la Société Scientifique de Skoplje 9, Odeljenje prirodnih nauka 3: 1–28, pls. 1–11 [in Serbian, French summary] (pdf file available on request from the author)

[B458] Pavlović PS (1932) Novi prilošci, za poznovanje fosilne faune iz Kosovske i Metohijsko-Podrimske kotline / Nouvelles données pour la connaissance de la faune fossile de Kosovo Polje, Melokija et Podrimige dans le Serbie du Sud. Vestnik Geoloskog Instituta Kraljevine Jugoslavije / Bulletin du Service Géologique du Royaume de Yougoslavie Beograd 1: 231–250, 2 pls. [in Serbian, French summary]

[B459] Penecke KA (1886) Beiträge zur Kenntnis der Fauna der slavonischen Paludinenschichten. II. Congeria, Pisidum, Cardium und die Gasteropoden. Beiträge zur Paläontologie Österreich-Ungarns und des Orients 4: 15–44, pls. 9–10 (resp. 6–7); www.landesmuseum.at/pdf_frei_remote/BPalOeU_004_0015-0044.pdf, http://archive.org/stream/beitrgezurpal04wien#page/14/mode/2up (text); http://archive.org/stream/beitrgezurpal04wien#page/n271/mode/2up (plates)

[B460] Petit RE (2006) Notes on Sowerby’s The genera of recent and fossil shells (1821–1834). Archives of Natural History 33(1): 71–89. doi: 10.3366/anh.2006.33.1.71; http://www.euppublishing.com/doi/abs/10.3366/anh.2006.33.1.71?journalCode=anh, doi: 10.3366/anh.2006.33.1.71 (free abstract, download of pdf requires subscription)

[B461] Petit RE (2007) Lovell Augustus Reeve (1814–1865): malacological author and publisher. Zootaxa 1648: 1–120. http://www.mapress.com/zootaxa/2007f/zt01648p120.pdf

[B462] Pfeiffer C (1821) Naturgeschichte deutscher Land- und Süßwassermollusken, Vol. 1: 10 + 135 pp. + 8 pls. https://play.google.com/store/books/details?id=4PRAAAAAcAAJ&rdid=book-4PRAAAAAcAAJ&rdot=1 (requires registration at google-books)

[B463] Philippi RA (1836) Enumeratio molluscorum Siciliæ cum viventium tum in tellure tertiaria fossilium, quae in itinere suo observavit. Vol. 1 Berolini, Schropp, 14 + 267 pp., pls. 1–12 http://reader.digitale-sammlungen.de/de/fs2/object/display/bsb10231737_00012.html

[B464] Piaget J (1913) Supplément au catalogue des mollusques du Canton de Neuchâtel. Bulletin de la Société neuchâtelloise des sciences naturelles 39: 74–89 (there 1 plate) http://archive.org/stream/bulletindelasoci3839soci#page/74/mode/2up

[B465] Piaget J (1914) Premières recherches sur les mollusques profonds du Lac de Neuchâtel. Bulletin de la Société neuchâtelloise des sciences naturelles 40: 148–171 (there 1 plate) http://archive.org/stream/bulletindelasoc40soci#page/148/mode/2up

[B466] Picaglia L (1892) Molluschi terrestri e fluviatili viventi nelle provincie di Modena e Reggio. Bullettino della Società malacologica italiana 16: 83–232. http://archive.org/stream/bullettino16soci#page/82/mode/2up

[B467] Pierce HG (1993) The Nonmarine Mollusks of the Late Oligocene - Early Miocene Cabbage Patch Fauna of Western Montana. III. Aquatic Mollusks and Conclusions. Journal of Paleontology 67(6): 980–993. http://www.jstor.org/stable/1306113

[B468] Pierce HG Constenius KN (2001) Late Eocene - Oligocene nonmarine mollusks of the Northern Kishenehn Basin, Montana and British Columbia. Annals of the Carnegie Museum of Natural History 70(1): 1–112.

[B469] Piersanti C (1951) Una nuova specie italica di *Valvata* troglobia, *Valvata pusilla*, Mihi. Bolletino della Società Naturalisti in Napoli Supplement 61 (18): 1–3, 1 pl.

[B470] Pilsbry HA (1899) Description of new species of Mexican land and fresh-water mollusks. Proceedings of the Academy of Natural Sciences, Philadelphia 51: 391–405 (no pls.) http://archive.org/stream/proceedingsofaca51acaduoft#page/390/mode/2up

[B471] Pilsbry HA (1903) Mexican land and freshwater snails. Proceedings of the Academy of Natural Sciences, Philadelphia 55: 761–789, pls. 47–54 http://archive.org/stream/proceedingsofaca55acad#page/760/mode/2up (text); http://archive.org/stream/proceedingsofaca55acad#page/n957/mode/2up (plates)

[B472] Pilsbry HA (1908) *Valvata humeralis californica* n. subsp. The Nautilus 22: 82 http://archive.org/stream/nautilus22amer#page/82/mode/2up

[B473] Pilsbry HA (1916) Note on *Valvata micra* Pils. And Ferr. The Nautilus 30(7): 83–84. http://archive.org/stream/nautilus30amer#page/82/mode/2up

[B474] Pilsbry HA (1934) Pliocene fresh-water fossils of the Kettleman Hills and neighboring Californian oil fields. The Nautilus 48: 15–17. http://archive.org/stream/nautilus48amer#page/14/mode/2up

[B475] Pilsbry HA (1935) Mollusks of the fresh-water Pliocene beds of the Kettleman Hills and neighbouring oil fields, California. Proceedings of the Academy of Natural Sciences of Philadelphia 86: 541–570, textfigs 1–2, pls. 18–23 http://www.jstor.org/discover/10.2307/4064162?uid=3737864&uid=2&uid=4&sid=21101859043141 (free abstract, download of pdf requires subscription of JSTOR)

[B476] Pilsbry HA (1943) The Type of *Euamnicola* Crosse and Fischer. The Nautilus 57(2): 68–69. http://archive.org/stream/nautilus57amer#page/68/mode/2up

[B477] Pilsbry HA Ferriss JH (1906) Mollusca of Southern States. II. Proceedings of the Academy of Natural Sciences of Philadelphia 58: 123–175, pls. 5–9 http://archive.org/stream/proceedingsofaca58acaduoft#page/122/mode/2up (text); http://archive.org/stream/proceedingsofaca58acaduoft#page/n637/mode/2up (plates)

[B478] Poliński V (1929) Limnoloshka ispitivanja Balkanskog Poluostrva. I. Reliktna fauna gasteropoda Ochridskog Jezera / La faune reliquaire des gastéropodes du lac d’Ochrida Glasnik Srpske Kraljevske Akademije, Beograd 137(65): 129–182. [in Serbian, French summary]

[B479] Poliński V (1932) Die reliktäre Gastropodenfauna des Ochrida-Sees. Zoologische Jahrbücher, Abteilung Systematik 62(5–6): 611–666, pls. 7–8 [German translation of Poliński 1929]

[B480] Pollonera C (1888a) Molluschi dello Scioa e della Valle dell’Havash. Bullettino della Società Malacologica Italiana 13: 49–86, pls. 2–3 http://archive.org/stream/bullettino13soci#page/48/mode/2up (text); http://archive.org/stream/bullettino13soci#page/n257/mode/2up (plates)

[B481] Pollonera C (1888b) Molluschi fossili post-Plioceni del Contorno di Torino. Memorie della Reale Accademia delle Scienze di Torino, Classe di Scienze fisiche matematiche naturali (2) 38: 25–56, pl. 38 http://archive.org/stream/memoriedellareal2381888real#page/n61/mode/2up

[B482] Pollonera C (1898) Intorno alcune conchiglie del Friuli. Bollettino dei Musei di Zoología ed Anatomía comparata della Reale Universita di Torino 13(334): 4 pp. http://archive.org/stream/bollettinodeimus1398univ#page/n243/mode/2up

[B483] Ponder WF Avern GJ (2000) The Glacidorbidae (Mollusca: Gastropoda: Heterobranchia) of Australia. Records of the Australian Museum 52(3): 307–353. http://australianmuseum.net.au/Uploads/Journals/17898/1318_complete.pdf, doi: 10.3853/j.0067-1975.52.2000.1318

[B484] Popova SM (1981) Kaynozoyskaya kontinental’naya malakofauna yuga Sibiri i sopredel’nykh territoriy : sistematicheskiy sostav, biostratigrafiya, istoriya malakofauny, paleolimnologiya. / Cenozoic continental malacofauna of the south of Siberia and neighbouring territories: systematic review, history of malacofauna and paleolimnology. Nauka, Moskva, 188 pp. [in Russian]

[B485] Porumbaru R-C (1881) Étude géologique des environs de Craïova, parcours Bucovatzu-Cretzesca. Première partie. Gauthier-Villars, Paris, 42 pp., 10 pls.

[B486] Preston HB (1916) Descriptions of new freshwater shells from Japan. The Annals and Magazine of Natural History (8) 17: 159–163, pl. 9 http://archive.org/stream/ser8annalsmagazi17londuoft#page/158/mode/2up (text); http://archive.org/stream/ser8annalsmagazi17londuoft#page/n509/mode/1up (plate)

[B487] Prié V (2005) Répartition de *Heraultiella exilis* (Paladilhe, 1867) (Gastropoda, Caenogastropoda, Rissooidea). MalaCo 1: 8–9. http://www.journal-malaco.fr/documents/Prie_Malaco1_2005.pdf

[B488] Prozorova LA (1988) Новый вид рода *Cincinna* (Gastropoda, Pectinibranchia) с юга Приморского края. [A new species of the genus *Cincinna* (Gastropoda, Pectinibranchia from the south of the Primorye territory]. Zoologicheskii Zhurnal 67(16): 1736–1738. [in Russian, English summary]

[B489] Prozorova LA Starobogatov YaI (1998) Подрод *Sibirovalvata* рода Cincinna (Pectinibrahnchia, Valvatidae) в России и на сопредельных территориях [Subgenus *Sibirovalvata* of the genus *Cincinna* (Pectinibranchia,Valvatidae) in Russia and adjacent area]. Bulletin of the Russian Far East Malacological Society 2: 54–74. [in Russian, English abstract] http://rfems.dvo.ru/bulletin/vol_002/article_04.pdf

[B490] Prozorova LA Starobogatov YaI (1999) *Cincinna japonica* (Martens, 1877) (Pectinibranchia, Valvatidae) и новый вид рода с Южных Курил. [On *Cincinna japonica* (Martens, 1877) (Pectinibranchia, Valvatidae), with a description of a new species of the genus from the southern Kurile Islands]. Ruthenica 9(1): 51–55. [in Russian, English abstract]

[B491] Pulteney R (1799) Catalogues of the birds, shells, and some of the more rare plants, of Dorsetshire from the New and Enlarged Edition of Mr Hutchins’s History of That County. Edine, London, 102 pp. [According to Animal Base the work was digitized by the British Library with a National License, but not public domain]

[B492] Put AL Polyszcuk VV (1969) Hовi види молюскiв нижньої дiлянки Дунаю [New species of mollusks from the lower section of the Danube]. Dopovidi Akademii Nauk Ukraïny RSR (ser. B) 1969(7): 651–653. (pdf-file available on request from the author) [in Ukrainan, English summary]

[B493] Radoman P (1978) Neue Vertreter der Gruppe Hydrobioidea von der Balkanhalbinsel. Archiv für Molluskenkunde 109(1–3): 27–43, pls. 4–5.

[B494] Radoman P (1985) Hydrobioidea, a superfamily of Prosobranchia (Gastropoda). II. Origin, zoogeography, evolution in the Balkans and Asia Minor. Monographs, Institute of Zoology (Faculty of Science), Beograd, 1(1): 1–173.

[B495] Rambur P (1858–1866) Catalogue systématique des Lépidoptères de l’Andalousie. Librairie de J.B. Baillière, Paris, 412 pp. 32 pls. www.bsb-muenchen-digital.de/~web/web1030/bsb10308626/images/index.html?digID=bsb10308626&pimage=1&v=pdf&nav=0&l=de

[B496] Raspail J (1909) Note sur le gisement du Vouast près Montjavoult. La Feuille des Jeunes Naturalistes (4) 39: 165–172, 195–202. http://archive.org/stream/lafeuilledesjeun39pari#page/n841/mode/2up (part 1); http://archive.org/stream/lafeuilledesjeun39pari#page/n877/mode/2up (part 2)

[B497] Rath E (1986) Beiträge zur Anatomie und Ontogenie der Valvatidae (Mollusca: Gastropoda). Dissertation (unpublished PhD-Thesis) am Institut für Zoologie der Universität Wien, D730: 1–264. (pdf-file available on request from the author)

[B498] Rath E (1988) Organization and systematic position of the Valvatidae. In: Ponder WF (Ed) Prosobranch Phylogeny. Malacological Reviews Suppl 4: 194–204.

[B499] Reeve LA (1859) Elements of Conchology; an introduction to the natural history of shells and of the animals which form them. Vol. 1: 8 + 260 pp. + 21 pls. (for citation problems see Petit (2007: 83ff), in the present paper the citation of the Biodiversity Heritage Library is taken) http://archive.org/stream/elementsofconcho00reev#page/n5/mode/2up

[B500] Reischütz PL Sattmann H (1993) Beiträge zur Nomenklatur der europäischen Binnenmollusken, V. Die Taxa der Hydrobioidea des griechischen Festlands mit valvatoidem Gehäuse und Festlegung eines Lectotypus von *Valvata (Cincinna) hellenica* Westerlund 1898 (Gastropoda: Prosobranchia). Heldia 2: 51–52, Pl. 8a.

[B501] Reuss AE (1868) Paläontologische Beiträge (Zweite Folge). Sitzungsberichte der Kaiserlichen Akademie der Wissenschaften mathematisch-naturwissenschaftlichen Classe 57: 79–109, pls. 1–3 www.landesmuseum.at/pdf_frei_remote/SBAWW_57_0079-0109.pdf, http://archive.org/stream/sitzungsberichte57kais#page/n93/mode/2up (text); http://archive.org/stream/sitzungsberichte57kais#page/108/mode/2up (plates)

[B502] Risso A (1826) Histoire naturelle des principales productions de l’Europe méridionale et particulièrement de celles des environs de Nice et des Alpes Maritimes. Tome quatrième Levrault, Paris, 1–3, 1–7, 1–439, pls. 1–12 http://www.animalbase.uni-goettingen.de/digiref/ref-id-2758.pdf; http://archive.org/stream/histoirenaturell04riss#page/n7/mode/2up (text); http://archive.org/stream/histoirenaturell04riss#page/n457/mode/2up (plates)

[B503] Robinson WI (1915) Two new freshwater gastropods from the Mesozoic of Arizona. The American Journal of Science (4) 40: 649–651. www.ajsonline.org/content/s4-40/240/649.full.pdf (free abstract, download of pdf requires subscription) http://archive.org/stream/americanjourna4401915newh#page/648/mode/2up

[B504] Roffiaen F (1868) Mollusques terrestres et fluviatiles recueillis en Suisse. Annales de la Société malacologique du Belgique 3: 65–84, pl. 1 http://archive.org/stream/annalesdelasoci031868soci#page/n67/mode/2up (text); http://archive.org/stream/annalesdelasoci031868soci#page/n263/mode/2up (plate)

[B505] Rolle F (1860) Die Lignit-Ablagerungen des Beckens von Schönstein in Unter-Steiermark und ihre Fossilien. Sitzungsberichte der kaiserlichen Akademie der Wissenschaften Wien, mathematisch-naturwissenschaftlichen Classe 41: 7–46, pls 1–2 www.landesmuseum.at/pdf_frei_remote/SBAWW_41_0007-0052.pdf http://archive.org/stream/sitzungsberichte41kais#page/6/mode/2up (text); http://archive.org/stream/sitzungsberichte41kais#page/52/mode/2up (plates)

[B506] Rolle F (1861) Über einige neue oder wenig bekannte Mollusken-Arten aus Tertiär-Ablagerungen. Sitzungsberichte der kaiserlichen Akademie der Wissenschaften Wien, mathematisch-naturwissenschaftlichen Classe 44: 205–224, 2 pls. www.landesmuseum.at/pdf_frei_remote/SBAWW_44_0205-0224.pdf http://archive.org/stream/sitzungsberichte441861kai#page/204/mode/2up (text + plates)

[B507] Röpstorf P Riedel F (2004) Deep-water gastropods endemic to Lake Baikal - an SEM study on protoconchs and radulae. Journal of Conchology 38(3): 253–282. http://www.conchsoc.org/node/4725 (abstract only)

[B508] Ross CP (1960) Geology of Glacier National Park and the Flathead region, northwestern Montana. U.S. Geological Survey Professional Paper 296: 1–121. www.nps.gov/history/history/online_books/geology/publications/pp/296/contents.htm, http://pubs.er.usgs.gov/publication/pp296

[B509] Rossmässler EA (1835–1858) Iconographie der Land- und Süsswasser-Mollusken, mit vorzüglicher Berücksichtigung der europäischen noch nicht abgebildeten Arten. Dresden (Arnold) und Leipzig (Costenoble). Vol. 1(1): 6 + 132 pp., Taf. 1–5 (April 1835); (2): 3 + 28 pp., Taf. 6–10 (August 1835); (3): 4 + 33 pp., Taf. 11–15 (March 1836); (4): 4 + 27 pp., Taf. 16–20 (September 1836); (5/6): 6 + 70 pp., Taf. 21–30 (July 1837); Vol. 2(7/8): 4 + 44 pp., Taf. 31–40 (June 1838); (9/10): 4 + 46 pp., Taf. 41–50 (September 1839); (11): 4 + 15 pp., Taf. 51–55 (July 1842); (12): 4 + 37 pp., Taf. 56–60 (September 1844); Vol. 3(13/14): 8 + pp. 1–39, Taf. 61–70 (September 1854); (15/16): 8 + pp. 41–77, Taf. 71–80 (August 1856); (17/18): 8 + pp. 81–140, Taf. 81–90 (December 1858). www.biodiversitylibrary.org/bibliography/16258; http://archive.org/stream/iconographiederl12ross#page/n5/mode/2up [for bibliographic analysis see Bank (1989)]

[B510] Rossmässler EA Kobelt W (1910) Iconographie der Land- und Süsswasser-Mollusken, mit vorzüglicher Berücksichtigung der europäischen noch nicht abgebildeten Arten. (N.F.) 15 Kreidel Verlag, Wiesbaden, 1–84, pls. 391–420 http://archive.org/stream/iconographiederl1518ross#page/n5/mode/2up (text); http://archive.org/stream/iconographiederl1518ross#page/n93/mode/2up (plates)

[B511] Royo Gómez J (1922) El Mioceno continental ibérico y su fauna malacológica. Comisón de Investigaciones Paleontológicas y Prehistóricas. Junta Ampliación Estudios Investigaciones Científicas, Memorias 30 Reprint 2006: Acta Salamandres, Bibliotheca de la Ciencias, 81 1–230. http://books.google.de/books?id=HhZgkoT3DdkC&pg=PP8&lpg=PP10&ots=3cPenna5W3&dq=El+Mioceno+continental+ib%C3%A9rico+y+su+fauna+malacol%C3%B3gica&hl=de (requires subscription at google-books)

[B512] Russell LS (1938) New species of Gastropoda from the Oligocene of Colorado. Journal of Paleontology 12(5): 505–507. http://www.jstor.org/stable/i255601 (free abstract, download of pdf requires subscription of JSTOR)

[B513] Russell LS (1952) Molluscan fauna of the Kishenehn formation, southeastern British Columbia. National Museum of Canada Bulletin 126: 120–141.

[B514] Russell LS (1964) Cretaceous non-marine faunas of Northwestern North America. Life Sciences Contributions, Royal Ontario Museum, University of Ontario 61: 1–21. http://archive.org/stream/cretaceousnonmar00russ#page/n5/mode/2up

[B515] Sacco F (1886) Fauna malacologica delle alluvioni plioceniche del Piemonti. Memorie della Reale Accademia delle Scienze di Torino, Classe di Scienze fisiche matematiche naturali (2) 37(1): 169–206, 2 pls. http://emeroteca.braidense.it/beic_attacc/sfoglia_articolo.php?IDTestata=909&CodScheda=000P&IDT=15&IDV=120&IDF=0&IDA=450

[B516] Saint-Simon MA de (1870) Description d’espèces nouvelles du Midi de la France. Annales de Malacologie 1: 20–33. http://archive.org/stream/annalesdemalacol1187084serv#page/20/mode/2up

[B517] Sandberger F (1858–63) Die Conchylien des Mainzer Tertiär-Beckens. Kreidel Verlag, Wiesbaden, 5 + 4 + 458 pp. + 35 pls. http://reader.digitale-sammlungen.de/resolve/display/bsb10231850.html; https://play.google.com/store/books/details?id=C-9AAAAAcAAJ&rdid=book-C-9AAAAAcAAJ&rdot=1; http://archive.org/stream/dieconchyliendes00sand#page/n5/mode/2up

[B518] Sandberger F (1870–75) Die Land- und Süßwasser-Conchylien der Vorwelt. Kreidel Verlag, Wiesbaden 8 + 1000 pp., 36 pls., 1 table Exact publication dates from Woodward (1906) and Welter-Schultes (2012: 655) are: Lots 1–3: pp. 1–96, pls. 1–12 (1870); Lots 4–5: pp. 97–160, pls. 13–20 (1871); Lots 6–8: pp. 161–256, pls. 21–32 (1872); Lots 9–10: pp. 257–352, pls. 33–36 (1873); Lot 11: pp. I-VIII, 353–1000 (1875). http://129.237.145.244/bivalve/Sandberger/ (text); http://archive.org/stream/dielandundssswas00sand#page/n5/mode/2up (plates)

[B519] Say T (1817) Descriptions of seven species of American fresh water and land shells, not noticed in the systems. Journal of the Academy of Natural Sciences, Philadelphia 1(1): 13–16, 17–18. http://archive.org/stream/journalofacademy01acaduoft#page/n21/mode/2up

[B520] Say T (1821) Description of the univalve shells of the United States. Journal of the Academy of Natural Sciences of Philadelphia 2: 149–178. http://archive.org/stream/journalofacademy21821acad#page/148/mode/2up

[B521] Say T (1824) Appendix, C. Class Mollusca. In: Keating WH (Ed) Narrative of an expedition to the source of the St. Peter’s River . . . under the command of Major Stephen H. Long compiled from the notes of Major Long, Messrs. Say, Keating, and Calhoun, Vol. 2 H. C. Carey, I. Lea, Philadelphia, 256–266, pl. 15 (included in text) http://archive.org/stream/narrativeofexp02keat#page/256/mode/2up

[B522] Say T (1829–31) Descriptions of some new terrestrial and fluviatile shells of North America. In: The New Harmony. Disseminator of use knowledge. II. New Harmony, Indiana 26 pp. http://archive.org/stream/descriptionsofso00sayt#page/n3/mode/2up

[B523] Schlesch H (1925) Beiträge zur Fauna der Land- und Süßwasser-Mollusken Süd-Seelands. Archiv für Molluskenkunde 57(3): 81–94.

[B524] Schlickum WR Puissegur JJ (1978) Die Molluskemfauna der Schichten mit *Viviparus burgundinus* und *Pyrgula nododotiana* von Montagny les Beaume (Dép. Côte d’Or). Archiv für Molluskenkunde 109: 1–26, pls. 1–3.

[B525] Schlickum WR Strauch F (1979) Die Land- und Süsswassermollusken der Pliozänen Deckschichten der rheinischen Braunkohle. Schlickum, Strauch: Systematischer Teil II. Strauch: Auswertung der Fauna. Abhandlungen der Senckenbergischen naturforschenden Gesellschaft 536: 1–144, pls. 1–11.

[B526] Schlosser M (1906) Ueber fossile Land und Süsswassergastropoden aus Central-Asien und China. Annales Musei Nationalis Hungarici 4: 372–405, pl. 10 http://publication.nhmus.hu/pdf/annHNHM/Annals_HNHM_1906_Vol_4_2_372.pdf

[B527] Schlosser M (1907) Ueber Säugetiere und Süsswasser-Gastropoden aus Pliocän-ablagerungen Spaniens und über die natürliche Grenze von Miocän und Pliocän. Neues Jahrbuch der Mineralogie, Geologie und Paläontologie 2: 1–41, 1 pl http://catalog.hathitrust.org/Record/001993844 (U.S.- access only)

[B528] Schlüter F (1838) Kurzgefasstes systematisches Verzeichniss meiner Conchyliensammlung nebst Andeutung aller bis jetzt von mir bei Halle gefundenen Land- und Flussconchylien. Zur Erleichterung des Tausches für Freunde der Conchyliologie zusammengestellt. Gebauer, Halle, 7 + 40 pp. http://archive.org/stream/kurzgefasstessys00schl#page/n7/mode/2up http://129.237.145.244/bivalve/Schluter1838.pdf

[B529] Schmidt FJ (1847) Systematisches Verzeichniss der in der Provinz Krain vorkommenden Land- und Süsswasser-Conchylien, mit Angabe der Fund-Orte. Jos. Blasnik, Laibach, 27 pp. http://resolver.staatsbibliothek-berlin.de/SBB0000810500000000

[B530] Schmidt A (1856) Verzeichniss der Binnenmollusken Norddeutschlands mit kritischen Bemerkungen. Zeitschrift für die Gesammten [sic]Naturwissenschaften 8: 120–159. http://archive.org/stream/zeitschriftfrd08natu#page/120/mode/2up

[B531] Scholz H Glaubrecht M (2010) A new and open coiled *Valvata* (Gastropoda) from the Pliocene Koobi Fora Formation of the Turkana Basin, Northern Kenya. Journal of Paleontology 84(5): 996–1002. www.bioone.org/doi/pdf/10.1666/10-014.1, doi: 10.1666/10-014.1 (free abstract, download of pdf requires subscription)

[B532] Schröter JS (1779) Die Geschichte der Flußconchylien mit vorzüglicher Rücksicht auf diejenigen welche in den thüringischen Wassern leben. Gebauer, Halle, 434 pp., 11 pls. http://archive.org/stream/diegeschichteder00schr#page/n5/mode/2up

[B533] Schütt H (1962) Neue Süßwasser-Prosobranchier Griechenlands. Archiv für Molluskenkunde 91: 157–166. (pdf-copy available on request from the author)

[B534] Schütt H (1980) Zur Kenntnis griechischer Hydrobiiden. Archiv für Molluskenkunde 110: 115–149.

[B535] Schütt H (1984) Känozoische Landschnecken der Türkei (Känozoikum und Braunkohlen der Türkei 25). Archiv für Molluskenkunde 115(4–6): 179–223, 4 pls., 1 map.

[B536] Schütt H (1988) Ergänzungen zur Kenntnis der Molluskenfauna oberpliozäner Süßwasserkonglomerate Syriens. Archiv für Molluskenkunde 118(4/5/6): 129–143.

[B537] Schütt H Kavusan G (1984) Mollusken der miozänen Süßwasserablagerungen in der Umgebung von Harmancik bei Kütahya - Bursa in Nordwestanatolien. Archiv für Molluskenkunde 114(4/6): 217–229.

[B538] Schütt H Velitzelos E (1991) Mollusken aus dem verkieselten Wald von Kerassia im Nordteil der Insel Euboea (Griechenland). Documenta naturae. München 67: 1–19, 2 pls.

[B539] Sclater PN (1862) Catalogue of a Collection of American Birds. N. Trubner, Co., London, 14 + 368 pp. http://archive.org/stream/catalogueofcolle00scla#page/n5/mode/2up

[B540] Senoner A (1884) Cenni Biografici. Giornale di Scienze Naturali il Naturalisti Siciliano, 1884–85, vol. 4: 45–60. http://archive.org/stream/ilnaturalistasic48485soci#page/44/mode/2up

[B541] Sepkoski JJ Jr (2002) A compendium of fossil marine animal genera. Bulletins of American Paleontology 363: 1–560. http://archive.org/stream/bulletinsofameri363pale#page/n3/mode/2up

[B542] Şereflişani H Yildirim MZ Şereflişani M (2009) The gastropod fauna and their abundance, and some physicochemical parameters of Lake Gölbasi (Hatay, Turkey). Turkish Journal of Zoology 33: 287–296. http://journals.tubitak.gov.tr/zoology/issues/zoo-09-33-3/zoo-33-3-5-0806-7.pdf

[B543] Servain G (1880) Étude sur les mollusques recueillis en Espagne et en Portugal. D. Bardin, Saint-Germain, 3 + 172 pp., 8 pls.

[B544] Servain G (1881) Histoire malacologique du Lac Balaton en Hongrie. P. Kliensick, Paris, 5–125. http://archive.org/stream/histoiremalacolo00serv#page/n5/mode/2up

[B545] Servain G (1884) Excursions malacologiques en Bosnie aux environs de Sarajewo et aux sources de la Bosna. Annales de Malacologie 1(4): 341–380. http://archive.org/stream/annalesdemalacol1187084serv#page/340/mode/2up

[B546] Servain G (1888) Aperçu sur la faune des mollusques fluviatiles des environs de Hambourg. Bulletins de la Société Malacologique de France 5: 287–340, pls. 8–9 http://archive.org/stream/bulletinsdelasoc588soci#page/286/mode/2up

[B547] Settepassi F Verdel U (1965) Continental Quaternary Mollusca of Lower Liri Valley (Southern Latium). Geologica Romana 4: 369–452 incl. 29 figs + 2 tables http://tetide.geo.uniroma1.it/dst/grafica_nuova/pubblicazioni_DST/geologica_romana/Volumi/VOL%204/GR_4_369_451_Settepassi%20et%20al.pdf

[B548] Sherborn CD (1922–1931) Index animalium sive index nominum quae ab a.d. MDCCLVIII generibus et speciebus animalium imposita sunt. Sectio secunda a kalendis Ianuariis, MDCCCI usque ad finem Decembris, MDCCCL. British Museum Natural History, London, 136 + 7056, pp. [database www.sil.si.edu/digitalcollections/indexanimalium/TaxonomicNames/].

[B549] Sinzov / Sinzow / Sintzov / Sintsoff IT (1876) Opisanie novych i maloizslědovanych form rakovin iz tredičnych obrazovanij Novorossii [Beschreibung der neuen und wenig bekannten Schalen aus Tertiärablagerungen in der Novorossia / Description of new and less well known shells from Tertiary deposits in the Novorossia]. Zapiski novorussijskago Obščestva estestvoispytatelej / Memoires de la Société des Naturalistes de la Nouvelle-Russie 3(2): 1–4. [in Russian]

[B550] Sinzov / Sinzow / Sintzov / Sintsoff IT (1877) Opisanie novych i maloizslědovanych form rakovin iz tredičnych obrazovanij Novorossii [Beschreibung der neuen und wenig bekannten Schalen aus Tertiärablagerungen in der Novorossia / Description of new and less well known shells from Tertiary deposits in the Novorossia]. Zapiski novorossijskago Obščestva estestvoispytatelej / Memoires de la Société des Naturalistes de la Nouvelle-Russie 4: 61–83, pls. 5–7. [in Russian]

[B551] Sinzov / Sinzow / Sintzov / Sintsoff IT (1880) Opisanie novych i maloizslědovanych form rakovin iz tredičnych obrazovanij Novorossii [Beschreibung der neuen und wenig bekannten Schalen aus Tertiärablagerungen in der Novorossia / Description of new and less well known shells from Tertiary deposits in the Novorossia]. Zapiski novorossijskago Obščestva estestvoispytatelej / Memoires de la Société des Naturalistes de la Nouvelle-Russie 8: 1–16, pl. 8 (pdf-file available from the author) [in Russian]

[B552] Sinzov / Sinzow / Sintzov / Sintsoff IT (1883) Geologičeskoe izslědovanie Bessarabie i prilegajuščej k nej časti chersonskoj guvernii [Geological description of Bessarabia and nearby parts of the Chernons’gouvernments]. Materialii dlja geologii Rossii 11: 1–142 (not seen) [in Russian]

[B553] Sinzov / Sinzow / Sintzov / Sintsoff IT (1884) Opisanie novych i maloizslědovanych form rakovin iz tredičnych obrazovanij Novorossii [Beschreibung der neuen und wenig bekannten Schalen aus Tertiärablagerungen in der Novorossia / Description of new and less well known shells from Tertiary deposits in the Novorossia]. Zapiski novorossijskago Obščestva estestvoispytatelej / Memoires de la Société des Naturalistes de la Nouvelle-Russie 9(3): 1–13, pl. 9 [in Russian]

[B554] Siodiropoulou VTh (2003) Simvolí sti meléti ton limnéon Plio-Plistokenikón gasteropódon, ke dithíron tis lekánis Ptolemaídas (Ditikí Makedonía - Elláda) [Participation in the study of lake Plio-Pleistocene gastropods and clams of the Ptolemaida Basin (Western Macedonia - Greece)]. Unpublished PhD-Thesis, Makedonia, 281 pp. [in Greek, English summary] http://invenio.lib.auth.gr/record/4577/files/gri-2004-216.pdf

[B555] Sitnikova TYa (1983) Система байкальских эндемичных видов рода *Megalovalvata* и некоторые вопросы систематики семейства Valvatidae (Gastropoda, Pectinibranchia) [The system of Baical endemic species of the genus *Megalovalvata* and some problems of taxonomy of the family Valvatidae (Gastropoda, Pectinibranchia)]. Zoologicheskii Zhurnal 62(1): 32–44. [in Russian, English summary]

[B556] Sitnikova TYa Kijashko PV Sysoev AV (2012) Species names of J.-R. Bourguignat and their application in current taxonomy of fresh-water gastropods of the Russian fauna. The Bulletin of the Russian Far East Malacological Society 15/16: 87–116. http://rfems.dvo.ru/bulletin/vol_015_016/article_04.pdf

[B557] Sitnikova TYa Starobogatov YaI Chernogorenko EV (1986) Poд *Borysthenia* (Gastropoda, Valvatidae), eгo cиcтемaтичecкоe лoлoжeниe и видoвoй cocтaв [The genus *Borysthenia* (Gastropoda, Valvatidae): Its systematic position and species composition]. Vestnik Zoologii 1986(1): 9–14. (pdf-file available on request from the author) [in Russian]

[B558] Sitnikova TYa Starobogatov YaI Shirokaya AA Shibanova IV Korobkova NV Adov FV (2004) Брюхоногие моллюски (Gastropoda) [Gastropod molluscs (Gastropoda)]. In: Timoshkin OA (Ed) Аннотированный список фауны озера Байкал и его водосборного бассейна. [Index of animal species inhabiting Lake Baikal and its catchment area. Guides and Keys to Identification of Fauna and Flora of Lake Baikal]. Nauka, Novosibirsk, 1(2): 937–1002. [in Russian]

[B559] Smith BJ (1973) A new species of snail from Lake Pedder, Tasmania, belonging to the family Valvatidae. Journal of the Malacological Society of Australia 2: 429–434.

[B560] Solem A (1961) New Caledonian land and freshwater snails. An annotated check list Fieldiana Zoology 41(3): 413–487. http://archive.org/stream/newcaledonianlan413sole#page/n9/mode/2up

[B561] Soós ML (1934) Az öcsi felsö-pontusi Mollusca-faunája. Állattani Közlem 31(3–4): 183–210.

[B562] Sowerby GB (1821–34) The genera of recent and fossil shells for the use of students in conchology and geology. 265 plates with unnumbered text Petit (2006) should be considered. http://resolver.sub.uni-goettingen.de/purl?PPN639539777 (from a copy of Università di Firenze, Italy, with 950 unnumbered text pages and 133 plates) https://play.google.com/store/books/details?id=dA0AAAAAQAAJ&rdid=book-dA0AAAAAQAAJ&rdot=1 (requires registration at Google-books)

[B563] Sowerby GB (1838) Comparison of *Cyrena*, *Valvata*, and *Unio*, found at Grays, with recent species. The Magazine of Natural History (n.s.) 2: 546–548. http://archive.org/stream/magazineofnatura12loud#page/546/mode/2up

[B564] Starmühlner F (1952) Zur Anatomie, Histologie und Biologie einheimischer Prosobranchier. Österreichische Zoologische Zeitschrift 3(5): 546–590. http://www.landesmuseum.at/pdf_frei_remote/OEZ_03_0546-0590.pdf

[B565] Starobogatov YaI (1972) Новые виды брюхоногих моллюсков из источников и подземных вод Средней Азии. [New species of gastropods from springs and subterranean waters of Middle Asia]. In: Фауна грунтовых вод Средней Азии [Fauna of sediment waters of Middle Asia]. Trudy Zoologicheskogo Instituta (Leningrad), Akademija Nauk SSSR 50: 165–172. [in Russian] http://malacolog.com/files/Starobogatov_1972.pdf

[B566] Starobogatov YaI Prozorova LA Bogatov VV Saenko EM (2004) Моллюски. В кн.: Определитель пресноводных беспозвоночных России и сопредельных территорий. Т. 6 Моллюски, полихеты, немертины. [Molluscs. In: Tsalolikhin S (Ed) Key to freshwater invertebrates of Russia and adjacent lands, Volume 6 Molluscs, Polychaetes, Nemerteans]. Akad. Nauka, St. Petersburg 6–492. [in Russian]

[B567] Starobogatov YaI Streletzkaja EA (1967) Состав и зоогеографическая характеристика пресноводной малакофауны Восточной Сибири и севера Дальнего Востока. Моллюски и их роль в биоценозах и формировании фаун. [Composition and zoogeographical characteristics of freshwater malacofauna of the East Siberia and northern part of the Far East. In: Molluscs and their role in biocoenoses and formation of faunas] Trudy Zoologicheskogo Instituta (Leningrad), Akademija Nauk SSSR 42: 221–268. http://malacolog.com/node/364 (rar – format) [in Russian, English abstract]

[B568] Starobogatov YaI Zatravkin MN (1985) К системе Valvatidae (Gastropoda, Pectinibranchia) южных районов Дальнего Востока СССР. [On the system of Valvatidae (Gastropoda, Pectinibranchia) from the south of the Soviet Far East]. Zoologicheskii Zhurnal 64(8): 1154–1161. (pdf-file available on request from the author) [in Russian, English summary]

[B569] Ştefănescu Sabba (1896) Études sur les Terrains tertiaires de la Roumanie. Contribution à l’étude des faunes sarmatique, pontique et levantine. Mémoirs de la Société Géologique de France 15: 144 pp., 12 pls. http://goo.gl/PKX4IU

[B570] Stein JPEF (1850) Die lebenden Schnecken und Mollusken der Umgegend Berlins. Reimer, Berlin, 8 + 120 pp., 3 pls. https://play.google.com/books/reader?id=WWkYAAAAYAAJ&printsec=frontcover&output=reader&authuser=0&hl=de&pg=GBS.PP5 (free copy)

[B571] Steusloff U (1922) Zwergformen aus dem Kreise der *Valvata piscinalis* (Müll.). Archiv für Molluskenkunde 54: 81–88. http://archive.org/stream/archivfrmollus541922unse#page/n101/mode/2up

[B572] Stevanovich PM Ilyina LB (1982) Cтратiграфия мэотиca Восточнои ceрьии и coceдниx рeгиoнов лo мoллюcкaм. [Maeotian stratigraphy of the eastern Serbia and adjacent regions based on mollusks]. Bulletin de l’Academie Serbe des Sciences et des Arts, Classe des Sciences Techniques 1982(23): 105–136 including 3 pls. (pdf-file available on request from the author) [in Serbian]

[B573] Stoliczka F (1862) Beiträge zur Kenntnis der Molluskenfauna der Cerithien und Inzersdorfer Schichten des ungarischen Tertiärbeckens. Verhandlungen der kaiserlich-königlichen zoologisch-botanischen Gesellschaft in Wien 12: 529–558, pl. 17 www.landesmuseum.at/pdf_frei_remote/VZBG_12_0529-0538.pdf

[B574] Strebel H (1873) Ein Beitrag zur Kenntniss der Fauna mexicanischer Land- und Süsswasser-Conchylien. Abhandlungen auf dem Gebiete der Naturwissenschaften herausgegeben von dem naturwissenschaftlichen Verein Hamburg. G.J. Herbst, Hamburg, 144 pp. + 19 pls. http://archive.org/stream/beitragzurkenntn00stre#page/n5/mode/2up

[B575] Strobel J Strobel P v (1855) Beitrag zur Molluskenfauna von Tirol. Uebersicht der von den Gebrüdern Josef und Peregrin von Strobel in Tirol gesammelten Land-Schnecken, nebst Angabe ihrer Fundorte und ihrer Nord- und Süd-Grenze gegen das Donau- und das Po-Thal. Verhandlungen des Zoologisch-Botanischen Vereins in Wien 5: 153–176. http://archive.org/stream/verhandlungendes555zool#page/152/mode/2up; www.landesmuseum.at/pdf_frei_remote/VZBG_5_0153-0176.pdf

[B576] Strobel P v (1851) Notizie malacostatiche sul Trentino. Malacologia trentina 1: 114 pp. Pavia, Tipografia Fusi e Comp. www.bsb-muenchen-digital.de/~web/web1030/bsb10309013/images/index.html?digID=bsb10309013&pimage=2&v=pdf&nav=0&l=de

[B577] Strong EE Gargominy O Ponder WF Bouchet P (2008) Global diversity of gastropods (Gastropoda; Mollusca) in freshwater. Hydrobiologia 395: 149–166. http://link.springer.com/journal/10750/395/0/page/1, doi: 10.1007/s10750-007-9012-6 (free abstract, download of pdf requires subscription)

[B578] Struckmann C (1880) Die Wealden-Bildungen der Umgegend von Hannover. Eine geognostisch-paläontologisch-statistische Darstellung. Hahn’sche Buchhandlung, Hannover, 122 pp., 5 pls.

[B579] Studer S (1820) Kurzes Verzeichniss der bis jetzt in unserm Vaterlande entdecken Conchylien. Naturwissenschaftlicher Anzeiger der Allgemeinen Schweizerischen Gesellschaft für die Gesamten Naturwissenschaften 3(11): 83–90, 3(12): 91–94. http://babel.hathitrust.org/cgi/pt?id=njp.32101059540615;view=1up;seq=289

[B580] Sturany R (1894) Zur Molluskenfauna der europäischen Türkei. Annalen des k.k. Naturhistorischen Hofmuseums 9(3): 369–390, pls. 18–20 www.landesmuseum.at/pdf_frei_remote/ANNA_9_0369-0390.pdf; http://archive.org/stream/annalendesnaturh09natu#page/368/mode/2up (text); http://archive.org/stream/annalendesnaturh09natu#page/390/mode/2up (plates)

[B581] Sturany R (1900) W.A. Obrutschew’s Mollusken-Ausbeute aus Hochasien. Denkschriften der Kaiserlichen Akademie der Wissenschaften, Mathematisch-Naturwissenschaftliche Classe 70: 17–48, pls. 1–4 www.landesmuseum.at/pdf_frei_remote/DAKW_70_0017-0048.pdf http://archive.org/stream/waobrutschewsmol00stur#page/n5/mode/2up (text + plates)

[B582] Swammerdam J (1737) Biblia Naturae; sive Historia Insectorum, in Classes certas redacta, nec non exemplis, et Anatomico variorum animalculorum examine, aeneisque Tabulis Illustrata. Insertis numerosis rariorum naturae observationibus. Omnia in Lingua Batava. Acc. Praefatio, in qua vitam auctoris descripsit H. Boerhaave. Latinam versionem adscripsit H.D. Gaubius. Vol. 1 Leydae, 362 pp., 16 pls. http://resolver.sub.uni-goettingen.de/purl?PPN483385700

[B583] Taczanowski L (1871–1872) Les Araneides de la Guyane française. Horae Societatis entomologicae rossicae 8: 32–132 resp. (as separate print) 1–101, pls. 3–4 https://play.google.com/books/reader?id=xnFTAAAAcAAJ&printsec=frontcover&output=reader&authuser=0&hl=de&pg=GBS.PP1 (requires registration at google-book)

[B584] Taner G (1973) Denizli bölgesi Neojeninin paleontolojik ve stratigrafik etüdü. Maden Tetnik ve Arama (MTA) Dergisi 82 Ankara, 89–126, 10 pls (photos), 10 pls (drawnings) www.mta.gov.tr/v2.0/daire-baskanliklari/bdt/kutuphane/mtadergi/82_8.pdf

[B585] Tardy M (1883) Nouvelles observationes de sur la Bresse. Bulletin de la Société géologique de France (3) 11: 543–585. http://goo.gl/v6i6Rf

[B586] Tassinari G (1858) Mollusci fluviatilis italici nova species *Valvata agglutinans*. Imola, Galeati, à Forocornelii, 2 pp.

[B587] Tate R (1873) On the palaeontology of Skye and Raasay. The Quarterly Journal of the Geological Society of London 29: 339–350, pl. 12 http://archive.org/stream/quarterlyjourna291873geol#page/n405/mode/2up (text); http://archive.org/stream/quarterlyjourna291873geol#page/n419/mode/2up (plate)

[B588] Tausch L (1886) Ueber die Fauna der nicht marinen Ablagerungen der oberen Kreide des Csingerthales bei Ajka im Bakony (Veszprimer Comitat, Ungarn) und über einige Conchylien des Gosaumergel von Aigen bei Salzburg. Abhandlungen der kaiserlich königlichen Geologischen Reichsanstalt 12: 1–32, pls. 1–3 http://archive.org/stream/abhandlungender12geol#page/n11/mode/2up (text); http://archive.org/stream/abhandlungender12geol#page/n301/mode/2up (plates)

[B589] Taylor DW (1966) Summary of North American Blancan nonmarine mollusks. Malacologia 4(1): 1–172. http://archive.org/stream/malacologia41966inst#page/n13/mode/2up

[B590] Taylor DW Smith GR (1981) Pliocene molluscs and fishes from Northeastern California and Northwestern Nevada. Contributions of the Museum of Paleontology, University of Michigan 25(18): 339–413, 19 pls http://deepblue.lib.umich.edu/handle/2027.42/48508

[B591] Tennison Woods JE (1876) On the freshwater shells of Tasmania. Papers and Proceedings of the Royal Society of Tasmania 1875: 66–82, additional note http://archive.org/stream/papersproceeding1876roya#page/n89/mode/2up

[B592] Thompson W (1840) Catalogue of the land and freshwater Mollusca of Ireland. Annals and Magazine of Natural History 6(1): 16–34. http://archive.org/stream/annalsmagazineof06lond#page/16/mode/2up, doi: 10.1080/03745484009443267

[B593] Tournouër R (1866) Sur les terrains tertiaires de la vallée supérieure de la Saône. Bulletin de la Société Géologique de France (2) 23: 769–804. http://goo.gl/zaBXdX

[B594] Tournouër R (1875) Note sur quelques fossiles d’eau douce recueillis dans le forage d’un puits au fort de Vancia près de Lyon. Bulletin de la Société Géologique de France (3) 3: 741–748, pl. 28 http://archive.org/stream/bulletindelasoc92frangoog#page/n757/mode/2up (with scan-blackouts on each lower left side) http://goo.gl/5SJvea

[B595] Tournouër R (1877) Coquilles fossiles d’eau douce de l’ile de Rhodes. In: Fischer P (Ed) Paléontologie des terraines tertiaires de l’ile de Rhodes. Mémoires de la Société Géologique de France (3) 1(2): 47–58. http://goo.gl/fgcYE8

[B596] Tryon GW (1863) Descriptions of new species of fresh water Mollusca, belonging to the families of Amnicolidae, Valvatidae and Limnaeidae; inhabiting California. Proceedings of the Academy of Natural Sciences, Philadelphia 15: 147–150, pl. 1 http://archive.org/stream/proceedingsofaca15acaduoft#page/148/mode/2up (text); http://archive.org/stream/proceedingsofaca15acaduoft#page/144/mode/2up (plate)

[B597] Uhl F (1926) Die *Valvata*-Formen des Weißensees bei Füssen. Archiv für Molluskenkunde 58: 259–267.

[B598] Van Damme D (1984) The freshwater Mollusca of Northern Africa. Distribution, Biogeography and Palaeoecology. Developments in Hydrobiology Vol. 25 Springer Verlag, Berlin, 164 pp.

[B599] Vanatta EG (1915) Two new varieties of *Valvata*. The Nautilus 28(9): 104–105. http://archive.org/stream/nautilus28amer#page/104/mode/2up

[B600] Vidal-Abarca R Suárez ML (1985) Lista faunística y bibliográfica de los moluscos (Gastrópodos y Bivalvia) de las aguas continentales de la Península Ibérica e Islas Baleares. Asociación Española de Limnología. Madrid. Listas de las flora y la fauna de las aguas continentales de la Península Ibérica, nº 2 Madrid, 190 pp.

[B601] Villa A Villa JB (1841) Dispositio systematica conchyliarum terrestrium et fluviatilium quae adservantur in collection fratrum Ant. et Jo. Bapt. Villa conspectu abnormitatum novarumque specierum descriptionibus adjectis. Mediolani, Borroni, Scotti, 63 pp., 2 pls https://play.google.com/books/reader?id=_1ZOAAAAcAAJ&printsec=frontcover&output=reader&authuser=0&hl=de&pg=GBS.PA1 (requires registration at google books)

[B602] Vimpère J (2003) Étude de *Valvata bourguignati* Letourneux 1869 dans son locus typicus en Vendée et son rattachement à *Islamia moquiniana* (Dupuy 1851), (Mollusca : Gastropoda). La Naturaliste Vendéen 3: 111–117. www.naturalistes-vendeens.org/naturalistesvend/nv3-p111-117.pdf

[B603] Vinarski MV Andreeva SI Andreev NI Lazutkina EA Karimov AV (2008) (“2007”) Diversity of gastropods in the inland waterbodies of Western Siberia. Invertebrate Zoology 4(2): 173–183. (year of publication according to a personal note of M.V. Vinarski) www.nature.air.ru/invertebrates/pdf_files/vol4_2/invert4_2_173_183_Vinarski.pdf; pdf-file with “addenda et corrigenda” by M.V. Vinarski available on requestion from the author

[B604] Vinarski MV Glöer P Andreyeva SI Lazutkina EA (2013a) Taxonomic notes on Euro-Siberian molluscs. 5. *Valvata (Cincinna) ambigua* Westerlund, 1873 – a distinct species of the group of *Valvata piscinalis* O.F. Müller, 1774. Journal of Conchology 41(3): 295–303. (pdf-file available on request from the author)

[B605] Vinarski MV Nekhaev IO Glöer P Proschwitz Tv (2013b) Type materials of freshwater gastropod species described by C.A. Westerlund and accepted in current malacological taxonomy: A taxonomic and nomenclatorial study. Ruthenica 23(2): 79–108. www.ruthenica.com/documents/VOL23_Vinarski_et_al_79-108_standard.pdf

[B606] Walker B (1902) A revision of the carinate Valvatas of the United States. The Nautilus 15(11): 121–125. http://archive.org/stream/nautilus15amer#page/120/mode/2up

[B607] Walker B (1906) Notes on *Valvata*. The Nautilus 20(3): 25–32, pl. 1 http://archive.org/stream/nautilus20amer#page/n39/mode/2up

[B608] Walker B (1917) *Valvata tricarinata perconfusa* n.n. The Nautilus 31: 36 http://archive.org/stream/nautilus31amer#page/36/mode/2up

[B609] Walker B (1918) A Synopsis of the Classification of the Fresh-water Mollusca of North America, North of Mexico and a Catalogue of the more recently described species, with notes. Part I: Synopsis. Part II: Catalogue. University of Michigan Publications 6: 213 pp. http://archive.org/stream/synopsisofclassi00walk#page/n9/mode/2up, http://deepblue.lib.umich.edu/bitstream/handle/2027.42/56251/MP006.pdf?sequence=1

[B610] Warén A (1991) New and little known Mollusca from Iceland and Scandinavia. Sarsia 76: 53–124. www.tandfonline.com/doi/abs/10.1080/00364827.1991.10413466 (free abstract, download of pdf requires subscription)

[B611] Wattebled G (1884) Description de Mollusques inédits, recueillis par M. le capitaine Dorr en Cochinchine. Journal de Conchyliologie 32: 125–131, pl. 6. http://archive.org/stream/journaldeconchyl321884pari#page/124/mode/2up (text); http://archive.org/stream/journaldeconchyl321884pari#page/n469/mode/2up (plate)

[B612] Welter-Schultes FW (1999) Systematisches Conchylien-Cabinet von Martini und Chemnitz (1837–1920), bibliography of the volumes in Göttingen. Archives of Natural History 26(2): 157–203. http://www.euppublishing.com.ezp-prod1.hul.harvard.edu/toc/anh/26/2, doi: 10.3366/anh.1999.26.2.157 (free abstract, pdf-download requries subscription)

[B613] Welter-Schultes FW (2012a) European Non-marine Molluscs, a Guide for Species Identification. Planet Poster Editions, Göttingen, A1-A3, 1–679, Q1-Q78.

[B614] Welter-Schultes FW (2012b) Guidelines for the Capture and Management of Digital Zoological Names. Information: Version 1.0 (Juni 2012) GBIF 126 pp. imsgbif.gbif.org/CMS_ORC/?doc_id=2784&download=1; http://community.gbif.org/pg/file/read/22111/

[B615] Welter-Schultes F Audibert C (2013) Under construction: molluscan names and their authorships. Folia Conchyliologica 22: 3–32. http://www.cernuelle.com/download.php?lng=fr

[B616] Welter-Schultes FW Audibert C Bertrand A (2011) Liste des mollusques terrestres et dulcicoles de France continentale (excl. hydroboïdes). Folia Conchyologica 12: 4–44. http://www.cernuelle.com/download.php?lng=fr

[B617] Wenz W (1923) Gastropoda extramarina Tertiaria, Vol. 8, Pars 17: 352 pp. In: Diener C (Ed) Fossilium Catalogus, I: Animalia. published in 11 parts Berlin (For exact dating of the various parts see Bouchet et al. 2006: 365.) http://archive.org/stream/Fossiliumcatalo17n#page/n1/mode/2up

[B618] Wenz W (1928a) Zur Systematik Tertiärer Land- und Süsswasser-Gastropoden VII. Senckenbergiana 10: 120.

[B619] Wenz W (1928b) Gastropoda extramarina Tertiaria, Vol. 8, Pars 38 In: Diener C (Ed) Fossilium Catalogus, I: Animalia. 3387 pp., published in 11 parts Berlin 2231–2502. (For exact dating of the various parts see Bouchet et al. 2005: 365.) pdf-file of Valvatidae-part (2422–2492) [available on request from the author]

[B620] Wenz W (1930) Nomenclature of Tertiary land and fresh water gastropods. Senckenbergiana 12(1): 64–66.

[B621] Wenz W (1942) Die Mollusken des Pliozäns der rumänischen Erdöl-Gebiete als Leitversteinerungen für die Aufschluß-Arbeiten. Senckenbergiana 24: 1–293.

[B622] Westerlund CA (1873) Fauna molluscorum terrestrium et fluviatilium Sveciae, Norvegiae et Daniae = Sveriges, Norges och Danmarks land och sötvatten-mollusker. II. Sötvattenmollusker. Bonnier, Stockholm, 1–3, 1–5, 297–651. http://archive.org/stream/faunamolluscorum112west#page/n307/mode/2up

[B623] Westerlund CA (1877) (“1876”) Sibiriens land- och sötvatten-mollusker. Kungliga / Kongliga Svenska Vetenskaps Akademiens Handlingar (NF) 14(12): 1–111, pl. 1 http://archive.org/stream/kunglsvenskavete14kung#page/n155/mode/2up

[B624] Westerlund CA (1876–1878) Fauna Europaea Molluscorum Extramarinorum Prodromus. Sistens descriptiones systematicas et criticas omnium generum et specierum horum animalium in Europa viventium et hodie cognitarum. Berlingia, Lundae, 1–320. http://archive.org/stream/faunaeuropamol01west#page/n1/mode/2up [fasciculus 1 (1876: pp. 1–160) only, fasciculus 2 (1878: pp. 161–320) not available online]

[B625] Westerlund CA (1879) *Valvata minuta* Drap. - eine biographische Skizze. Nachrichtsblatt der Deutschen Malakozoologischen Gesellschaft 11(2–3): 17–24. https://archive.org/stream/nachrichtsblattd111879deut#page/16/mode/2up

[B626] Westerlund CA (1881) Malakologiska Bidrag. Öfversigt af Kongl. Vetenskaps-akademiens forhandlingar 38(4): 35–69. http://archive.org/stream/fversigtafkong381881kung#page/n197/mode/2up

[B627] Westerlund CA (1883) Noch einige von der Vega-Expedition gesammelte Mollusken. Nachrichtsblatt der Deutschen Malakozoologischen Gesellschaft 15: 164–174. http://archive.org/stream/nachrichtsblattd151883deut#page/164/mode/2up

[B628] Westerlund CA (1885) Land- och Sötwatten-Mollusker isamlade under Vega-Expedition af O. Nordquist och A. Stuxberg. In: Nordenskiöld AE (Ed) Vega-Expeditionens vetenskapliga iakttagelser. Bejer, Stockholm, Vol 4: 143–220, pls. 2–6 http://archive.org/stream/vegaexpeditionen04nord#page/142/mode/2up (text); http://archive.org/stream/vegaexpeditionen04nord#page/n597/mode/2up (plates)

[B629] Westerlund CA (1886) II. Fauna der in der paläarktischen Region lebenden Binnenconchylien. VI. Fam. Ampullaridae, Paludinidae, Hydrobiidae, Melanidae, Valvatidae, Neritidae. Register. Lund H Ohlsson’s Buchdr, 156 pp. + 13 pp. http://archive.org/stream/faunaderinderpal12west#page/n159/mode/2up

[B630] Westerlund CA (1889) Sur la faune malacologique extra-marine de l’Europe arctique. Comptes rendus hebdomadaires des séances de l’Académie des sciences Paris 108: 1315–1317. http://archive.org/stream/comptesrendusheb1081889acad#page/1314/mode/2up

[B631] Westerlund CA (1894) Specilegium Malacologicum. Neue Binnenconchylien aus der Paläarktischen Region. V. Schluss. Nachrichtsblatt der Deutschen Malakozoologischen Gesellschaft 26(11–12): 190–205. http://archive.org/stream/nachrichtsblattd2528189396deut#page/n429/mode/2up

[B632] Westerlund CA (1897a) Synopsis Molluscorum extramarinorum Scandinaviae (Sueciae, Norvegiae, Daniae, Fenniae). Acta Societatis pro Fauna et Flora Fennica 13(7): 238 pp., no pls http://archive.org/stream/synopsismollusco00wests#page/n1/mode/2up

[B633] Westerlund CA (1897b) Beiträge zur Molluskenfauna Russlands. Ezhegodnik zoologicheskago Muzeya Imperatorskoj Akademii Nauk. St.-Petersburg / Annuaire du Musée Zoologique de l’Academie Impériale des Sciences de St.-Pétersbourg 2: 117–143. http://archive.org/stream/ezhegodnikzoolog18972impe#page/n3/mode/2up

[B634] Westerlund CA (1898) Novum Specilegium Malacologicum. Ezhegodnik zoologicheskago Muzeya Imperatorskoj Akademii Nauk. St.-Petersburg / Annuaire du Musée Zoologique de l’Académie Impériale des Sciences de St.-Pétersbourg 3(2): 155–183. http://archive.org/stream/ezhegodnikzoolog18982impe#page/154/mode/2up

[B635] Westerlund CA (1902) Methodus dispositionis conchyliorum extramarinorum in Regione palaearctica viventium, familias, genera, subgenera, et stirpes sistens. Rad Jugoslavenske Akademije Znanosti i Umjetnosti, Matematičko-Prirodoslovni Razred [Yugoslav Academy of Arts and Sciences, mathematic and natural class] 151(32). Zagreb, 82–139

[B636] White CA (1895) The Bear River formation and its characteristic fauna. Bulletin of the United States Geological Survey 128: 13–86, pl. 2–11 https://play.google.com/books/reader?id=XKsPAAAAIAAJ&printsec=frontcover&output=reader&authuser=0&hl=de&pg=GBS.PP1

[B637] Whiteaves JF (1885) Report on the Invertebrata of the Laramie and Cretaceous Rocks of the North-west Territory. Contributions to Canadian Palaeontology. Geological and Natural History Survey of Canada, Palaeontology 1(1). Dawson Bros, Montreal, 89 pp., 6 pls http://archive.org/stream/cu31924003872409#page/n5/mode/2up

[B638] Willmann R (1981) Evolution, Systematik und Stratigraphische Bedeutung der Neogenen Süsswassergastropoden von Rhodos und Kos/Ägäis. Palaeontographica 174(1–6): 10–235, pls. 1–13.

[B639] Winckworth R (1936) Journal de Conchyliologie: Dates of Publication. Journal of Molluscan Studies 22(3): 153–156. http://mollus.oxfordjournals.org/content/22/3.toc (free abstract, download of pdf requires subscription)

[B640] Wohlbereth O (1909) Zur Fauna Montenegros und Nordalbaniens (Mollusken, Käfer, Isopoden, Chilopoden). Wissenschaftliche Mitteilungen aus Bosnien und der Herzegowina 11: 585–722, pls. 47–57 http://archive.org/stream/zurfaunamonteneg00wohl#page/n9/mode/2up (text); http://archive.org/stream/zurfaunamonteneg00wohl#page/138/mode/2up (plates)

[B641] Woodward BB (1906) On the dates of publication of C. L. F. von Sandberger‘s ‚Die Land- und Süsswasser-Conchylien der Vorwelt‘, 4to, Wiesbaden (C. W. Kreidel), 1870–1875. Proceedings of the Malacological Society of London 7(1): 3–4. http://mollus.oxfordjournals.org.3632.emedia1.bsb-muenchen.de/content/7/1.toc (free abstract, download of pdf requires subscription of Journal of Molluscan Studies)

[B642] Wüst E (1901) Untersuchungen über das Pliozän und das Älteste Pleistozän Thüringens, nördlich vom Thüringer Walde und westlich von der Saale. Abhandlungen der naturforschenden Gesellschaft Halle 23: 19–368, 9 pls [short version at http://archive.org/stream/untersuchungen00ws#page/n1/mode/2up]

[B643] Wüst E (1912) *Valvata woodwardi* Kennard = *Valvata goldfussiana* Wüst. Nachrichtsblatt der Deutschen Malakozoologischen Gesellschaft 44: 21–22. http://archive.org/stream/nachrichtsblattd4344191112deut#page/20/mode/2up

[B644] Xinjiang Dizhi Ju [fide Zoological Record and Nomenclator Zoologicus: there is no author given, the meaning is « Geology of Xinjang Province »] (1984) Dizhi Diaocha Dadui. Xibei Diqu gu shengwu tuce: Xinjiang Weiwuer Dizhi Qu fence [Atlas of fossils in Xinjiang Province]. Dizhi Chuban She, Beijing Vol. 3: 7 +211 pp., 81 pls (not seen) [in Chinese]

[B645] Yakushina / Jakuschina AA (1991) Rannemelovye mollyuski Severo-Vostoka Mongol’skoy Narodnoy Respubliki (Severo-Choybalsanskiy rayon) [Early Cretaceous mollusks of the north east Mongolian People’s Republic (North Choibalsan region)]. Ezhegodnik Vsesoyuznogo Paleontologicheskogo Obshchestva / Annual of the all-union Paleontological Society 34: 63–76 including pls. 1–3 [in Russian]

[B646] Yen TC (1946) Late Tertiary fresh-water mollusks from Southeastern Idaho. Journal of Paleontology 20(5): 485–494, pl. 76 http://www.jstor.org/stable/i255649 (free abstract, download of pdf requires subscription to JSTOR)

[B647] Yen TC (1947) Pliocene fresh-water mollusks from Northern Utah. Journal of Paleontology 21(3): 268–277, pl. 43 http://www.jstor.org/stable/i255653 (free abstract, download of pdf requires subscription to JSTOR)

[B648] Yen TC (1950a) Fresh-water mollusks of Cretaceous age from Montana and Wyoming. Geological Survey Professional Paper 223A: 1–20, pls. 1–2 http://www.dtic.mil/cgi-bin/GetTRDoc?AD=ADA296486

[B649] Yen TC (1950b) A molluscan fauna from the type section of the Truckee Formation. The American Journal of Science 248(3): 180–193, pl. 1 www.ajsonline.org/content/248/3/180.full.pdf (free abstract, download of pdf requires subscription)

[B650] Yen TC (1952) (“1950”) Molluscan fauna from the Morrison Formation. Geological Survey Professional Paper 233B: 21–51, 3–6. http://pubs.usgs.gov/pp/0233b/report.pdf

[B651] Yen TC (1969) Fossile nicht-marine Mollusken-Faunen aus Nordchina. Sitzungsberichte der Österreichischen Akademie der Wissenschaften in Wien, mathematisch-naturwissenschaftliche Klasse, Abteilung 1 177: 21–64, 4 pls. www.landesmuseum.at/pdf_frei_remote/SBAWW_177_0021-0064.pdf

[B652] Yen TC Reeside JB (1946) Fresh-water mollusks from the Morrison Formation (Jurassic) of Sublette County, Wyo. Journal of Paleontology 20(1): 52–58, http://www.jstor.org/stable/i255645 (text figs) (free abstract, download of pdf requires subscription to JSTOR) http://rogov.zwz.ru/Joliaf/Yen%20Teng-chien,%20Reeside,%201946.pdf

[B653] Youluo (i.e. “the editorial group”) (1978) 渤海沿岸地区早第三纪腹足类 in pinyin: Bóhǎi yán’àn dìqū zǎo dì sān jì fù zú lèi / in World Catalog: Bohai yan’an diqu zao disan ji fuzulei [Early Tertiary gastropod fossils from the coastal region of Bohai]. Geological Publishing House, Nanjing, China, 6 + 157 pp., 33 pls. and legends [in Chinese, English summary]

[B654] Yü W (1965) 山西垣曲垣曲羣上部淡水腹足类化石的新材料 (pinyin transcription: Shānxī yuán qū yuán qū qún shàngbù dànshuǐ fù zú lèi huàshí de xīn cáiliào) [New materials of fresh-water Gastropoda from the upper part of the Yuanchü Chun, Yuanchü, Shansi]. Acta Paleontologica Sinica 13(1): 29–57. [in Chinese, 44–54 in English] http://159.226.74.248:8000/pagelinks/14332.pdf

[B655] Yü W (1974) 2. [Early Jurassic gastropods. In: Handbook of Stratigraphy and Palaeontology in Southwest China]. Edited by Nanjing Institute of Geology and Palaeontology, Academia Sinica, Science Press, Beijing, 372–374. [in Chinese]

[B656] Yü W (1977) Cretaceous and Early Tertiary non-marine gastropods and their stratigraphic significance. Acta Paleontologica Sinica 16(2): 191–213, pls. 1–4 http://en.cnki.com.cn/Article_en/CJFDTOTAL-GSWX197702004.htm (free abstract, pdf-download requires subscription)

[B657] Yü W (1982) [Some fossil gastropods from Xizang]. In: The series of the comprehensive scientific expedition to the Qinghai-Xizang Plateau. Palaeontology of Xizang (Tibetan) Plateau 4 Science Press, Beijing, 255–281, pls. 1–10 [in Chinese, English abstract]

[B658] Yü W (1983) [The sequence and distribution of Late Cretaceous and Early Tertiary gastropod assemblages in China]. Zhongguo Kexueyuan Nanjing Dizhi Gushengwu Yanjiusuo Congkau / Bulletin of the Nanjing Institute of Geology and Palaeontology 1983(6): 321–353, pls. 1–3 [in Chinese, English summary]

[B659] Yü W Pan HZ (1980) [Nonmarine Mesozoic Gastropoda in Zhejiang and southern Anhui]. In: Divisions and correlation of the Mesozoic volcano-sedimentary rocks in Zhejiang and Anhui provinces, China. Academia Sinica, Nanking Institute of Geology and Paleontology, Nanking, China, 135–172. (not seen)

[B660] Yü W Pan HZ (1982) 皖南晚白垩世和早第三纪非海相腹足类化石 - pinyin transcription: Wǎnnán wǎn bái’è shì hé zǎo dì sān jì fēi hǎi xiàng fù zú lèi huàshí [Eocene non-marine Gastropoda from Zhou Xian, Hebei]. Zhongguo Kexueyuan Nanjing Dizhi Gushengwu Yanjiusuo Congkau / Bulletin of Nanjing Institute of Geology and Palaeontology 1982(4): 189–212, pls. 1–4 [in Chinese, English summary]

[B661] Yü W Pan HZ Wang HJ (1974) [Triassic Gastropoda]. In: Nanjing Institute of Geology and Palaeontology, Academia Sinica (Ed) Handbook of Stratigraphy and Palaeontology of Southwestern Region of China. Publishing House of Science, Beijing, 320–326, pls. 169, 171–172 [in Chinese]

[B662] Yü W Pan HZ Wang HJ (1982) [Late Cretaceous and Early Tertiary non-marine gastropods from southern Anhui] Chung-kuo k’o hsüeh yüan Nan-ching ti chih ku sheng wu yen chiu so chi k’an / zhong guo ke xue yuan nan jing di zhi gu sheng wu yan jiu suo ji kan / Memoirs of Nanjing Institute of Geology and Palaeontology, Academia Sinica 17: 1–36 (including pls. 1–5) http://archive.org/stream/zhongguokexueyua17zhon#page/n7/mode/2up [in Chinese, English summary]

[B663] Yü W Wang HJ (1977) 江苏晚白垩世 [?] 新生代腹足类化石 - pinyin transcription: Jiāngsū wǎn bái’è shì ? xīnshēng dài fù zú lèi huàshí [The late Cretaceous and Cenozoic Gastropoda from Jiangsu Province]. Chung-kuo k’o hsüeh yüan Nan-ching ti chih ku sheng wu yen chiu so chi k’an / Zhong guo ke xue yuan nan jing di zhi gu sheng wu yan jiu suo ji kan / Memoirs of Nanjing Institute of Geology and Palaeontology, Academia Sinica 8(5): 1–100 (including pls. 1–9) http://archive.org/stream/zhongguokexueyua08zhon#page/n3/mode/2up

[B664] Yü W Zhang XQ (1982) 广东三水盆地晚白垩世和早第三纪非海相腹足类化石 - pinyin transcription: Guǎngdōng sān shuǐ péndì wǎn bái’è shì hé zǎo dì sān jì fēi hǎi xiàng fù zú lèi huàshí [Late Creatceous and Early Tertiary non-marine gastropods from Sanshui Basin, Giangdong] Chung-kuo k’o hsüeh yüan Nan-ching ti chih ku sheng wu yen chiu so chi k’an / Zhong guo ke xue yuan nan jing di zhi gu sheng wu yan jiu suo ji kan / Memoirs of Nanjing Institute of Geology and Palaeontology, Academia Sinica 17: 37–84 (including pls. 1–8) http://archive.org/stream/zhongguokexueyua17zhon#page/36/mode/2up [in Chinese, English summary pp. 69–73]

[B665] Yü XH (1987) Late Jurassic and Early Createous fresh-water gastropods (Mollusca) from Western Liaoning Province, China. In: Yu Xihan Wang Wuli Liu Xianting Zhang Wu et al. (Eds) Mesozoic Stratigraphy and Palaeontology of Western Liaoning Vol. 3: 28–116. (including plates) (Not seen)

[B666] Zhu XG (1980) [Phylum Mollusca] [in Chinese] In: Paleontological Atlas of Northwest China. Vol 2: Mesozoic and Cenozoic. Geological Publishing House, Beijing 8–59. (Not seen)

[B667] Zhu XG (1985) 甘肃西丽盆地晚新生代非海相腹足类化石 - pinyin transcription: Gānsù xi lì péndì wǎn xīnshēng dài fēi hǎi xiàng fù zú lèi huàshí [Late Cenozoic non-marine gastropods from Xili Basin in Gansu]. Acta Palaeontologica Sinica 24(6): 672–679, pls. 1–2 [in Chinese, English summary] http://en.cnki.com.cn/Article_en/CJFDTotal-GSWX198506011.htm (free English abstract, pdf-download of full paper requires subscription)

[B668] Zhu XG (1994) 山东侏罗纪白垩世非海相腹足类化石 - pinyin transcription: Shāndōng zhū luó jì bái’è shì fēi hǎi xiàng fù zú lèi huàshí [Jurassic-Cretaceous nonmarine gastropods from Northern Xinjiang]. Acta Paleontologica Sinica 33(1): 85–105, pls. 1–4 [in Chinese, extended English summary and English species diagnoses] http://en.cnki.com.cn/Article_en/CJFDTOTAL-GSWX198302010.htm (free English abstract, download of full paper requires subscription) (pdf-file available on request by the author)

[B669] Zieten CH von (1830–1833) Die Versteinerungen Württembergs oder naturgetreue Abbildungen der in den vollständigsten Sammlungen, namentlich der in dem Kabinet des Oberamts-Arzt Dr. Hartmann befindlichen Petrefacten: Mit Angabe der Gebirgs–Formationen, in welchen dieselben vorkommen und der Fundorte. [German/French] 4 Vols., 12 Issues, 72 pls Verlag, Lithographie der Expedition des Werkes unserer Zeit, Stuttgart www.e-rara.ch/zut/content/pageview/4206543

